# The E3 Ligase RNF115 Aggravates Pathological Cardiac Hypertrophy via Ubiquitin‐Mediated Degradation of SPTBN1

**DOI:** 10.1002/advs.76077

**Published:** 2026-06-15

**Authors:** Yan Zu, Shiqing Chen, Ji Chen, Liuliu Ruan, Bowen Li, Weijian Chu, Shichen Huang, Dian Yu, Jing Tian, Yuhan Zhao, Yi Han, Xin Tang, Yong Ji

**Affiliations:** ^1^ Key Laboratory of Drug Targets and Translational Medicine For Cardio‐cerebrovascular Diseases Medical Basic Research Innovation Center For Cardiovascular and Cerebrovascular Diseases Key Laboratory of Targeted Intervention of Cardiovascular Disease Ministry of Education Collaborative Innovation Center For Cardiovascular Disease Translational Medicine Nanjing Medical University Nanjing China; ^2^ State Key Laboratory of Frigid Zone Cardiovascular Diseases (SKLFZCD) Heilongjiang Provincial Key Laboratory of Cardiovascular Disease and Molecular Intervention Harbin Medical University Harbin China; ^3^ Department of Critical Care The Second Affiliated Hospital of Harbin Medical University Harbin China; ^4^ The First School of Clinical Medicine The First Affiliated Hospital of Nanjing Medical University Nanjing China

**Keywords:** cardiac hypertrophy, cardiomyocytes, RNF115, ubiquitination

## Abstract

Pathological cardiac hypertrophy represents an adaptive alteration in cardiac structure and function and serves as a critical process in heart failure. Ubiquitination, a classical post‐translational modification that regulates protein function and degradation, plays a crucial role in cardiac hypertrophy. In this study, the E3 ubiquitin ligase ring finger protein 115 (RNF115) is identified as a key pro‐hypertrophic factor. RNF115 expression is significantly elevated in heart tissues from patients with heart failure, in mice subjected to transverse aortic constriction (TAC) surgery, and in cardiomyocytes treated with angiotensin II (Ang II). RNF115 knockdown attenuates Ang II‐induced cardiomyocyte hypertrophy in vitro, whereas cardiomyocyte‐specific RNF115 knockout protects against TAC‐induced cardiac hypertrophy and dysfunction in vivo. Mechanistically, upregulation of RNF115 promotes the ubiquitination and degradation of spectrin β, non‐erythrocytic 1 (SPTBN1), causing filamentous actin (F‐actin) depolymerization, thereby inactivates the Hippo‐Yes associated protein (YAP) pathway and drives pro‐hypertrophic gene expression. DTD (dithiocarbamate disulfide derivatives) is a potent inhibitor of RNF115 that suppresses SPTBN1 degradation and exerts a protective role in cardiac hypertrophy, indicating that inhibition of RNF115 may represent a potential therapeutic strategy for pathological cardiac hypertrophy and heart failure.

## Graphical abstract

In response to hypertrophic stimuli, increased c‑JUN phosphorylation upregulates RNF115, leading to SPTBN1 ubiquitination and degradation. which promotes F‑actin depolymerization and YAP activation, driving cardiac hypertrophy. The RNF115 inhibitor DTD effectively suppresses SPTBN1 ubiquitination and cardiac hypertrophy.

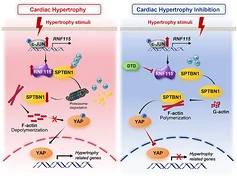

## 1 Introduction

Cardiovascular diseases exhibit substantial morbidity and mortality worldwide in which heart failure (HF) being the major cause of death [1, 2]. Pathological cardiac hypertrophy, characterized by ventricular dilation and systolic dysfunction, is considered a critical process in the clinical course of HF [3]. However, the underlying mechanisms of cardiac hypertrophy remain unclear [4]. Recently, the potential of post‐translational modifications of key proteins involved in cardiomyocyte pathophysiology has received increasing attention [5, 6]. Maintaining the homeostasis of key proteins may play an essential role in the pathological cardiac hypertrophy.

Ubiquitination is a dynamic post‐translational modification that regulates protein homeostasis through proteasome‐mediated degradation or other non‐degradative signaling, participating in the progression of various cardiovascular diseases [7, 8, 9]. The E3 ubiquitin ligases (E3s) are primarily classified into three types: Homologous to the E6‐AP C‐Terminus‌ (HECT) E3s, Really Interesting New Gene (RING)‐type E3s, and RING‐Between‐RING (RBR) E3s [10]. They confer substrate specificity by recognizing and binding target proteins, initiating the critical step of proteasomal degradation [11]. Our previous studies found that neuronal precursor cell expressed developmentally downregulated 4 (NEDD4)‐mediated S‐nitrosoglutathione reductase (GSNOR) degradation aggravates cardiac hypertrophy [12], while SMAD specific E3 ubiquitin ligase 2 (SMURF2) inhibition protects cardiac function by reducing axis inhibition protein 1 (AXIN1) degradation [13]. These studies have underscored the importance of HECT E3s (NEDD4/SMURF2) in cardiac function regulation. Recently, we have focused on the role of the RING family—another important E3 ubiquitin ligase family, in cardiovascular diseases.

RING E3s are the most abundant type of ubiquitin ligases, mediating direct transfer of ubiquitin to substrates. Previous studies revealed that tripartite motif‐containing protein 16 (TRIM16) reduces cardiac oxidative stress by decreasing peroxiredoxin 1 (PRDX1) phosphorylation [6], and ‌ring finger protein 220 (RNF220) exacerbates cardiac remodeling by stabilizing signal transducer and activator of transcription 3 (STAT3) [14]. These findings demonstrate that RING E3s play a crucial role in maintaining cardiac structure and function.

Ring finger protein 115 (RNF115), also known as breast cancer‐associated gene 2 (BCA2), is an E3 ligase containing a RING finger domain that plays an important role in various pathological processes by ubiquitination of distinct targets [15]. Recent evidence suggests that RNF115 promotes tumorigenesis and malignant cell migration in multiple tumor models [16, 17, 18], but its role in cardiovascular diseases remains poorly understood. Our study identified RNF115 as significantly upregulated in transcriptomic datasets from human and mouse cardiac hypertrophy models, with relatively high basal expression in the heart, suggesting it may be a key factor in cardiac hypertrophy. Notably, Disulfiram (DSF), a compound reported to inhibit RNF115's ubiquitin ligase activity [19], has been shown to suppress RNF115‐mediated ubiquitination, thereby blocking stimulator of interferon genes (STING) overactivation and Golgi translocation, ameliorating autoimmune diseases [20]. These findings highlight the potential of inhibiting RNF115 as a therapeutic target.

This study focused on investigating the function and molecular mechanisms of RNF115 in cardiac hypertrophy. We found that RNF115 was significantly upregulated in hypertrophic models, and cardiomyocyte‐specific knockout of RNF115 effectively attenuated myocardial hypertrophy. Mechanistically, RNF115 interacted with Spectrin β, non‐erythrocytic 1 (SPTBN1) and promoted its ubiquitination and subsequent degradation. This triggered destabilization and enhanced depolymerization of filamentous actin (F‐actin), resulting in sustained yes‐associated protein (YAP) activation, which in turn exacerbated cardiac remodeling and dysfunction. Together, these findings provide the evidence supporting pro‐hypertrophic activity of RNF115, suggesting that targeting RNF115 may serve as a novel therapeutic strategy for pathological cardiac hypertrophy.

## 2 Results

### 2.1 RNF115 is Upregulated in Pathological Cardiac Hypertrophy

**FIGURE 1:**
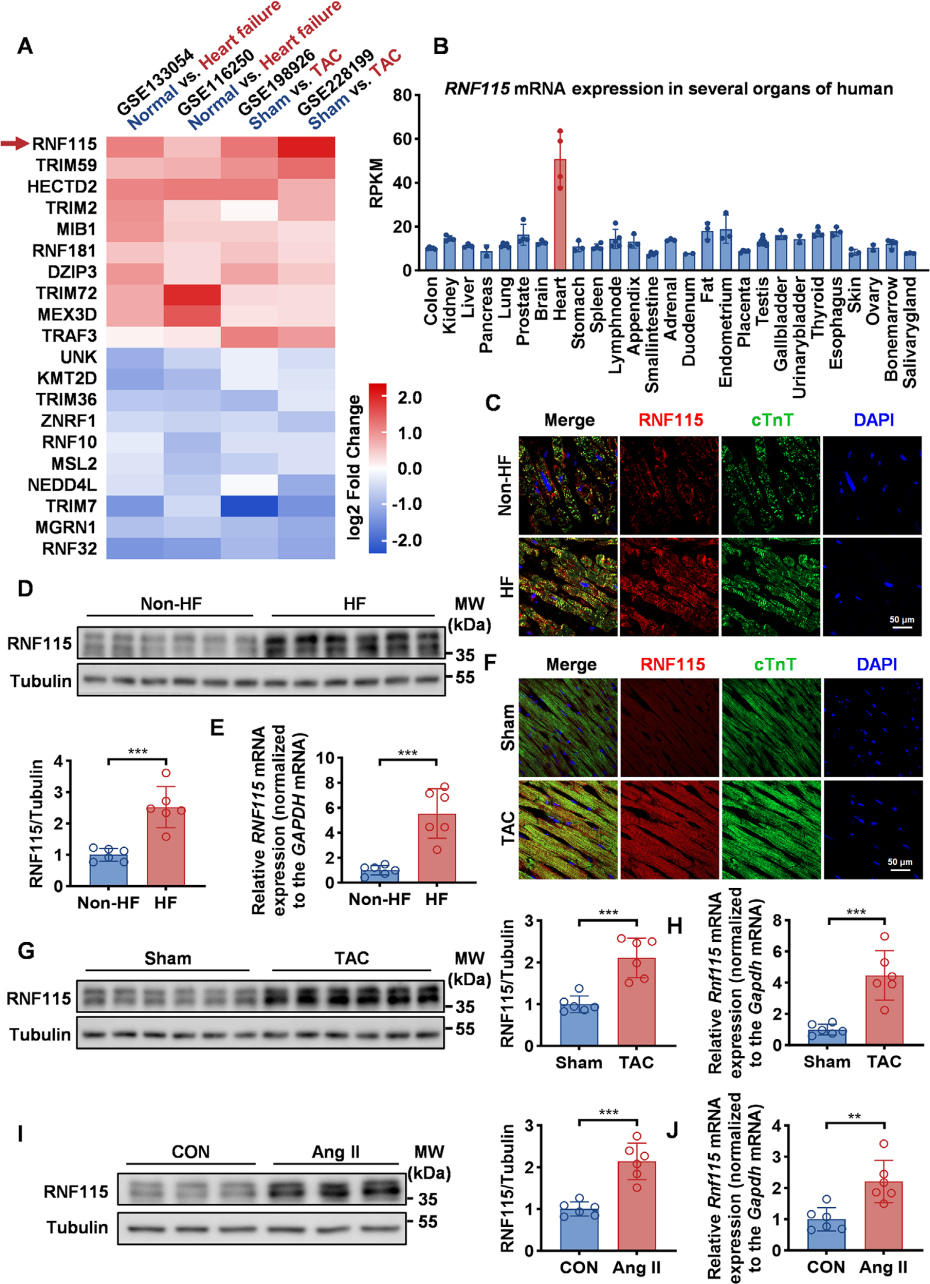
RNF115 is upregulated in pathological cardiac hypertrophy. (A) Heatmap showing differential gene expression of E3 ubiquitin ligases in heart tissues of heart failure or non‐heart failure patients and transverse aortic constriction (TAC) or sham mice in indicated GEO databases. Red and blue represent the log2 fold change in gene expression compared to the control group. (B) The mRNA levels of *RNF115* in several organs of human. Data available in Array Express under accession number E‐MTAB‐1733. (C) Immunofluorescence images of RNF115 (red), cTnT (green), and DAPI (blue) in heart samples of non‐heart failure (Non‐HF) and heart failure (HF) patients (*n* = 3). Scale bar = 50 µm. (D) Immunoblotting analysis and quantitation of RNF115 protein levels in heart samples of Non‐HF and HF patients (*n* = 6). (E) The mRNA levels of *RNF115* in heart samples of Non‐HF and HF patients were determined by quantitative polymerase chain reaction (qPCR) (*n* = 6). (F) Immunofluorescence images of RNF115 (red), cTnT (green), and DAPI (blue) in heart tissues from C57BL/6 mice under sham or TAC surgery (*n* = 3). Scale bar = 50 µm. (G) Immunoblotting analysis and quantitation of RNF115 protein levels in heart tissues from C57BL/6 mice under sham or TAC surgery (*n* = 6). (H) The mRNA levels of *Rnf115* in heart tissues from C57BL/6 mice under sham or TAC surgery were determined by qPCR (*n* = 6). (I) Immunoblotting analysis and quantitation of RNF115 protein levels in neonatal rat cardiomyocytes (NRCMs) treated with angiotensin II (Ang II) or vehicle for 48 h (*n* = 6). (J) The mRNA levels of *Rnf115* in NRCMs treated with Ang II or vehicle were determined by qPCR (*n* = 6). Data shown as mean ± SD, **p < 0.01, ***p < 0.001. Statistical analysis assessed by unpaired *t*‐test (D and E, G–J).

We first analyzed a series of published datasets from the Gene Expression Omnibus (GEO) to identify potential key E3 ubiquitin ligases associated with pathological cardiac hypertrophy. Among these, we identified RNF115, a RING‐finger protein, whose mRNA expression exhibited the most significant upregulation in cardiac tissue from heart failure patients and transverse aortic constriction (TAC) mice (Figure 1A). Furthermore, among the top three genes identified in the heatmap, RNF115 was highly expressed in the heart (Figure 1B; Figure S1A, Supporting Information) [21]. Immunofluorescence staining and immunoblotting assays revealed that RNF115 protein levels were significantly elevated in cardiac tissue from heart failure (HF) patients compared to non‐heart failure (Non‐HF) patients (Figure 1C,D). Consistent with changes in protein levels, the mRNA levels of *RNF115* were also increased (Figure 1E). Correlation analysis showed a negative correlation between both RNF115 protein and mRNA expression and left ventricular ejection fraction (LVEF) in these HF and Non‐HF subjects (Figure S1B, Supporting Information). Next, we validated these findings in TAC‐induced cardiac hypertrophy mouse model. Similarly, both protein and mRNA levels of RNF115 were markedly upregulated (Figure 1F–H).

To identify the cellular sources of expression for RNF115 in the heart, we used published interactive open‐access database—the Human Protein Atlas (https://www.proteinatlas.org) and found that RNF115 expression was mainly distributed in cardiomyocytes (Figure S2A, Supporting Information) [22]. Immunofluorescence staining and immunoblotting assays also confirmed higher RNF115 expression in neonatal mouse cardiomyocytes (NMCMs) than non‑cardiomyocytes (Figure 1F; Figure S2B, Supporting Information). Moreover, RNF115 protein levels gradually increased in neonatal rat cardiomyocytes (NRCMs) under angiotensin II (Ang II) stimulation, peaking at 48 h (Figure 1I; Figure S2C,D, Supporting Information), with concurrent mRNA upregulation (Figure 1J). The significant correlation between RNF115 expression and cardiac hypertrophy reveals the key role of RNF115 in the progression of this disease.

### 2.2 Cardiomyocyte‐Specific RNF115 Knockout Attenuates Cardiac Hypertrophy

**FIGURE 2:**
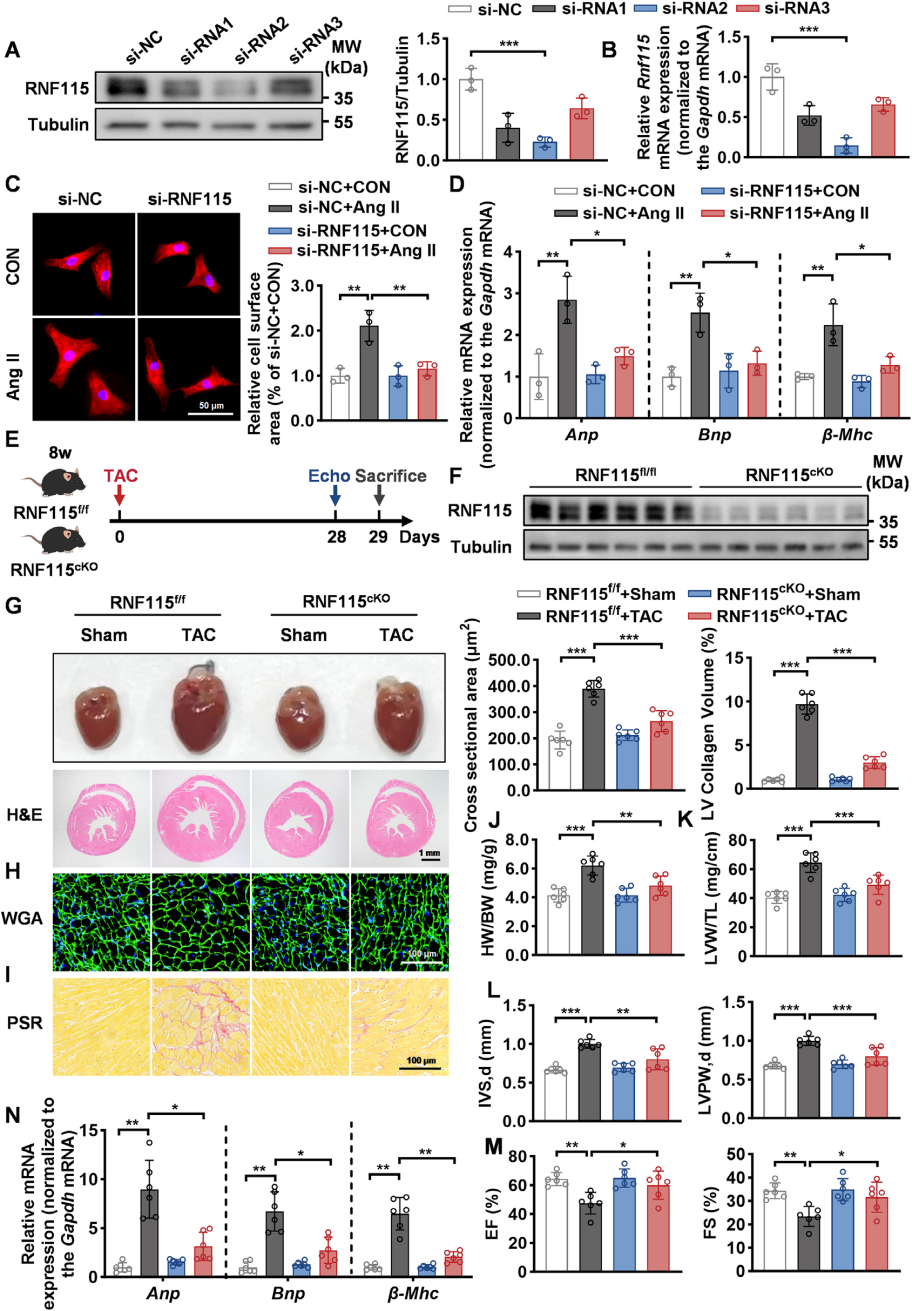
Cardiomyocyte‐specific RNF115 knockout attenuates cardiac hypertrophy. (A) Immunoblotting analysis and quantitation of RNF115 protein levels in NRCMs transfected with negative control (NC) or RNF115 siRNAs for 48 h (*n* = 3). (B) The mRNA levels of *Rnf115* in NRCMs transfected with NC or RNF115 siRNAs for 48 h were determined by qPCR (*n* = 3). (C) Immunofluorescence images and quantitation of relative cell surface area of NRCMs stained against anti‐α‐actinin (red) and DAPI (blue). The NRCMs were transfected with NC or RNF115 siRNAs and treated with Ang II or vehicle for 48 h (*n* = 3). Scale bar = 50 µm. (D) The mRNA levels of hypertrophy‐related markers *Anp*, *Bnp*, and *β‐Mhc* in NRCMs were determined by qPCR (*n* = 3). (E) Schematic diagram depicting the experimental strategy. Eight‐week‐old male RNF115^f/f^ and RNF115^cKO^ mice were subjected to sham or TAC surgery. The echocardiographic measurements were performed four weeks after surgery. (F) Immunoblotting analysis of RNF115 protein levels in heart tissue from RNF115^f/f^ and RNF115^cKO^ mice (*n* = 6). (G) Representative images of heart from each group. Histological sections of heart tissues were stained with hematoxylin and eosin (H&E) (*n* = 6). Scale bar = 1 mm. (H,I) Histological sections of heart tissues were stained with wheat germ agglutinin (WGA) and picrosirius red (PSR), and the cardiomyocyte cross‐sectional area and LV collagen volume were quantified (*n* = 6). Scale bar = 100 µm. (J) The ratio of heart weight to body weight (HW/BW) were examined in mice (*n* = 6). (K) The ratio of left ventricular weight to tibia length (LVW/TL) were examined in mice (*n* = 6). (L,M) Interventricular septum (IVS) thickness, left ventricular posterior wall (LVPW) thickness, ejection fraction (EF) and fractional shortening (FS) were examined in mice (*n* = 6). (N) The mRNA levels of *Anp*, *Bnp*, and *β‐Mhc* in heart tissues of mice were determined by qPCR (*n* = 6). Data shown as mean ± SD, *p < 0.05, **p < 0.01, ***p < 0.001. Statistical analysis assessed by one‐way ANOVA with Tukey's post‐hoc (A–M), and Brown‐Forsythe and Welch's ANOVA with Tamhane's T2 post‐hoc (N).

To investigate the role of RNF115 in cardiomyocytes during cardiac hypertrophy, we first silenced RNF115 by small interfering RNA (siRNA) and validated its efficient knockdown in NRCMs (Figure 2A,B). Ang II‐induced increases in cardiomyocyte surface area were significantly suppressed following RNF115 knockdown (Figure 2C), and mRNA levels of hypertrophy‐related markers atrial natriuretic peptide (*Anp*), brain natriuretic peptide (*Bnp*), and β‐myosin heavy chain (*β‐Mhc*) were also reduced (Figure 2D). These results indicated that downregulation of RNF115 could improve cardiomyocyte hypertrophy in vitro. Next, based on our previous finding that cardiomyocyte‐derived RNF115 is a key regulator in cardiac hypertrophy, we generated cardiomyocyte‐specific RNF115 knockout mice (RNF115^cKO^) by crossing RNF115^flox/flox^ (RNF115^f/f^) mice with α‐MHC Cre mice, and subsequently verified efficient RNF115 deletion in the heart (Figure 2F). At 8 weeks of age, both RNF115^cKO^ and control (RNF115^f/f^) mice underwent TAC or sham surgery, followed by echocardiography and tissue collection 4 weeks later (Figure 2E). Hematoxylin‐eosin (H&E) staining revealed that RNF115^cKO^ mice exhibited markedly attenuated TAC‐induced myocardial structural remodeling (Figure 2G), wheat germ agglutinin (WGA) staining demonstrated reduced cardiomyocyte cross‐sectional area (Figure 2H), and picrosirius red (PSR) staining indicated less collagen deposition compared to controls (Figure 2I). Consistently, both heart weight to body weight (HW/BW) and left ventricular weight to tibia length (LVW/TL) ratios were significantly lower in RNF115^cKO^ mice under TAC surgery (Figure 2J,K). Echocardiographic analysis further confirmed that RNF115^cKO^ mice effectively improved pathological cardiac hypertrophy after TAC surgery and exhibited superior cardiac function, as evidenced by reduced diastolic interventricular septal thickness (IVS, d) and left ventricular posterior wall thickness (LVPW, d) (Figure 2L), along with markedly improved ejection fraction (EF) and fractional shortening (FS) (Figure 2M). Finally, quantitative polymerase chain reaction (qPCR) analysis of heart tissues corroborated these improvements, showing suppressed *Anp*, *Bnp*, and *β‐Mhc* mRNA levels in RNF115^cKO^ mice after TAC surgery (Figure 2N). Together, these findings demonstrated that cardiomyocyte‐specific deficiency of RNF115 alleviates cardiac hypertrophy and dysfunction.

### 2.3 Spectrin β, Non‐Erythrocytic 1 (SPTBN1) is the Downstream Target of RNF115 in Cardiac Hypertrophy

**FIGURE 3:**
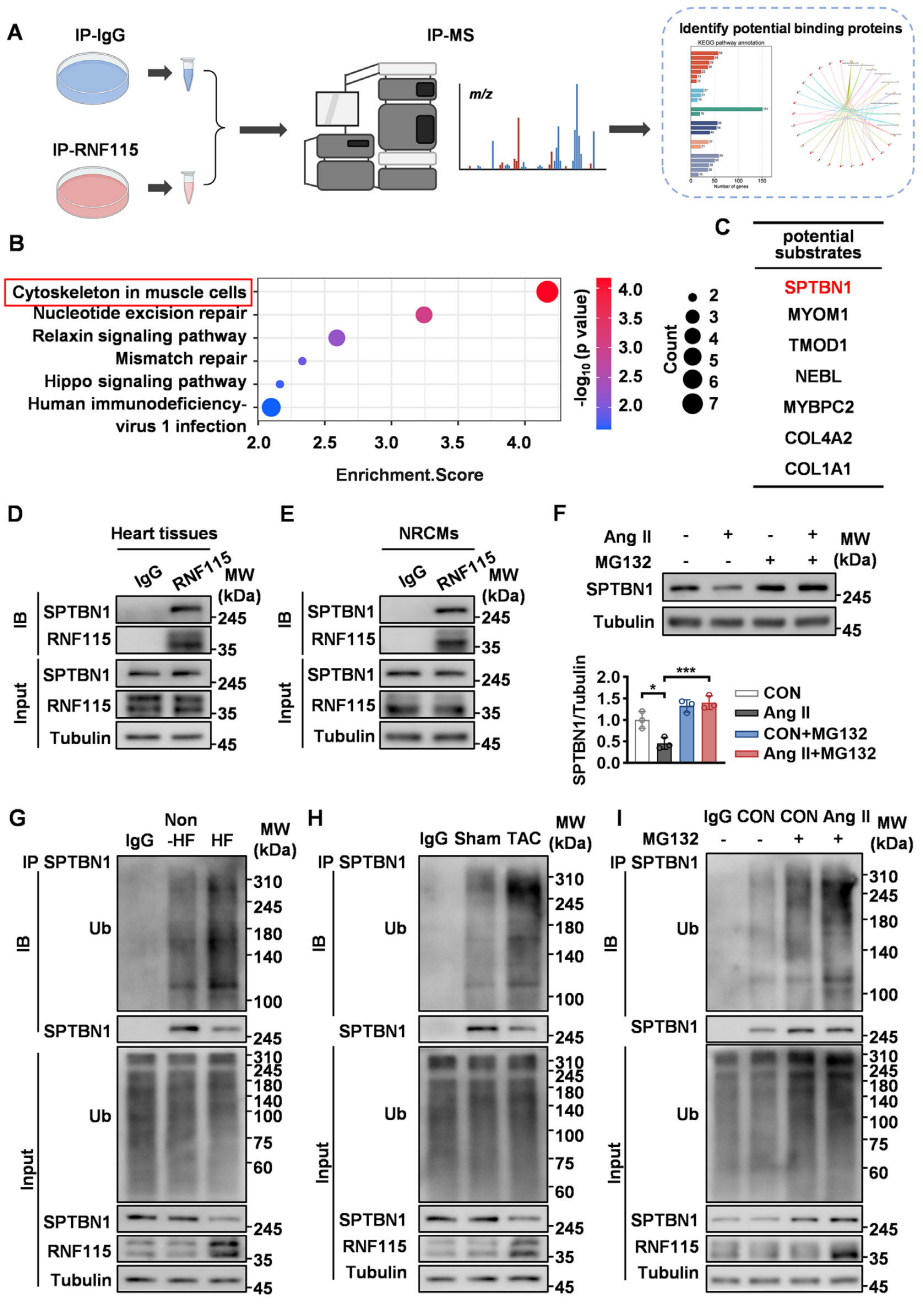
SPTBN1 was the downstream target of RNF115 in cardiac hypertrophy. (A) Schematic illustration of screening for RNF115 target interacting proteins. (B) Kyoto Encyclopedia of Genes and Genomes (KEGG) pathway analysis of proteins identified by IP‐MS. (C) The table displayed the candidate substrates of RNF115 associated with cytoskeletal signaling pathways, screened by interactomes. (D) Immunoprecipitation analysis of the interaction of RNF115 and SPTBN1 in heart tissues of mice (*n* = 3). (E) Immunoprecipitation analysis of the interaction of RNF115 and SPTBN1 in NRCMs (*n* = 3). (F) Immunoblotting analysis and quantitation of SPTBN1 protein levels in NRCMs treated with Ang II or vehicle for 48 h. The NRCMs were treated with or without MG132 (proteasome inhibitor) for 6 h before being harvested (*n* = 3). (G) Ubiquitination level of SPTBN1 was detected in heart samples of Non‐HF and HF patients (*n* = 3). (H) Ubiquitination level of SPTBN1 was detected in heart tissues from C57BL/6 mice under sham or TAC surgery (n = 3). (I) Ubiquitination level of SPTBN1 was detected in NRCMs treated with Ang II or vehicle for 48 h. The NRCMs were treated with or without MG132 for 6 h before being harvested (*n* = 3). Data shown as mean ± SD, *p < 0.05, ***p < 0.001. Statistical analysis assessed by one‐way ANOVA with Tukey's post‐hoc (F).

Given the essential role of RNF115 in cardiac hypertrophy, we proceeded to investigate its molecular mechanisms. We utilized immunoprecipitation‐mass spectrometry (IP‐MS) to identify proteins interacting with RNF115, followed by Kyoto Encyclopedia of Genes and Genomes pathway (KEGG) analysis (Figure 3A,B). Enrichment analysis revealed that proteins interacting with RNF115 were highly enriched in muscle cell cytoskeleton‐related pathways, among which SPTBN1 scored the highest (Figure 3C). The mass spectrometry results were further confirmed by immunoprecipitation, which revealed that RNF115 interacts with SPTBN1 in mouse heart tissues and NRCMs (Figure 3D,E). Therefore, we focused on SPTBN1 as a potential downstream substrate of RNF115.

Considering that RNF115 functions as an E3 ubiquitin ligase, we first investigated whether SPTBN1 is regulated by ubiquitination. In both TAC‐operated mouse model and Ang II‐induced cardiomyocyte hypertrophy model, *Sptbn1* mRNA levels remained unchanged while hypertrophy markers were upregulated (Figure S3A,B, Supporting Information). Immunoblotting assay also revealed that treatment with MG132 (proteasome inhibitor) reversed the Ang II‐induced decrease of SPTBN1 expression in NRCMs (Figure 3F). We further examined the ubiquitination levels of SPTBN1 in different hypertrophic myocardial samples. Compared to the control group, elevated ubiquitination levels of SPTBN1 were detected in heart tissue of heart failure patients and TAC‐operated mice (Figure 3G,H). A similar increase was also observed in Ang II‐induced hypertrophic groups (Figure 3I). These findings indicated that SPTBN1 is degraded via ubiquitination in cardiac hypertrophy.

### 2.4 RNF115 Promotes Degradation of SPTBN1 via K48‐Linked Ubiquitination

**FIGURE 4:**
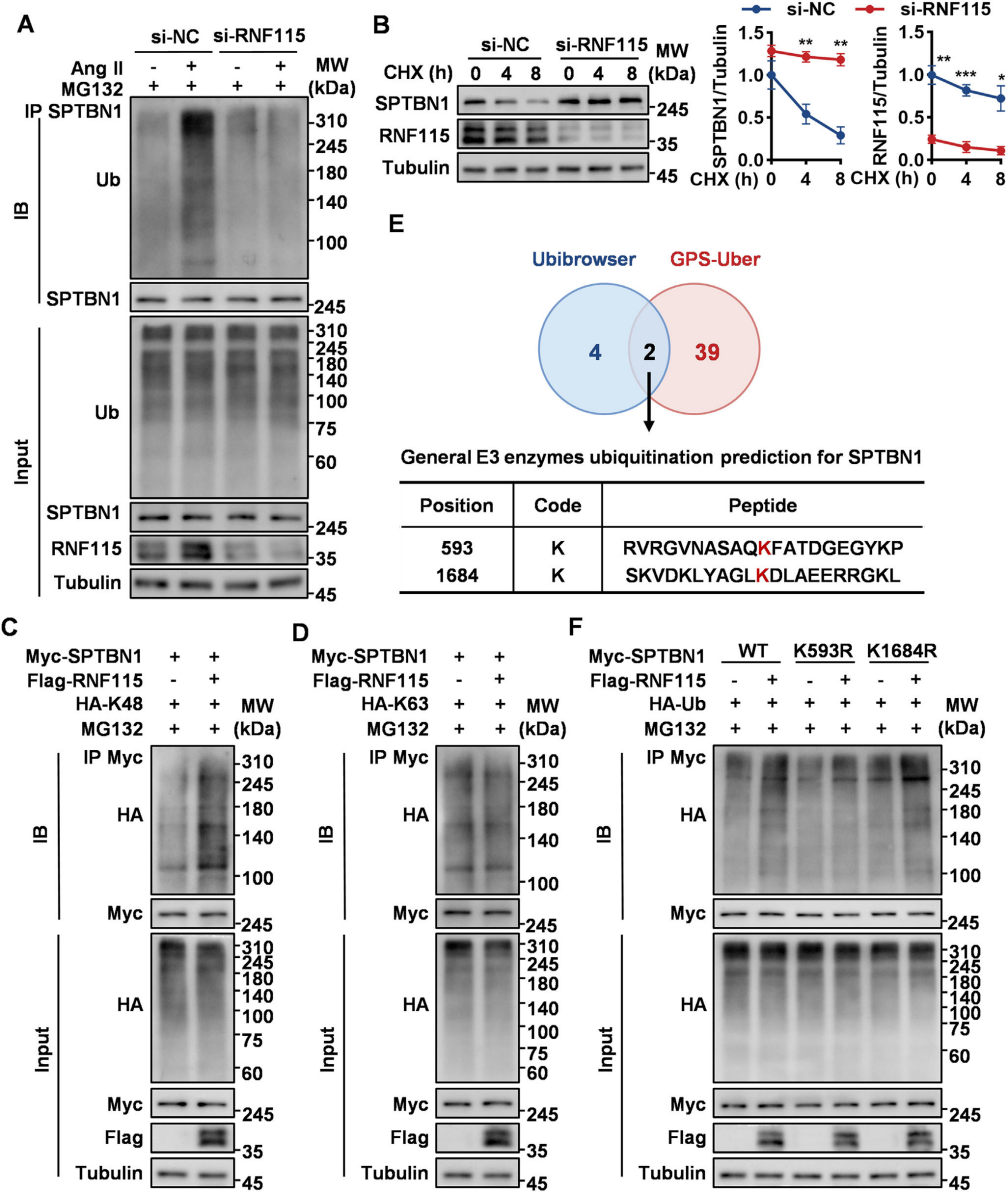
RNF115 promotes degradation of SPTBN1 via K48‐linked ubiquitination. (A) Ubiquitination level of SPTBN1 was detected in NRCMs. The NRCMs were transfected with NC or RNF115 siRNAs and treated with Ang II or vehicle for 48 h (*n* = 3). (B) Immunoblotting analysis and quantitation of RNF115 and SPTBN1 protein levels in NRCMs transfected with NC or RNF115 siRNAs and treated with cycloheximide (CHX, a protein synthesis inhibitor) for up to 8 h (*n* = 3). (C,D) Ubiquitination level of SPTBN1 was detected in HEK‐293T cells transfected with the corresponding plasmids for 48 h. Cells were treated with MG132 for 6 h before being harvested (*n* = 3). (E) Ubiquitination sites on SPTBN1 were predicted via ubibrowser database and GPS‐Uber database. (F) Ubiquitination level of SPTBN1 was detected in HEK‐293T cells transfected with the corresponding plasmids for 48 h. Cells were treated with MG132 for 6 h before being harvested (*n* = 3). Data shown as mean ± SD, *p < 0.05, **p < 0.01, ***p < 0.001. Statistical analysis assessed by two‐way ANOVA with Bonferroni post‐hoc (B).

To investigate whether the ubiquitination degradation of SPTBN1 is mediated by RNF115, we knocked down RNF115 expression in cardiomyocytes, and found that the ubiquitination level of SPTBN1 was significantly decreased (Figure 4A). We then performed a cycloheximide (CHX, a protein synthesis inhibitor) chase assay and found that RNF115 knockdown significantly extended the half‐life of SPTBN1 in NRCMs (Figure 4B). Next, to further clarify the ubiquitination modification mechanism by RNF115, we introduced hemagglutinin (HA)‐tagged ubiquitin mutants that retain the ability to form lysine (K) 48 or K63 linkages. Ubiquitination experiments demonstrated that RNF115 specifically enhanced K48‐linked polyubiquitin chain formation on SPTBN1 rather than K63‐linked chains (Figure 4C,D). Furthermore, ubiquitination sites mediated by RNF115 on SPTBN1 were predicted by ubibrowser database (ubibrowser.ncpsb.org.cn) and GPS‐Uber database (http://gpsuber.biocuckoo.cn/) [23, 24]. Lysine residues K593 and K1684 were identified as potential ubiquitination sites on SPTBN1 (Figure 4E). We mutated these K residues to arginine (R) and transfected these mutant constructs or wild‐type SPTBN1 into HEK‐293T cells. Immunoblotting assay revealed that the SPTBN1 K593R mutation significantly reduced ubiquitination levels (Figure 4F). The above results indicated that RNF115 mediates SPTBN1 degradation by promoting K48‐linked polyubiquitination at the K593 site.

To investigate the role of SPTBN1 ubiquitination at lysine 593 in cardiomyocytes, we constructed adenoviruses carrying empty vector, wild type (WT) SPTBN1, SPTBN1 K593R mutant, and RNF115, and infected NRCMs. Our results showed that the ubiquitination level of SPTBN1 was increased under Ang II stimulation in, and further elevated by RNF115 overexpression. In contrast, SPTBN1 K593R mutation prevented the elevation of ubiquitination level (Figure S4A, Supporting Information). Moreover, we found that RNF115 overexpression promoted Ang II‐induced hypertrophic genes expression and enlargement of cardiomyocyte surface area. This effect was improved by wild type SPTBN1 overexpression, and SPTBN1 K593R mutation further reduced the hypertrophic growth of cardiomyocytes (Figure S4B,C, Supporting Information). These results indicate that K593 is the key ubiquitination site of SPTBN1 in cardiomyocytes and the K593R mutation may suppress RNF115‐mediated SPTBN1 degradation and hypertrophy.

### 2.5 RNF115 Deficiency Stabilizes SPTBN1 to Attenuate Ang II‐Induced Cardiomyocyte Hypertrophy Via Reducing Filamentous Actin Depolymerization and YAP Activation

**FIGURE 5:**
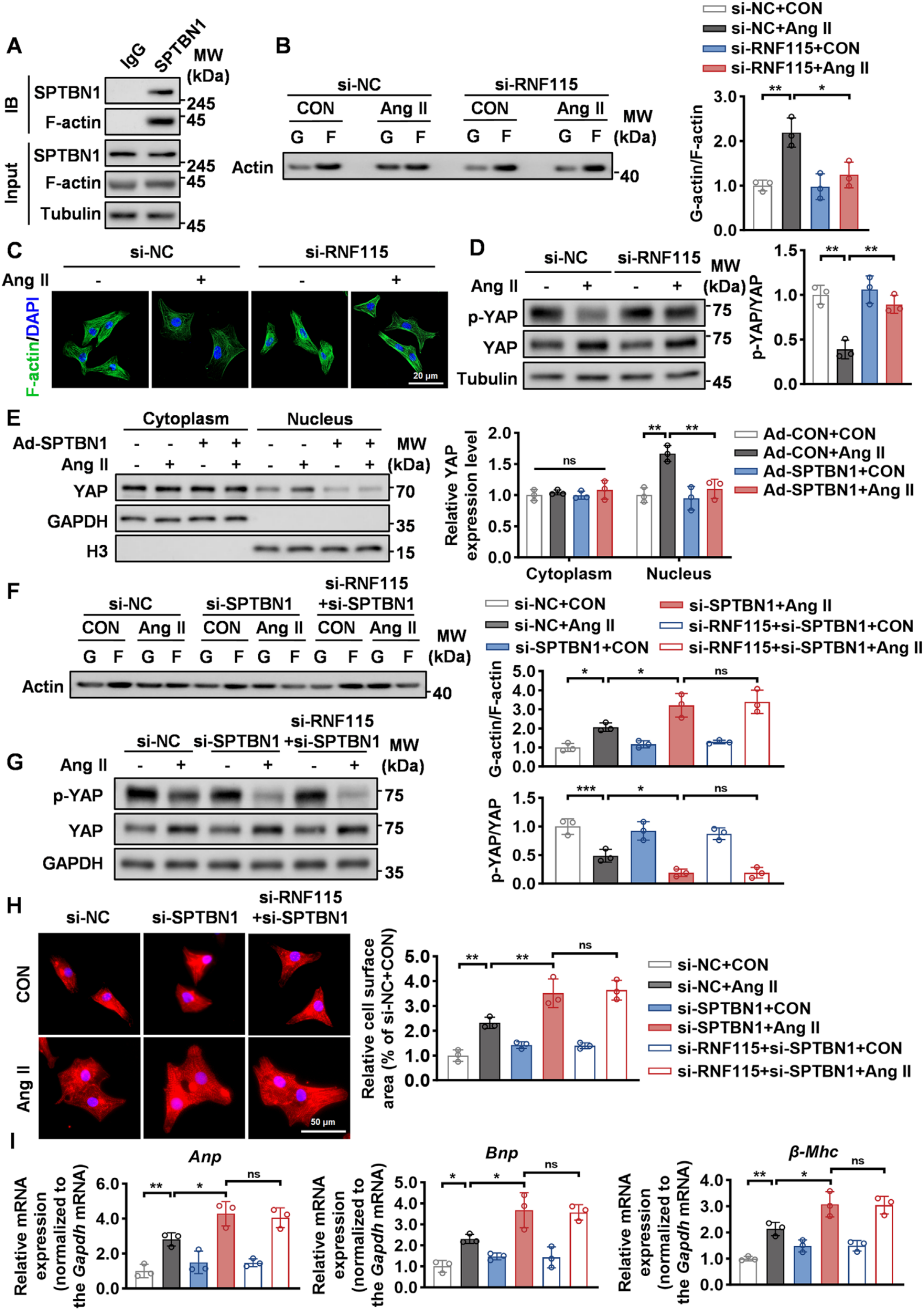
RNF115 deficiency stabilizes SPTBN1 to attenuate Ang II‐induced cardiomyocyte hypertrophy via reducing filamentous actin depolymerization and YAP activation. (A) Immunoprecipitation analysis of the interaction of SPTBN1 and F‐actin in NRCMs (*n* = 3). (B) Immunoblotting analysis and quantitation of G‐actin and F‐actin protein levels in NRCMs transfected with NC or RNF115 siRNAs and treated with Ang II or vehicle for 48 h (*n* = 3). (C) Immunofluorescence images of F‐actin (green) in NRCMs transfected with NC or RNF115 siRNAs and treated with Ang II or vehicle for 48 h (*n* = 3). Scale bar = 20 µm. (D) Immunoblotting analysis and quantitation of p‐YAP and YAP protein levels in NRCMs transfected with NC or RNF115 siRNAs and treated with Ang II or vehicle for 48 h (*n* = 3). (E) Immunoblotting analysis and quantitation of cytoplasm and nucleus expression of YAP in NRCMs. The NRCMs were infected with adenoviruses carrying empty vector or SPTBN1, and treated with Ang II or vehicle for 48 h (*n* = 3). (F) Immunoblotting analysis and quantitation of G‐actin and F‐actin protein levels in NRCMs. The NRCMs were transfected with NC, SPTBN1, or both SPTBN1 and RNF115 siRNAs, and treated with Ang II or vehicle for 48 h (*n* = 3). (G) Immunoblotting analysis and quantitation of p‐YAP and YAP protein levels in NRCMs. The NRCMs were transfected with NC, SPTBN1, or both SPTBN1 and RNF115 siRNAs, and treated with Ang II or vehicle for 48 h (*n* = 3). (H) Immunofluorescence images and quantitation of relative cell surface area of NRCMs stained against anti‐α‐actinin (red) and DAPI (blue). The NRCMs were transfected with NC, SPTBN1, or both SPTBN1 and RNF115 siRNAs, and treated with Ang II or vehicle for 48 h (*n* = 3). Scale bar = 50 µm. (I) The mRNA levels of *Anp*, *Bnp*, and *β‐Mhc* in NRCMs were determined by qPCR. The NRCMs were transfected with NC, SPTBN1, or both SPTBN1 and RNF115 siRNAs, and treated with Ang II or vehicle for 48 h (*n* = 3). Data shown as mean ± SD, *p < 0.05, **p < 0.01, ***p < 0.001, ns: not significant. Statistical analysis assessed by one‐way ANOVA with Tukey's post‐hoc (B, D–I).

SPTBN1 is a cytoskeletal protein that directly links to filamentous actin (F‐actin) to maintain cellular structural integrity, which has been reported to be critical for sustaining normal cardiac contraction function [25, 26]. Therefore, we first confirmed the interaction between SPTBN1 and F‐actin in NRCMs by immunoprecipitation (Figure 5A). F‐actin is formed through the polymerization of globular actin monomers (G‐actin) in an aligned arrangement, and maintaining the polymerization homeostasis of F‐actin plays a crucial role in cardiac hypertrophy [27, 28]. We next investigate the effect of RNF115 on F‐actin homeostasis. Interestingly, under sustained Ang II stimulation, F‐actin depolymerization increased, which was reversed upon knockdown of RNF115, as measured by decreased ratio of G‐actin to F‐actin (Figure 5B). Phalloidin staining also revealed an improvement in cytoskeletal disorganization following RNF115 knockdown (Figure 5C). Moreover, increasing evidence indicates that YAP activity is regulated by actin cytoskeleton [29, 30]. As a key downstream effector of the Hippo signaling pathway, hyperactivation of YAP in the heart drive cardiac hypertrophy and even heart failure [31]. We found that Ang II stimulation upregulates YAP expression while reducing its phosphorylation levels, indicating sustained YAP activation but suppressed following RNF115 knockdown (Figure 5D). Given that RNF115 knockdown increases SPTBN1 protein levels, we next overexpressed SPTBN1 by adenovirus to investigate its effect on Hippo/YAP activation. Immunoblotting analysis revealed that SPTBN1 overexpression could inhibit YAP nuclear translocation (Figure 5E) and downregulated the expression levels of TEA domain (TEAD) target genes *β‐Mhc* and *α‐Sma* under Ang II stimulation (Figure S5A, Supporting Information). The Hippo signaling pathway comprises a complex enzymatic cascade. Previous studies suggesting that LATS kinase functions as a critical signaling factor linking the actin cytoskeleton to Hippo signaling, whereas MST are not the key regulator in this specific signaling axis [32, 33]. Our results also showed that Ang II stimulation reduced phosphorylated large tumor suppressor homolog 1 (p‐LATS1) levels, which was attenuated by SPTBN1 overexpression. In contrast, phosphorylated mammalian sterile‐20‐like kinase 1 (p‐MST1) levels remained largely unchanged (Figure S5B, Supporting Information). These results indicate that RNF115 deficiency and SPTBN1 overexpression similarly suppress Ang II‐induced YAP activation. To further determine whether SPTBN1 acts downstream of RNF115 in this regulatory axis, we simultaneously knocked down RNF115 and SPTBN1 expression in NRCMs. We found that SPTBN1 knockdown further enhanced Ang II‐induced F‐actin depolymerization and YAP activation, and these effects were not improved by RNF115 and SPTBN1 double knockdown (Figure 5F,G; Figure S5C, Supporting Information). Immunofluorescence staining assay revealed that SPTBN1 knockdown exacerbated cardiomyocyte hypertrophy, which was not alleviated by additional knockdown of RNF115 (Figure 5H). The mRNA levels of hypertrophy markers *Anp*, *Bnp*, and *β‐Mhc* showed a consistent trend (Figure 5I). We further co‑overexpressed RNF115 and SPTBN1 using adenoviruses. The results showed that RNF115 overexpression further enhanced YAP activation compared with Ang II stimulation alone. Notably, wild type SPTBN1 overexpression suppressed YAP activation, whereas overexpression of SPTBN1 K593R mutant exerted a more significant inhibitory effect (Figure S5D, Supporting Information). These findings suggest that SPTBN1 functions downstream of RNF115. Finally, to further determine whether the regulatory effect of RNF115 on YAP is mediated through F‑actin polymerization, we treated NRCMs with Latrunculin A (LAT‑A), an inhibitor of F‑actin polymerization [34]. The results showed that Ang II promoted YAP activation and hypertrophic gene expression, which was further exacerbated by LAT‐A treatment. In contrast, RNF115 knockdown could improve Ang II‐induced YAP activation and hypertrophic gene expression, but fail to improve the aggravated phenotype caused by LAT‐A (Figure S5E,F, Supporting Information). Taken together, our findings demonstrate that RNF115 promotes SPTBN1 degradation, leading to increased F‑actin depolymerization and YAP activation, thereby aggravating cardiac hypertrophy.

### 2.6 RNF115 is Upregulated by Jun Proto‐Oncogene (c‐JUN) During Cardiac Hypertrophy

**FIGURE 6:**
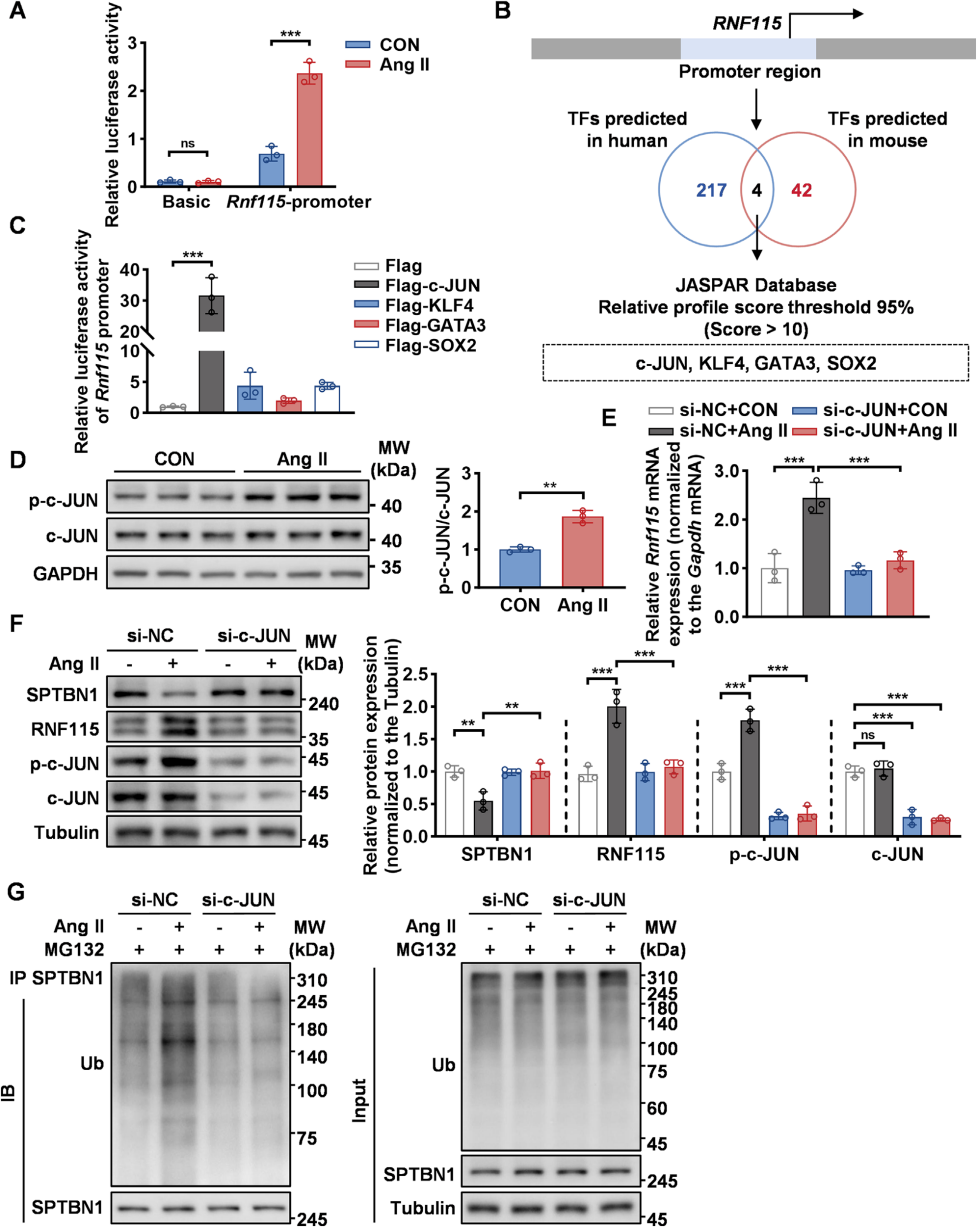
RNF115 is upregulated by Jun proto‐oncogene (c‐JUN) during cardiac hypertrophy. (A) Relative luciferase activity of *Rnf115* promoter in NMCMs transfected with pGL3‐Basic or pGL3‐*Rnf115*‐promoter and treated with Ang II or vehicle for 48 h (*n* = 3). (B) Venn diagram shows the overlap of transcription factors (TFs) of *Rnf115* promoter in the human and mouse datasets. The potential TFs of the *Rnf115* promoter were analyzed by JASPAR database. (C) Relative luciferase activity of *Rnf115* promoter transfected with Flag, Flag‐c‐JUN, Flag‐KLF4, Flag‐GATA3, or Flag‐SOX2 plasmids for 24 h in HEK‐293T cells (*n* = 3). (D) Immunoblotting analysis and quantitation of p‐c‐JUN and c‐JUN in NRCMs treated with Ang II or vehicle for 48 h (*n* = 3). (E) The mRNA levels of *Rnf115* in NRCMs were determined by qPCR. The NRCMs were transfected with NC or c‐JUN siRNAs and treated with Ang II or vehicle for 48 h (*n* = 3). (F) Immunoblotting analysis and quantitation of SPTBN1, RNF115, p‐c‐JUN, and c‐JUN in NRCMs. The NRCMs were transfected with NC or c‐JUN siRNAs and treated with Ang II or vehicle for 48 h (*n* = 3). (G) Ubiquitination level of SPTBN1 was detected in NRCMs. The NRCMs were transfected with NC or c‐JUN siRNAs and treated with Ang II or vehicle for 48 h. Cells were treated with MG132 (proteasome inhibitor) for 6 h before being harvested (*n* = 3). Data shown as mean ± SD, **p < 0.01, ***p < 0.001, ns: not significant. Statistical analysis assessed by unpaired *t*‐test (A and D), and one‐way ANOVA with Tukey's post‐hoc (C, E, and F).

Next, we explored the mechanism underlying the upregulation of RNF115 in cardiac hypertrophy. Our study has shown that both the protein and mRNA levels of RNF115 were significantly elevated in myocardial hypertrophy models (Figure 1E,H,J). We further performed luciferase assay and found *Rnf115* promoter was activated in Ang II treated NMCMs (Figure 6A). These findings indicated that RNF115 was transcriptionally upregulated in cardiac hypertrophy. To identify the upstream regulatory factors of RNF115, we used the JASPAR database (https://jaspar.elixir.no) to predict candidate transcription factors (TFs) associated with *Rnf115* promoter. Intersection of the predictions derived from human and mouse datasets revealed four TFs: c‐JUN (Jun proto‐oncogene), KLF4 (Krüppel‐like Factor 4), GATA3 (GATA binding protein 3) and SOX2 (SRY‐box transcription factor 2) (Figure 6B). We found that c‐JUN had obvious transcriptional activity toward the *Rnf115* promoter, whereas KLF4, GATA3 and SOX2 showed negligible influence (Figure 6C). Previous studies have shown that mechanical stress could be conducted by the protein kinase cascade of phosphorylation. In mechanical stress induced cardiac hypertrophy, Ang II activates the MAPK/JNK signaling pathway via Angiotensin II Type 1 Receptor (AT1R), and c‐Jun NH2‐terminal kinase (JNK) acts as the main kinase to enhance c‐Jun transcriptional activity through phosphorylation [35, 36, 37, 38]. We also found the level of phosphorylated c‐JUN (p‐c‐JUN) was significantly elevated in Ang II‐stimulated NRCMs (Figure 6D). Knockdown of c‐JUN inhibited both the mRNA and protein expression of RNF115 (Figure 6E,F). Consistently, Ang II‐induced ubiquitination and degradation of SPTBN1 was suppressed after c‐JUN knockdown (Figure 6G). Our results demonstrated that c‐JUN, one of the mechanical stress effectors, undergoes increased phosphorylation, which upregulates RNF115 expression, leading to SPTBN1 ubiquitination and degradation during cardiac hypertrophy.

### 2.7 DTD (Dithiocarbamate Disulfide Derivatives), a Specific Inhibitor of RNF115, Alleviates Ang II‐Induced Cardiomyocytes Hypertrophy

**FIGURE 7:**
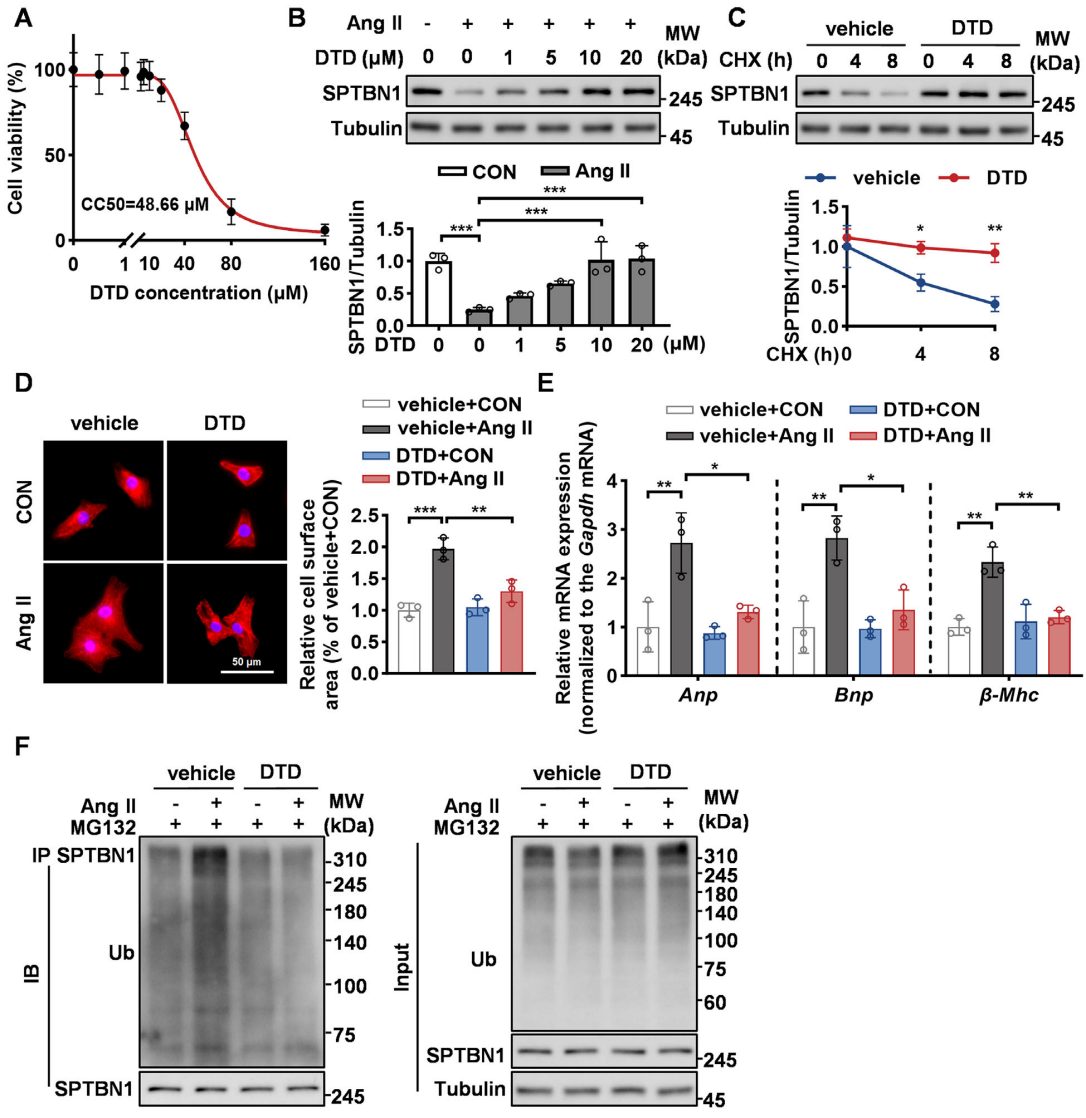
DTD (dithiocarbamate disulfide derivatives), a specific inhibitor of RNF115, alleviates Ang II‐induced cardiomyocytes hypertrophy. (A) Cytotoxicity of DTD was detected in NRCMs via cell counting kit‐8 (CCK‐8). (B) Immunoblotting analysis and quantitation of SPTBN1 protein levels in NRCMs treated with varying concentrations of DTD and stimulated with or without Ang II (*n* = 3). (C) Immunoblotting analysis and quantitation of RNF115 and SPTBN1 protein levels in NRCMs treated with DTD or vehicle and treated with CHX for up to 8 h (*n* = 3). (D) Immunofluorescence images and quantitation of relative cell surface area of NRCMs stained against anti‐α‐actinin (red) and DAPI (blue). The NRCMs were treated with DTD or vehicle for 24 h and stimulated with or without Ang II for 48 h (*n* = 3). Scale bar = 50 µm. (E) The mRNA levels of *Anp*, *Bnp*, and *β‐Mhc* in NRCMs were determined by qPCR (*n* = 3). (F) Ubiquitination level of SPTBN1 was detected in NRCMs treated with DTD or vehicle for 24 h and stimulated with or without Ang II for 48 h. Cells were treated with MG132 for 6 h before being harvested (*n* = 3). Data shown as mean ± SD, *p < 0.05, **p < 0.01, ***p < 0.001. Statistical analysis assessed by two‐way ANOVA with Bonferroni post‐hoc (C), and one‐way ANOVA with Tukey's post‐hoc (B, D and E).

Compound 5d/DTD, a previously reported inhibitor that effectively suppresses the ubiquitination activity of RNF115 [19], has an unclear role in cardiac hypertrophy. First, cytotoxicity assay demonstrated that DTD exhibited low cytotoxicity in NRCMs (Figure 7A). We then examined SPTBN1 expression levels at different safe concentrations, and found that 10 µM DTD effectively counteracted Ang II‐induced SPTBN1 reduction and prolonged its half‐life in NRCMs (Figure 7B,C). Next, we investigated the effect of DTD on cardiac hypertrophy in vitro. We found that DTD successfully attenuated the increase in cardiomyocyte surface area induced by Ang II (Figure 7D) and suppressed the elevation of mRNA levels for *Anp*, *Bnp*, and *β‐Mhc* (Figure 7E). Notably, Ang II‐induced degradation of SPTBN1 via the ubiquitin pathway was also suppressed following DTD treatment (Figure 7F).

### 2.8 DTD Improves Post‐TAC Cardiac Function and Reduction in Cardiac Hypertrophy

Next, we evaluated the protective effects of DTD against cardiac hypertrophy in vivo. We subjected 8‐week‐old C57BL/6 mice to either TAC or sham surgery, with intraperitoneal administration of varying doses of DTD (0, 10, 20, 40 mg·kg^−1^·d^−1^) starting 1 week prior to surgery and continuing for 5 weeks, then echocardiography analysis was performed at the end of the treatment period (Figure S6A, Supporting Information). The results showed that doses of 20 and 40 mg·kg^−1^·d^−1^ conferred significant cardioprotective effect without showing signs of hepatic or renal toxicity (Figure S6B; Table S3, Supporting Information). Therefore, we proceeded with subsequent experiment using DTD at a dosage of 20 mg·kg^−1^·d^−1^ (Figure S7A, Supporting Information). H&E staining of harvested hearts revealed that TAC‐induced cardiac enlargement was markedly attenuated by DTD treatment (Figure S7B, Supporting Information), and that similar improvements were also observed in cardiomyocyte cross‐sectional area and collagen deposition (Figure S7C,D, Supporting Information). Moreover, DTD effectively suppressed the TAC‐induced increases in HW/BW and LVW/TL ratios (Figure S7E,F, Supporting Information). The echocardiography analysis also showed decreased IVS and LVPW, along with increased EF and FS (Figure S7G,H, Supporting Information), accompanied by downregulation of *Anp*, *Bnp*, and *β‐Mhc* at the mRNA level (Figure S7I, Supporting Information). We further assessed ubiquitination levels in cardiac tissue following TAC surgery with or without DTD administration. Consistent with prior findings, TAC‐induced increase in ubiquitination of SPTBN1 was significantly suppressed by DTD, thereby maintaining SPTBN1 protein levels (Figure S7J, Supporting Information).

**FIGURE 8:**
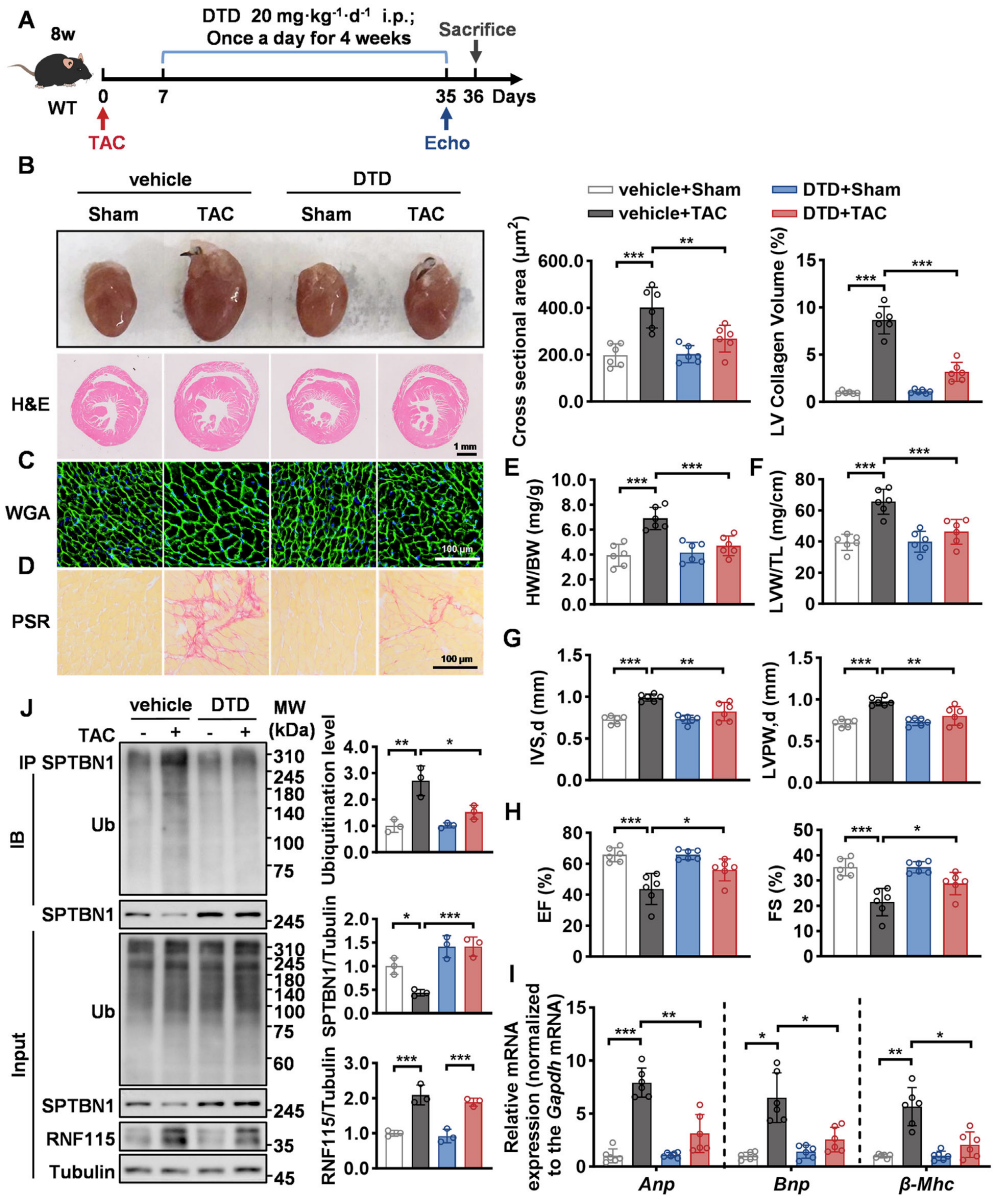
DTD (dithiocarbamate disulfide derivatives) improves post‐TAC cardiac function and reduction in cardiac hypertrophy. (A) Schematic diagram depicting the experimental strategy. Eight‐week‐old male C57BL/6 mice were subjected to sham or TAC surgery. One week after surgery, mice were treated with DTD (20 mg·kg^−1^·d^−1^) or vehicle control via intraperitoneal administration once a day for four weeks. The echocardiographic measurements were performed four weeks after DTD treatment. (B) Representative images of heart from each group. Histological sections of heart tissues were stained with H&E (*n* = 6). Scale bar = 1 mm. (C,D) Histological sections of heart tissues were stained with WGA and PSR, and the cardiomyocyte cross‐sectional area and LV collagen volume were quantified (*n* = 6). Scale bar = 100 µm. (E) The ratio of HW/BW were examined in mice (*n* = 6). (F) The ratio of LVW/TL were examined in mice (*n* = 6). (G,H) IVS, LVPW, EF and FS were examined in mice (*n* = 6). (I) The mRNA levels of *Anp, Bnp*, and *β‐Mhc* in mice heart tissues were determined by qPCR (*n* = 6). (J) Ubiquitination level of SPTBN1 and its quantitation were detected in heart tissues from mice (*n* = 3). Data shown as mean ± SD, *p < 0.05, **p < 0.01, ***p < 0.001. Statistical analysis assessed by one‐way ANOVA with Tukey's post‐hoc (C–H and J), and Brown‐Forsythe and Welch's ANOVA with Tamhane's T2 post‐hoc (I).

To further confirm the potential therapeutic benefit of DTD, 8‐week‐old C57BL/6 mice were subjected to sham or TAC surgery. 1 week after surgery, mice were treated with DTD (20 mg·kg^−1^·d^−1^) for 4 weeks (Figure 8A). It also showed similar improvement to cardiac hypertrophy as DTD prophylactic administration (Figure 8).

In summary, we demonstrated that under pathogenic stimuli, increased c‑JUN phosphorylation upregulates RNF115, leading to SPTBN1 ubiquitination and degradation, which promotes F‑actin depolymerization and YAP activation, driving cardiac hypertrophy. The RNF115 inhibitor DTD effectively suppresses SPTBN1 ubiquitination and attenuates cardiac hypertrophy.

## 3 Discussion

This study provides the first evidence that E3 ubiquitin ligase RNF115 is a key pro‐hypertrophic factor, while inhibiting RNF115 effectively ameliorates cardiac hypertrophy. Mechanistically, pathological stimuli increase c‐JUN phosphorylation, which upregulates RNF115 expression. RNF115 interacts with SPTBN1 in cardiomyocytes, promoting K48‐linked polyubiquitination at the K593 site, resulting in increased SPTBN1 degradation. Notably, SPTBN1 is closely associated with F‐actin polymerization dynamics. Increased degradation of SPTBN1 reduces F‐actin stability, leading to sustained YAP activation and promoting myocardial hypertrophy. Importantly, our study demonstrates for the first time that the RNF115 inhibitor DTD suppresses SPTBN1 degradation mediated by ubiquitination and effectively alleviates cardiac hypertrophy. These findings expand the molecular mechanisms and provide an effective intervention strategy for ameliorating cardiac hypertrophy and dysfunction.

Given the essential role of RNF115 as an E3 ubiquitin ligase, we employed immunoprecipitation‐mass spectrometry to screen for its directly interacting substrate proteins. Mass spectrometry analysis strongly indicated its regulatory function in the cytoskeleton, among which we identified SPTBN1 as the highest‐scoring factor. The spectrin family was first identified in erythrocytes and plays a crucial role in maintaining cellular structure. SPTBN1, the most common non‐erythrocyte spectrin isoform, is expressed in the heart, where it contributes to maintaining normal cardiac function and ameliorating TAC‐induced heart failure [26, 39]. Recent studies have confirmed that SPTBN1 can stabilize the cytoskeleton through cross‐linking with F‐actin and plays an important role in protecting mitochondrial respiratory function following ischemia‐reperfusion injury [25]. Our research demonstrated a direct interaction between RNF115 and SPTBN1. Upregulation of RNF115 in cardiac hypertrophy leads to increased K48‐linked polyubiquitination and degradation of SPTBN1 at the K593 site, resulting in excessive depolymerization of F‐actin and ultimately impaired cardiac contractile function. Increasing evidence indicates that YAP activity is regulated by cytoskeletal dynamics [30, 40, 41]. Interestingly, our enrichment pathway analysis also revealed that the Hippo signaling pathway is affected. F‐actin depolymerization‐induced imbalance in the Hippo‐YAP pathway exerts dual effects in cardiac diseases [31]. On one hand, as a key downstream effector of the Hippo signaling pathway, moderate activation of YAP is beneficial for cardiomyocyte survival [42, 43, 44]. On the other hand, in pressure overload‐induced cardiac hypertrophy, suppression of the Hippo pathway leads to sustained YAP activation, which in turn promotes the transcription of hypertrophy‐related genes [45, 46], thereby exacerbating heart failure and dysfunction. These studies suggest YAP plays distinct roles under different cardiac homeostasis, and the specific molecular mechanisms by which the RNF115‐SPTBN1‐F‐actin axis regulates Hippo‐YAP pathway balance to mediate cardioprotection effects require further investigation.

The immunoprecipitation‐mass spectrometry (IP‐MS) analysis of RNF115‐interacting proteins identified several relatively high‐confidence candidates, including Karyopherin Subunit Beta 1 (KPNB1), WD Repeat Domain 7 (WDR7), Importin 9 (IPO9), SHANK‐Associated RH Domain Interactor (SHARPIN), RNA Polymerase II Subunit H (POLR2H), SPTBN1, etc. Among them, KPNB1 and IPO9 are nucleocytoplasmic transport proteins [47]; WDR7 is involved in multiprotein complex assembly [49]; SHARPIN is a component of the linear ubiquitin assembly complex (LUBAC) essential for immune responses [50]; and POLR2H is a member of the POLR family that participates in nucleotide excision repair [48]. Moreover, KPNB1 and IPO9 exhibited gene expression alterations in published cardiac hypertrophy transcriptomic datasets, suggesting that they may be regulated at the transcriptional level. Whether these candidate genes play a functional role in cardiac hypertrophy has not yet been reported, and the contribution of these potential ubiquitination targets to RNF115‐mediated cardiac hypertrophy remains unclear. We have not explored them in this study, which warrants further investigation.

Disulfiram (DSF) is an FDA‐approved drug for alcohol dependence. Recent studies have reported that RNF115 promotes STING‐mediated inflammation, and pharmacological inhibition of RNF115 by DSF may serve as a potential therapeutic strategy for autoimmune diseases [20]. However, DSF inhibits both aldehyde dehydrogenase (ALDH) and RNF115, so its specificity is not fully selective. Moreover, several DSF‐derived compounds have been reported to exhibit inhibitory activity against RNF115 ubiquitin ligase, among which DTD shows specific inhibition of RNF115 without affecting ALDH. Furthermore, these DSF derivatives, including DTD, exhibit anti‐tumor effects [19]. These studies suggest therapeutic potential for inhibiting RNF115 in multiple diseases. However, whether RNF115 inhibition has a protective effect in the cardiovascular system has not been explored. Our study provides new evidence supporting the translational potential of targeting RNF115 in cardiac hypertrophy. Notably, the dosages employed in tumor and autoimmune diseases are relatively high and treatment durations are also long, leading to corresponding toxicity and side effects. In our study, DTD administration was not associated with hepatorenal toxicity or cardiac injury, further highlighting its translational potential. The balance between drug dosage and long‐term toxicity warrants further investigation.

SPTBN1 is indeed an important regulator of calcium homeostasis that modulates ryanodine receptor 2 (RYR2)‐mediated calcium release channels, and SPTBN1 deficiency has been shown to cause aberrant calcium release and arrhythmia [26, 51]. We also examined the expression of calcium handling‐related genes and found no significant differences after DTD treatment. Although calcium homeostasis is closely associated with cardiac hypertrophy, our results suggest that the protective effects of DTD in cardiac hypertrophy may not be directly related to changes in calcium homeostasis. In conclusion, this study identifies RNF115 as a novel pathological regulator of cardiac hypertrophy and elucidates the crucial regulatory role of the RNF115‐SPTBN1 axis in this process. By linking ubiquitination signaling to cytoskeletal dynamics, this work provides a new molecular mechanism underlying how ubiquitination regulates the pathological progression of cardiac hypertrophy. Furthermore, the RNF115 inhibitor DTD is demonstrated to exert significant cardioprotective effects, offering a potential drug target with translational value for the treatment of cardiac hypertrophy and heart failure.

## 4 Experimental Section

### 4.1 Human Heart Samples

This study was conducted in accordance with the Declaration of Helsinki. Human heart tissue analysis was approved by the ethics committee of Nanjing First Hospital Affiliated to Nanjing Medical University (Approval No. KY20190404‐03‐KS‐01). As previously described [52], all patients provided written informed consent. Heart failure tissue samples were collected from the left ventricle (LV) of dilated cardiomyopathy patients undergoing heart transplantation. Non‐heart failing tissue samples were obtained from non‐failing donors (whose next‐of‐kin had consented to the use of hearts declined for transplantation for research purposes) (Table S1, Supporting Information).

### 4.2 Animal Experiments

All animal experiments were approved by the Animal Care and Use Committee of Nanjing Medical University and conducted in accordance with the National Institutes of Health Guide for the Care and Use of Laboratory Animals (Approval No. IACUC‐1811063). Male C57BL/6 mice were purchased from the Experimental Animal Center of Nanjing Medical University. Compound 5d/DTD (S‐hexyl N,N‐diethyldithiocarbamate disulfide) has been reported as a disulfiram derivative that effectively inhibits RNF115 activity [19]. At 7 weeks of age, male C57BL/6 mice received intraperitoneal administration of DTD (10, 20, 40 mg·kg^−1^) or vehicle control (DMSO) once a day for 5 weeks, followed by sham or TAC surgery 1 week after administration. 4 weeks later, echocardiography was performed and cardiac tissues were harvested for subsequent studies. We measured heart weight (HW), left ventricular weight (LVW), body weight (BW), and tibial length (TL) in the mice. Cardiomyocyte‐specific RNF115 knockout mice (RNF115^cKO^) were generated by crossing the α‐MHC Cre mice with the RNF115^flox/flox^ mice (RNF115^f/f^). The RNF115^f/f^ mice in the C57BL/6 background were obtained from Nanjing Medical University. These mice were all in a specific pathogen‐free (SPF) environment with a 12 h light/dark cycle. For toxicity test, blood biochemical examination was used to measure alanine aminotransferase (ALT), aspartate aminotransferase (AST), total protein (TP), albumin (ALB), total bilirubin (TBIL), uric acid (UA), blood urea nitrogen (BUN) and creatinine (CREA). The test was performed on a biochemistry automatic analyzer (7100, HITACHI, Tokyo, Japan) in Experimental Animal Center of Nanjing Medical University.

### 4.3 Echocardiography

The mice were anesthetized by inhalation of 2% isoflurane, placed on a heated pad to maintain body temperature at 37°C, underwent complete depilation of the thoracic region, and had ultrasound coupling gel applied to the chest wall. Using a high‐frequency linear array transducer on a small animal ultrasound system (Visual Sonic Vevo 2100 and 3100), a parasternal short‐axis view at the level of the left ventricular myocardium was obtained. M‐mode echocardiography tracings were recorded to measure interventricular septal thickness at diastole (IVS, d), and left ventricular posterior wall thickness at diastole (LVPW, d), while left ventricular ejection fraction (EF) and fractional shortening (FS) were calculated using Vevo Analysis software (version 2.2.3).

### 4.4 Transverse Aortic Constriction Model

8‐week‐old male C57BL/6 mice were anesthetized with 2% isoflurane and positioned supine on a heated pad to maintain body temperature. The chest area was depilated, and the skin was disinfected with 75% ethanol. Subsequently, the trachea was exposed via a midline neck incision, a breathing tube was inserted through the mouth, and it was connected to a circulating rodent ventilator. Using blunt dissection, the transverse aorta was dissected between brachiocephalic trunk and left common carotid artery. The aorta was ligated with a 27G needle and 7‐0 suture. Finally, closed the muscle layer and skin incision. For the sham surgery group, the same exposure procedure was performed, but sutured the incision without ligation. Mice were sacrificed after 4 weeks.

### 4.5 Cell Culture and Treatment

When the cell density reached 60%–70%, the culture medium was replaced to remove dead cells, followed by treatment with DTD (10 µM) or vehicle control (DMSO) for 24 h, angiotensin II (Ang II, 100 nM, A9525, Sigma‐Aldrich, USA) or vehicle control (PBS) for 48 h, Latrunculin A (LAT‐A, 250 nM, HY‐16929, MedChem Express, USA) or vehicle control (DMSO) for 1 h, and MG132 (50 µM, HY‐13259, MCE, USA) for 6 h before cell collection, while cycloheximide (CHX, 50 µM, HY‐12320, MCE, USA) was administered for the indicated periods. For endogenous RNF115 and SPTBN1 knockdown experiments, NRCMs were transfected with small interfering RNA (GenePharma, Shanghai, China) using a transfection reagent according to the manufacturer's instructions. For RNF115 and SPTBN1 overexpression experiments, NRCMs were infected with adenoviruses carrying empty vector (Ad‐CON), wild‐type SPTBN1 (Ad‐Myc‐SPTBN1‐WT), SPTBN1 K593R mutant (Ad‐Myc‐SPTBN1‐MUT), or RNF115 (Ad‐Flag‐RNF115). The adenoviruses were generated by Genechem (Shanghai, China). HEK‐293T cells were purchased from the Cell Bank of Shanghai Academy of Sciences and cultured in Dulbecco's Modified Eagle's Medium (DMEM, 11995065, Invitrogen, USA) containing 10% fetal bovine serum (FBS, F8318, Sigma‐Aldrich, USA) and 1% Penicillin‐Streptomycin (P/S, C0222, Beyotime, China) solution at 37°C incubator with 5% CO_2_. The expression plasmid was constructed by GeneChem (Shanghai, China). HEK‐293T cells were placed in 6‐well plates and transfected with plasmids at 50%–60% confluence, using Lipofectamine 3000 reagent (L3000015, Thermo Fisher, USA) following the manufacturer's instructions.

### 4.6 Protein Extraction and Immunoblotting

Heart tissues were perfused and immediately flash‐frozen in liquid nitrogen, while cells were subjected to different treatments before harvest. Both cardiac tissues and cells were washed twice with cold phosphate‐buffered saline (PBS), followed by lysis in RIPA buffer (P0013B, Beyotime, China) supplemented with a protease inhibitor cocktail (78438, Thermo Fisher, USA) to prevent protein degradation. The cell lysates were incubated on ice for 20 min, and centrifuged at 12 000 × *g* for 15 min at 4°C as the total protein sample. Protein concentration was quantified using the BCA protein assay kit (23225, Thermo Fisher, USA). Equal amounts of protein were mixed with loading buffer, denatured by boiling at 99°C for 5 min, separated by 8% SDS‐PAGE, and then transferred onto polyvinylidene difluoride (PVDF) membranes (ISEQ00010, Millipore, USA). The membranes were blocked with 5% milk for 2 h at room temperature, followed by incubation with primary antibodies against target proteins overnight at 4°C. The next day, membranes were incubated with secondary antibodies for 2 h at room temperature, and detected the band by ECL detection reagent (1805001, Tanon, China). The band intensities were quantified using Image J software. The following primary antibodies were used in this study: anti‐RNF115 (1:1000, ab18742, Abcam, USA), anti‐Tubulin (1:5000, AB0039, Abways, China), anti‐SPTBN1 (1:800, 19722‐1‐AP, Proteintech, USA), anti‐Ub (1:1000, ab134953, Abcam, USA), anti‐Myc (1:1000, 2276, CST, USA), anti‐Flag (1:1000, 14793, CST, USA), anti‐HA (1:1000, 3724, CST, USA), anti‐Pan‐Actin (1:1000, 8456, CST, USA), anti‐F‐actin (1:1000, ab130935, Abcam, USA), anti‐YAP1 (p‐S127) (1:1000, ab76252, Abcam, USA), anti‐YAP1 (1:1000, 13584‐1‐AP, Proteintech, USA), anti‐GAPDH (1:50000, 60004‐1‐Ig, Proteintech, USA), anti‐MST1 (1:1000, 22245‐1‐AP, Proteintech, USA), anti‐p‐MST1 (1:2000, 80093‐1‐RR, Proteintech, USA), anti‐LATS1 (1:1000, 17049‐1‐AP, Proteintech, USA), anti‐p‐LATS1 (1:5000, 28998‐1‐AP, Proteintech, USA), anti‐c‐JUN (1:1000, 24909‐1‐AP, Proteintech, USA), anti‐p‐c‐JUN (AP0105, ABclonal, China).

### 4.7 Co‐Immunoprecipitation

Heart tissues and cells were washed with cold PBS and lysed in lysis buffer consisting of 40 mM HEPES, pH 7.4, 2 mM EDTA, 100 mM pyrophosphate (P8010, Sigma‐Aldrich, USA), 100 mM β‐glycerophosphate (G9422, Sigma‐Aldrich, USA), 0.5% Triton X‐100 (ST795, Beyotime, China), with a protease inhibitor cocktail. After being incubated at 4°C for 30 min, the cell suspension was centrifuged at 12 000 × *g* at 4°C for 10 min. Sample lysates were incubated with Protein G Sepharose beads (17061801, Cytiva, USA) and anti‐SPTBN1 or anti‐RNF115 antibody at 4°C overnight. Immunoglobulin G (IgG) was used as a negative control. The next day, the beads were washed for five times and boiled with 2× loading buffer at 99°C for 10 min. Finally, the samples were analyzed by immunoblotting.

### 4.8 Ubiquitination Assays

Cells were treated with MG132 before being harvested. Cells and heart tissues were washed with cold PBS for three times and lysed in lysis buffer (50 mM Tris‐HCl, pH 7.5, 150 mM NaCl, 1 mM EDTA, 1 mM EGTA, 1% Triton X‐100, 1 mM PMSF [ST506, Beyotime, China], 20 mM N‐ethylmaleimide [NEM, 04259, Sigma‐Aldrich, USA]), with a protease inhibitor cocktail. The cell suspension was incubated at 4°C for 30 min and centrifuged at 12 000 × *g* at 4°C for 10 min. Then, the supernatant was incubated with Protein G Sepharose beads and anti‐SPTBN1 or anti‐Myc antibody at 4°C overnight. The next day, the beads were washed for five times and boiled with 2× loading buffer at 99°C for 10 min. The level of ubiquitination was analyzed by immunoblotting with anti‐Ub or anti‐HA antibody.

### 4.9 Nuclear and Cytoplasmic Fractionation

Cytoplasmic and nuclear proteins from the indicated cells were extracted using the NE‐PER Nuclear and Cytoplasmic Extraction Reagents (78833, Thermo Fisher Scientific, USA) according to the manufacturer's instructions. The detailed procedure was as follows: Cells were washed twice with PBS, detached with trypsin, and centrifuged at 500 × *g* for 3 min. Cytoplasmic Extraction Reagent I (CER I) and Cocktail (100:1) were added, incubation on ice for 10 min. Cytoplasmic Extraction Reagent II (CER II) was then added, and the sample was incubated on ice for 1 min. After centrifugation at 16 000 × *g* for 5 min at 4°C, the supernatant was cytoplasmic fraction. The pellet was washed three times with ice‐cold PBS. Nuclear Extraction Reagent (NER) and Cocktail (100:1) were added, and the sample was sonicated, followed by incubation on ice for 40 min with vigorous vortexing for 15 s every 10 min. After centrifugation at 16 000 × *g* for 10 min at 4°C the supernatant was nuclear fraction.

### 4.10 Isolation of Neonatal Rat Cardiomyocytes

Neonatal rat cardiomyocytes (NRCMs) were respectively isolated from Sprague‐Dawley rats (1 to 3 days) purchased from the Experimental Animal Center of Nanjing Medical University. After disinfecting the newborn rats with 75% ethanol, we opened the chest cavity with sterile forceps and scissors, and removed the heart. Next, the hearts were placed in a sterile petri dish containing DMEM, carefully removed the excess blood and debris with forceps, and cut it into small pieces. We transferred the heart fragments to a sterile conical flask, added 0.25% trypsin (C0201, Beyotime, China), and digested for 5 min each time. After each digestion, we transferred the supernatant to a new tube containing an equal volume of termination solution (DMEM contain 25% FBS) to neutralize the enzymes. Repeat the digestion process seven to ten times until tissues were completely dissociated. We centrifuged cell suspension at 2200 × *g* for 8 min, and the precipitate was the extracted cells. We resuspended them in DMEM containing 10% FBS and 1% P/S. After 3 h, we collected the cell suspension, which was the cardiomyocyte, and filtered through a 100 µm cell strainer to remove undigested tissue fragments. The cardiomyocytes were enriched and transferred to another dish for incubation at 37°C with 5% CO_2_.

### 4.11 Isolation of Neonatal Mouse Cardiomyocytes

Neonatal mouse cardiomyocytes (NMCMs) were respectively isolated from C57BL/6 mice (1 to 3 days) purchased from the Experimental Animal Center of Nanjing Medical University. Prepare sterilized instruments and cold PBS in advance. Under sterile conditions, the mice were disinfected with 75% ethanol, immobilized, and thoracotomized. Hearts were rapidly excised with curved ophthalmic forceps, rinsed in cold PBS to remove blood, transferred to 50 mL tubes, and washed with Hank's buffer until clear. Hank's buffer and trypsin were added for overnight digestion at 4°C. The next day, stop the digestion process and wash several times. Type II collagenase (LS004176, Worthington, USA) solution was used for digestions. We centrifuged the digestive supernatant at 1500 × *g* for 5 min and the precipitate was the extracted cells. Then the pellet was resuspended in DMEM containing 10% FBS and 1% P/S for plating. After 2 h incubation at 37°C, cardiomyocytes were collected via differential purification and cultured at 37°C with 5% CO_2_.

### 4.12 Luciferase assay

We cloned and constructed the promoter of *Rnf115* (genome: GRCm39, chr3:96632868‐96634868) to pGL3‐Basic vector. The luciferase signal was measured using Luciferase Reporter Assay Kit (MA0518, Meilunbio, China).

### 4.13 Isolation of F‐Actin and G‐Actin

The assay was performed using a commercially available kit (BK037, Cytoskeleton, USA) according to the manufacturer's instructions. Briefly, cultured cells were washed with PBS and lysed by warm LAS2 buffer. The collected lysates underwent incubation for 10 min at 37°C. The supernatant was ultra‐centrifuged at 100 000 × *g* at 37°C for 1 h to pellet F‐actin, leaving G‐actin in the supernatant. To depolymerize the pelleted F‐actin, 100 µL of F‐actin depolymerization buffer was added, followed by incubation on ice for 1 h. The samples were mixed with loading buffer and analyzed by immunoblotting.

### 4.14 Cytotoxicity Assays

According to the manufacturer's instructions, we used the Cell Counting Kit‐8 (40203ES80, Yeasen, China) to evaluate the cytotoxicity of DTD in NRCMs. The half‐maximal cytotoxic concentration (CC_50_) was then calculated by performing nonlinear regression analysis (dose‐response inhibition) using Prism software (version 10.1.2, GraphPad, USA).

### 4.15 Immunofluorescence Assays

Cells were washed with PBS for three times and fixed with 4% paraformaldehyde for 20 min, permeabilized with 0.3% Triton X‐100 for 20 min and blocked in 3% BSA (ST023, Beyotime, China) for 30 min. Then, the cells were incubated with primary antibodies at 4°C overnight. The next day, cells were incubated with secondary antibodies at 37°C for 1 h and cell nuclei were counterstained with DAPI (0100‐20, SouthernBiotech, USA). Samples were imaged through microscope (DMi8, Leica, Germany) and cell surface area was calculated using Image J software.

Heart tissue sections were placed in a 55°C oven to fix for 1 h. After deparaffinization and rehydration, the sections were immersed in antigen repair solution, heated at 95°C for 15 min and cooled to room temperature. The sections were incubated with 0.3% Triton X‐100 for 20 min to disrupt the membrane, and blocked with 10% BSA at room temperature for 30 min. Primary antibodies were then added, followed by incubation at 4°C overnight. The next day, sections were incubated with secondary antibodies at 37°C for 1 h and cell nuclei were counterstained with DAPI. Samples were imaged through confocal microscope (LSM 900, Zeiss, Germany) and quantified using Image J software.

The following antibodies were used in this study: anti‐α‐actinin antibody (1:200, a7811, Merck, Germany), anti‐RNF115 antibody (1:100, HPA019130, Sigma‐Aldrich, USA), anti‐cTnT antibody (1:100, ab8295, Abcam, USA).

### 4.16 Phalloidin Staining

Removed the culture medium from the cells and washed twice with 37°C PBS. Cells were fixed with 4% paraformaldehyde for 10 min, then permeabilized with 0.5% Triton X‐100 for 10 min, and washed three times with PBS. Next, we added 5 µg/mL FITC‐phalloidin (40774ES08, Yeasen, China) and incubated the cells at room temperature for 30 min, followed by 3 washes with PBS. The nuclei were stained with DAPI, and samples were imaged using a confocal microscope (LSM 900, Zeiss, Germany).

### 4.17 Picrosirius Red Staining

Heart tissues were fixed with paraformaldehyde and embedded in paraffin. After deparaffinization and rehydration, 5 µm thick heart tissue sections were stained with 0.1% picrosirius red (26357‐02, HEAD Biotechnology, China) for 90 min. After staining, the sections were briefly differentiated in 10 mM hydrochloric acid (7647‐01‐0, Nanjing Reagent, China) for 10 s, followed by dehydration through alcohol and xylene. Remove residual xylene liquid around the sections and seal with sealing cement (BA7004, BASO, China). The extent of fibrosis and collagen deposition were quantified by Image J software.

### 4.18 Hematoxylin‐Eosin Staining

Heart tissues were fixed with paraformaldehyde and embedded in paraffin. After deparaffinization and rehydration, 5 µm thick heart sections were stained with hematoxylin (G1004, Servicebio, China) for 3 min and rinsed with ddH_2_O. Subsequently, the sections were blued in Scott's tap water (20 g MgSO_4_·7H_2_O and 3.5 g NaHCO_3_ per 1000 mL ddH_2_O) and rinsed again with ddH_2_O. Next, we placed the heart sections in eosin staining (BA4024, BASO, China) for 3 min, and dehydrated in alcohol and xylene. Finally, the sections were fixed with sealing cement. We observed the general morphology of the heart under a light microscope (BX63, Olympus, Japan).

### 4.19 Wheat Germ Agglutinin Staining

Heart tissues were excised and embedded in OCT compound (4583, SAKURA, USA) and cut into 5 µm sections. The sections were baked in oven at 55°C for 3 h to get fixed, permeabilized with 0.1% Triton X‐100, and incubated with FITC‐conjugated WGA (5 µg/mL, W11261, Invitrogen, USA) at 37°C for 1 h. DAPI was used to label the nuclei. Images were acquired by fluorescence microscope (BX63, Olympus, Japan). The cardiomyocyte cross‐sectional areas were measured using Image J software.

### 4.20 RNA Isolation and qPCR

Total RNA was extracted from cultured cells or heart tissues using TRIzol reagent (15596026, Thermo Fisher, USA) according manufacturer's protocol. Use 0.5–1 µg of total RNA for reverse transcription by HiScript II Q RT SuperMix (R222‐01, Vazyme, China), and gene expression was quantified by primers and SYBR Green Master Mix (Q131‐02, Vazyme, China). The experiments used *Gapdh* as an endogenous reference gene and data analysis using ΔΔCt method. All primers used are listed in Table S2, Supporting Information.

### 4.21 Statistical Analysis

All data were presented as mean ± standard deviation (SD), except for blood biochemistry, which was expressed as mean ± standard error of the mean (SEM). Data distribution was initially analyzed for normality using the Shapiro‐Wilk test. Unpaired two‐tailed Student's *t* test was used for comparisons between two groups when data passed normality and equal variance test; otherwise, Mann‐Whitney *U* test was used. One‐way or two‐way ANOVA was used followed by post hoc analysis using Tukey method to adjust for multiple comparisons in more than two groups, Brown‐Forsythe and Welch's ANOVA were used for data with unequal variances, followed by Tamhane's T2 method for post hoc analysis. In all cases, *p* ≤ 0.05 was considered statistically significant. All graphs and statistical analyses were performed using GraphPad Prism 8 (GraphPad Software, San Diego, CA, USA).

## Author Contributions

**Shichen Huang**: investigation. **Bowen Li**: investigation. **Dian Yu**: methodology. **Yong Ji**: conceptualization, funding acquisition, project administration, resources, supervision. **Jing Tian**: formal analysis. **Ji Chen**: formal analysis, software. **Shiqing Chen**: methodology, investigation. **Yi Han**: funding acquisition, supervision, writing – review and editing. **Yuhan Zhao**: writing – review and editing. **Weijian Chu**: investigation. **Liuliu Ruan**: methodology, investigation. **Yan Zu**: methodology, investigation, writing – original draft. **Xin Tang**: conceptualization, writing – review and editing, funding acquisition, methodology, data curation, validation. All authors read and approved the final manuscript.

## Conflicts of Interest

The authors declare no conflict of interest.

## Supporting information




**Supporting File**: advs76077‐sup‐0001‐SuppMat.pdf.

**Supporting File**: advs76077‐sup‐0002‐Data.zip.

## Data Availability

The data that support the findings of this study are available from the corresponding author upon reasonable request.
