# Supplementary material for: The E3 Ligase RNF115 Aggravates Pathological Cardiac Hypertrophy via Ubiquitin‐Mediated Degradation of SPTBN1
Source: Adv Sci (Weinh). 2026 Jun 15;13(47):e76077. doi: 10.1002/advs.76077 (PMC13336878; doi:10.1002/advs.76077)
Supplement: Supplementary file 1 [file ADVS-13-e76077-s001.pdf]

Supporting Information

The E3 Ligase RNF115 Aggravates Pathological Cardiac Hypertrophy via Ubiquitin-Mediated Degradation of SPTBN1

The supplementary file contents

Supplementary tables (S1-S4)
Supplementary figures (S1-S7)

1. Supporting tables

Table S1. Clinical characteristics of Non-HF and HF patients

Patient	Gender	Age	Heart Rate (BPM)	LVEF (%)	Dilated Cardiomyopathy (DCM)	Atrial Fibrillation	Congenital Heart Disease
Non-HF 1	M	57	62	65	N	N	N
Non-HF 2	M	42	69	72	N	N	N
Non-HF 3	M	17	65	68	N	N	N
Non-HF 4	M	53	75	66	N	N	N
Non-HF 5	F	49	83	59	N	N	N
Non-HF 6	M	54	76	67	N	N	N
HF 1	M	70	76	20	Y	Y	N
HF 2	F	56	61	28	Y	N	N
HF 3	M	55	76	27	Y	N	N
HF 4	M	51	88	29	Y	Y	N
HF 5	M	58	61	20	Y	N	N
HF 6	F	63	86	29	Y	Y	N
P value	> 0.999	0.070	0.612	< 0.001	0.002	0.182	> 0.999

Non-HF: non-heart failure; HF: heart failure; LVEF: left ventricular ejection fraction; Y: Yes; N: No. Two-tailed student's *t*-test was used for the statistical comparison of continuous variables and Fisher's exact test was used for the categorical variables.

Table S2. Sequences of primers used for qPCR assay

Gene	Forward	Reverse
RNF115 (human)	AGCTGACAAGGAAAAGATCACA	CTAGCCACGGCACAATACAAC
GAPDH (human)	GTCTTCACCACCATGGAGAAGG	GCCTGCTTCACCACCTTCTTGA
Rnf115 (mouse)	GTCCCAGATGTGACTCAGGCTT	GGATTGCTACTTAGAAATGGTC
Gapdh (mouse)	GGCATCTTGGGCTACACTGA	GGGATAGGGCCTCTCTTGCT
Anp (mouse)	GCTTCCAGGCCATATTGGAG	GGGGGCATGACCTCATCTT
Bnp (mouse)	GAGGTCACTCCTATCCTCTGG	GCCATTTCCTCCGACTTTTCTC
β-Mhc (mouse)	ACTGTCAACACTAAGAGGGTCA	TTGGATGATTTGATCTTCCAGGG
Sptbn1 (mouse)	CCAGACTGCTATCGCCTCAG	AGCTCATTCCAGCCAGTGTC
Rnf115 (rat)	TGCCACTTTTGTAAGGGCGA	GTGCTATTGTCTGTCCGGCT
Gapdh (rat)	GGAGCGAGATCCCTCCAAAAT	GGCTGTTGTCATACTTCTCATGG
Anp (rat)	ATCTGCCCTCTTGAAAAGCA	GGATCTTTTGCGATCTGCTC
Bnp (rat)	ATCGGCGCAGTCAGTCGCTT	GGTGGTCCCAGAGCTGGGGAA
β-Mhc (rat)	AGGTGAAGGCCTACAAGCGC	GGGTTGGGTAGCACAAGATC
Sptbn1 (rat)	CGAAGTGGCCCTTGATTACAA	GAGGTACTCGTTGCCATCACTTAG

Table S3. Prophylactically administered DTD has no side effects on liver and kidney function

	Sham		TAC	
Dose (mg/kg/d)	0	40	0	40
ALT (U/L)	40.07 ± 2.94	33.86 ± 2.43	34.49 ± 3.40	36.35 ± 2.94
AST (U/L)	114.48 ± 5.87	119.41 ± 4.67	111.88 ± 7.17	116.53 ± 9.81
TP (g/L)	50.04 ± 3.52	44.73 ± 2.73	48.02 ± 3.23	41.44 ± 4.97
ALB (g/L)	25.26 ± 3.06	27.81 ± 1.61	30.54 ± 2.38	29.43 ± 2.57
TBIL (μmol/L)	0.62 ± 0.07	0.74 ± 0.08	0.72 ± 0.11	0.79 ± 0.08
BUN (mmol/L)	13.86 ± 1.79	15.52 ± 1.03	13.14 ± 1.90	16.87 ± 1.37
CREA (μmol/L)	16.50 ± 1.41	18.29 ± 1.16	17.73 ± 2.17	19.03 ± 2.18
UA (μmol/L)	111.8 ± 8.57	120.81 ± 6.23	108.90 ± 7.47	117.79 ± 8.78

Serum was collected from mice that underwent sham or transverse aortic constriction (TAC) surgery with prophylactically administered DTD (40 $mg \cdot kg^{-1} \cdot d^{-1}$) prior to sacrifice. Blood biochemical examination was used to measure alanine aminotransferase (ALT), aspartate aminotransferase (AST), total protein (TP), albumin (ALB), total bilirubin (TBIL), blood urea nitrogen (BUN), creatinine (CREA) and uric acid (UA). Values are shown as means ± SEM (n=6 per group). Statistical analysis assessed by one-way ANOVA with Tukey's post-hoc.

Table S4. IP-MS screening of candidate RNF115 binding proteins

No.	Protein Name	Score	Intensity
1	KPNB1	38.290	284750000
2	WDR7	37.694	213250000
3	IPO9	35.499	147530000
4	SHARPIN	30.881	49420000
5	POLR2H	27.858	108850000
6	SPTBN1	26.414	96158000
7	AGFG1	24.423	96003000
8	RBM33	21.793	194500000
9	YTHDF1	20.625	179370000
10	SMARCC2	18.855	208640000
11	LUC7L3	18.822	117490000
12	CDC42BPA	18.813	237930000
13	UFD1L	17.403	282110000
14	SEC23IP	17.323	50021000
15	NAA35	16.691	123680000
16	NFX1	16.684	25341000
17	WDR37	16.600	255920000
18	PDS5A	16.526	273020000
19	PDF	15.436	346650000
20	MYOM1	15.316	97680000
21	PRPF8	15.225	78062000
22	SMC2	14.867	205980000
23	GBAS	14.686	56449000
24	ALDH2	14.456	102190000
25	POLR2I	14.344	73498000
26	CBX3	14.263	91637000
27	OPHN1	14.179	110970000
28	PRKAA1	13.888	37710000
29	TMOD1	13.881	54938000
30	NCAPG	13.029	66787000

2. Supporting figures

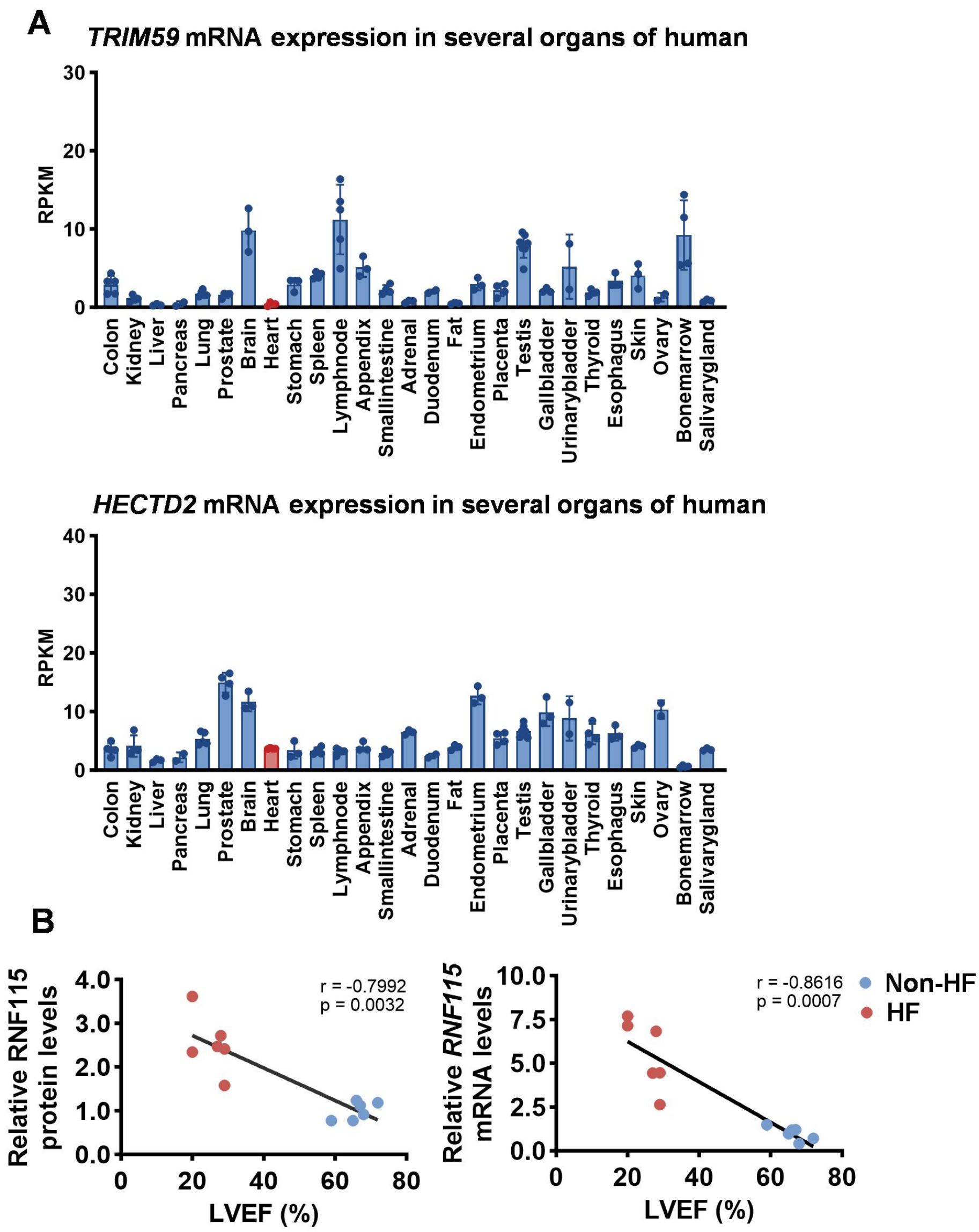

Figure S1. TRIM59 and HECTD2 exhibit low gene expression in cardiac tissue and RNF115 expression is closely related to cardiac function. A) The mRNA levels of *TRIM59* and *HECTD2* in several organs of human. Data available in Array Express under accession number E-MTAB-1733. B) The correlation between RNF115 protein/mRNA expression and LVEF in human heart tissues by using Pearson's correlation and partial correlation adjusted by age (n = 6). All statistical tests were two-sided, and $p < 0.05$ was considered statistically significant (B).

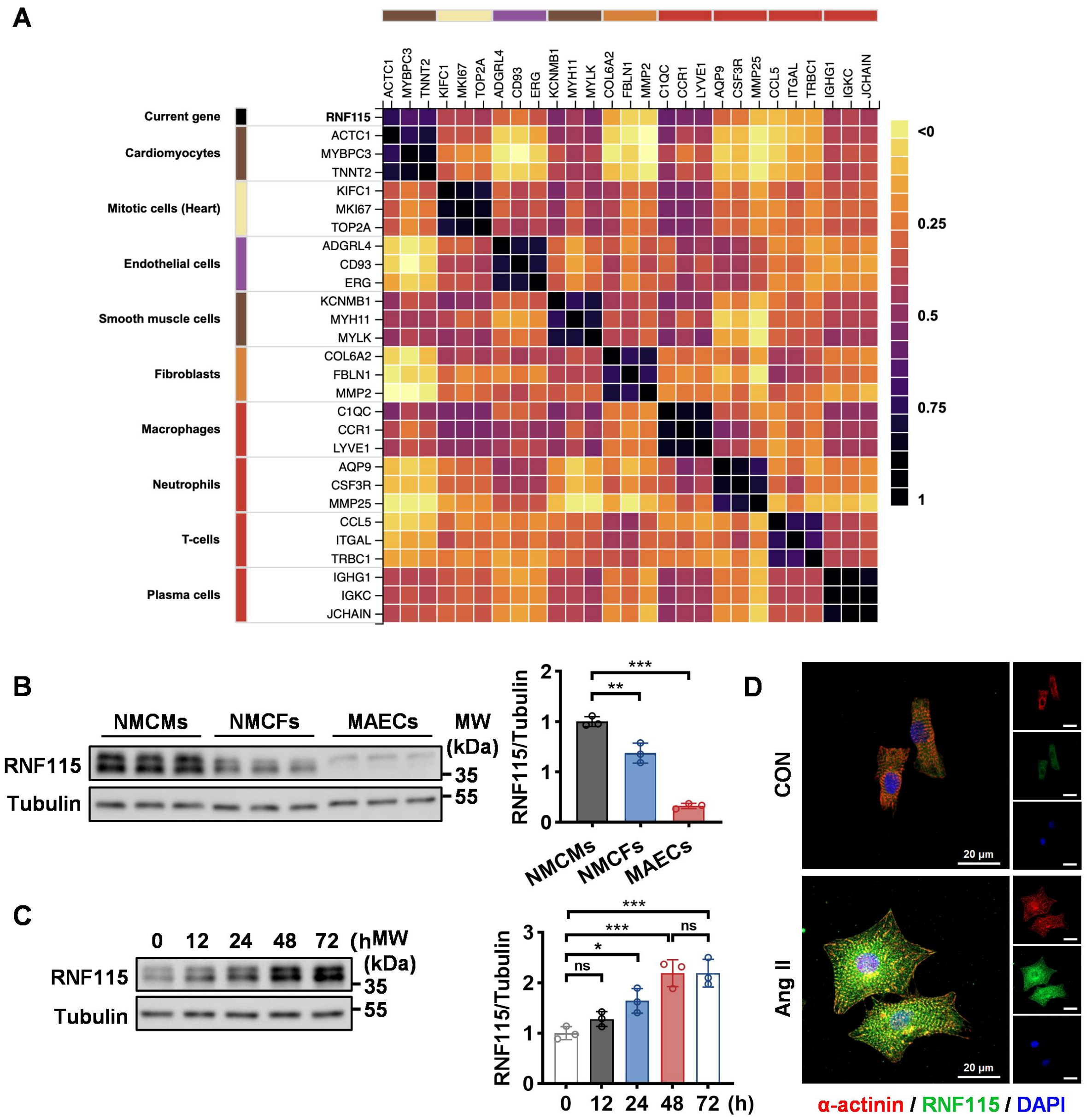

Figure S2. RNF115 is highly expressed in cardiomyocytes. A) Single-cell database analysis of the cellular sources of RNF115 in myocardial tissue. B) Immunoblotting analysis and quantitation of RNF115 protein levels in neonatal mouse cardiomyocytes (NMCMs), neonatal mouse cardiac fibroblasts (NMCFs), and mouse aortic endothelial cells (MAECs) (n = 3). C) Immunoblotting analysis and quantitation of RNF115 protein levels in angiotensin II (Ang II)-treated cardiomyocytes at indicated time points (n = 3). D) Immunofluorescence images of RNF115 (green), α-actinin (red), and DAPI (blue) in neonatal rat cardiomyocytes (NRCMs) treated with Ang II or vehicle (n = 3). Scale bar = 20 μm. Data shown as mean ± SD, *P < 0.05, **P < 0.01, ***P < 0.001, ns: not significant. Statistical analysis assessed by one-way ANOVA with Tukey's post-hoc (B and C).

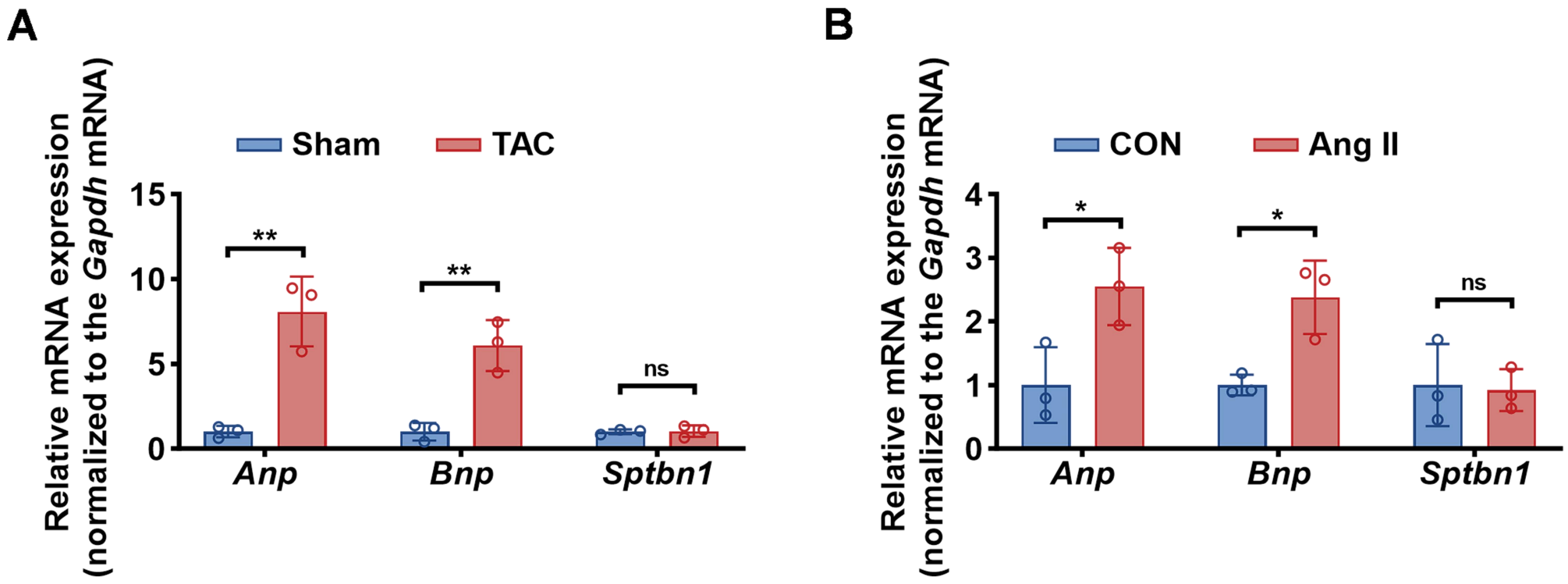

Figure S3. *Sptbn1* mRNA levels remain unchanged in hypertrophic models. A) The mRNA levels of *Anp*, *Bnp*, and *Sptbn1* in heart tissues from C57BL/6 mice under sham or TAC surgery were determined by qPCR (n = 3). B) The mRNA levels of *Anp, Bnp, and Sptbn1* in NRCMs treated with Ang II or vehicle were determined by qPCR (n = 3). Data shown as mean ± SD, *P < 0.05, **P < 0.01, ns: not significant. Statistical analysis assessed by unpaired t-test (A and B).

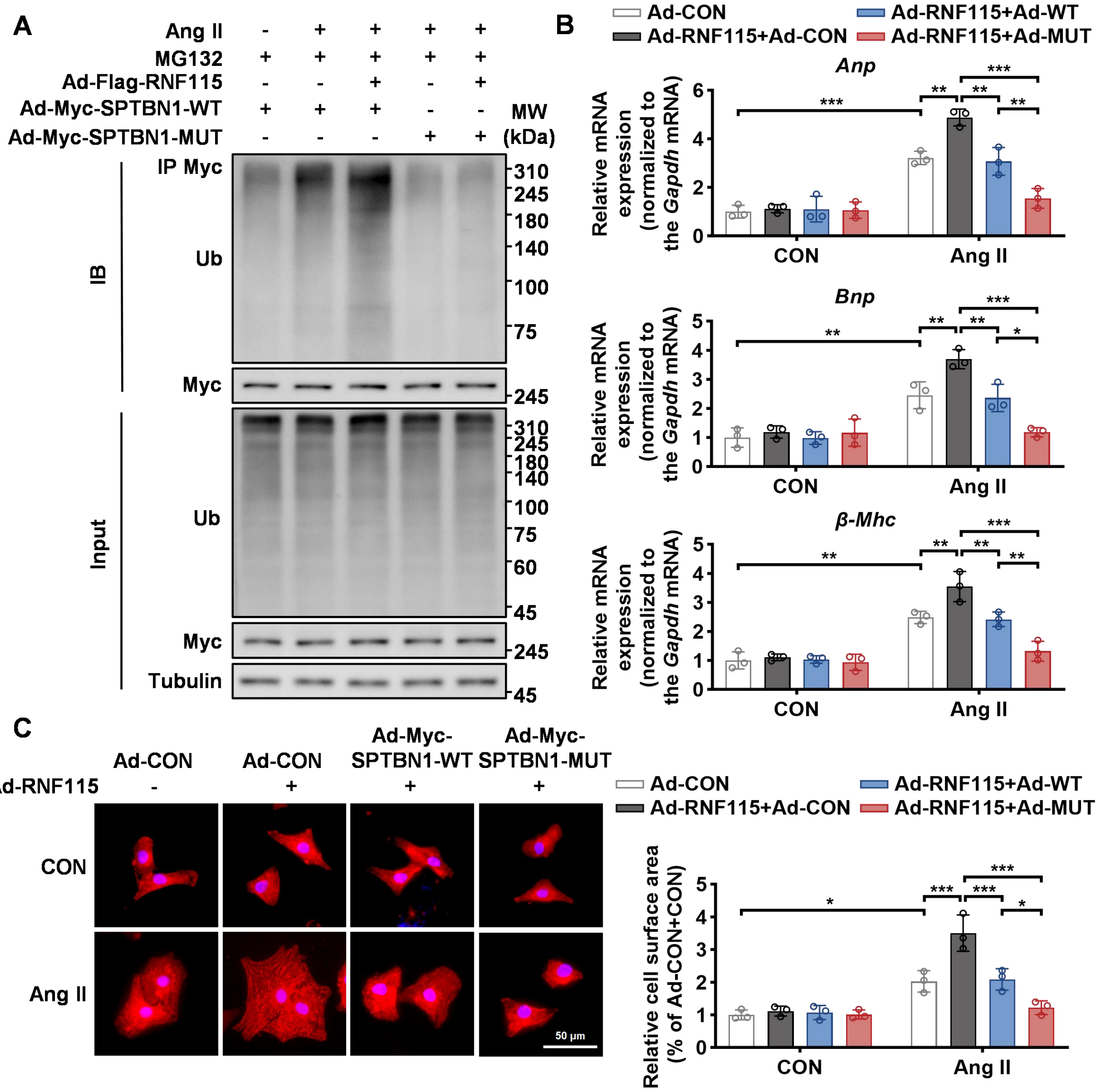

Figure S4. Inhibition of lysine 593 ubiquitination of SPTBN1 attenuates RNF115-mediated cardiomyocyte hypertrophy. A) Ubiquitination level of SPTBN1 was detected in NRCMs. The NRCMs were infected with adenoviruses carrying empty vector (Ad-CON), wild type SPTBN1 (Ad-Myc-SPTBN1-WT) or SPTBN1 K593R mutant (Ad-Myc-SPTBN1-MUT) under control and RNF115-overexpressing conditions, and treated with Ang II or vehicle for 48 h. Cells were treated with MG132 for 6 h before being harvested (n = 3). B) The mRNA levels of *Anp*, *Bnp*, and *β-Mhc* in NRCMs were determined by qPCR. The NRCMs were infected with adenoviruses carrying empty vector (Ad-CON), SPTBN1 wild type (Ad-WT) or SPTBN1 K593R mutant (Ad-MUT) under control and RNF115-overexpressing conditions, and treated with Ang II or vehicle for 48 h (n = 3). C) Immunofluorescence images and quantitation of relative cell surface area of NRCMs stained against anti-α-actinin (red) and DAPI (blue). The NRCMs were infected with adenoviruses carrying empty vector (Ad-CON), SPTBN1 wild type (Ad-WT) or SPTBN1 K593R mutant (Ad-MUT) under control and

RNF115-overexpressing conditions, and treated with Ang II or vehicle for 48 h (*n* = 3). Scale bar = 50 μm. Data shown as mean ± SD, *P < 0.05, **P < 0.01, ***P < 0.001. Statistical analysis assessed by two-way ANOVA with Tukey's post-hoc (B and C).

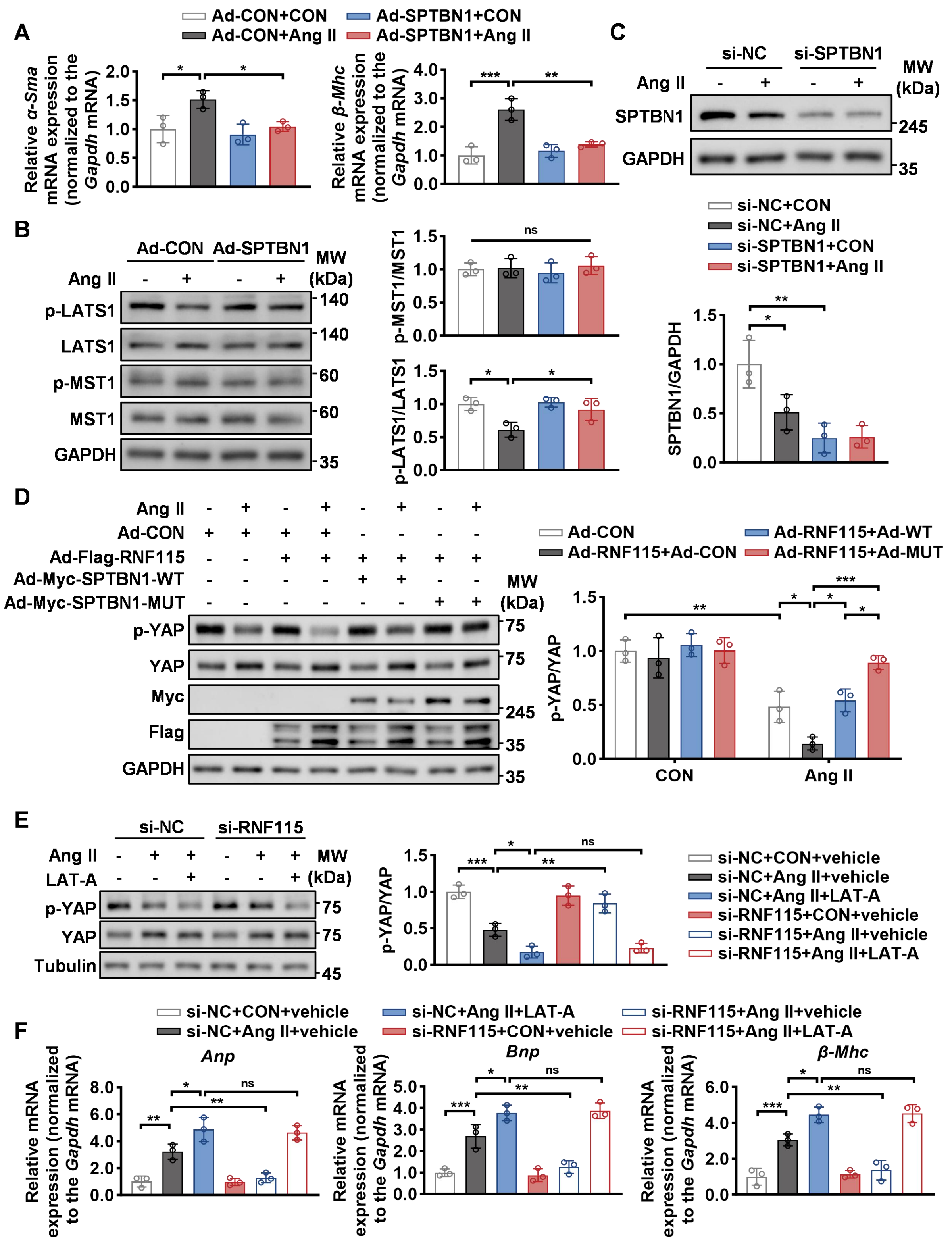

Figure S5. RNF115/SPTBN1axis regulates the Hippo-YAP signaling pathway in myocardial hypertrophy. A) The mRNA levels of *α-Sma* and *β-Mhc* in NRCMs were determined by qPCR. The NRCMs were infected with adenoviruses carrying empty vector or SPTBN1, and treated with Ang II or vehicle for 48 h (*n* = 3). B) Immunoblotting analysis and quantitation of p-LATS, LATS, p-MST1 and MST1 protein levels in NRCMs. The NRCMs were infected

with adenoviruses carrying empty vector or SPTBN1, and treated with Ang II or vehicle for 48 h (*n* = 3). C) Immunoblotting analysis and quantitation of SPTBN1 protein levels in NRCMs transfected with NC or SPTBN1 siRNAs and treated with Ang II or vehicle for 48 h (*n* = 3). D) Immunoblotting analysis and quantitation of p-YAP and YAP protein levels in NRCMs. The NRCMs were infected with adenoviruses carrying empty vector (Ad-CON), wild type SPTBN1 (Ad-WT) or SPTBN1 K593R mutant (Ad-MUT) under control and RNF115-overexpressing conditions, and treated with Ang II or vehicle for 48 h (*n* = 3). E) Immunoblotting analysis and quantitation of p-YAP and YAP protein levels in NRCMs transfected with NC or RNF115 siRNAs, treated with Latrunculin A (LAT-A) or vehicle for 1 h, and stimulated with Ang II or vehicle for 48 h (*n* = 3). F) The mRNA levels of *Anp*, *Bnp*, and *β-Mhc* in NRCMs were determined by qPCR. The NRCMs were transfected with NC or RNF115 siRNAs, treated with LAT-A or vehicle for 1 h, and stimulated with Ang II or vehicle for 48 h (*n* = 3). Data shown as mean ± SD, *$P < 0.05$, **$P < 0.01$, ***$P < 0.001$, ns: not significant. Statistical analysis assessed by one-way ANOVA with Tukey's post-hoc (A–C, E and F), and two-way ANOVA with Tukey's post-hoc (D).

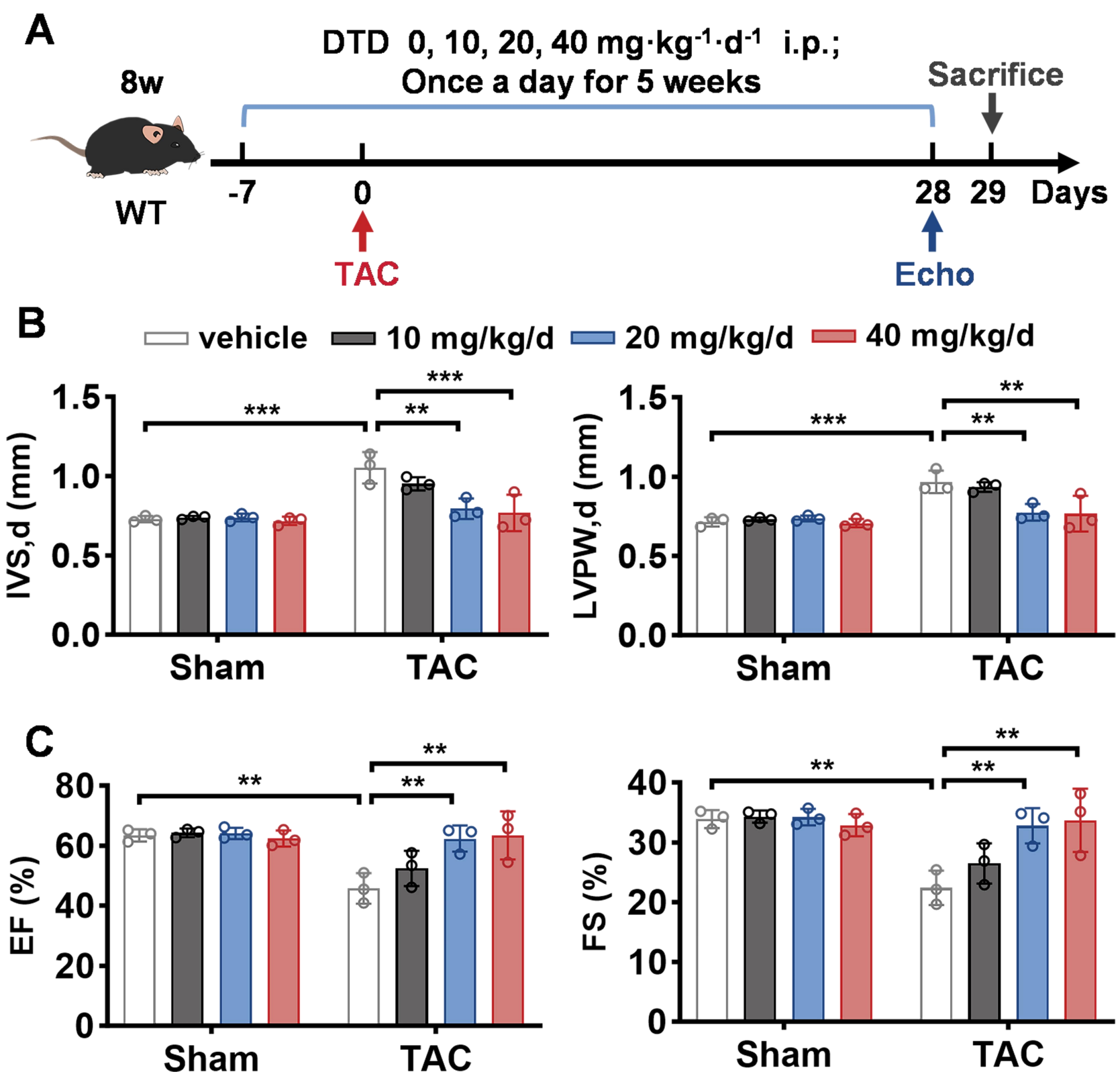

Figure S6. DTD *in vivo* dose finding. A) Schematic diagram depicting the experimental strategy. Eight-week-old male C57BL/6 mice were subjected to sham or TAC surgery, with intraperitoneal administration of DTD (10, 20, 40 mg·kg^{-1}·d^{-1}) or vehicle starting one week prior to surgery and continuing for five weeks. The echocardiographic measurements were performed four weeks after surgery. B–C) Interventricular septum (IVS) thickness, left ventricular posterior wall (LVPW) thickness, ejection fraction (EF) and fractional shortening (FS) were examined in mice (n = 3). Data shown as mean ± SD, $^{**}P < 0.01$, $^{***}P < 0.001$. Statistical analysis assessed by two-way ANOVA with Tukey's post-hoc (B).

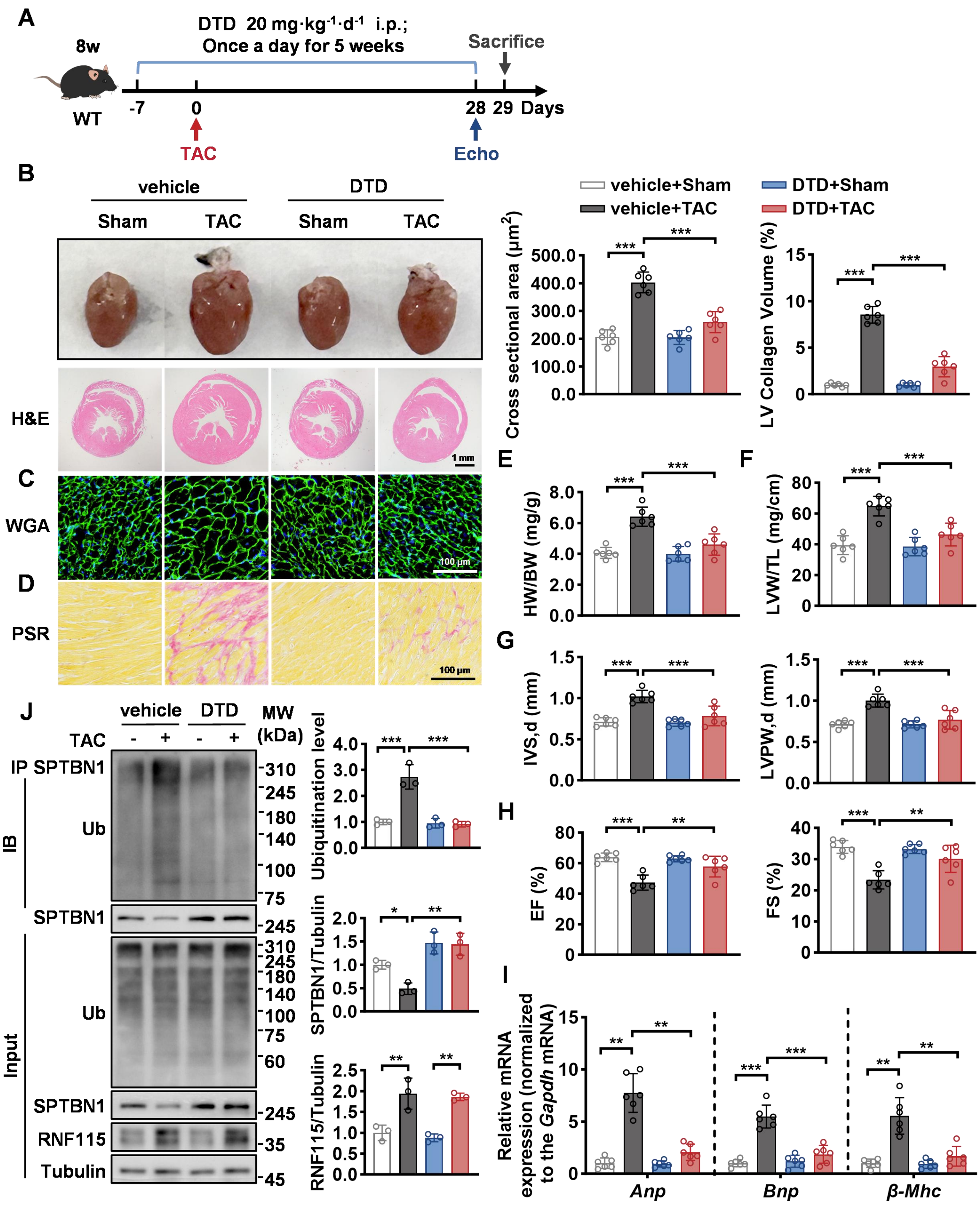

Figure S7. DTD attenuates TAC-induced cardiac hypertrophy in mice. A) Schematic diagram depicting the experimental strategy. Eight-week-old male C57BL/6 mice were subjected to sham or TAC surgery, with intraperitoneal administration of DTD (20 mg·kg^{-1}·d^{-1}) or vehicle starting one week prior to surgery and continuing for five weeks. The echocardiographic measurements were performed four weeks after surgery. B) Representative images of heart from each group. Histological sections of heart tissues were stained with H&E (n = 6). Scale bar = 1 mm. C–D) Histological sections of heart tissues were stained with WGA and PSR,

and the cardiomyocyte cross-sectional area and LV collagen volume were quantified (*n* = 6). Scale bar = 100 μm. E) The ratio of HW/BW were examined in mice (*n* = 6). F) The ratio of LVW/TL were examined in mice (*n* = 6). G–H) IVS, LVPW, EF and FS were examined in mice (*n* = 6). I) The mRNA levels of *Anp*, *Bnp*, and *β-Mhc* in mice heart tissues were determined by qPCR (*n* = 6). J) Ubiquitination level of SPTBN1 and its quantitation was detected in heart tissues from mice (*n* = 3). Data shown as mean ± SD, **P* < 0.05, ***P* < 0.01, ****P* < 0.001. Statistical analysis assessed by one-way ANOVA with Tukey's post-hoc (C–H and J), and Brown-Forsythe and Welch’s ANOVA with Tamhane's T2 post-hoc (I)
