# Supplementary material for: The E3 Ligase RNF115 Aggravates Pathological Cardiac Hypertrophy via Ubiquitin‐Mediated Degradation of SPTBN1
Source: Adv Sci (Weinh). 2026 Jun 15:e76077. Online ahead of print. doi: 10.1002/advs.76077 (PMC13336878; doi:10.1002/advs.76077)
Supplement: Supplementary file 2 — Supporting File: advs76077‐sup‐0002‐Data.zip. [file ADVS-9999-e76077-s001.zip › Microscope Images-R1.pdf]

Figure1C

n=3

N1

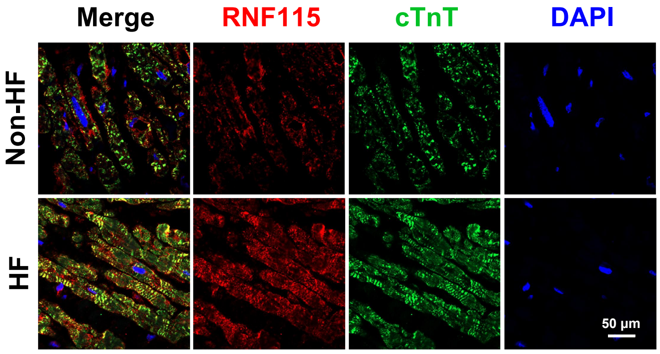

N2

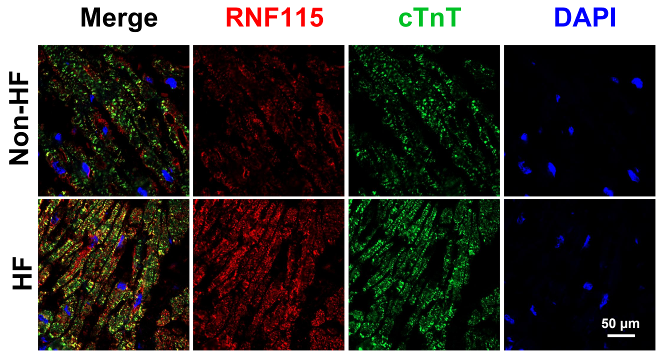

N3

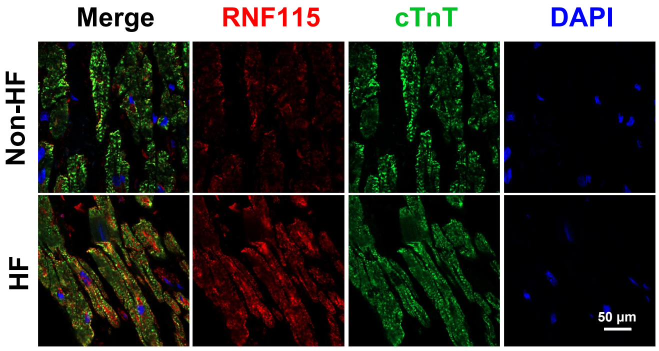

Figure1F

n=3

N1

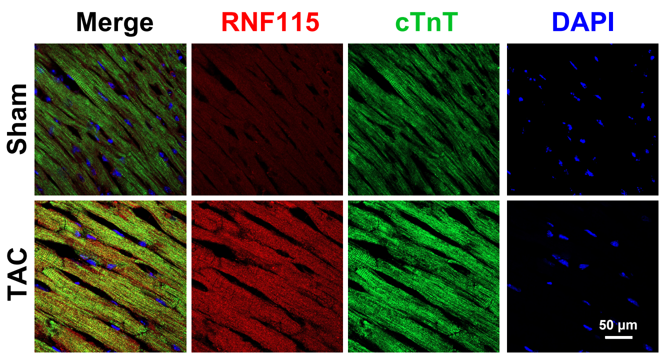

N2

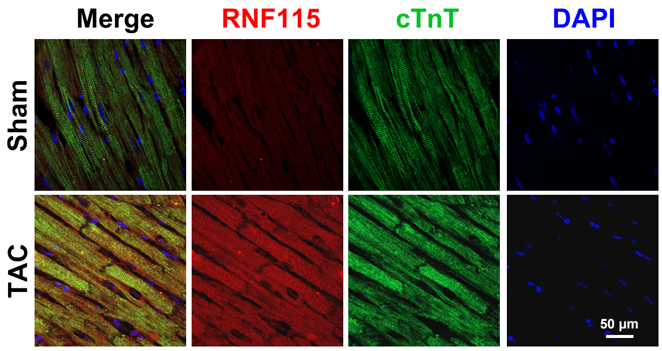

N3

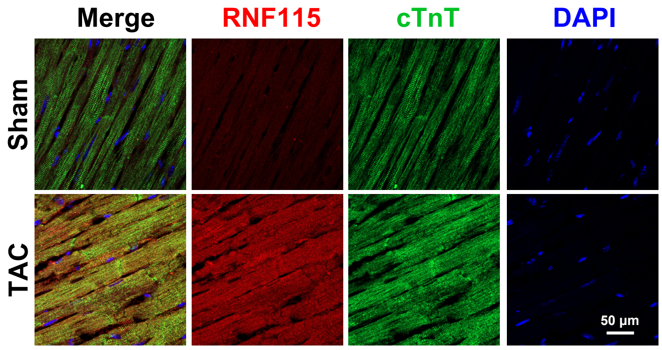

Supplementary Figure2D

N1

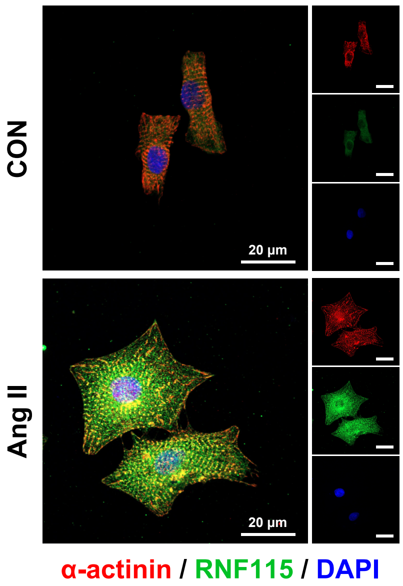

N2

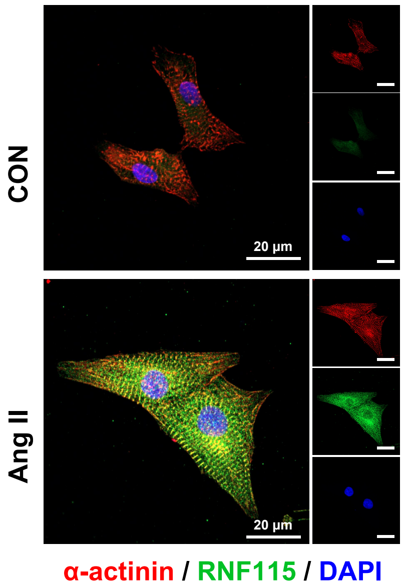

N3

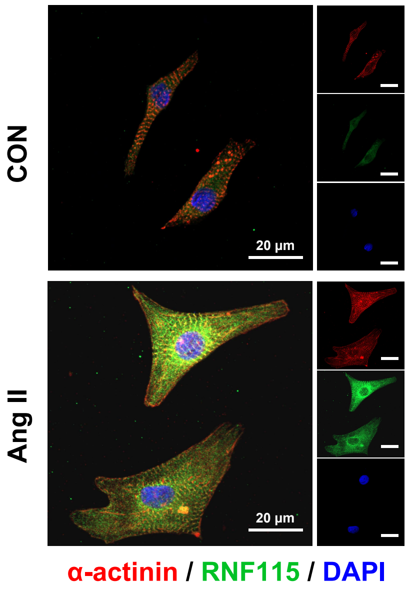

Figure3C

N1

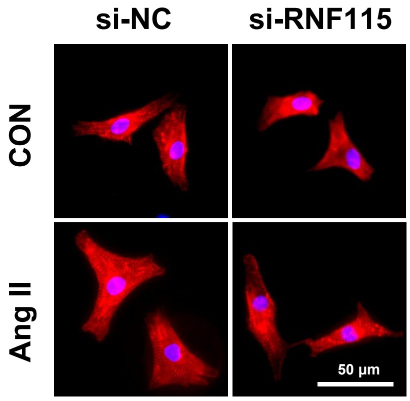

N2

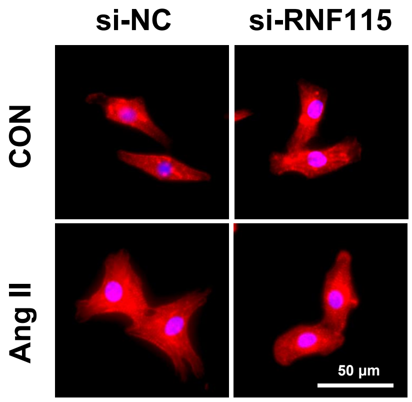

N3

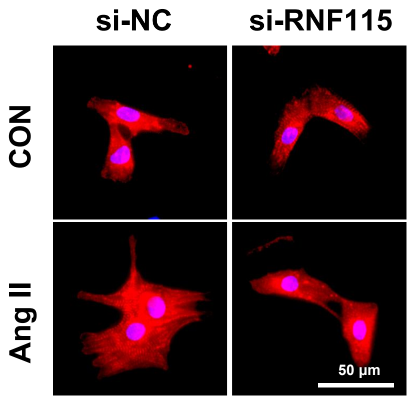

Supplementary Figure4C

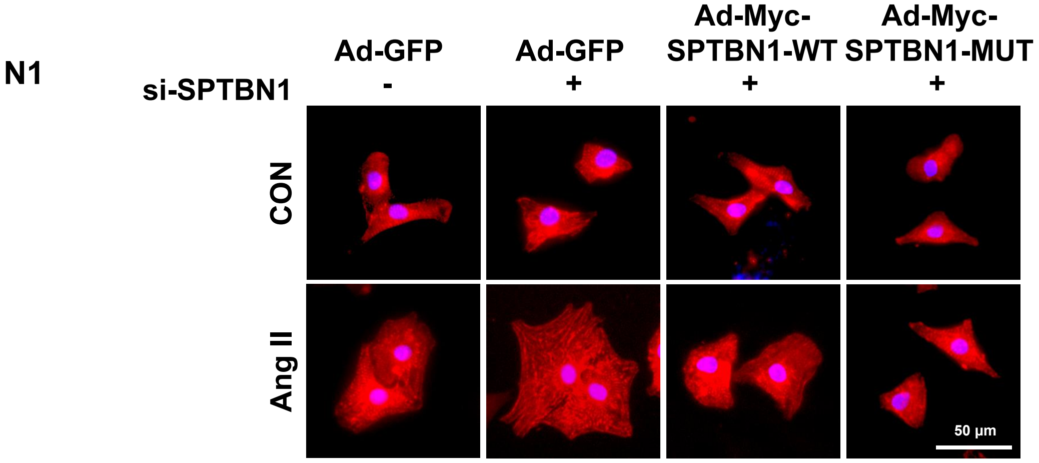

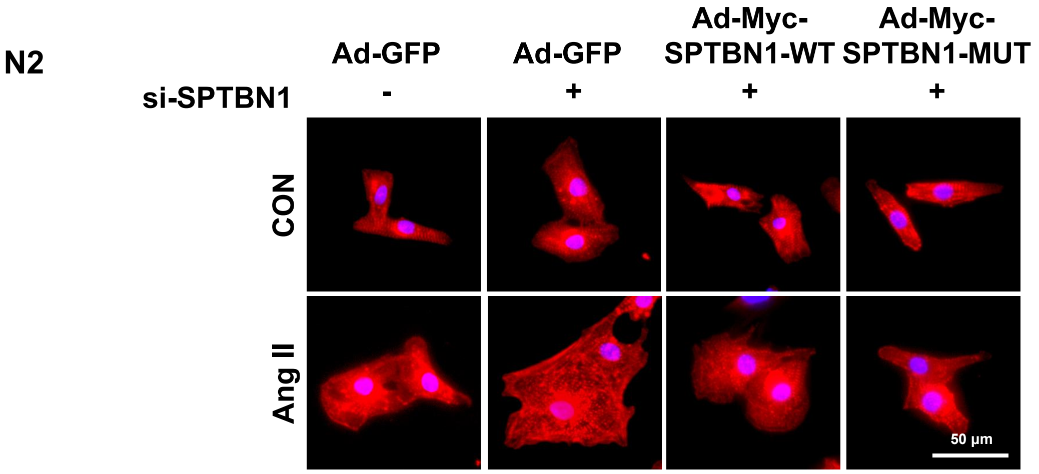

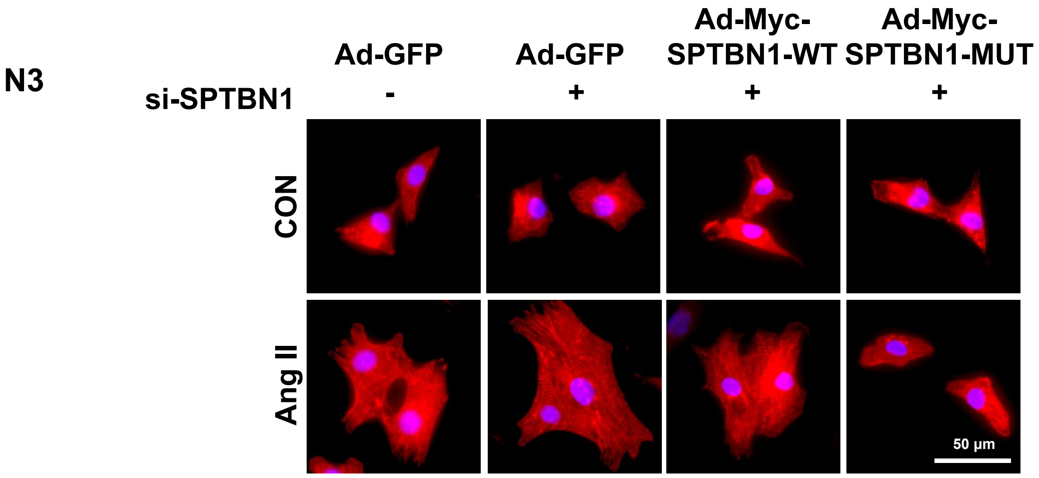

Figure5H

N1

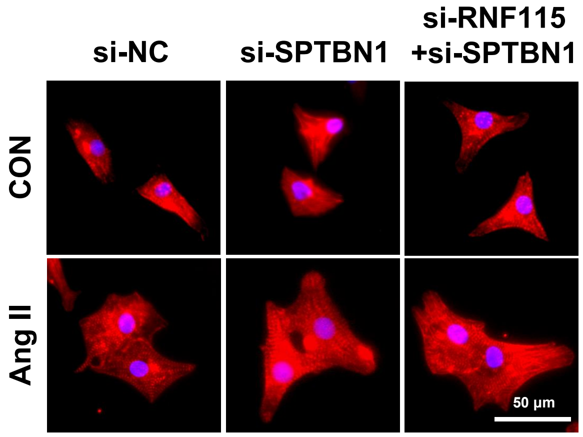

N2

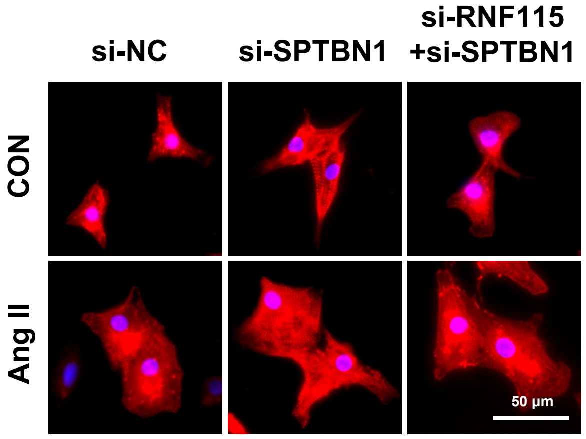

N3

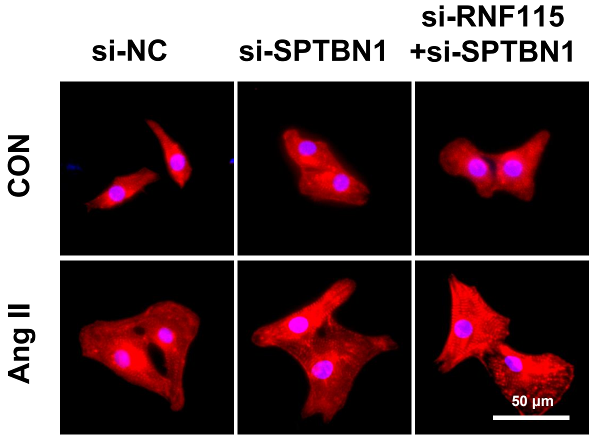

Figure7D

N1

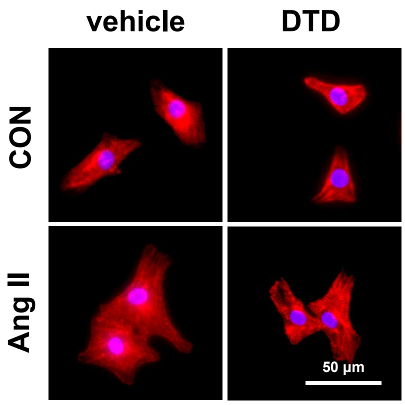

N2

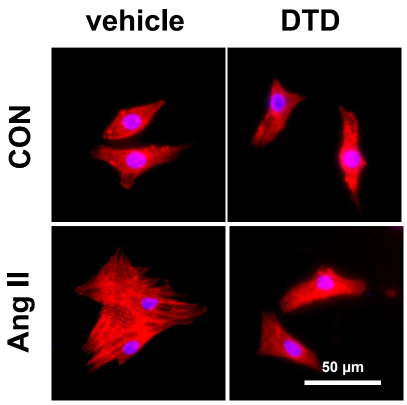

N3

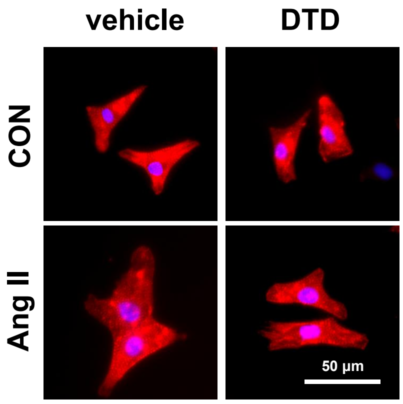

Figure5C

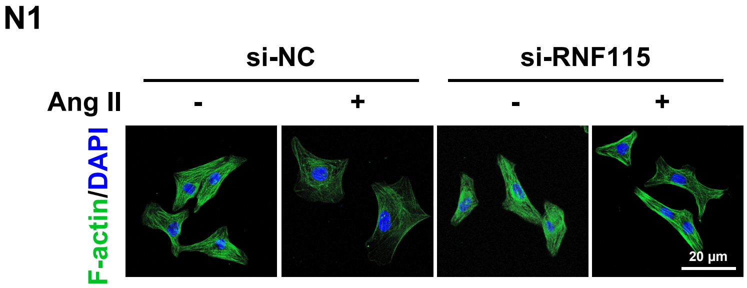
N1
si-NC
si-RNF115
Ang II
-
+
-
+
F-actin/DAPI
20 μm

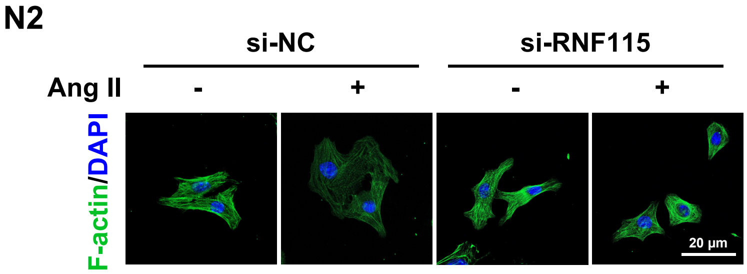
N2
si-NC
si-RNF115
Ang II
-
+
-
+
F-actin/DAPI
20 μm

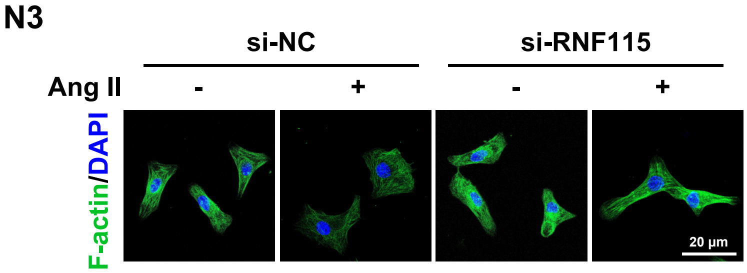
N3
si-NC
si-RNF115
Ang II
-
+
-
+
F-actin/DAPI
20 μm

Supplementary Figure 7B

n=6

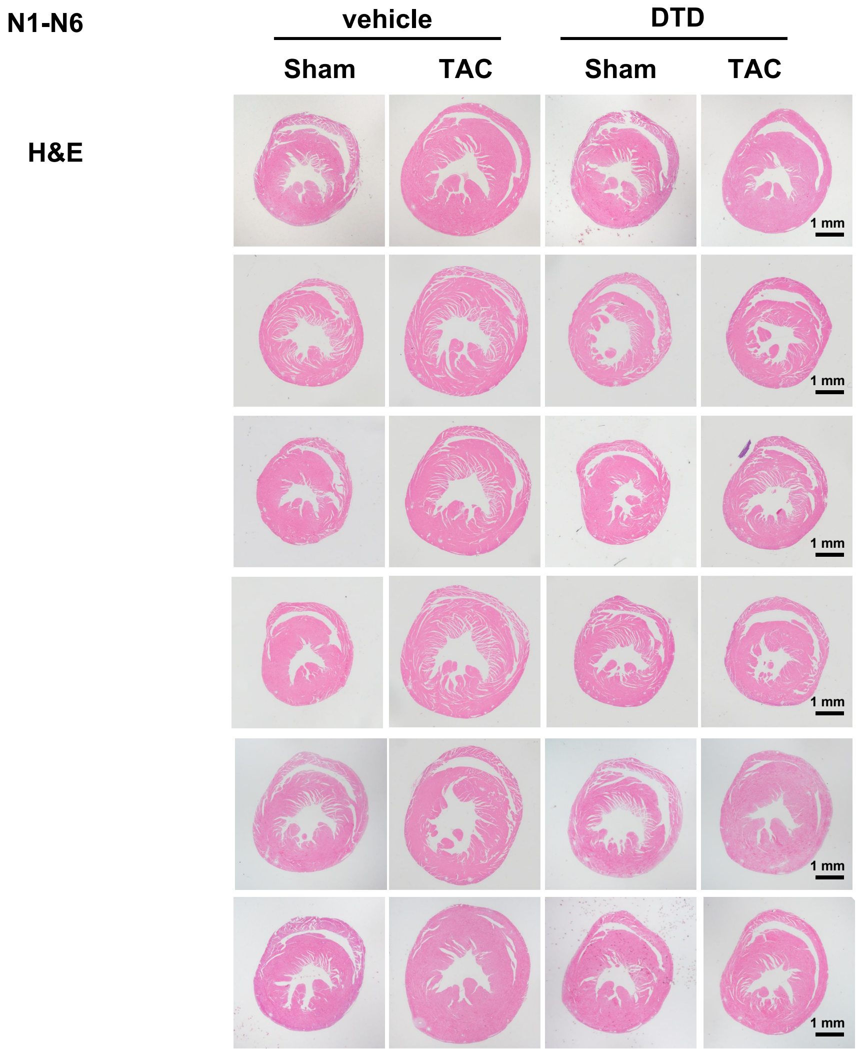

Supplementary Figure 7C

n=6

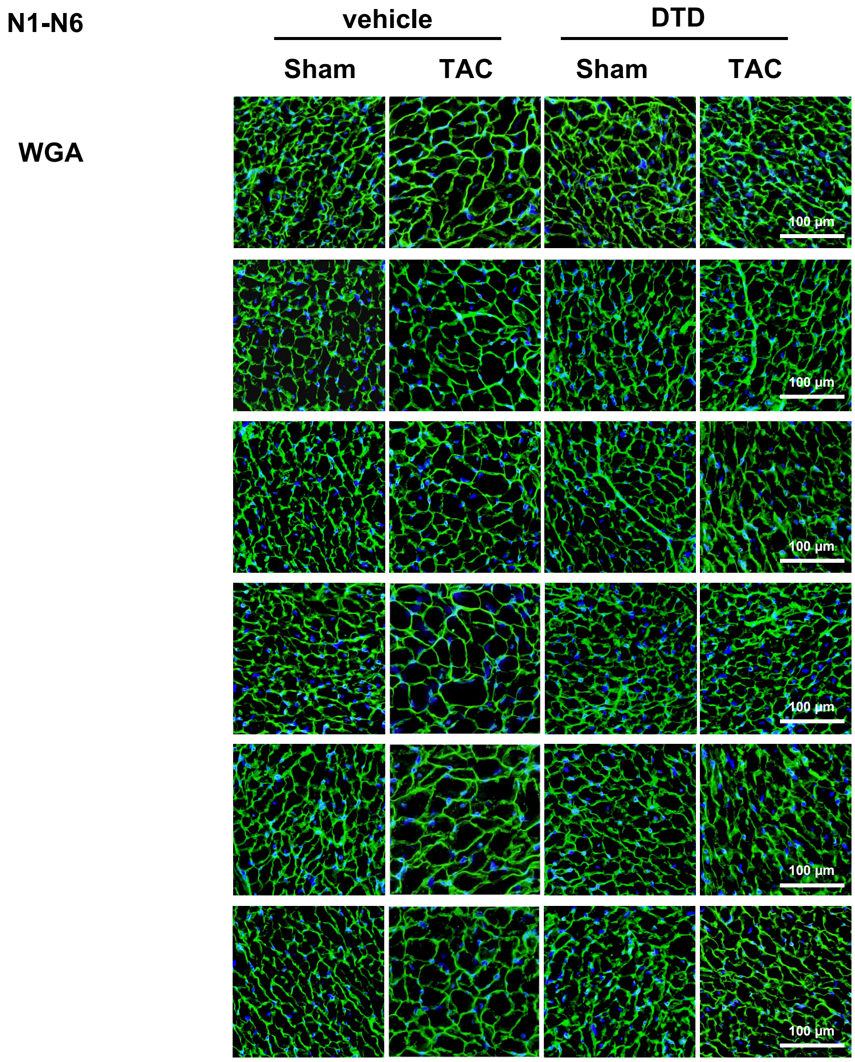

Supplementary Figure 7D

n=6

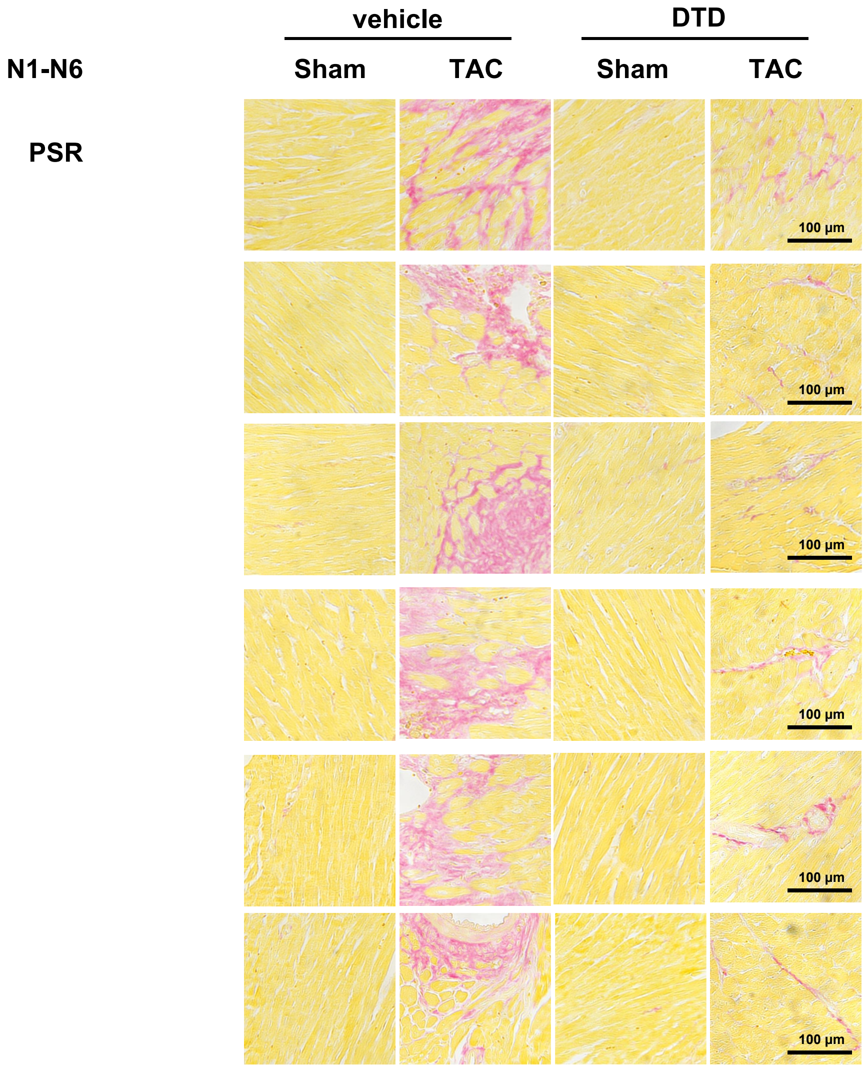

Figure8B

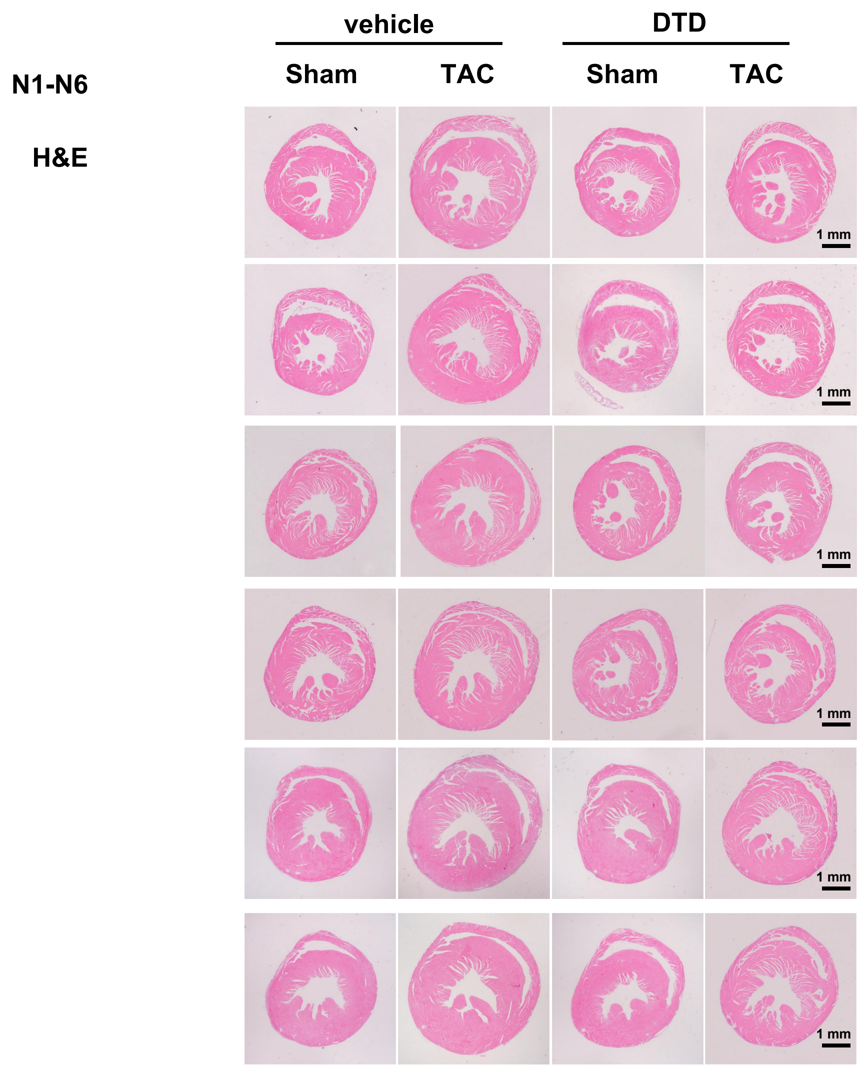

Figure8C

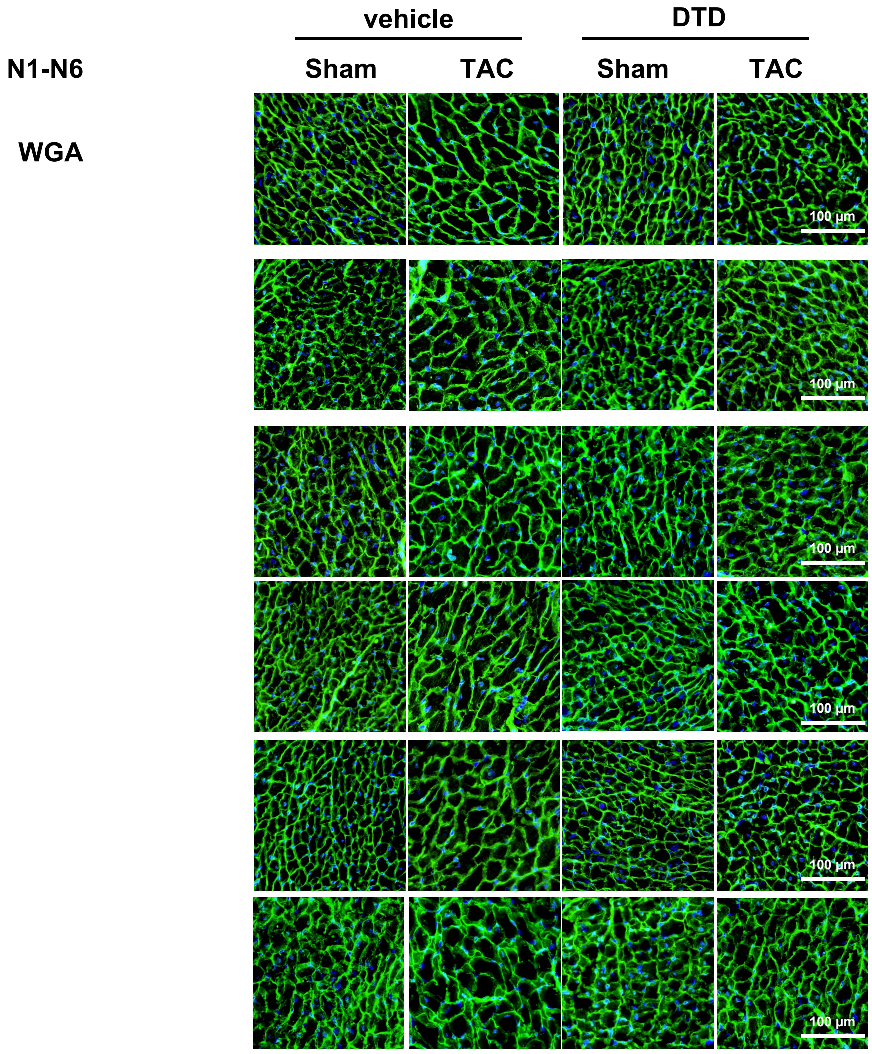
vehicle
DTD
N1-N6
Sham
TAC
Sham
TAC
WGA
100 µm
100 µm
100 µm
100 µm
100 µm
100 µm

Figure8D

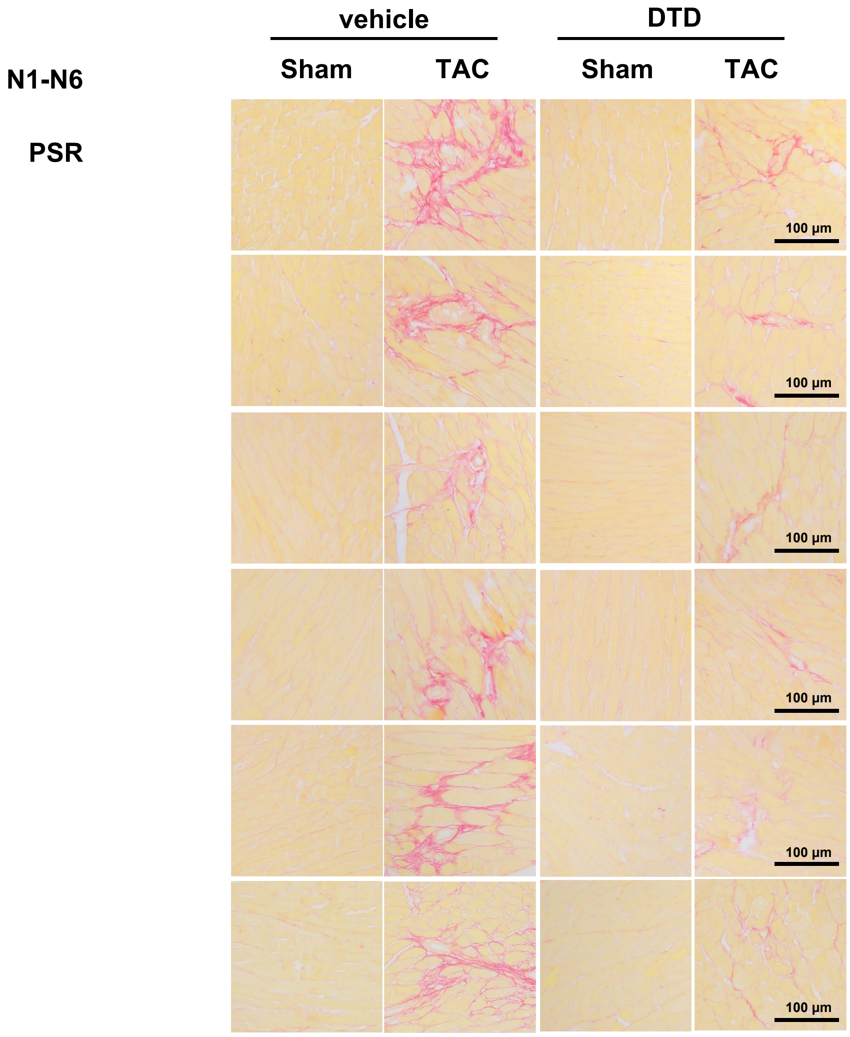

vehicle
DTD
Sham
TAC
Sham
TAC
N1-N6
PSR
100 µm
100 µm
100 µm
100 µm
100 µm
100 µm
