# Supplementary material for: The E3 Ligase RNF115 Aggravates Pathological Cardiac Hypertrophy via Ubiquitin‐Mediated Degradation of SPTBN1
Source: Adv Sci (Weinh). 2026 Jun 15:e76077. Online ahead of print. doi: 10.1002/advs.76077 (PMC13336878; doi:10.1002/advs.76077)
Supplement: Supplementary file 2 — Supporting File: advs76077‐sup‐0002‐Data.zip. [file ADVS-9999-e76077-s001.zip › RNF115 uncut gels-R1.pdf]

Figure1D

n=6

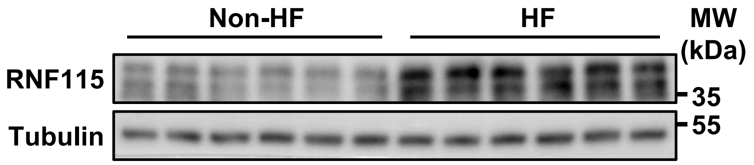

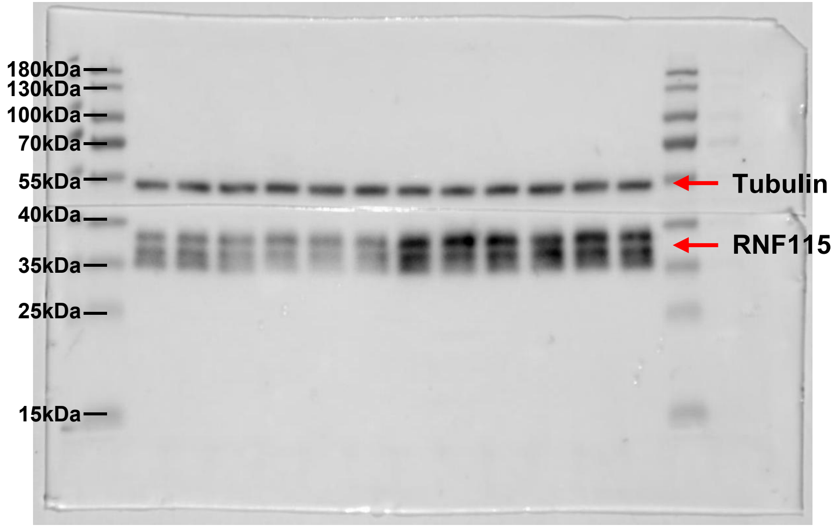

Figure1G

n=6

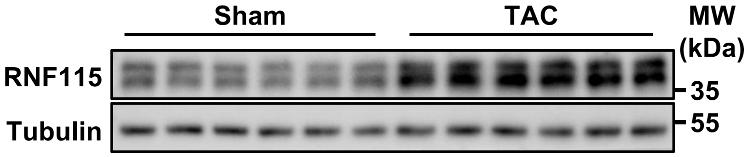

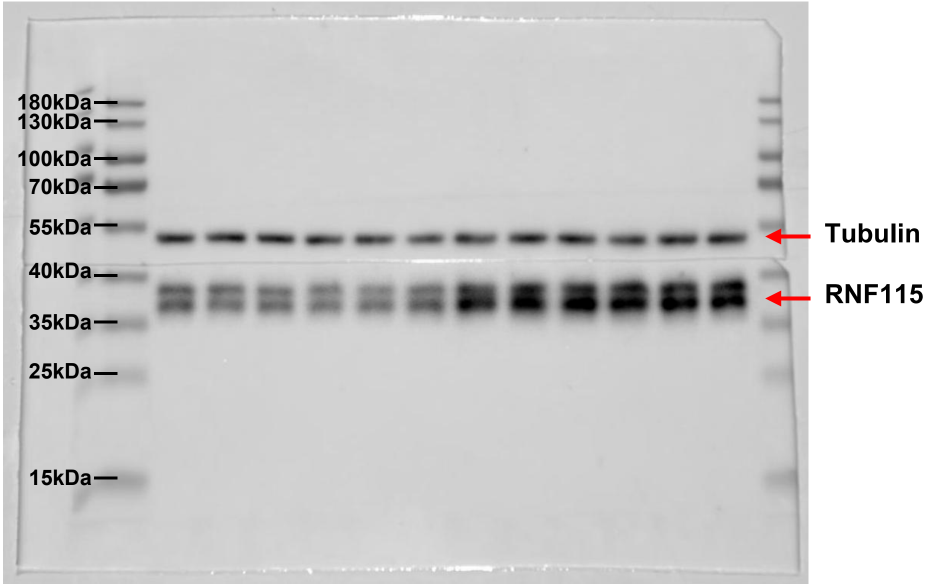

Figure1I

n=6

N1-N3

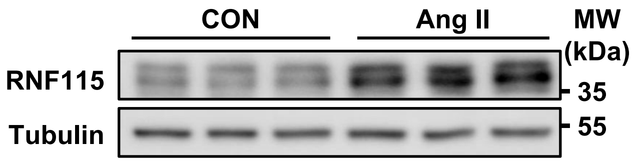

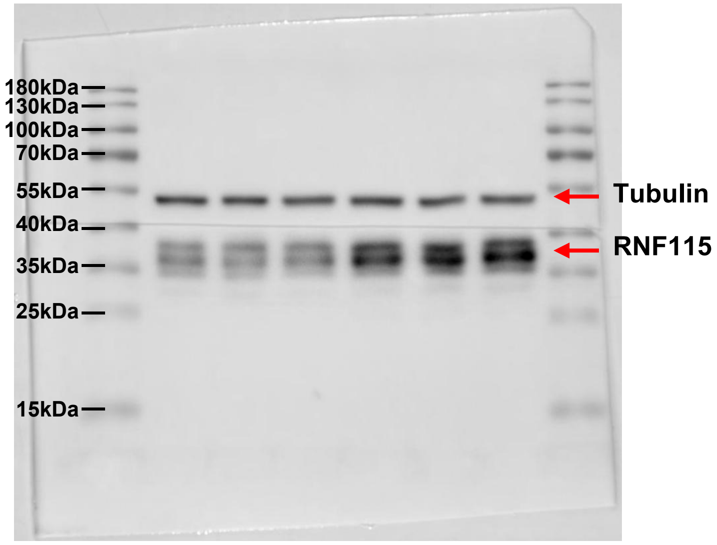

N4-N6

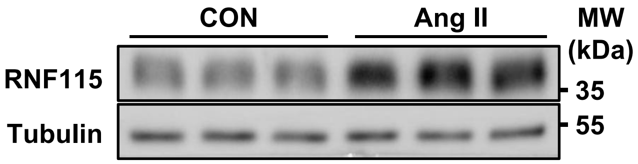

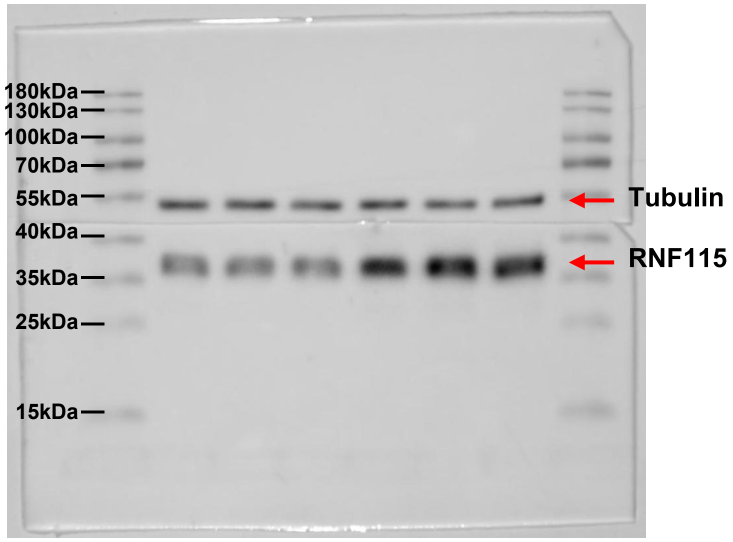

Figure2A

n=3

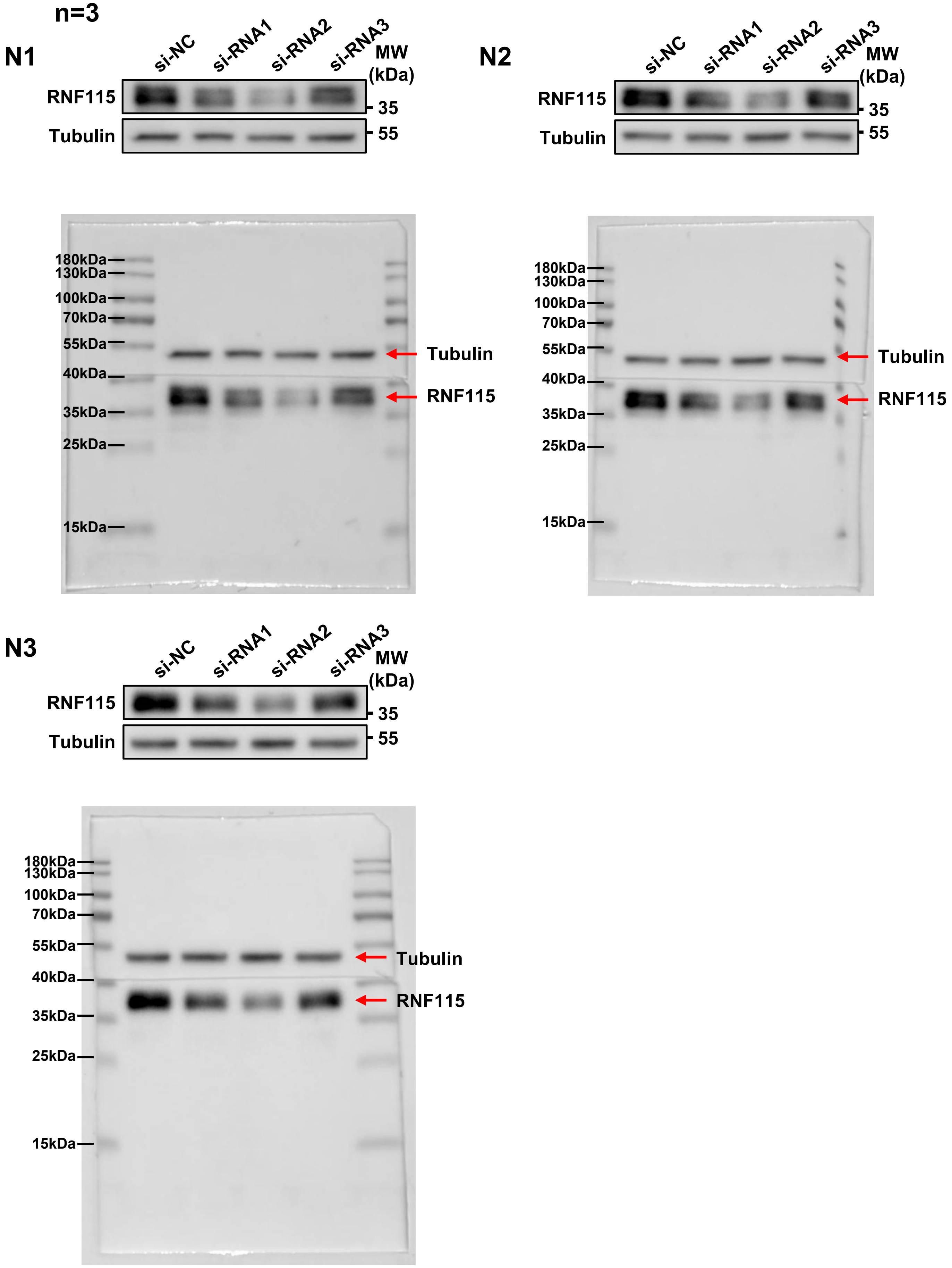
N1
si-NC
si-RNA1
si-RNA2
si-RNA3
MW (kDa)
RNF115
Tubulin
35
55
180kDa
130kDa
100kDa
70kDa
55kDa
40kDa
35kDa
25kDa
15kDa
Tubulin
RNF115
N2
si-NC
si-RNA1
si-RNA2
si-RNA3
MW (kDa)
RNF115
Tubulin
35
55
180kDa
130kDa
100kDa
70kDa
55kDa
40kDa
35kDa
25kDa
15kDa
Tubulin
RNF115
N3
si-NC
si-RNA1
si-RNA2
si-RNA3
MW (kDa)
RNF115
Tubulin
35
55
180kDa
130kDa
100kDa
70kDa
55kDa
40kDa
35kDa
25kDa
15kDa
Tubulin
RNF115

Figure2F

n=6

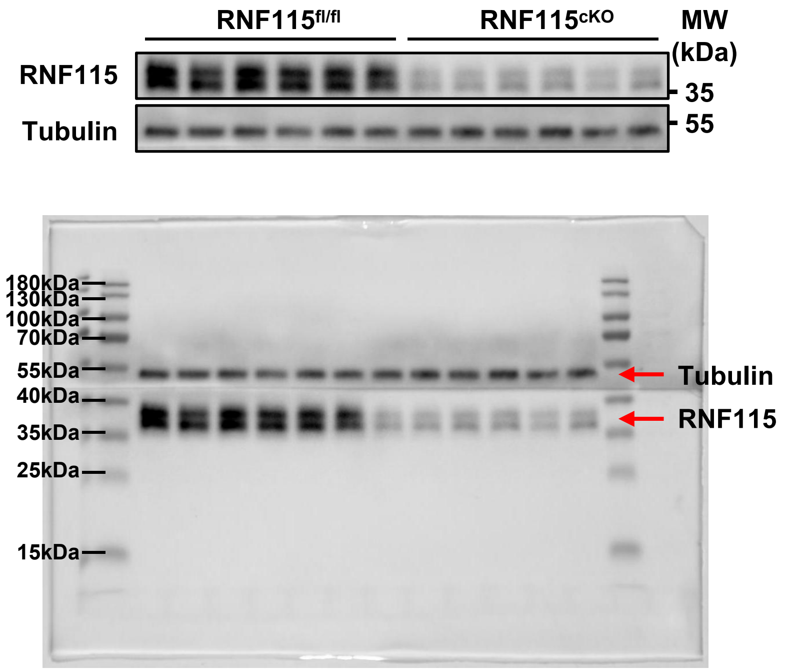
RNF115fl/fl
RNF115cKO
MW (kDa)
RNF115
Tubulin
35
55
180kDa
130kDa
100kDa
70kDa
55kDa
40kDa
35kDa
25kDa
15kDa
Tubulin
RNF115

Figure3D

n=3

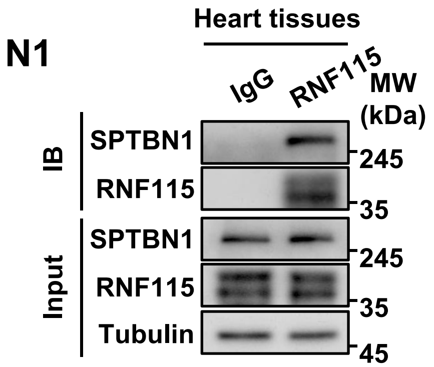
N1
Heart tissues
IgG
RNF115
MW
(kDa)
IB
SPTBN1
RNF115
Input
SPTBN1
RNF115
Tubulin
245
35
245
35
45

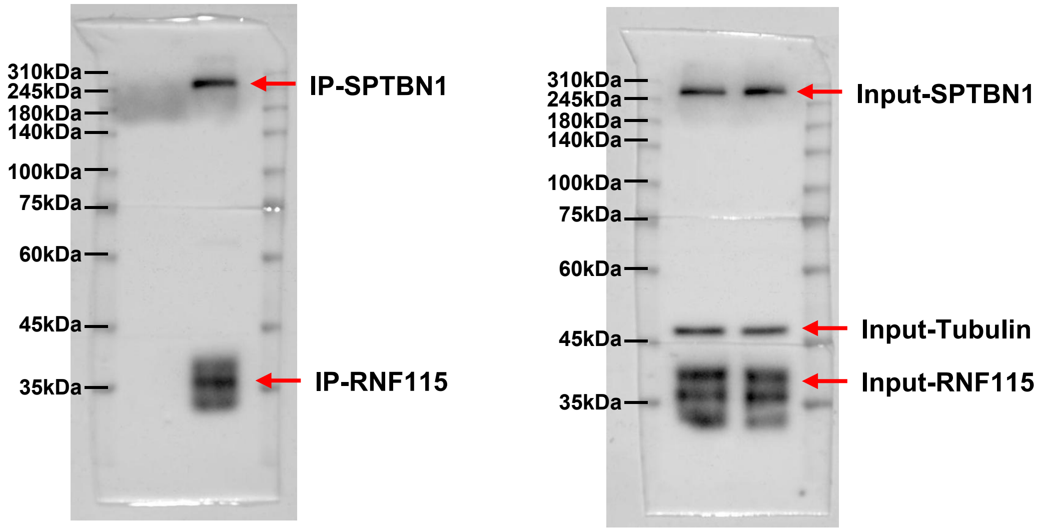
310kDa
245kDa
180kDa
140kDa
100kDa
75kDa
60kDa
45kDa
35kDa
IP-SPTBN1
IP-RNF115
Input-SPTBN1
Input-Tubulin
Input-RNF115

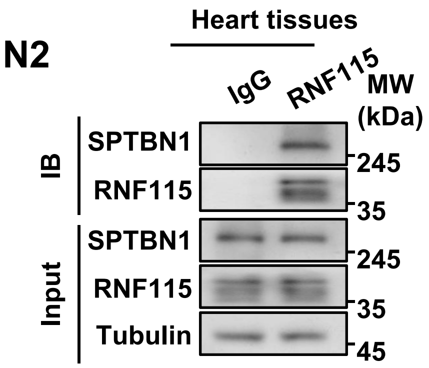
N2
Heart tissues
IgG
RNF115
MW
(kDa)
IB
SPTBN1
RNF115
Input
SPTBN1
RNF115
Tubulin
245
35
245
35
45

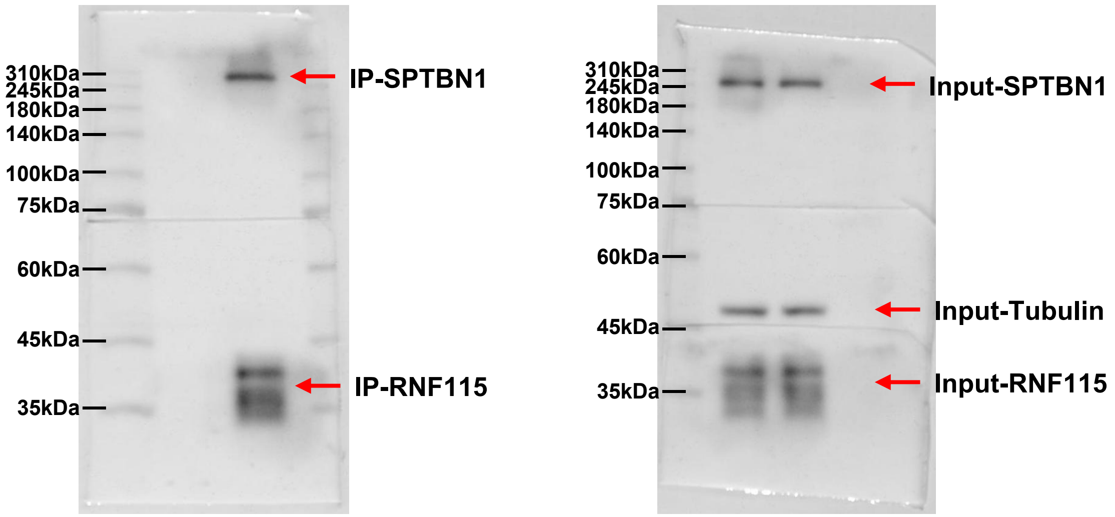
310kDa
245kDa
180kDa
140kDa
100kDa
75kDa
60kDa
45kDa
35kDa
IP-SPTBN1
IP-RNF115
Input-SPTBN1
Input-Tubulin
Input-RNF115

N3

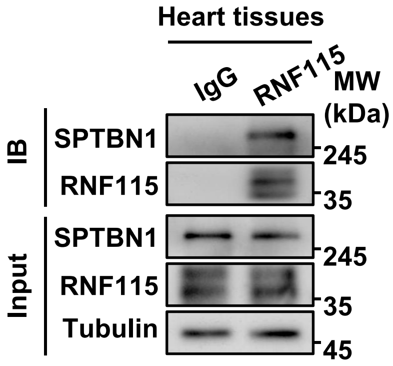
Heart tissues
IgG
RNF115
MW (kDa)
IB
SPTBN1
RNF115
Input
SPTBN1
RNF115
Tubulin
245
35
245
35
45

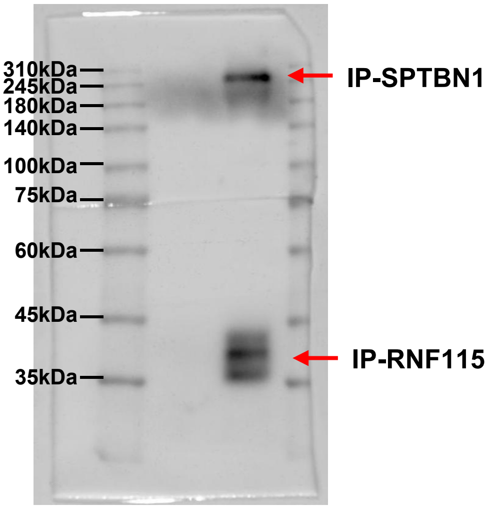
310kDa
245kDa
180kDa
140kDa
100kDa
75kDa
60kDa
45kDa
35kDa
IP-SPTBN1
IP-RNF115

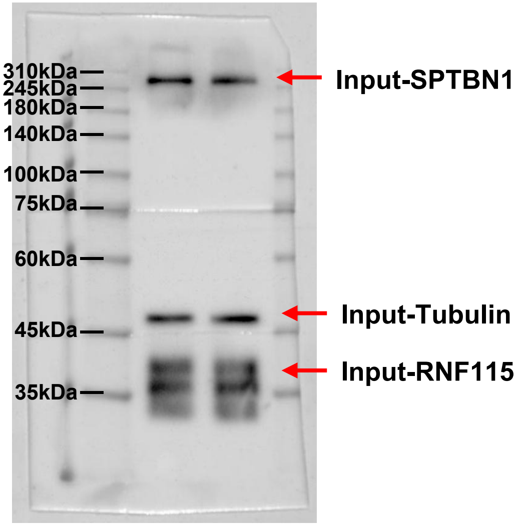
310kDa
245kDa
180kDa
140kDa
100kDa
75kDa
60kDa
45kDa
35kDa
Input-SPTBN1
Input-Tubulin
Input-RNF115

Figure3E

n=3

N1

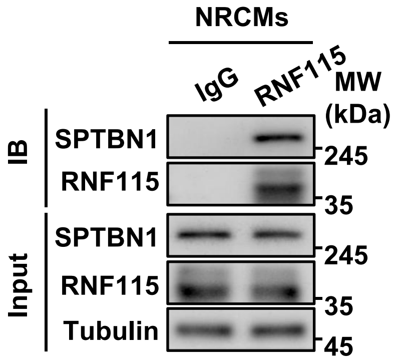

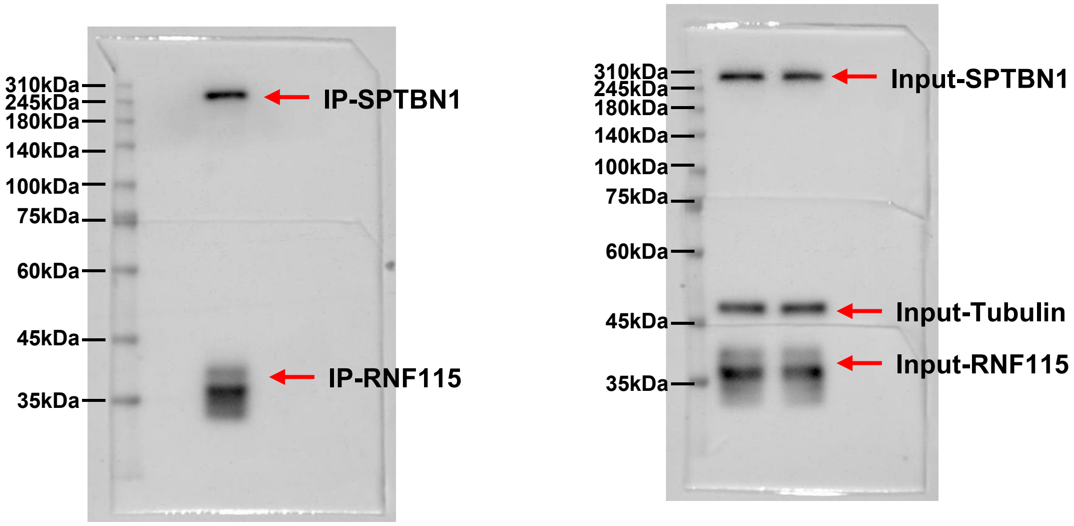

N2

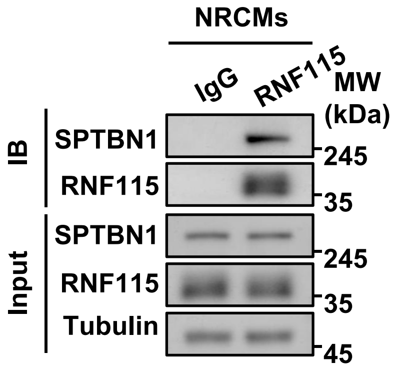

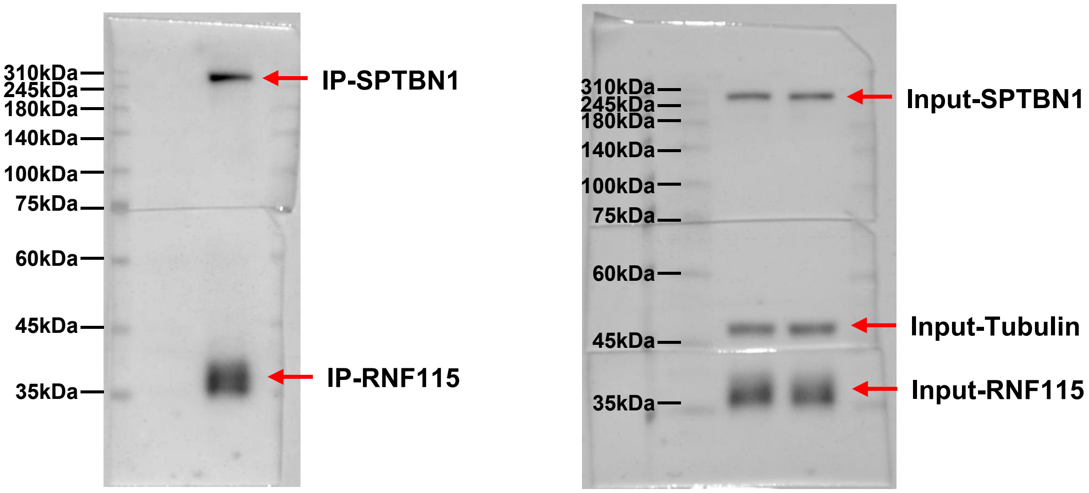

N3

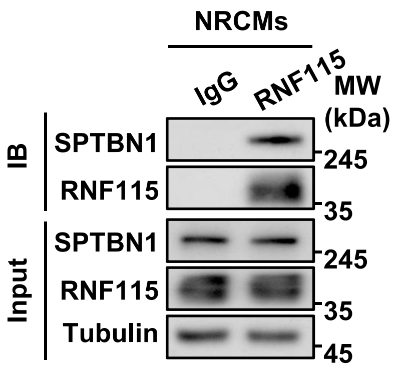

NRCMs
IgG
RNF115
MW
(kDa)
IB
SPTBN1
RNF115
Input
SPTBN1
RNF115
Tubulin
245
35
245
35
45

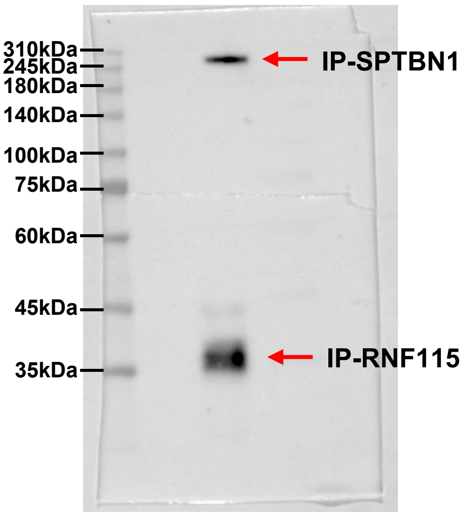

310kDa
245kDa
180kDa
140kDa
100kDa
75kDa
60kDa
45kDa
35kDa
IP-SPTBN1
IP-RNF115

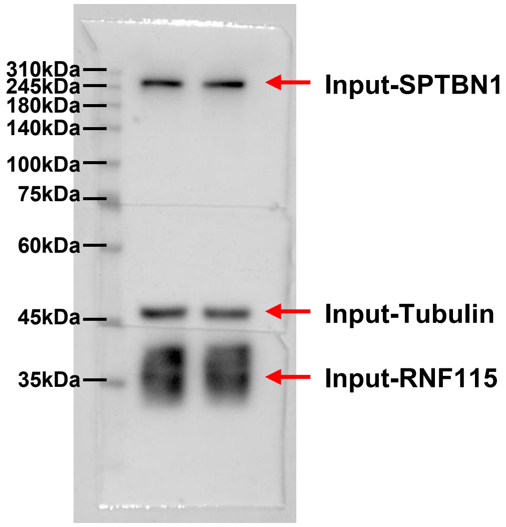

310kDa
245kDa
180kDa
140kDa
100kDa
75kDa
60kDa
45kDa
35kDa
Input-SPTBN1
Input-Tubulin
Input-RNF115

Figure3F

n=3

N1

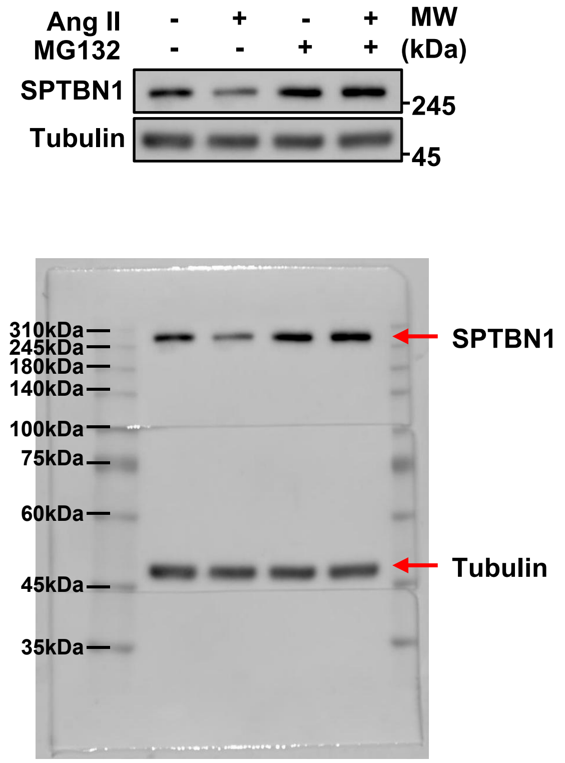

N2

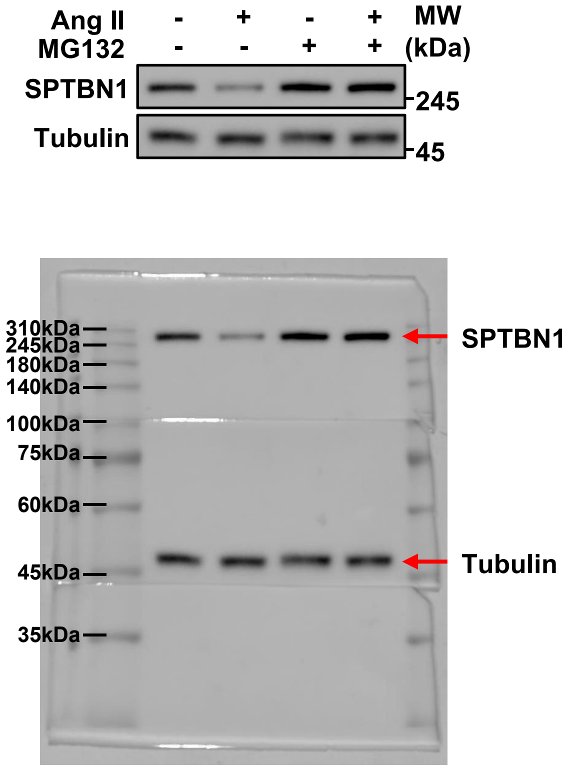

N3

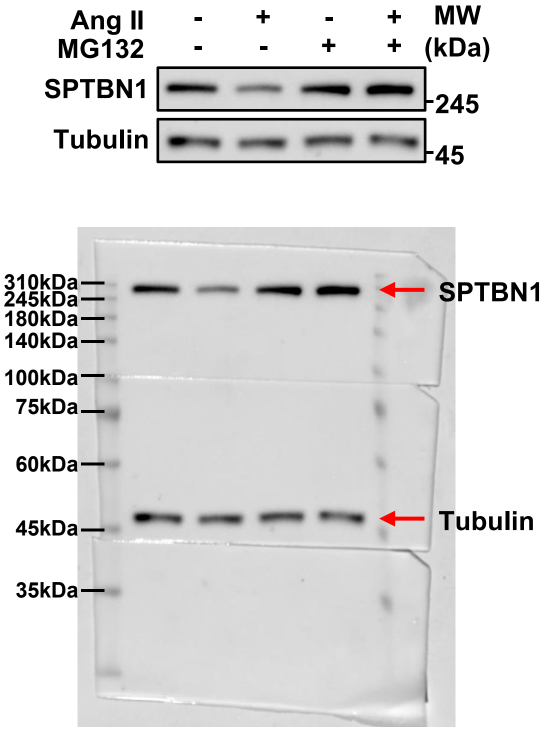

Figure3G

n=3

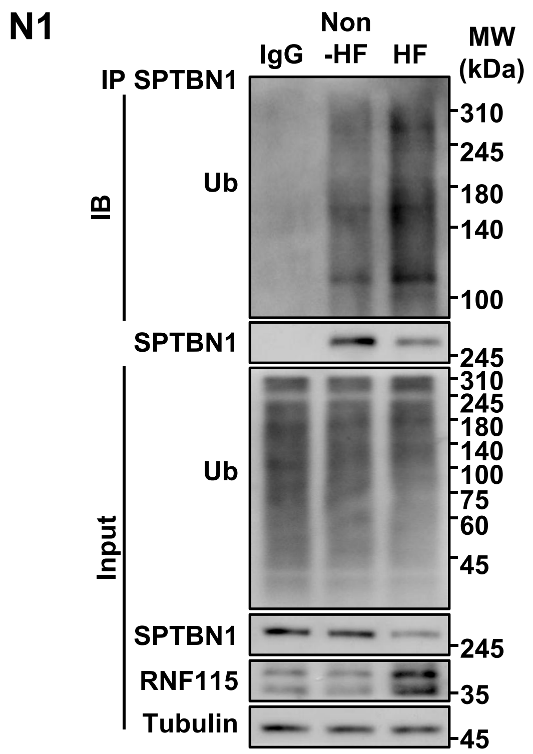
N1
IgG
Non -HF
HF
MW (kDa)
IP SPTBN1
IB
Ub
310
245
180
140
100
SPTBN1
245
Input
Ub
310
245
180
140
100
75
60
45
SPTBN1
245
RNF115
35
Tubulin
45

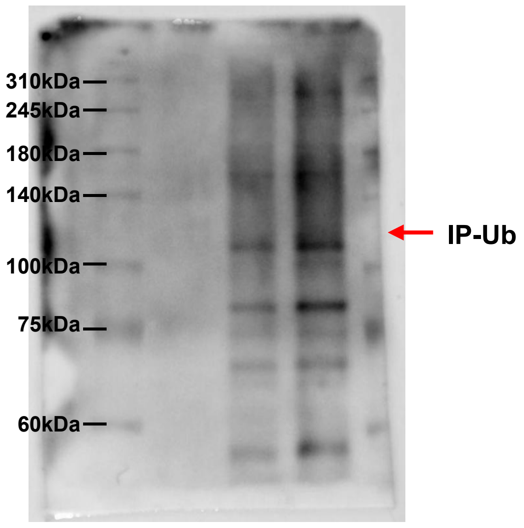
310kDa
245kDa
180kDa
140kDa
100kDa
75kDa
60kDa
IP-Ub

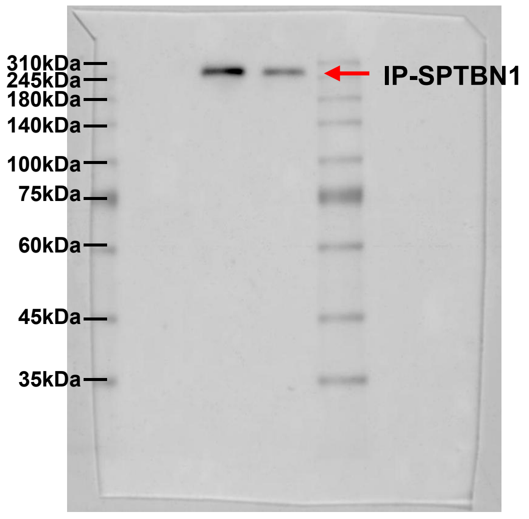
310kDa
245kDa
180kDa
140kDa
100kDa
75kDa
60kDa
45kDa
35kDa
IP-SPTBN1

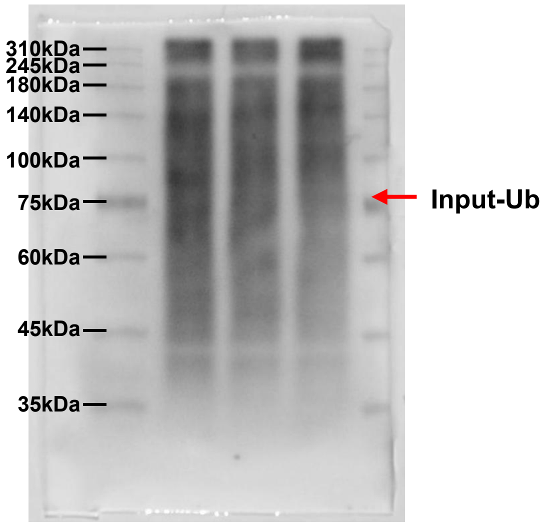
310kDa
245kDa
180kDa
140kDa
100kDa
75kDa
60kDa
45kDa
35kDa
Input-Ub

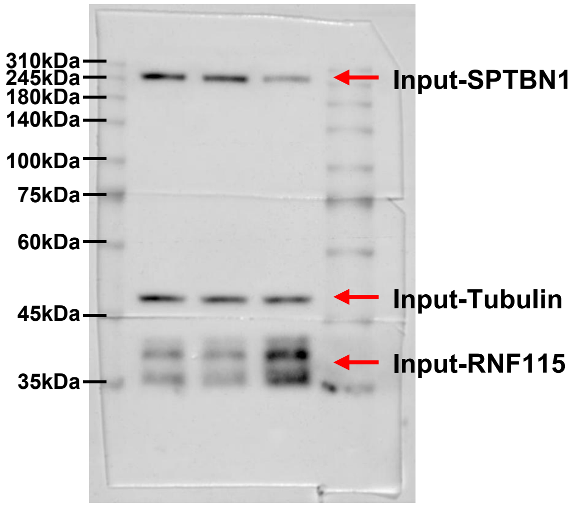
310kDa
245kDa
180kDa
140kDa
100kDa
75kDa
60kDa
45kDa
35kDa
Input-SPTBN1
Input-Tubulin
Input-RNF115

N2

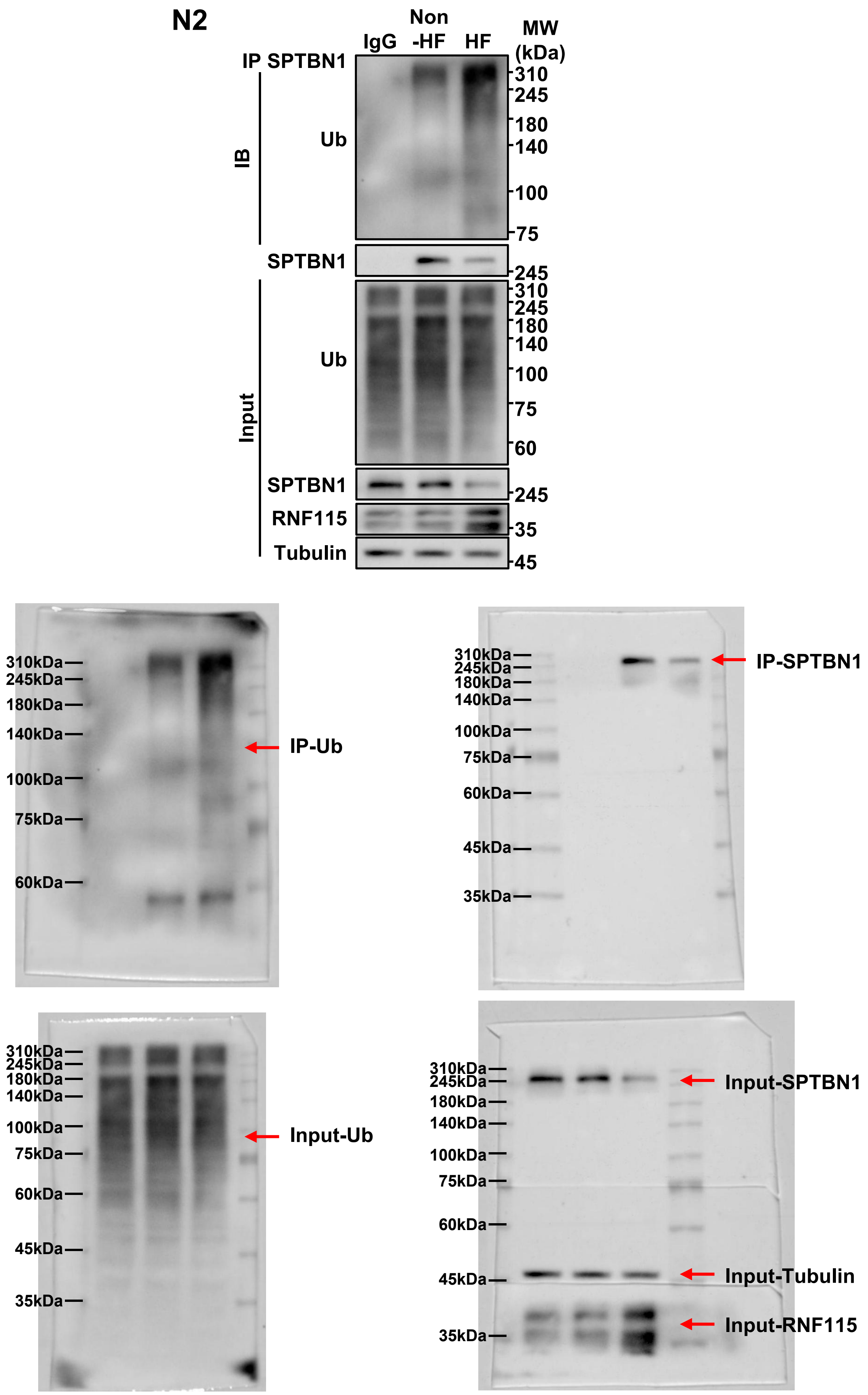

IgG
Non -HF
HF
MW (kDa)
IP SPTBN1
IB
Ub
310
245
180
140
100
75
SPTBN1
245
Input
Ub
310
245
180
140
100
75
60
SPTBN1
245
RNF115
35
Tubulin
45
310kDa
245kDa
180kDa
140kDa
100kDa
75kDa
60kDa
IP-Ub
310kDa
245kDa
180kDa
140kDa
100kDa
75kDa
60kDa
45kDa
35kDa
IP-SPTBN1
310kDa
245kDa
180kDa
140kDa
100kDa
75kDa
60kDa
45kDa
35kDa
Input-Ub
310kDa
245kDa
180kDa
140kDa
100kDa
75kDa
60kDa
45kDa
35kDa
Input-SPTBN1
Input-Tubulin
Input-RNF115

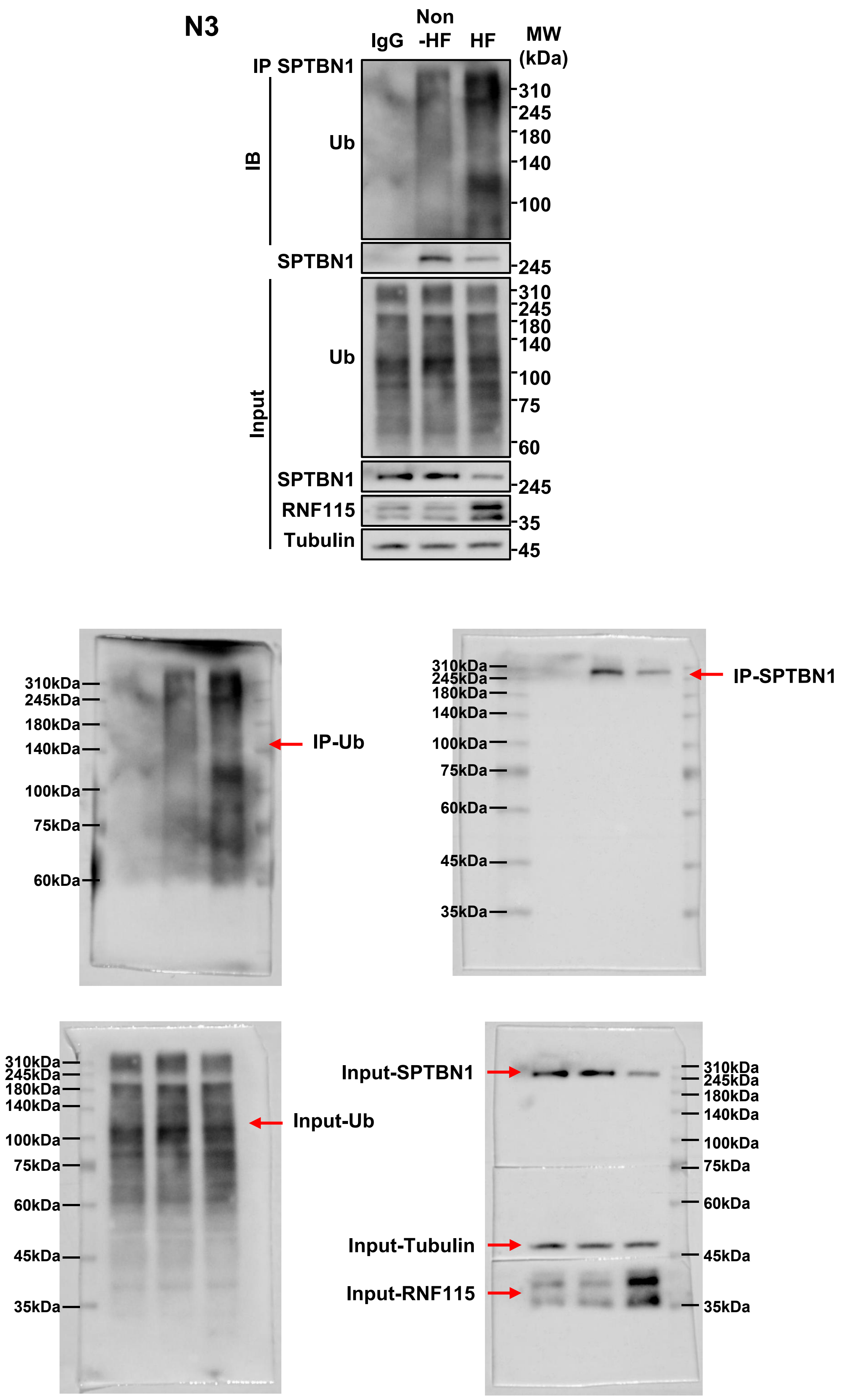

N3
IgG
Non -HF
HF
MW (kDa)
IP SPTBN1
IB
Ub
310
245
180
140
100
SPTBN1
245
Input
Ub
310
245
180
140
100
75
60
SPTBN1
245
RNF115
35
Tubulin
45
310kDa
245kDa
180kDa
140kDa
100kDa
75kDa
60kDa
IP-Ub
310kDa
245kDa
180kDa
140kDa
100kDa
75kDa
60kDa
45kDa
35kDa
IP-SPTBN1
310kDa
245kDa
180kDa
140kDa
100kDa
75kDa
60kDa
45kDa
35kDa
Input-Ub
Input-SPTBN1
Input-Tubulin
Input-RNF115
310kDa
245kDa
180kDa
140kDa
100kDa
75kDa
60kDa
45kDa
35kDa

Figure3H

n=3

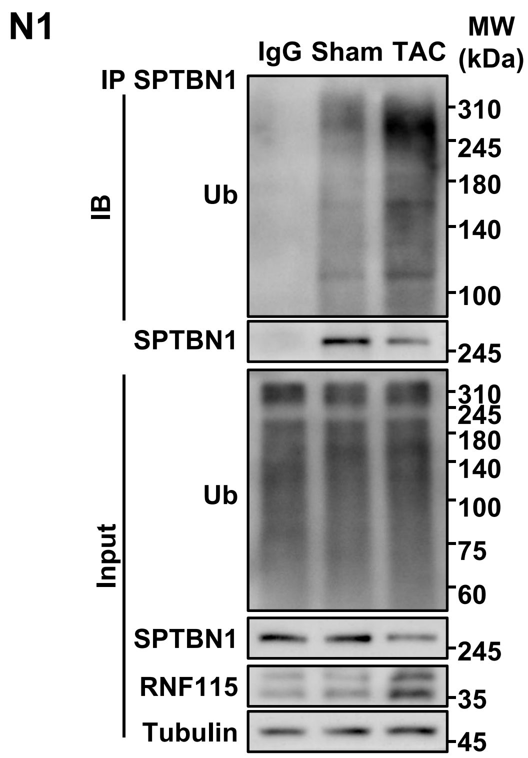
N1
IgG Sham TAC
MW (kDa)
IP SPTBN1
IB
Ub
SPTBN1
Input
Ub
SPTBN1
RNF115
Tubulin
310
245
180
140
100
245
310
245
180
140
100
75
60
245
35
45

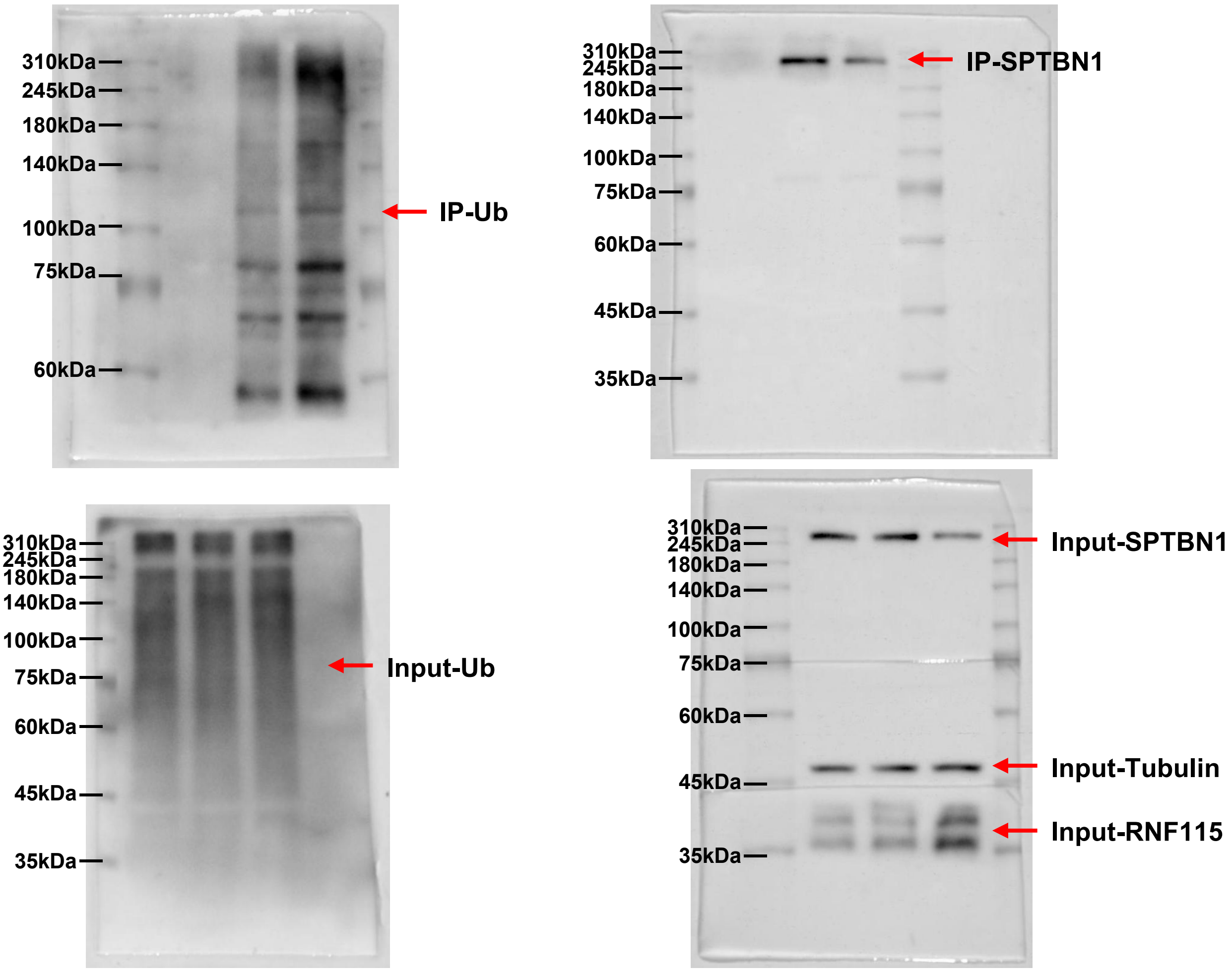
310kDa
245kDa
180kDa
140kDa
100kDa
75kDa
60kDa
IP-Ub
310kDa
245kDa
180kDa
140kDa
100kDa
75kDa
60kDa
45kDa
35kDa
IP-SPTBN1
310kDa
245kDa
180kDa
140kDa
100kDa
75kDa
60kDa
45kDa
35kDa
Input-Ub
310kDa
245kDa
180kDa
140kDa
100kDa
75kDa
60kDa
45kDa
35kDa
Input-SPTBN1
Input-Tubulin
Input-RNF115

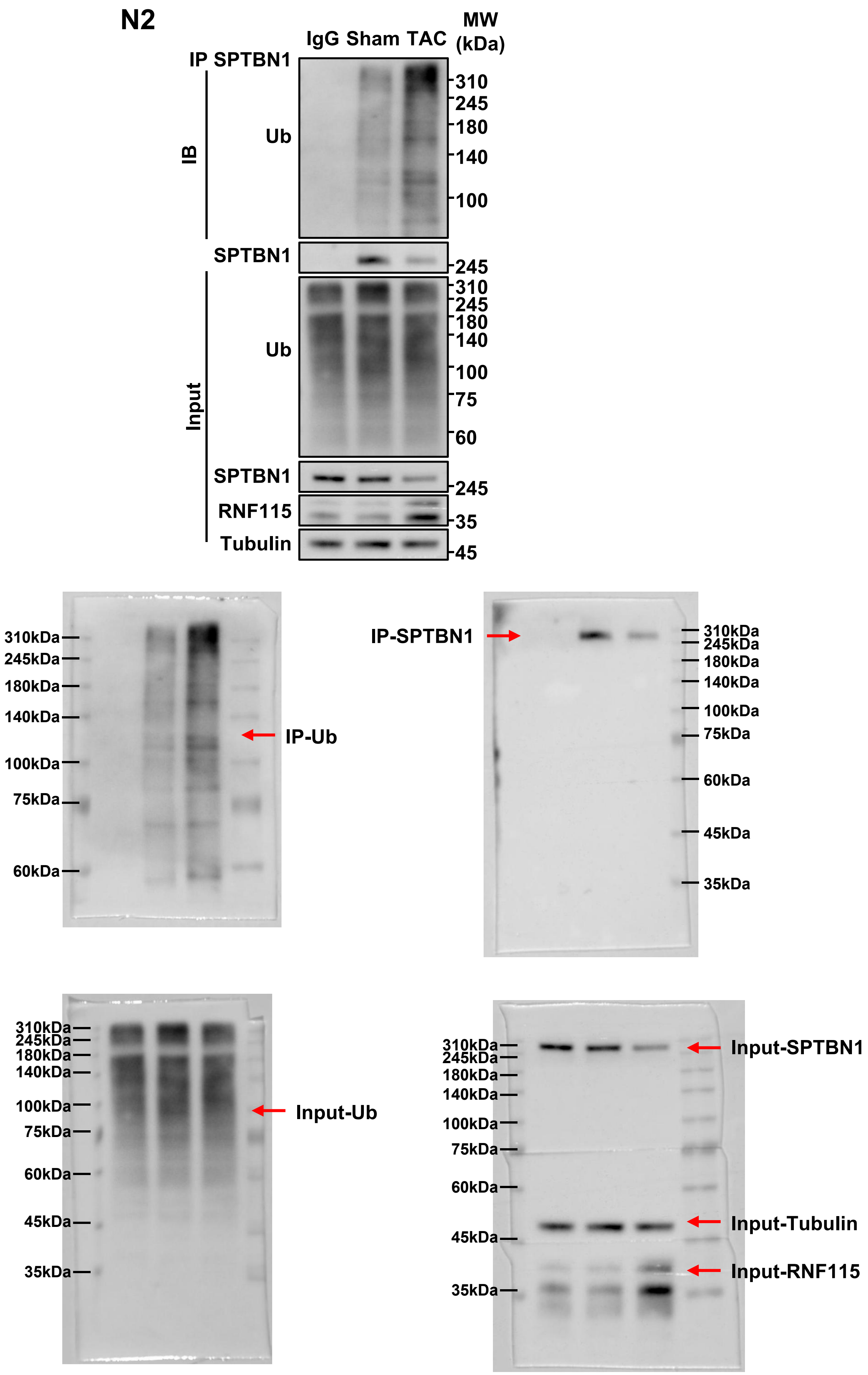
N2
IgG Sham TAC
MW (kDa)
IP SPTBN1
IB
Ub
310
245
180
140
100
SPTBN1
245
Input
Ub
310
245
180
140
100
75
60
SPTBN1
245
RNF115
35
Tubulin
45
310kDa
245kDa
180kDa
140kDa
100kDa
75kDa
60kDa
IP-Ub
IP-SPTBN1
310kDa
245kDa
180kDa
140kDa
100kDa
75kDa
60kDa
45kDa
35kDa
310kDa
245kDa
180kDa
140kDa
100kDa
75kDa
60kDa
45kDa
35kDa
Input-Ub
310kDa
245kDa
180kDa
140kDa
100kDa
75kDa
60kDa
45kDa
35kDa
Input-SPTBN1
Input-Tubulin
Input-RNF115

N3

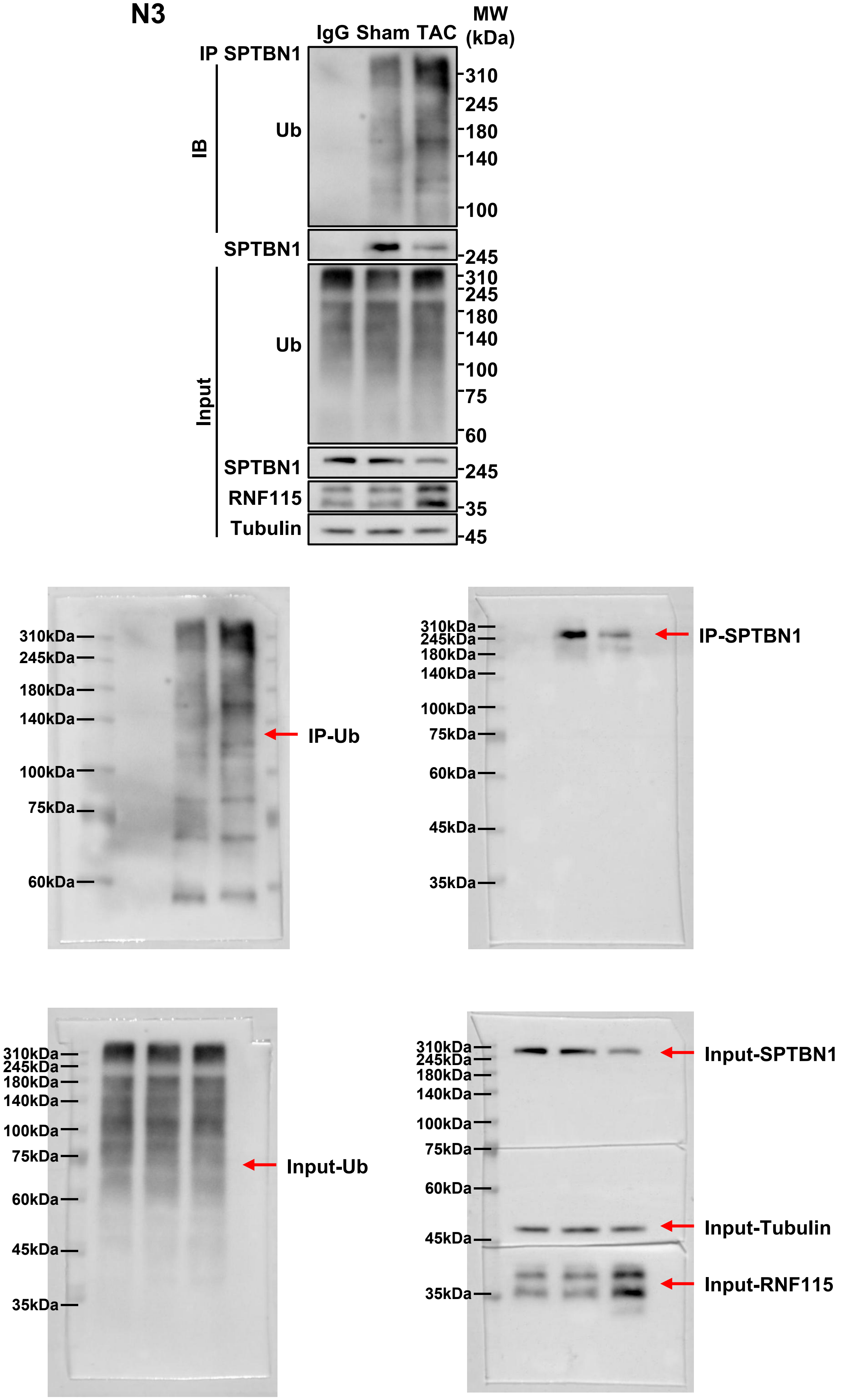
IgG Sham TAC
MW (kDa)
IP SPTBN1
IB
Ub
310
245
180
140
100
SPTBN1
245
Input
Ub
310
245
180
140
100
75
60
SPTBN1
245
RNF115
35
Tubulin
45
310kDa
245kDa
180kDa
140kDa
100kDa
75kDa
60kDa
IP-Ub
310kDa
245kDa
180kDa
140kDa
100kDa
75kDa
60kDa
45kDa
35kDa
IP-SPTBN1
310kDa
245kDa
180kDa
140kDa
100kDa
75kDa
60kDa
45kDa
35kDa
Input-Ub
310kDa
245kDa
180kDa
140kDa
100kDa
75kDa
60kDa
45kDa
35kDa
Input-SPTBN1
Input-Tubulin
Input-RNF115

Figure3I

n=3

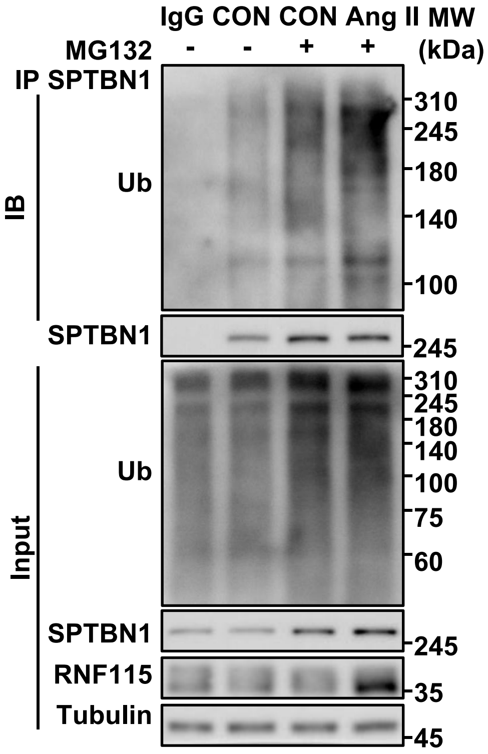
N1
IgG CON CON Ang II MW
MG132 - - + + (kDa)
IP SPTBN1
IB
Ub
310
245
180
140
100
SPTBN1
245
Input
Ub
310
245
180
140
100
75
60
SPTBN1
245
RNF115
35
Tubulin
45

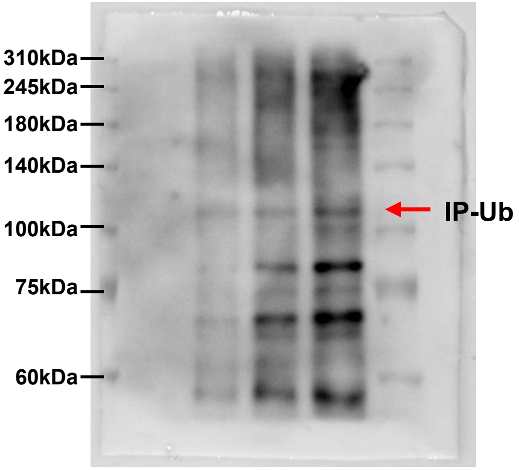
310kDa
245kDa
180kDa
140kDa
100kDa
75kDa
60kDa
IP-Ub

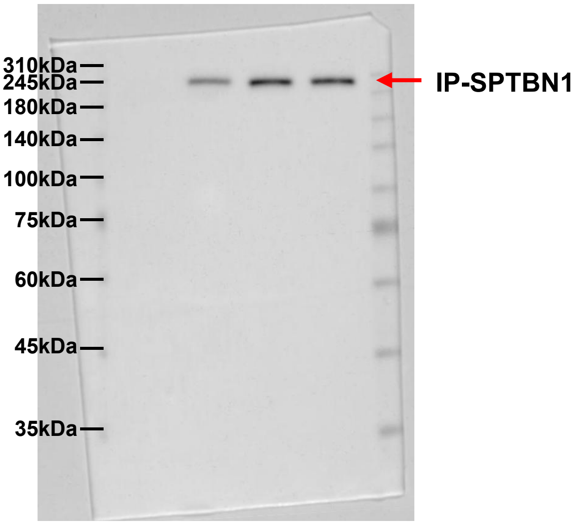
310kDa
245kDa
180kDa
140kDa
100kDa
75kDa
60kDa
45kDa
35kDa
IP-SPTBN1

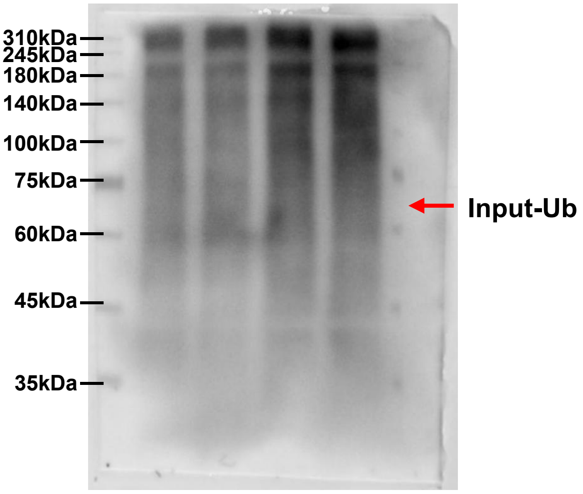
310kDa
245kDa
180kDa
140kDa
100kDa
75kDa
60kDa
45kDa
35kDa
Input-Ub

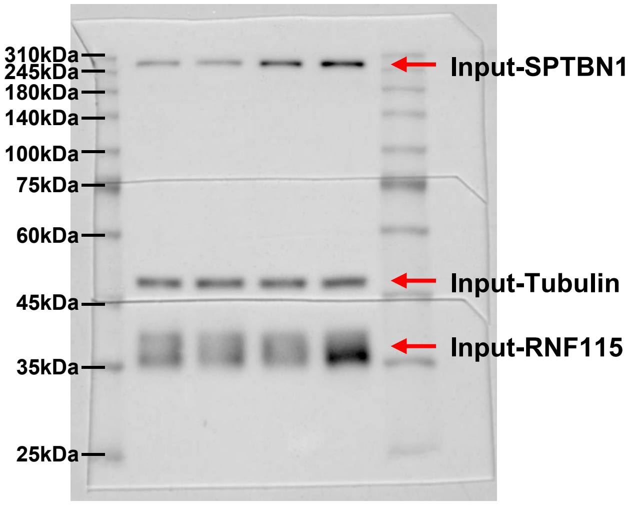
310kDa
245kDa
180kDa
140kDa
100kDa
75kDa
60kDa
45kDa
35kDa
25kDa
Input-SPTBN1
Input-Tubulin
Input-RNF115

N2

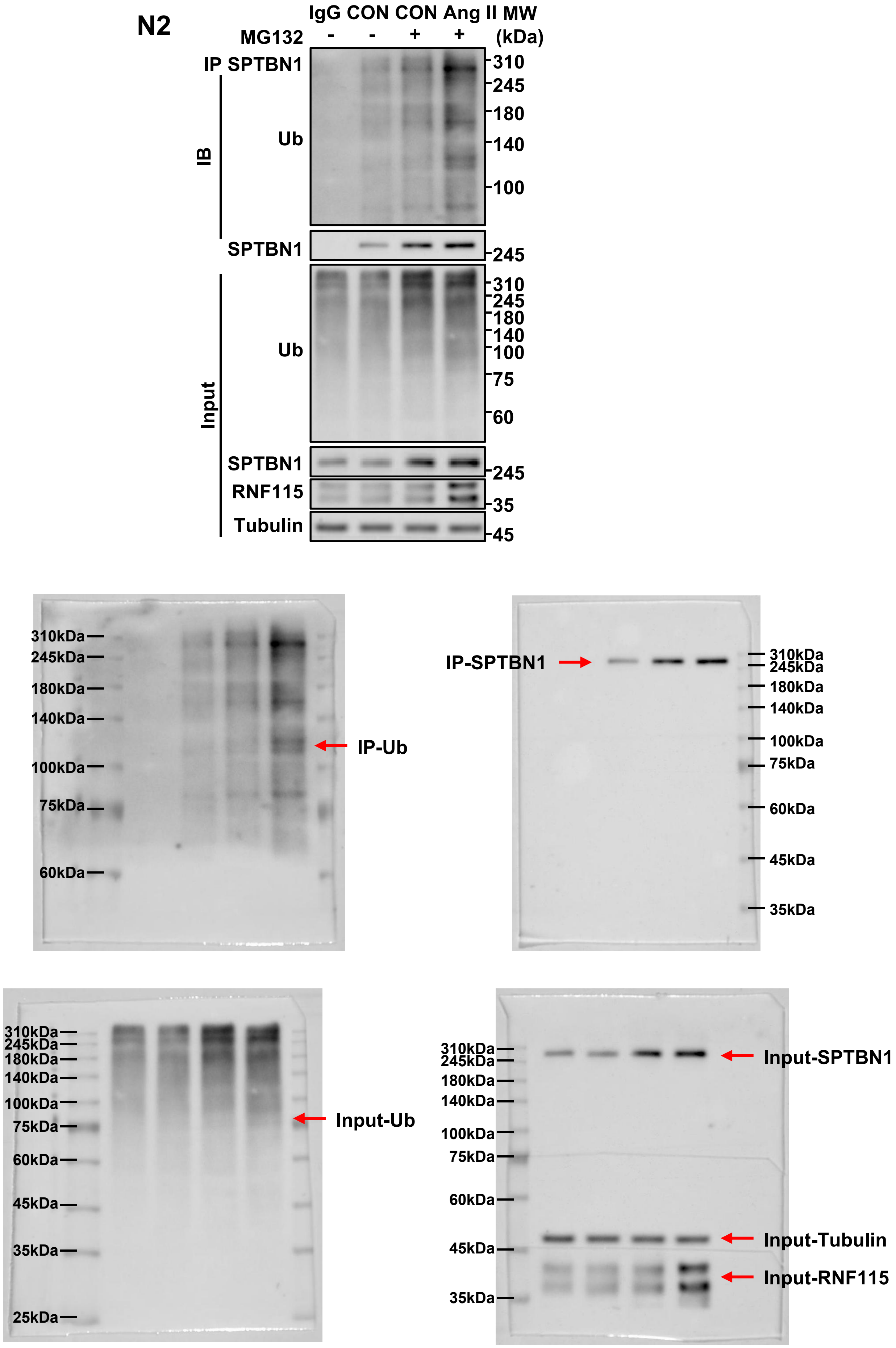
IgG CON CON Ang II MW
MG132 - - + + (kDa)
IP SPTBN1
IB
Ub
SPTBN1
Input
Ub
SPTBN1
RNF115
Tubulin
310
245
180
140
100
245
310
245
180
140
100
75
60
245
35
45
310kDa
245kDa
180kDa
140kDa
100kDa
75kDa
60kDa
IP-Ub
IP-SPTBN1
310kDa
245kDa
180kDa
140kDa
100kDa
75kDa
60kDa
45kDa
35kDa
310kDa
245kDa
180kDa
140kDa
100kDa
75kDa
60kDa
45kDa
35kDa
25kDa
Input-Ub
310kDa
245kDa
180kDa
140kDa
100kDa
75kDa
60kDa
45kDa
35kDa
Input-SPTBN1
Input-Tubulin
Input-RNF115

N3

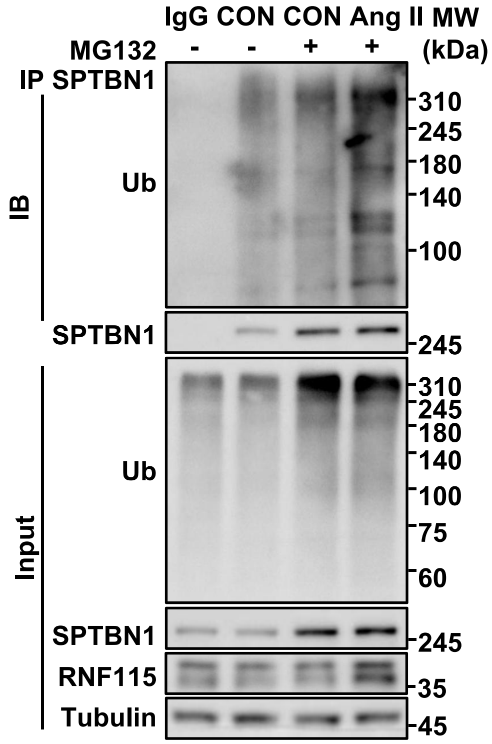
IgG CON CON Ang II MW
MG132 - - + + (kDa)
IP SPTBN1
IB
Ub
310
245
180
140
100
SPTBN1
245
Input
Ub
310
245
180
140
100
75
60
SPTBN1
245
RNF115
35
Tubulin
45

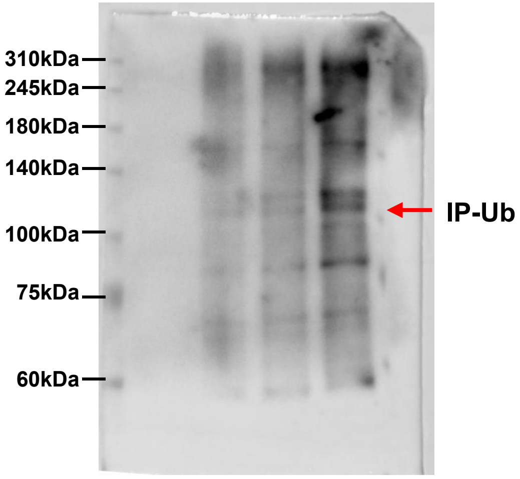
310kDa
245kDa
180kDa
140kDa
100kDa
75kDa
60kDa
IP-Ub

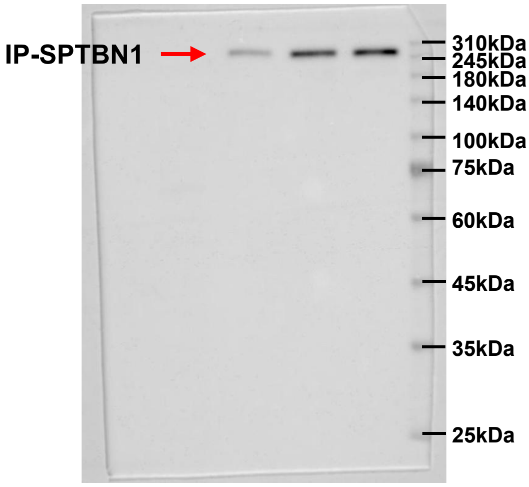
IP-SPTBN1
310kDa
245kDa
180kDa
140kDa
100kDa
75kDa
60kDa
45kDa
35kDa
25kDa

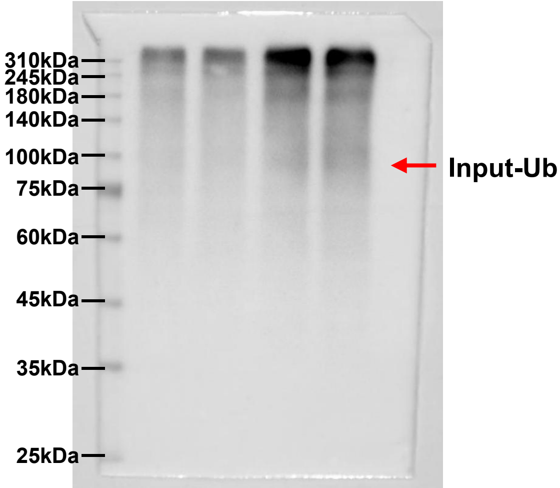
310kDa
245kDa
180kDa
140kDa
100kDa
75kDa
60kDa
45kDa
35kDa
25kDa
Input-Ub

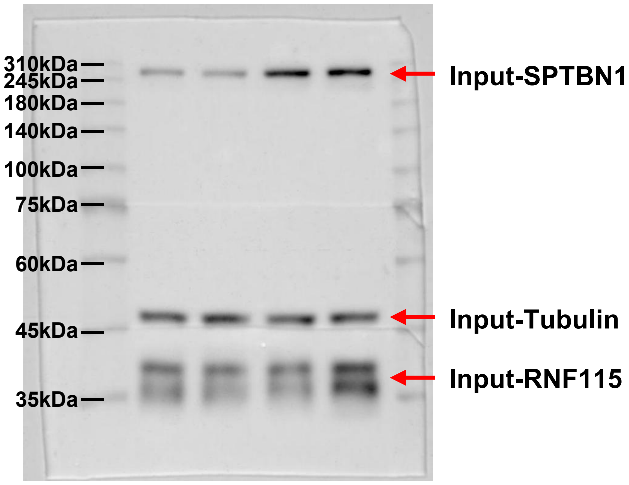
310kDa
245kDa
180kDa
140kDa
100kDa
75kDa
60kDa
45kDa
35kDa
Input-SPTBN1
Input-Tubulin
Input-RNF115

Figure4A

n=3

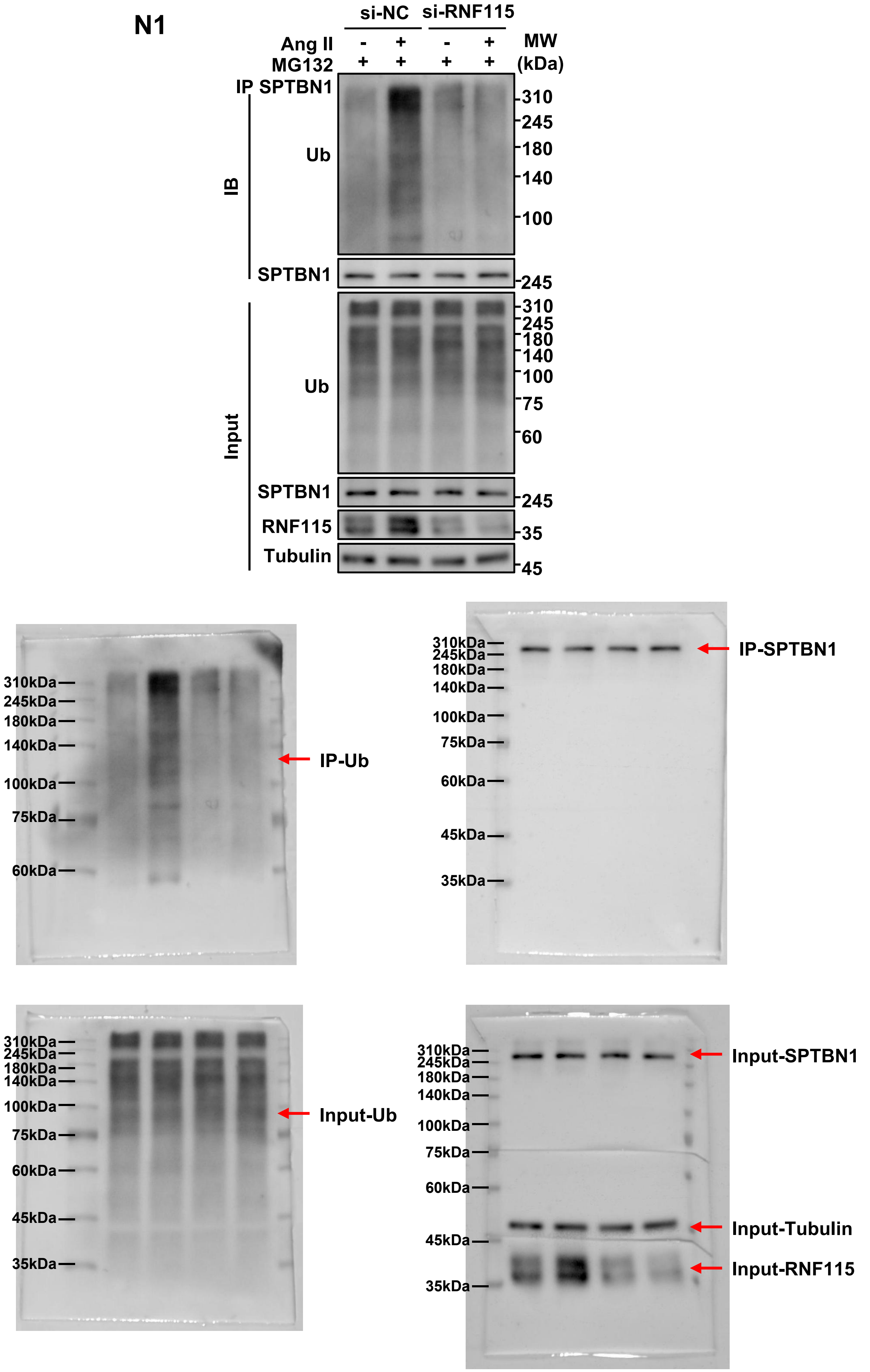

N1
si-NC
si-RNF115
Ang II
MG132
MW
(kDa)
IP SPTBN1
IB
Ub
SPTBN1
Input
Ub
SPTBN1
RNF115
Tubulin
310
245
180
140
100
75
60
35
45
IP-Ub
IP-SPTBN1
Input-Ub
Input-SPTBN1
Input-Tubulin
Input-RNF115

N2

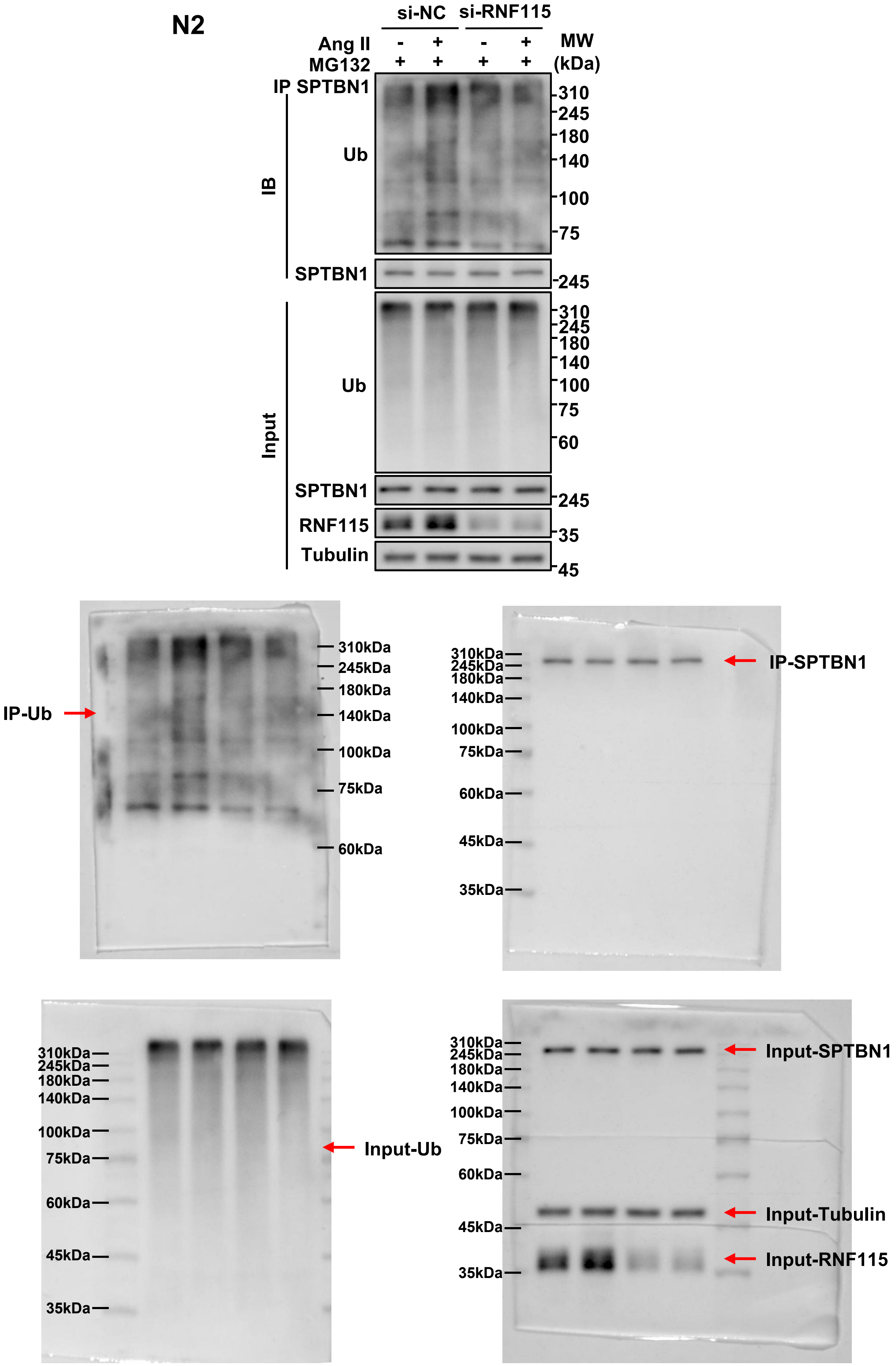
si-NC
si-RNF115
Ang II
MG132
MW
(kDa)
IP SPTBN1
IB
Ub
SPTBN1
Input
Ub
SPTBN1
RNF115
Tubulin
310
245
180
140
100
75
60
35
45
IP-Ub
IP-SPTBN1
Input-Ub
Input-SPTBN1
Input-Tubulin
Input-RNF115
310kDa
245kDa
180kDa
140kDa
100kDa
75kDa
60kDa
45kDa
35kDa

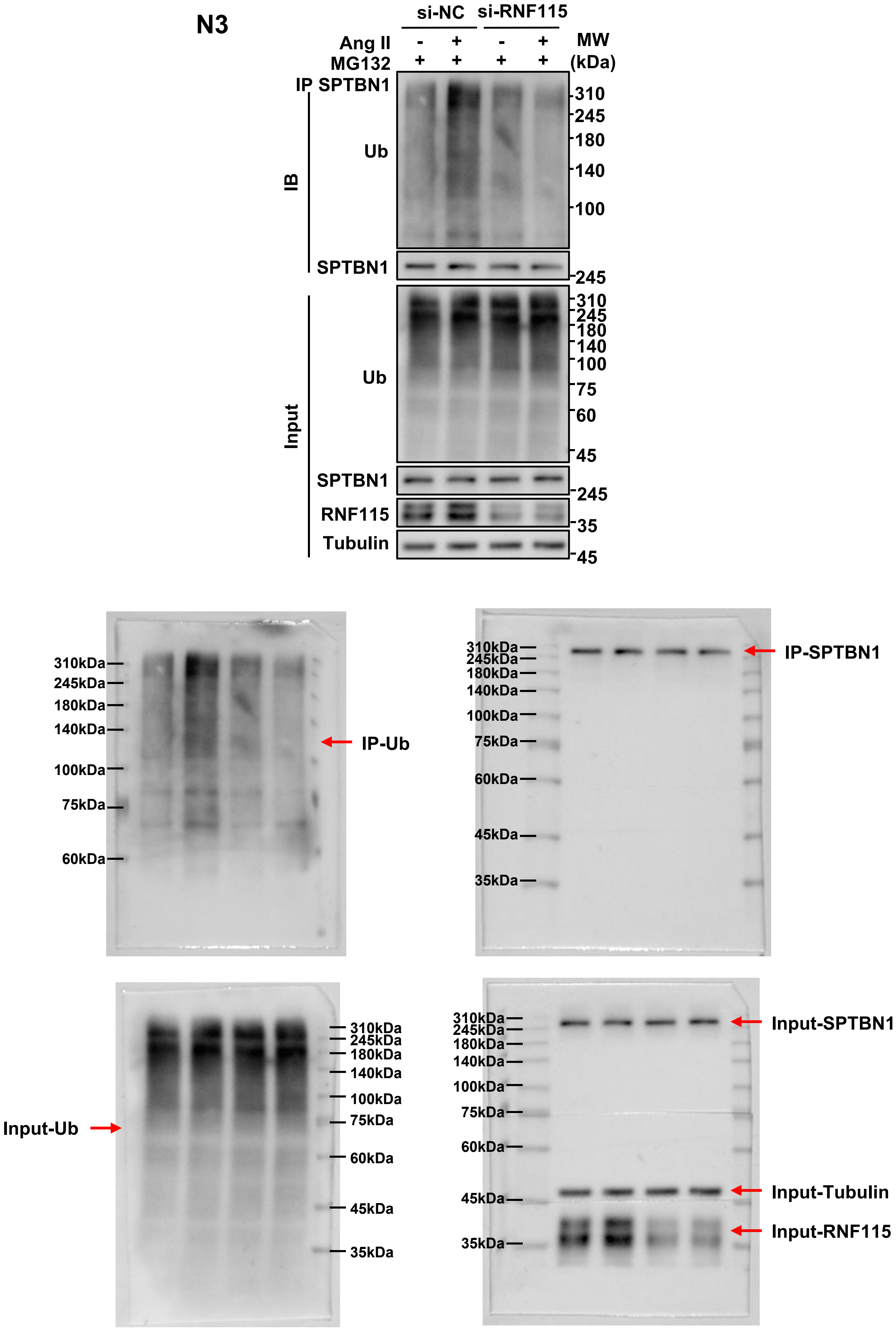
N3
si-NC
si-RNF115
Ang II - + - +
MG132 + + + +
MW
(kDa)
IP SPTBN1
IB
Ub
SPTBN1
Input
Ub
SPTBN1
RNF115
Tubulin
310
245
180
140
100
75
60
45
35
IP-Ub
IP-SPTBN1
Input-Ub
Input-SPTBN1
Input-Tubulin
Input-RNF115
310kDa
245kDa
180kDa
140kDa
100kDa
75kDa
60kDa
45kDa
35kDa

Figure4B

n=3

N1

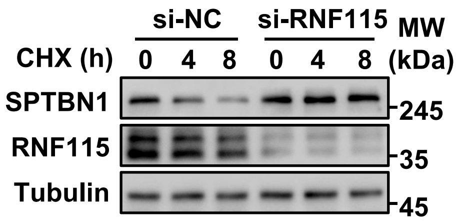

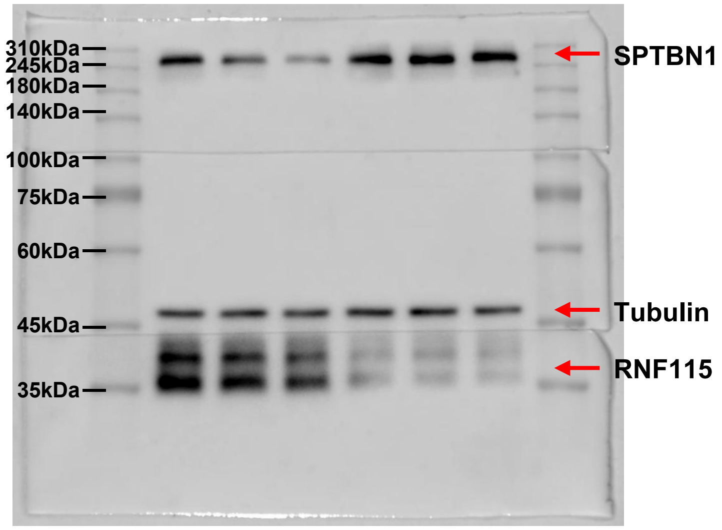

N2

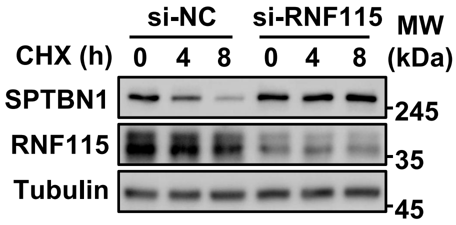

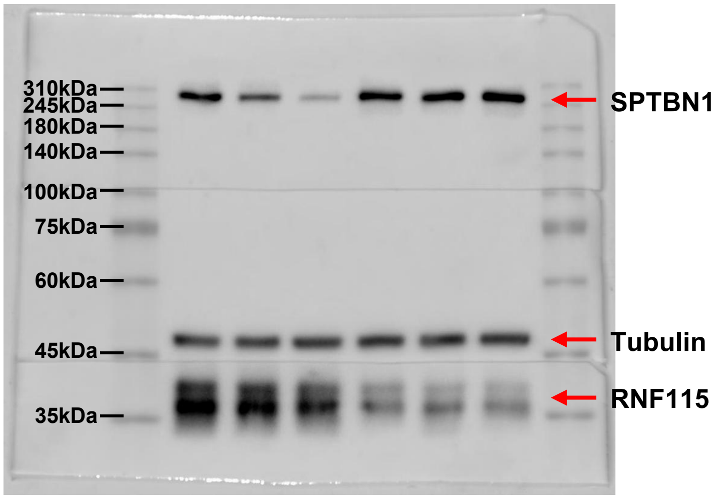

N3

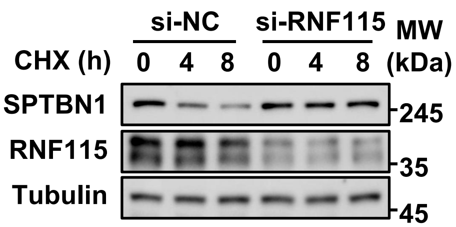
si-NC
si-RNF115
MW
(kDa)
CHX (h)
0
4
8
0
4
8
SPTBN1
245
RNF115
35
Tubulin
45

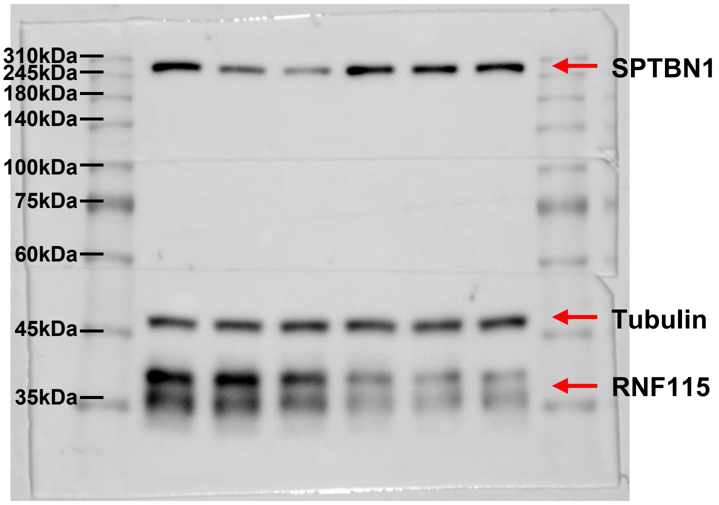
310kDa
245kDa
180kDa
140kDa
100kDa
75kDa
60kDa
45kDa
35kDa
SPTBN1
Tubulin
RNF115

Figure4C

n=3

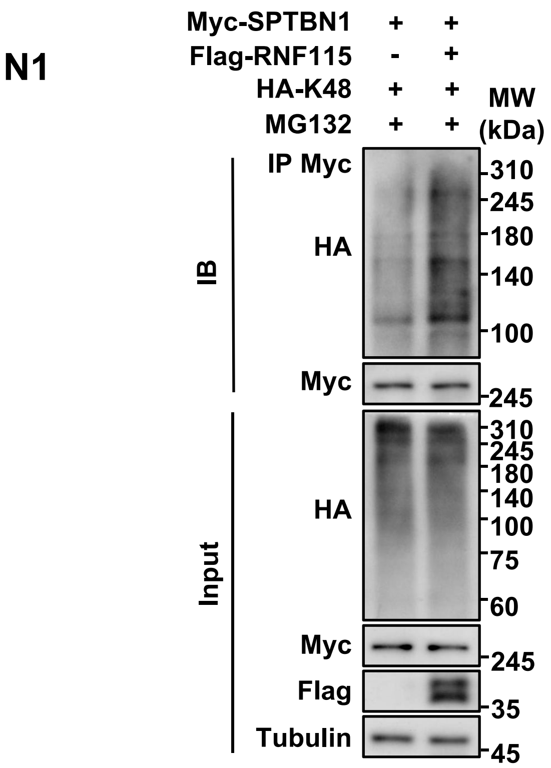
N1
Myc-SPTBN1 + +
Flag-RNF115 - +
HA-K48 + +
MG132 + +
MW (kDa)
IB
IP Myc
HA
310
245
180
140
100
Myc
245
Input
HA
310
245
180
140
100
75
60
Myc
245
Flag
35
Tubulin
45

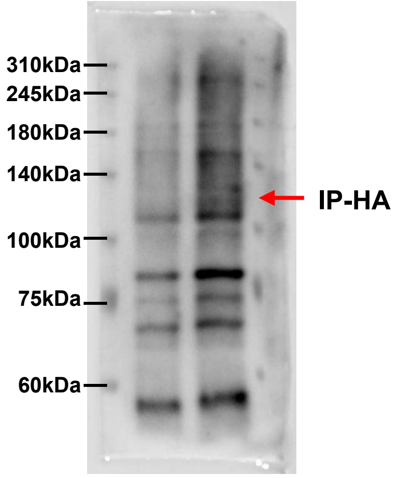
310kDa
245kDa
180kDa
140kDa
100kDa
75kDa
60kDa
IP-HA

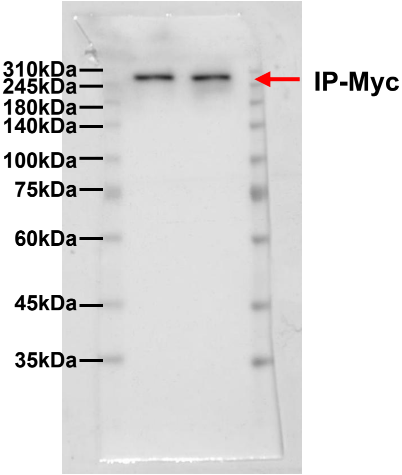
310kDa
245kDa
180kDa
140kDa
100kDa
75kDa
60kDa
45kDa
35kDa
IP-Myc

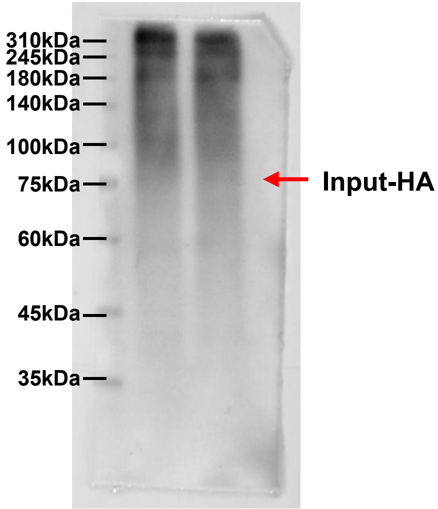
310kDa
245kDa
180kDa
140kDa
100kDa
75kDa
60kDa
45kDa
35kDa
Input-HA

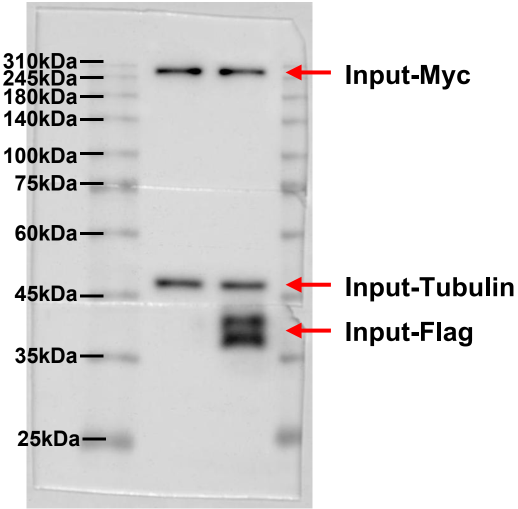
310kDa
245kDa
180kDa
140kDa
100kDa
75kDa
60kDa
45kDa
35kDa
25kDa
Input-Myc
Input-Tubulin
Input-Flag

N2

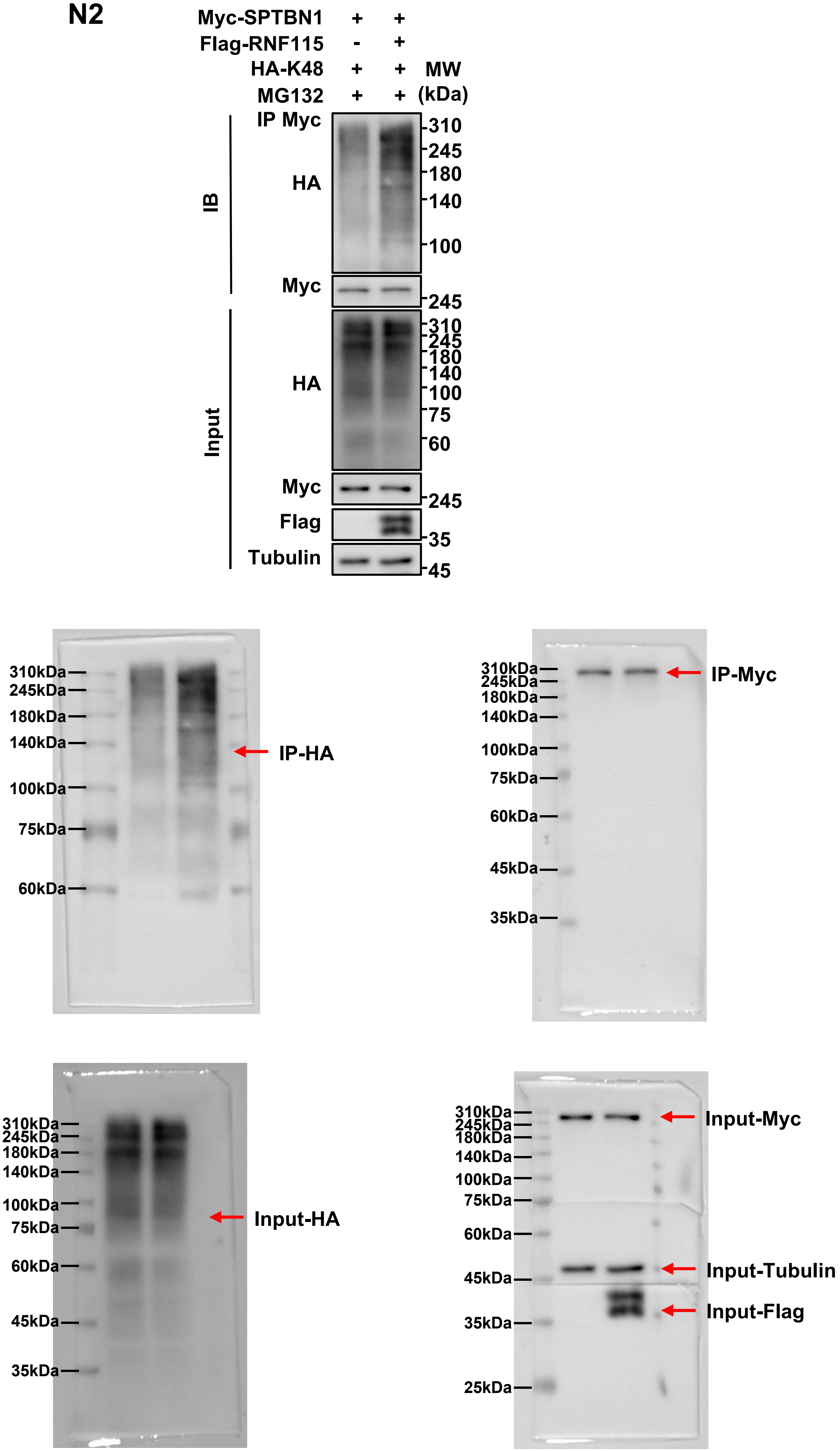

Myc-SPTBN1 + +
Flag-RNF115 - +
HA-K48 + +
MG132 + +
MW (kDa)
IB
IP Myc
HA
Myc
Input
HA
Myc
Flag
Tubulin
310
245
180
140
100
245
310
245
180
140
100
75
60
245
35
45
310kDa
245kDa
180kDa
140kDa
100kDa
75kDa
60kDa
IP-HA
310kDa
245kDa
180kDa
140kDa
100kDa
75kDa
60kDa
45kDa
35kDa
IP-Myc
310kDa
245kDa
180kDa
140kDa
100kDa
75kDa
60kDa
45kDa
35kDa
Input-HA
310kDa
245kDa
180kDa
140kDa
100kDa
75kDa
60kDa
45kDa
35kDa
25kDa
Input-Myc
Input-Tubulin
Input-Flag

N3

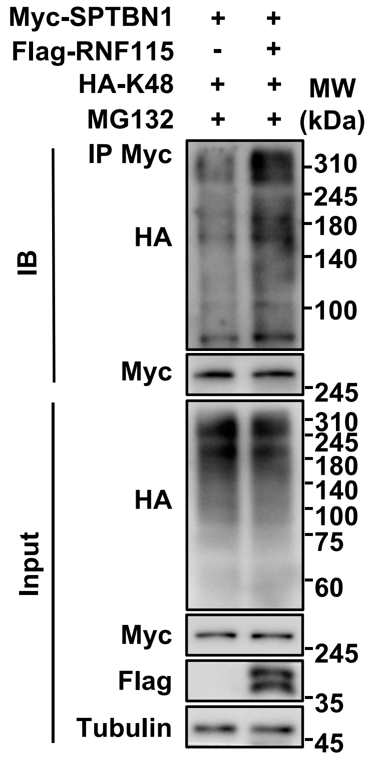

Myc-SPTBN1 + +
Flag-RNF115 - +
HA-K48 + +
MG132 + +
MW (kDa)
IB
IP Myc
HA
310
245
180
140
100
Myc
245
Input
HA
310
245
180
140
100
75
60
Myc
245
Flag
35
Tubulin
45

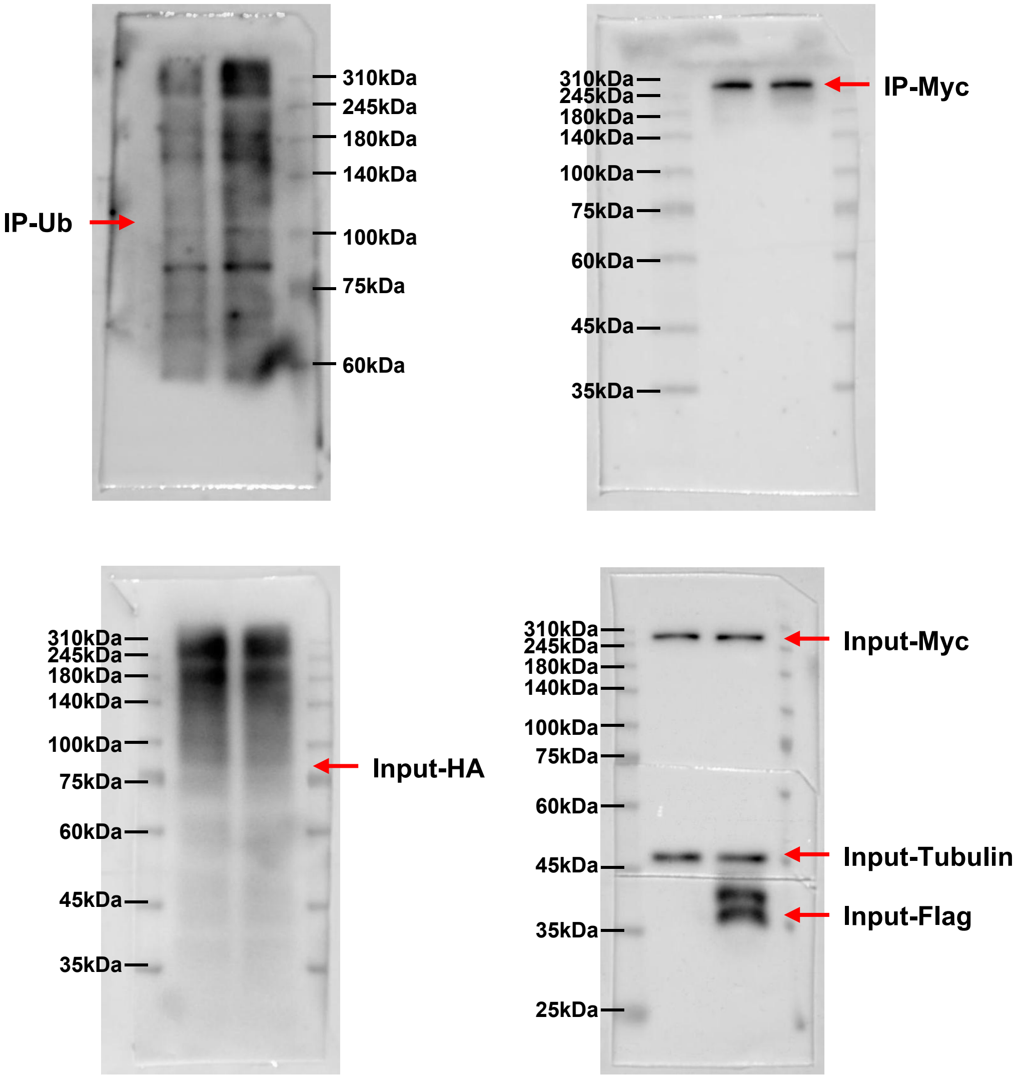

310kDa
245kDa
180kDa
140kDa
IP-Ub
100kDa
75kDa
60kDa
310kDa
245kDa
180kDa
140kDa
100kDa
75kDa
60kDa
45kDa
35kDa
IP-Myc
310kDa
245kDa
180kDa
140kDa
100kDa
75kDa
60kDa
45kDa
35kDa
Input-HA
310kDa
245kDa
180kDa
140kDa
100kDa
75kDa
60kDa
45kDa
35kDa
25kDa
Input-Myc
Input-Tubulin
Input-Flag

Figure4D

n=3

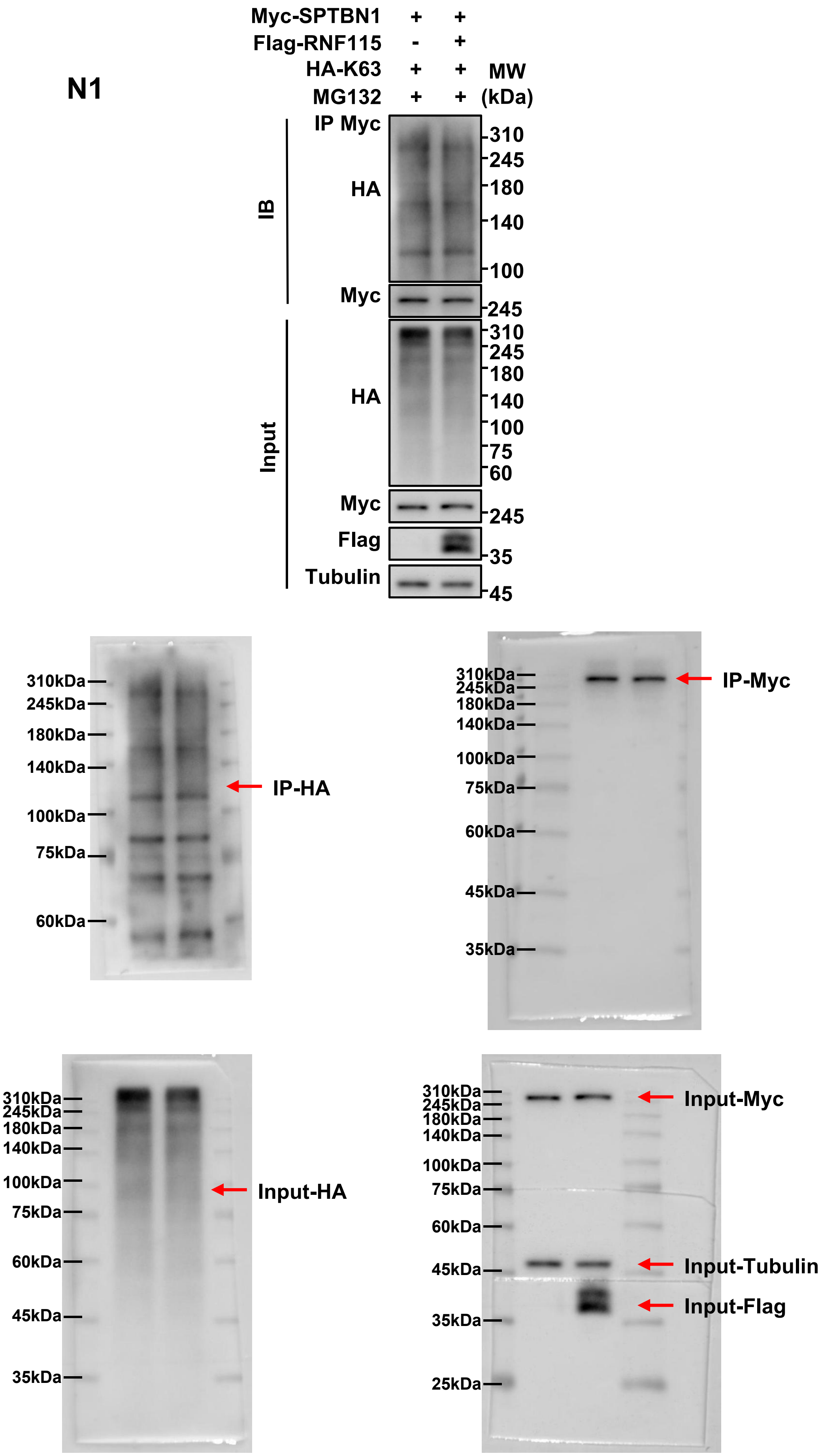

N1
Myc-SPTBN1 + +
Flag-RNF115 - +
HA-K63 + +
MG132 + +
MW (kDa)
IB
IP Myc
HA
310
245
180
140
100
Myc
245
Input
HA
310
245
180
140
100
75
60
Myc
245
Flag
35
Tubulin
45
310kDa
245kDa
180kDa
140kDa
100kDa
75kDa
60kDa
IP-HA
310kDa
245kDa
180kDa
140kDa
100kDa
75kDa
60kDa
45kDa
35kDa
IP-Myc
310kDa
245kDa
180kDa
140kDa
100kDa
75kDa
60kDa
45kDa
35kDa
Input-HA
310kDa
245kDa
180kDa
140kDa
100kDa
75kDa
60kDa
45kDa
35kDa
25kDa
Input-Myc
Input-Tubulin
Input-Flag

N2

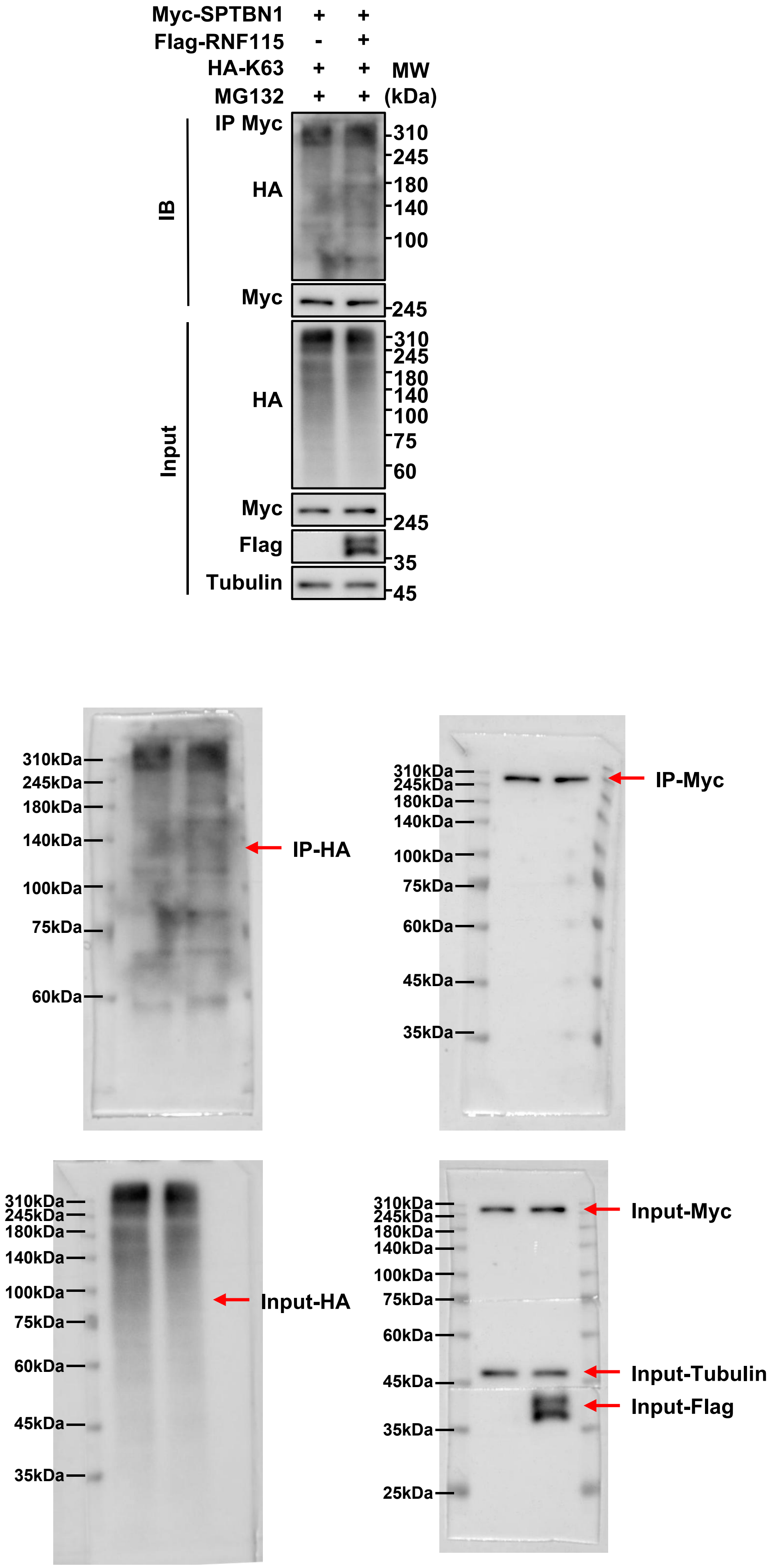
Myc-SPTBN1 + +
Flag-RNF115 - +
HA-K63 + +
MG132 + +
MW (kDa)
IP Myc
IB
HA
310
245
180
140
100
Myc
245
Input
HA
310
245
180
140
100
75
60
Myc
245
Flag
35
Tubulin
45
310kDa
245kDa
180kDa
140kDa
100kDa
75kDa
60kDa
IP-HA
310kDa
245kDa
180kDa
140kDa
100kDa
75kDa
60kDa
45kDa
35kDa
IP-Myc
310kDa
245kDa
180kDa
140kDa
100kDa
75kDa
60kDa
45kDa
35kDa
Input-HA
310kDa
245kDa
180kDa
140kDa
100kDa
75kDa
60kDa
45kDa
35kDa
25kDa
Input-Myc
Input-Tubulin
Input-Flag

N3

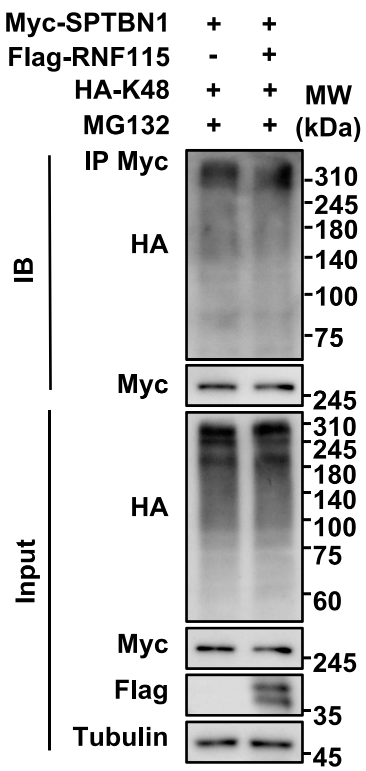
Myc-SPTBN1 + +
Flag-RNF115 - +
HA-K48 + +
MG132 + +
MW (kDa)
IB
IP Myc
HA
310
245
180
140
100
75
Myc
245
Input
HA
310
245
180
140
100
75
60
Myc
245
Flag
35
Tubulin
45

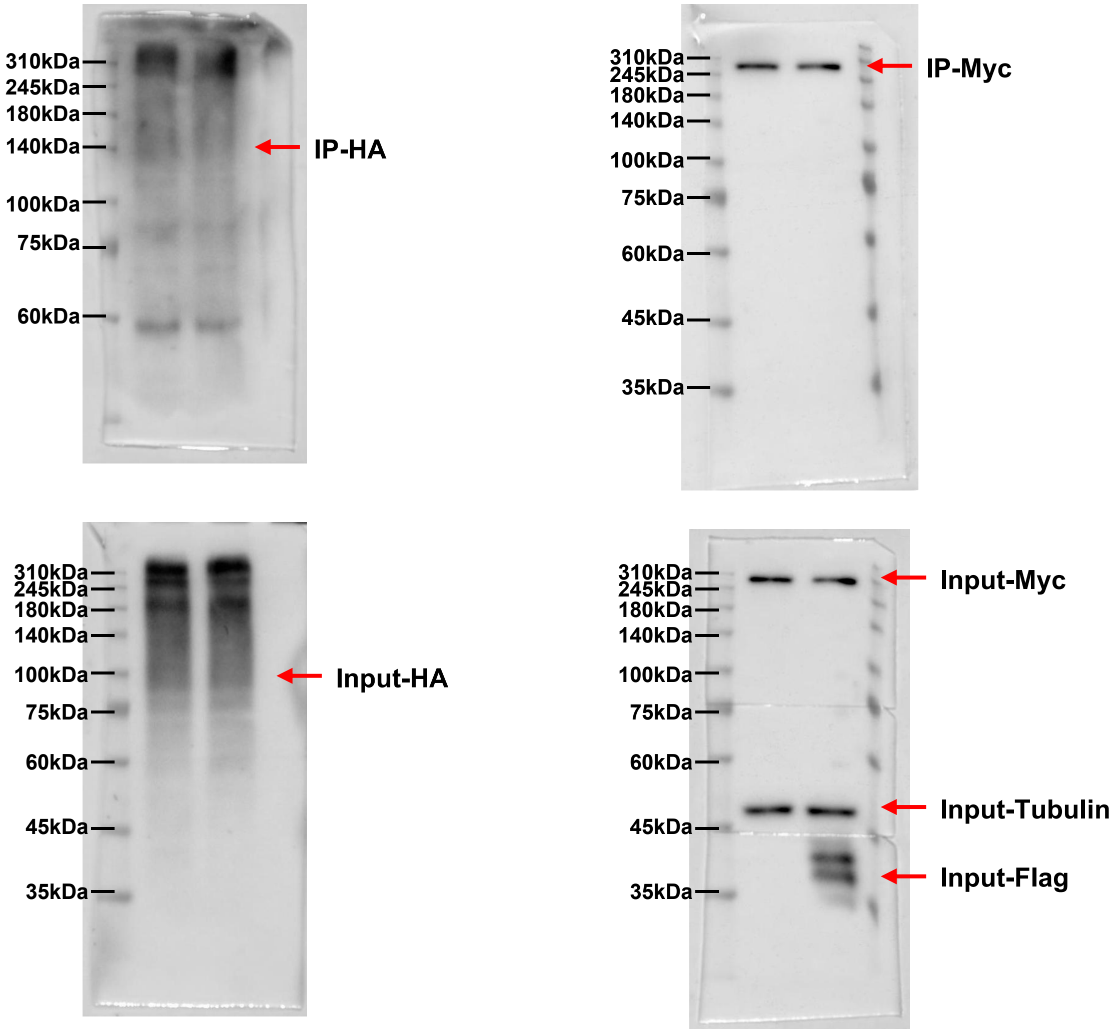
310kDa
245kDa
180kDa
140kDa
100kDa
75kDa
60kDa
IP-HA
310kDa
245kDa
180kDa
140kDa
100kDa
75kDa
60kDa
45kDa
35kDa
IP-Myc
310kDa
245kDa
180kDa
140kDa
100kDa
75kDa
60kDa
45kDa
35kDa
Input-HA
310kDa
245kDa
180kDa
140kDa
100kDa
75kDa
60kDa
45kDa
35kDa
Input-Myc
Input-Tubulin
Input-Flag

Figure4F

n=3

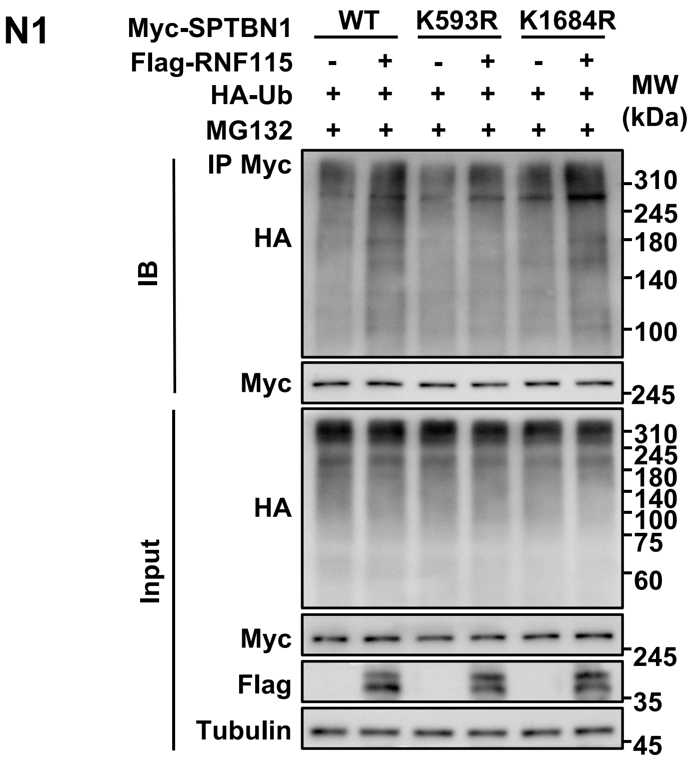
N1
Myc-SPTBN1
WT
K593R
K1684R
Flag-RNF115
HA-Ub
MG132
MW
(kDa)
IP Myc
IB
HA
Myc
Input
HA
Myc
Flag
Tubulin
310
245
180
140
100
75
60
35
45

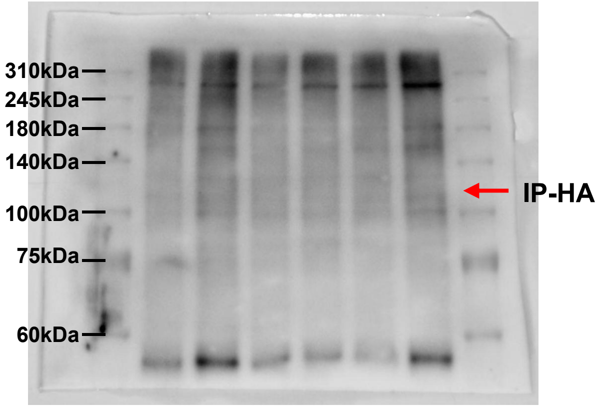
310kDa
245kDa
180kDa
140kDa
100kDa
75kDa
60kDa
IP-HA

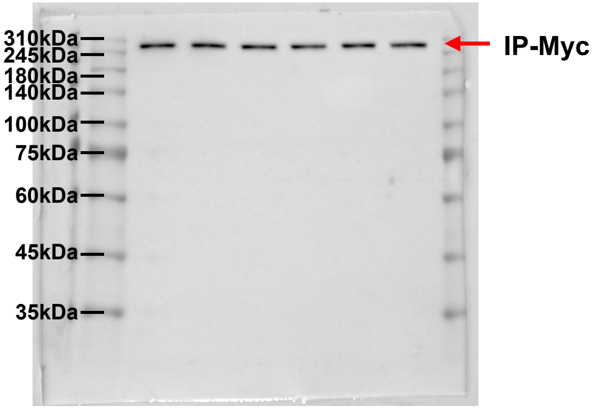
310kDa
245kDa
180kDa
140kDa
100kDa
75kDa
60kDa
45kDa
35kDa
IP-Myc

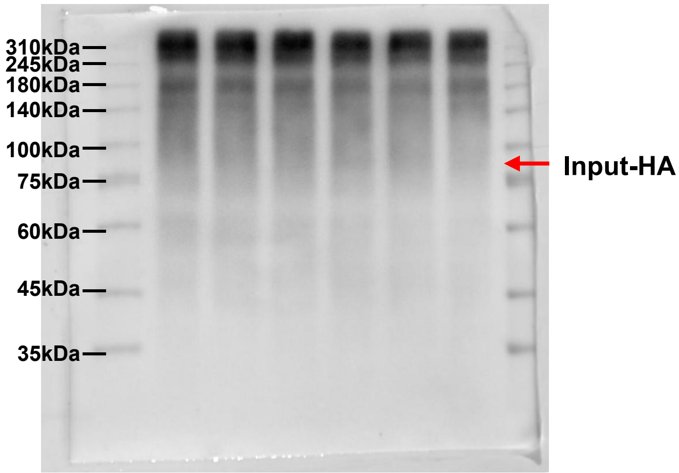
310kDa
245kDa
180kDa
140kDa
100kDa
75kDa
60kDa
45kDa
35kDa
Input-HA

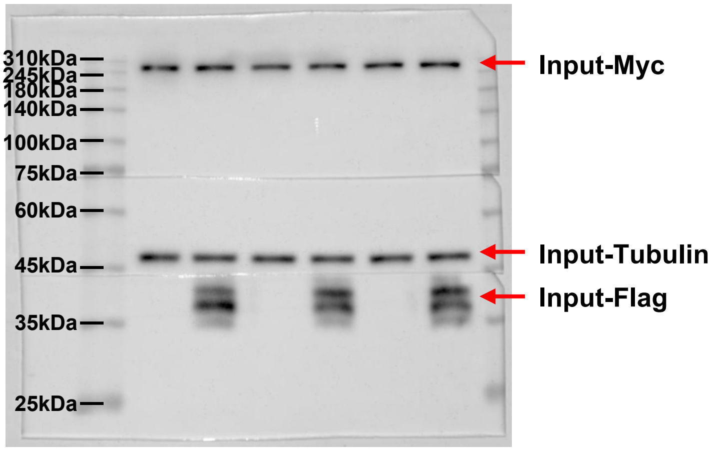
310kDa
245kDa
180kDa
140kDa
100kDa
75kDa
60kDa
45kDa
35kDa
25kDa
Input-Myc
Input-Tubulin
Input-Flag

N2

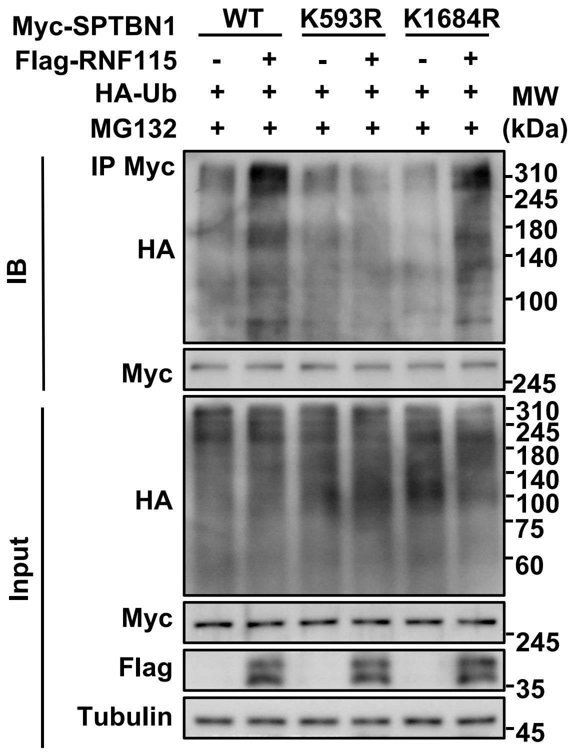
Myc-SPTBN1
WT
K593R
K1684R
Flag-RNF115
-
+
-
+
-
+
HA-Ub
+
+
+
+
+
+
MG132
+
+
+
+
+
+
MW
(kDa)
IB
IP Myc
HA
310
245
180
140
100
Myc
245
Input
HA
310
245
180
140
100
75
60
Myc
245
Flag
35
Tubulin
45

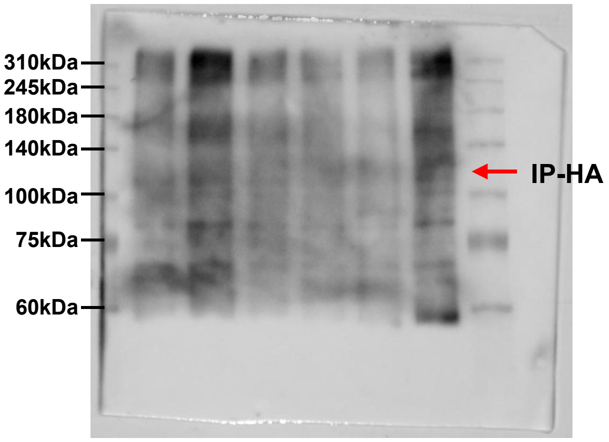
310kDa
245kDa
180kDa
140kDa
100kDa
75kDa
60kDa
IP-HA

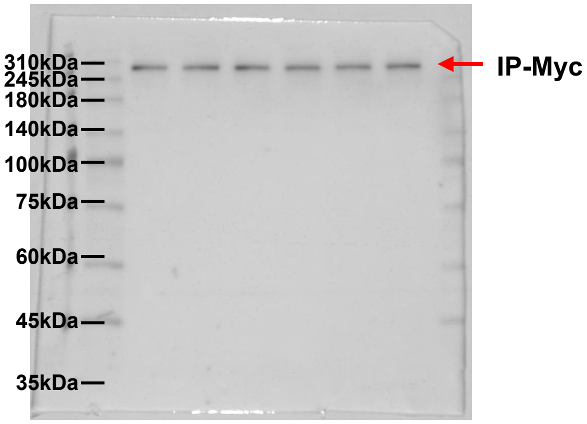
310kDa
245kDa
180kDa
140kDa
100kDa
75kDa
60kDa
45kDa
35kDa
IP-Myc

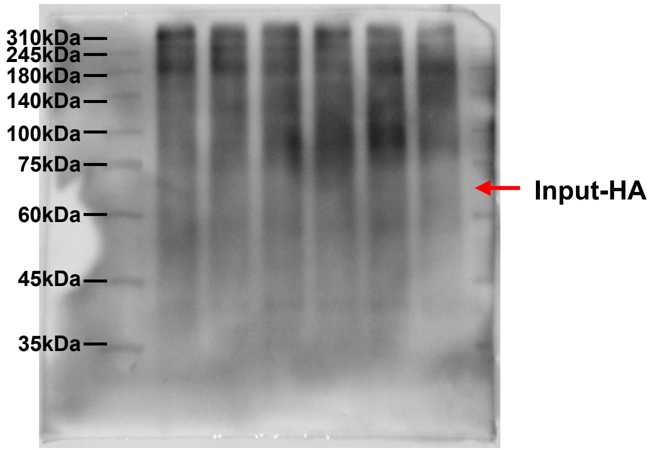
310kDa
245kDa
180kDa
140kDa
100kDa
75kDa
60kDa
45kDa
35kDa
Input-HA

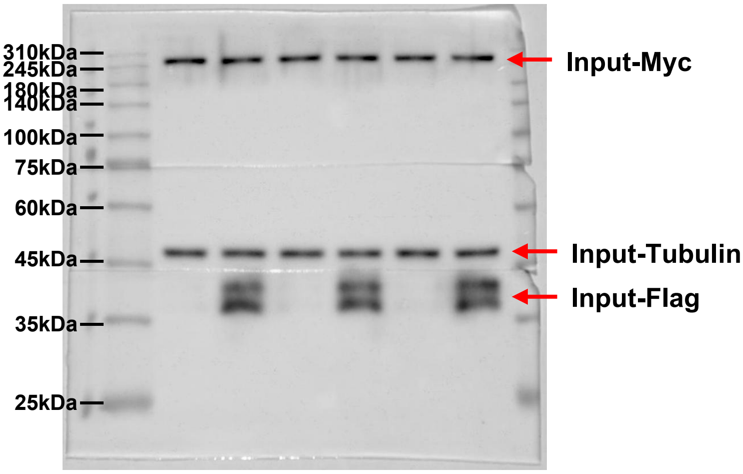
310kDa
245kDa
180kDa
140kDa
100kDa
75kDa
60kDa
45kDa
35kDa
25kDa
Input-Myc
Input-Tubulin
Input-Flag

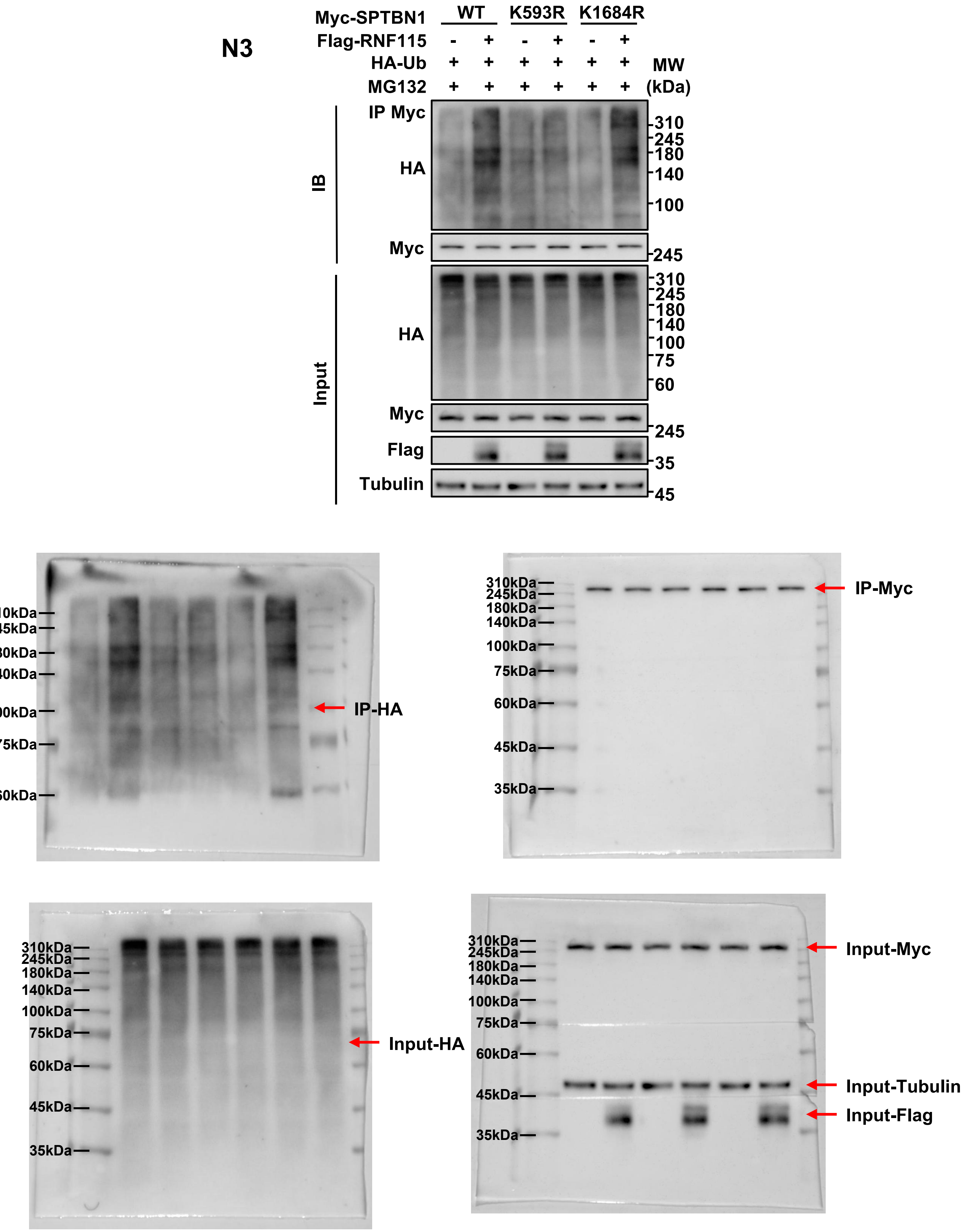
N3
Myc-SPTBN1
WT
K593R
K1684R
Flag-RNF115
HA-Ub
MG132
MW (kDa)
IB
IP Myc
HA
Myc
Input
HA
Myc
Flag
Tubulin
310
245
180
140
100
75
60
35
45
IP-HA
IP-Myc
Input-HA
Input-Myc
Input-Tubulin
Input-Flag
310kDa
245kDa
180kDa
140kDa
100kDa
75kDa
60kDa
45kDa
35kDa

Figure5A

n=3

N1

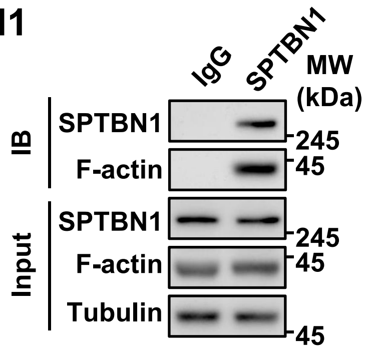

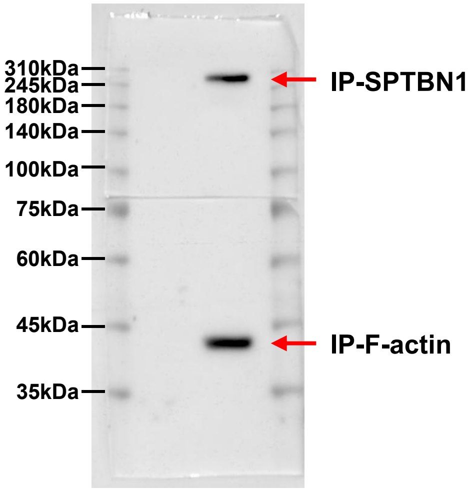

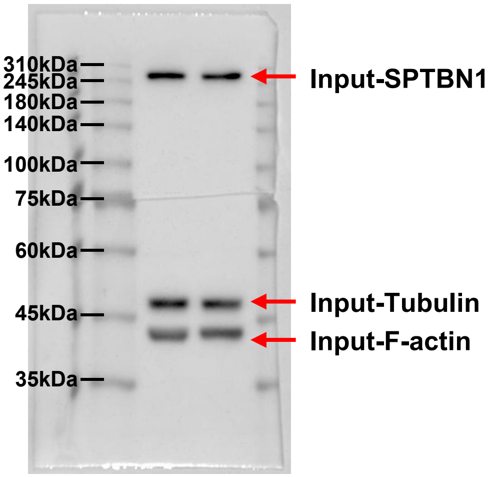

N2

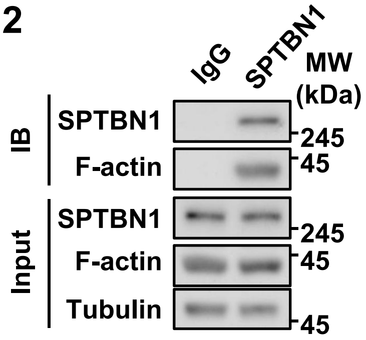

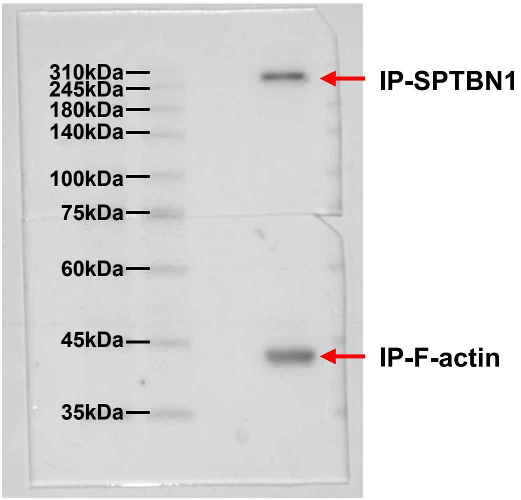

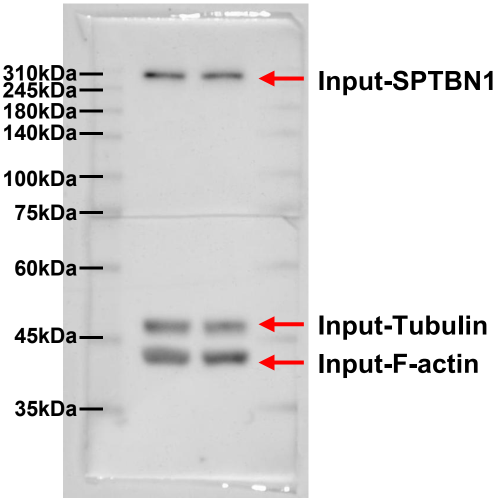

N3

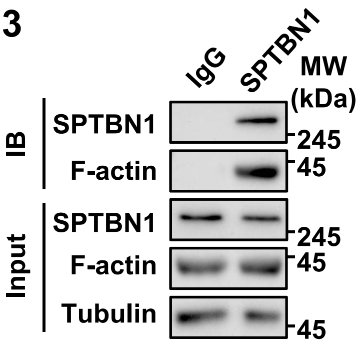

IgG
SPTBN1
MW
(kDa)
IB
SPTBN1
245
F-actin
45
Input
SPTBN1
245
F-actin
45
Tubulin
45

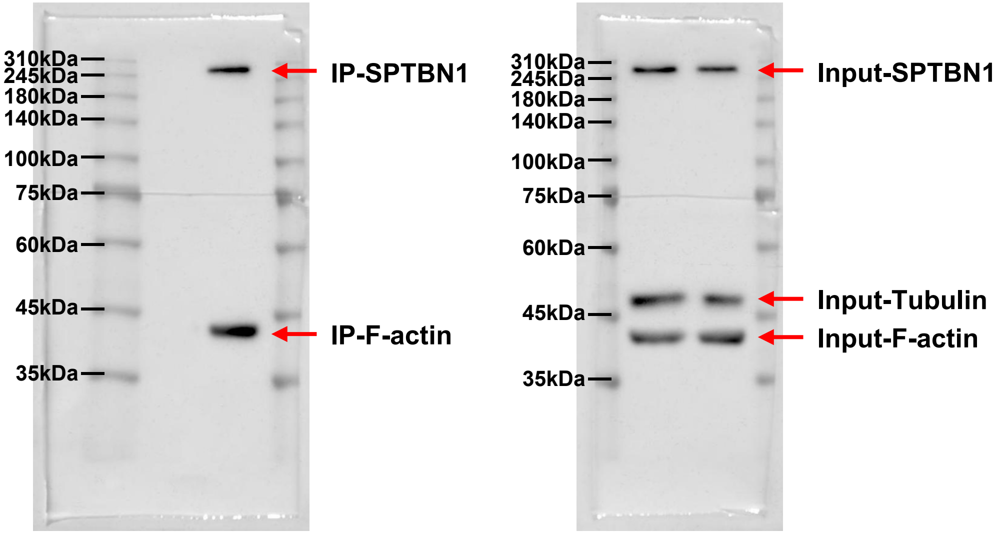

310kDa
245kDa
180kDa
140kDa
100kDa
75kDa
60kDa
45kDa
35kDa
IP-SPTBN1
IP-F-actin
310kDa
245kDa
180kDa
140kDa
100kDa
75kDa
60kDa
45kDa
35kDa
Input-SPTBN1
Input-Tubulin
Input-F-actin

Figure5B

n=3

N1

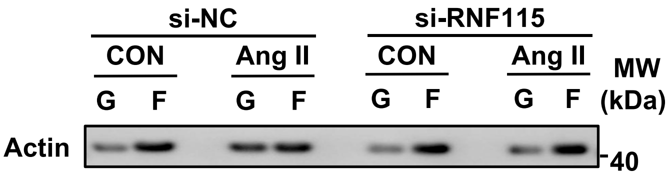

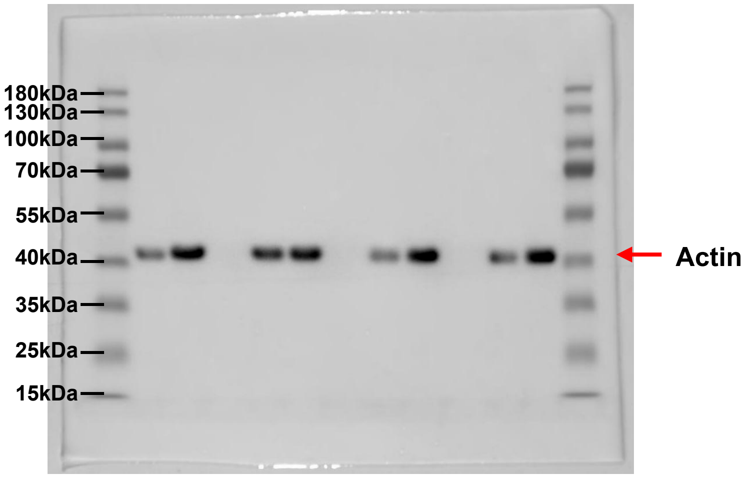

N2

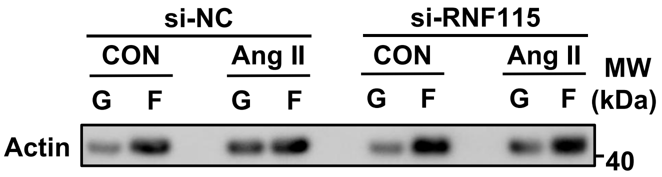

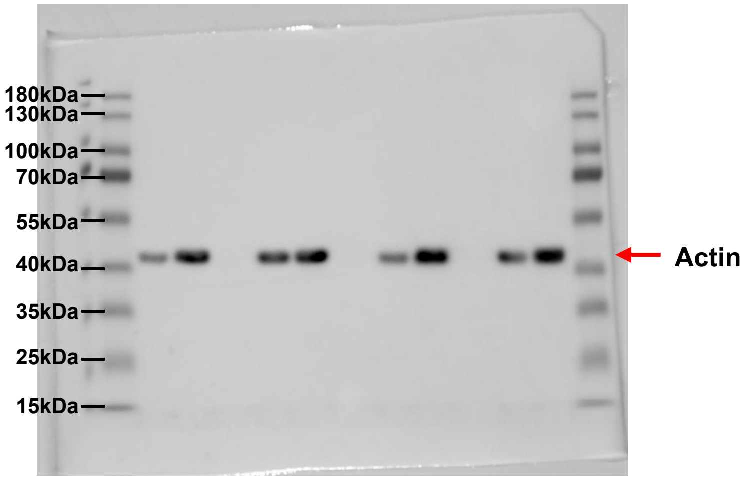

N3

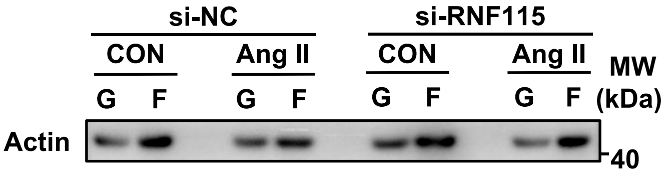
si-NC
si-RNF115
CON
Ang II
CON
Ang II
MW
(kDa)
G
F
G
F
G
F
G
F
Actin
40

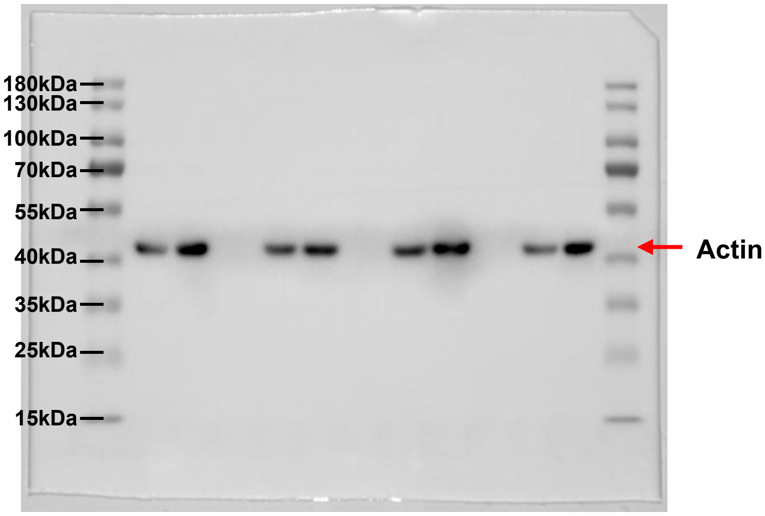
180kDa
130kDa
100kDa
70kDa
55kDa
40kDa
35kDa
25kDa
15kDa
Actin

Figure5D

n=3

N1

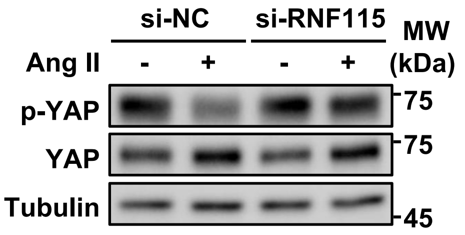

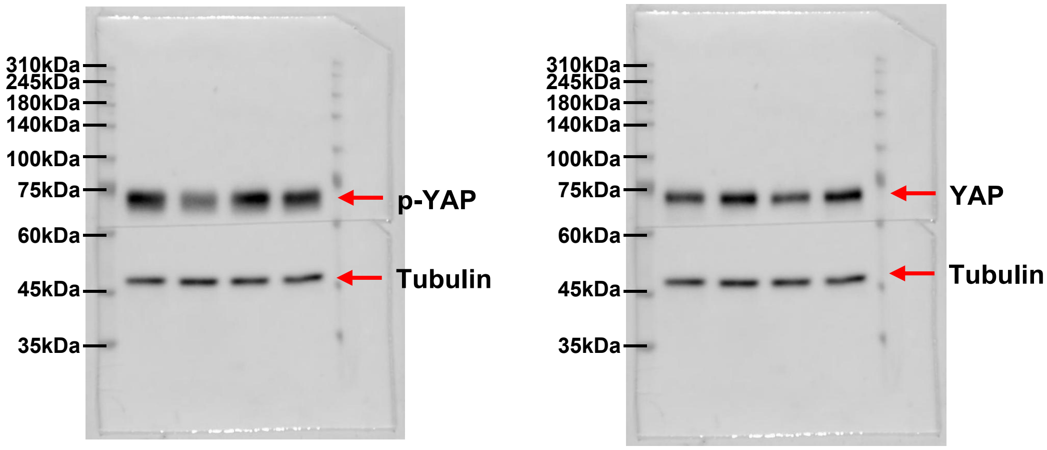

N2

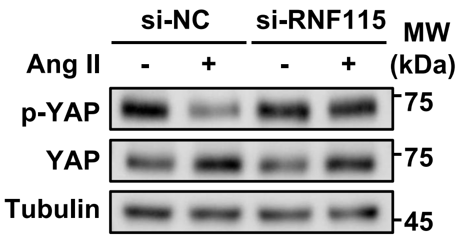

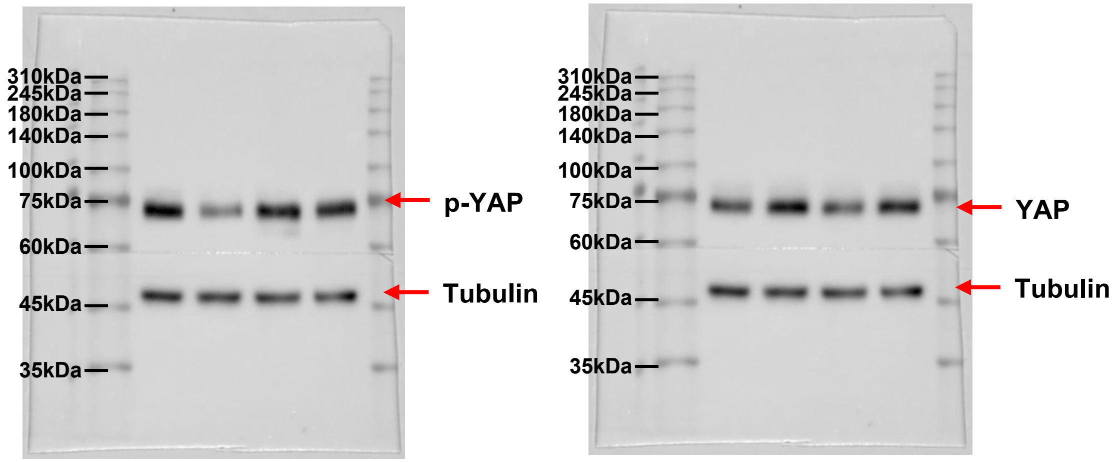

N3

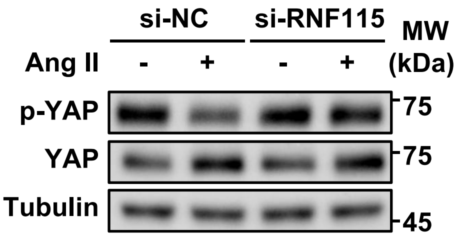
si-NC
si-RNF115
MW
(kDa)
Ang II
-
+
-
+
p-YAP
YAP
Tubulin
75
75
45

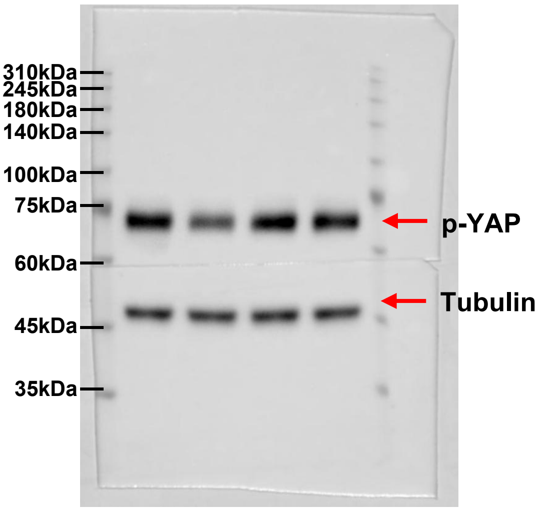
310kDa
245kDa
180kDa
140kDa
100kDa
75kDa
60kDa
45kDa
35kDa
p-YAP
Tubulin

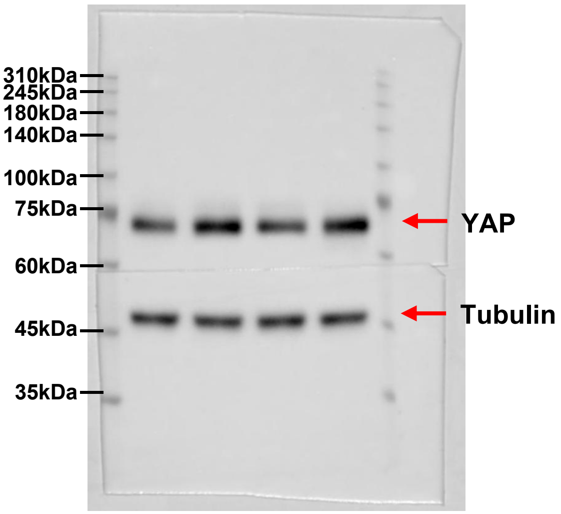
310kDa
245kDa
180kDa
140kDa
100kDa
75kDa
60kDa
45kDa
35kDa
YAP
Tubulin

Figure5E

n=3

N1

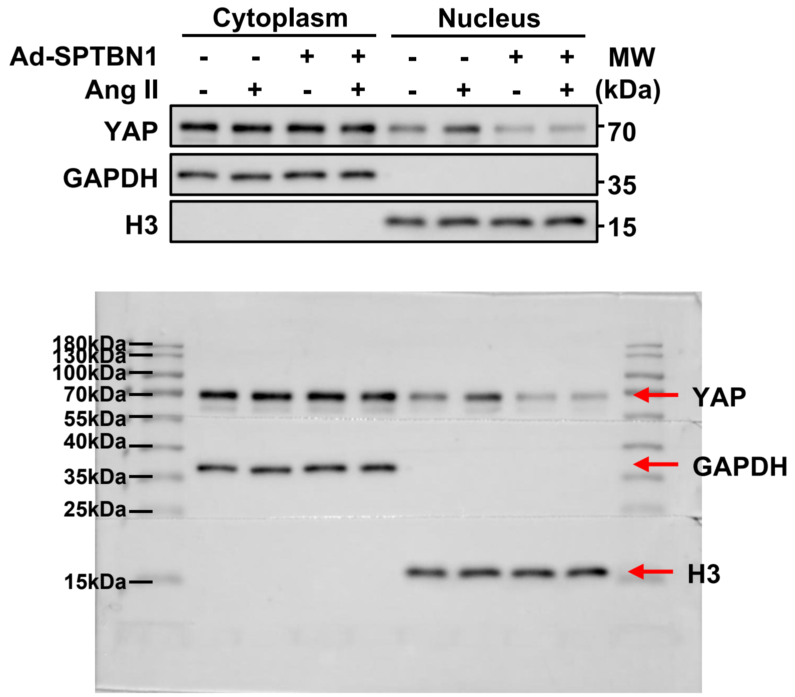

N2

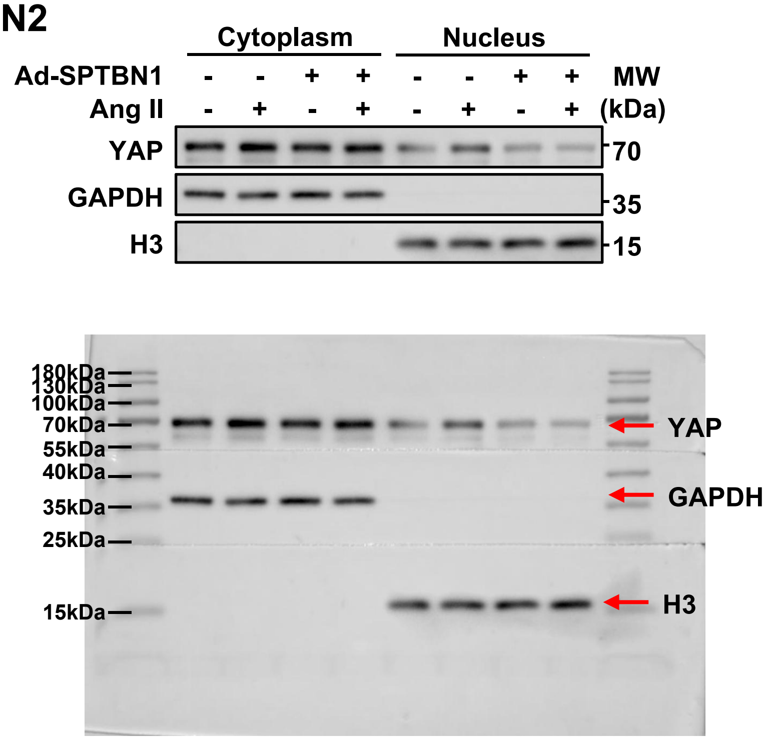

N3

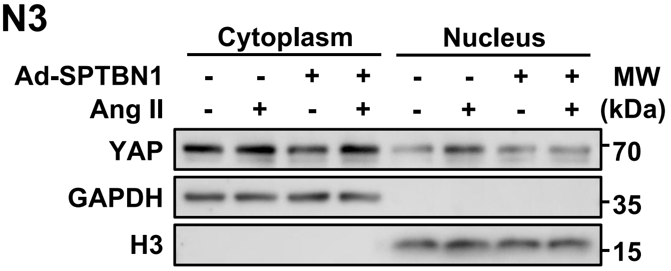
Cytoplasm
Nucleus
Ad-SPTBN1 - - + + - - + +
Ang II - + - + - + - +
MW
(kDa)
YAP
GAPDH
H3
70
35
15

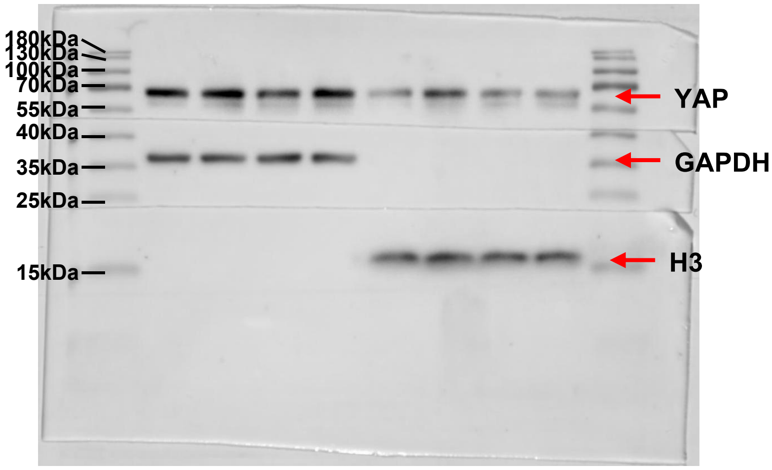
180kDa
130kDa
100kDa
70kDa
55kDa
40kDa
35kDa
25kDa
15kDa
YAP
GAPDH
H3

Figure5F

n=3

N1

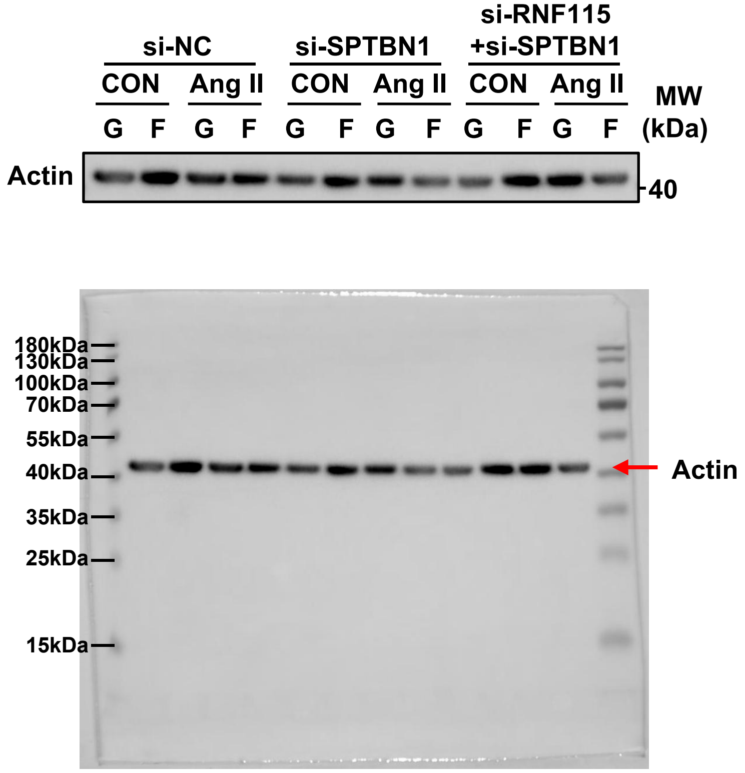

N2

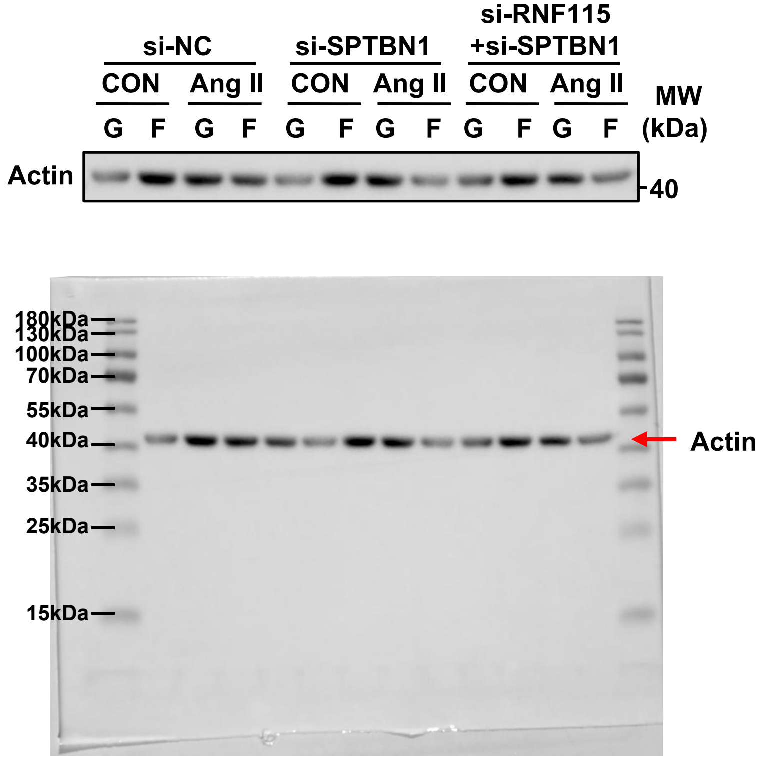

N3

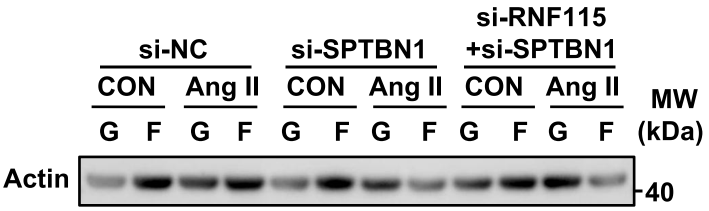
si-NC
si-SPTBN1
si-RNF115
+si-SPTBN1
CON
Ang II
CON
Ang II
CON
Ang II
MW
(kDa)
G F G F G F G F G F G F
Actin
40

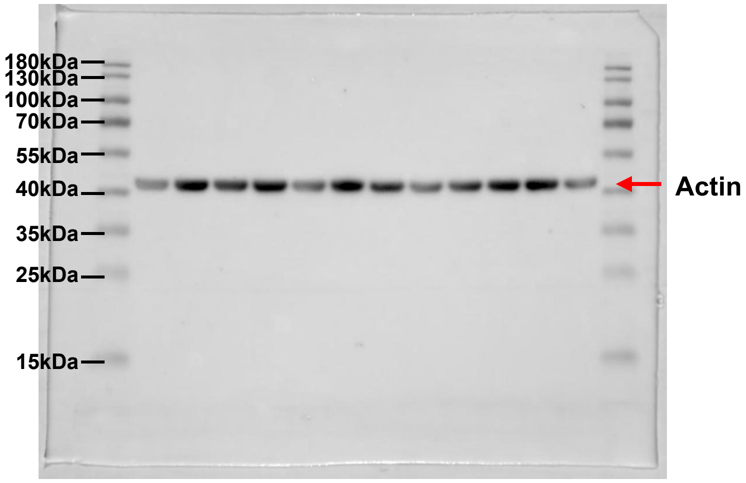
180kDa
130kDa
100kDa
70kDa
55kDa
40kDa
35kDa
25kDa
15kDa
Actin

Figure5G

n=3

N1

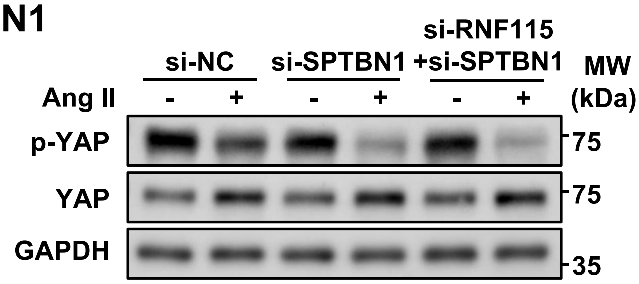

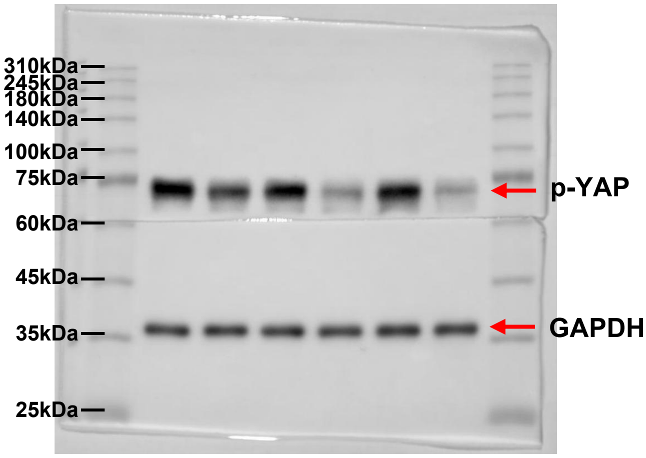

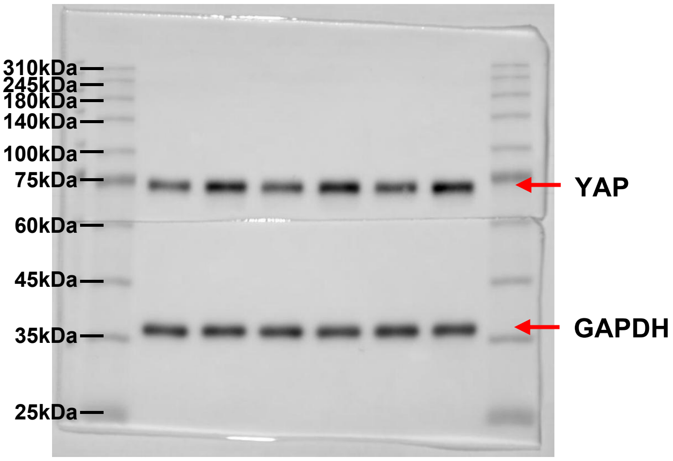

N2

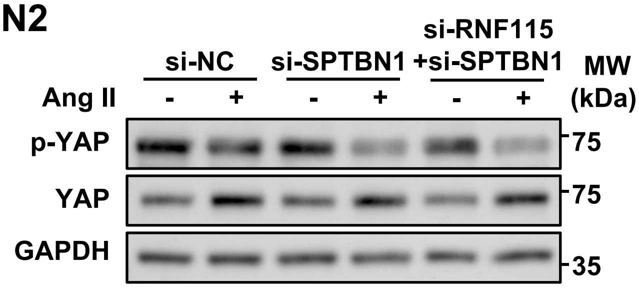

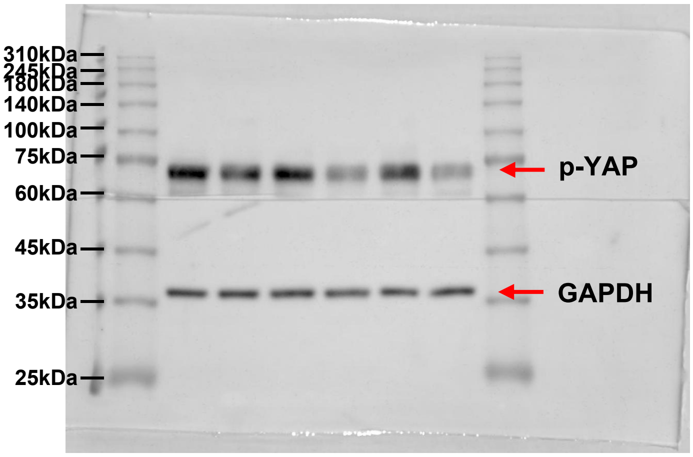

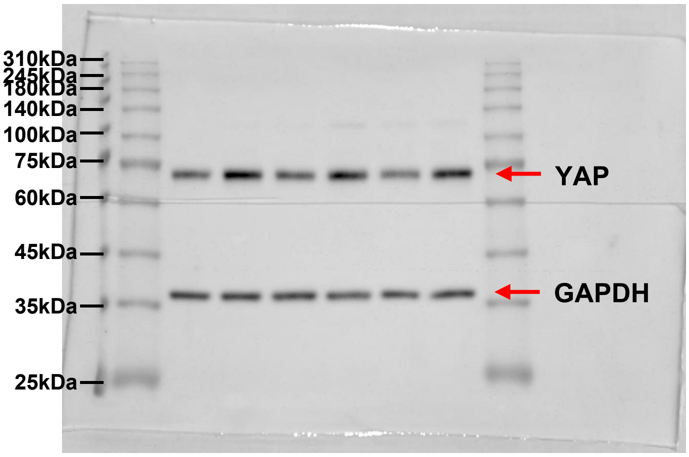

n=3

N3

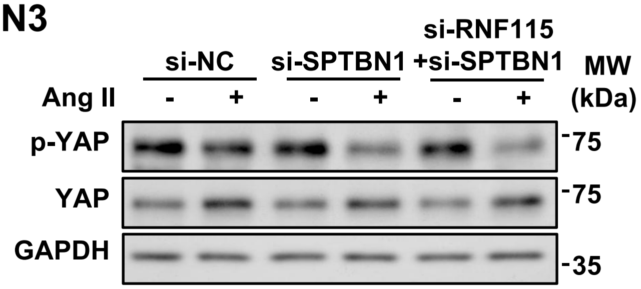

si-NC
si-SPTBN1
si-RNF115
+si-SPTBN1
MW
(kDa)
Ang II
- + - + - +
p-YAP
YAP
GAPDH
75
75
35

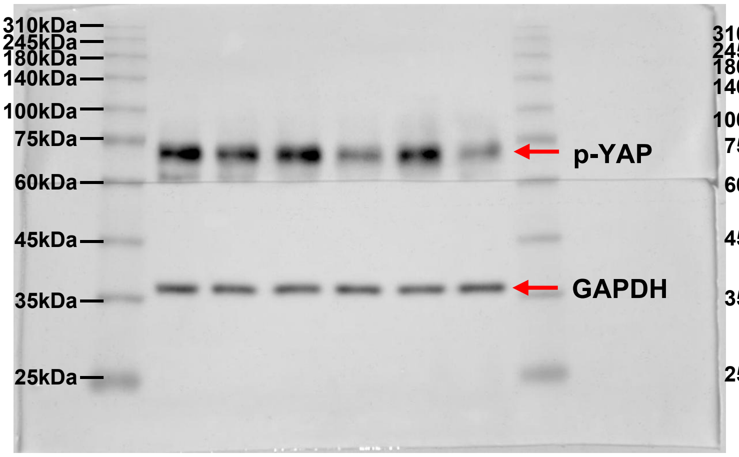

310kDa
245kDa
180kDa
140kDa
100kDa
75kDa
60kDa
45kDa
35kDa
25kDa
p-YAP
GAPDH

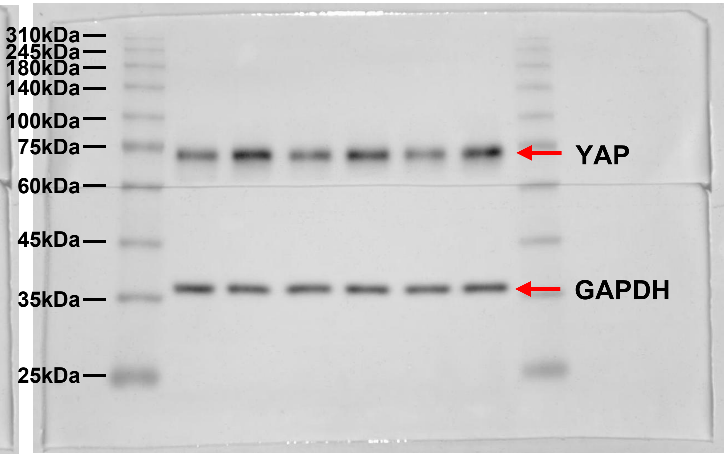

310kDa
245kDa
180kDa
140kDa
100kDa
75kDa
60kDa
45kDa
35kDa
25kDa
YAP
GAPDH

Figure6D

n=3

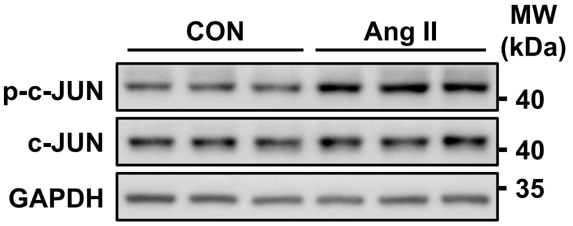
CON
Ang II
MW
(kDa)
p-c-JUN
c-JUN
GAPDH
40
40
35

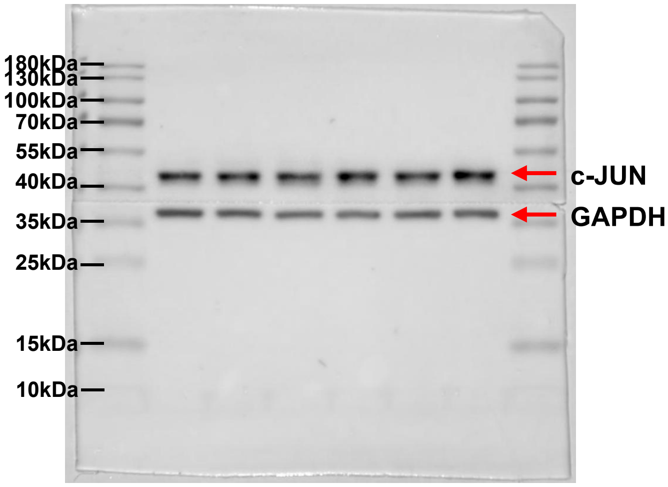
180kDa
130kDa
100kDa
70kDa
55kDa
40kDa
35kDa
25kDa
15kDa
10kDa
c-JUN
GAPDH

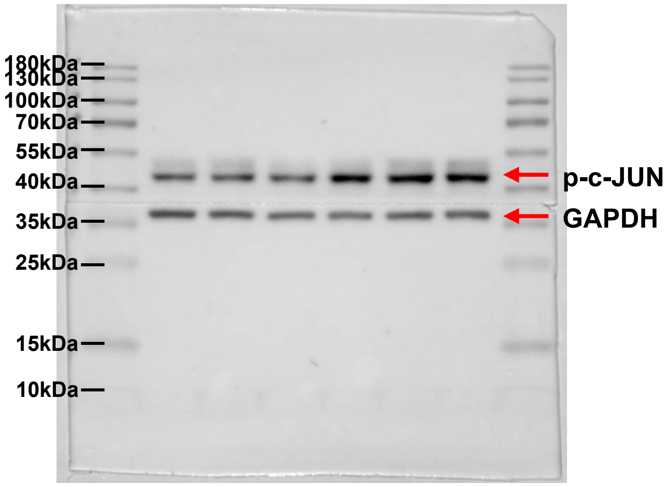
180kDa
130kDa
100kDa
70kDa
55kDa
40kDa
35kDa
25kDa
15kDa
10kDa
p-c-JUN
GAPDH

Figure6F

n=3

N1

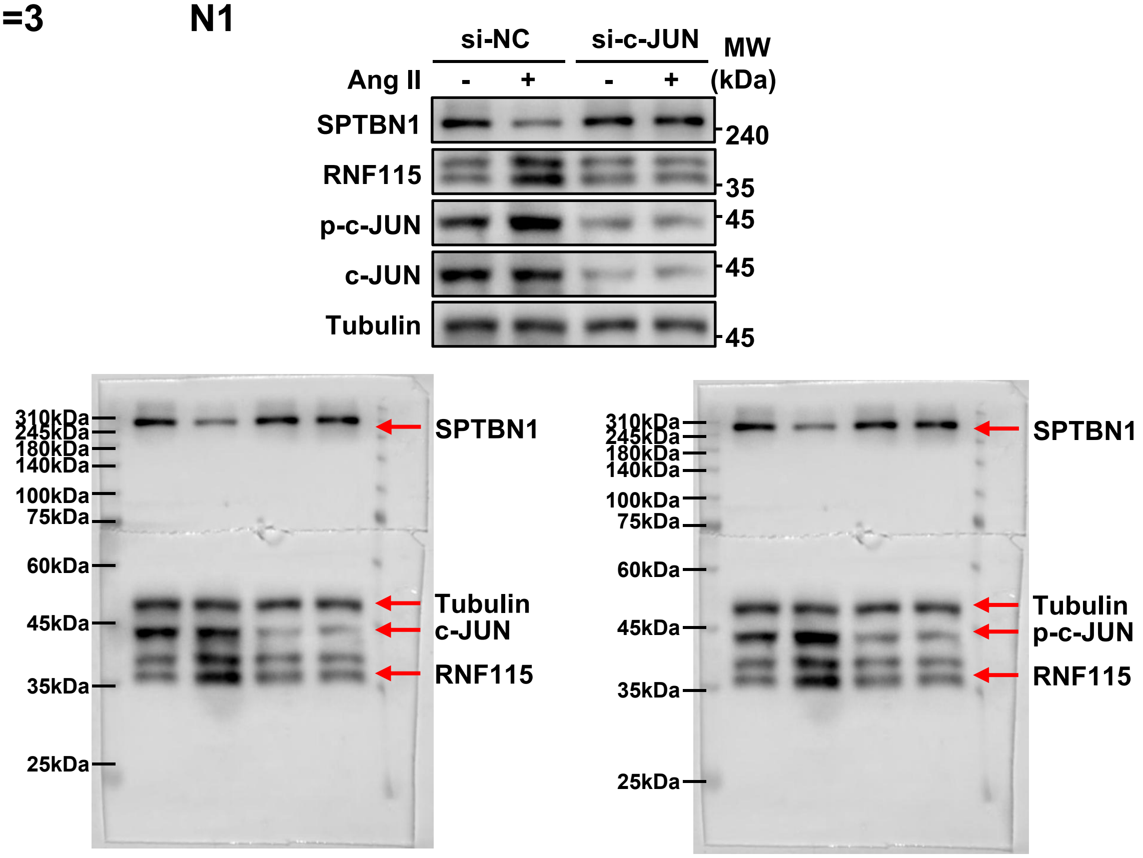

N2

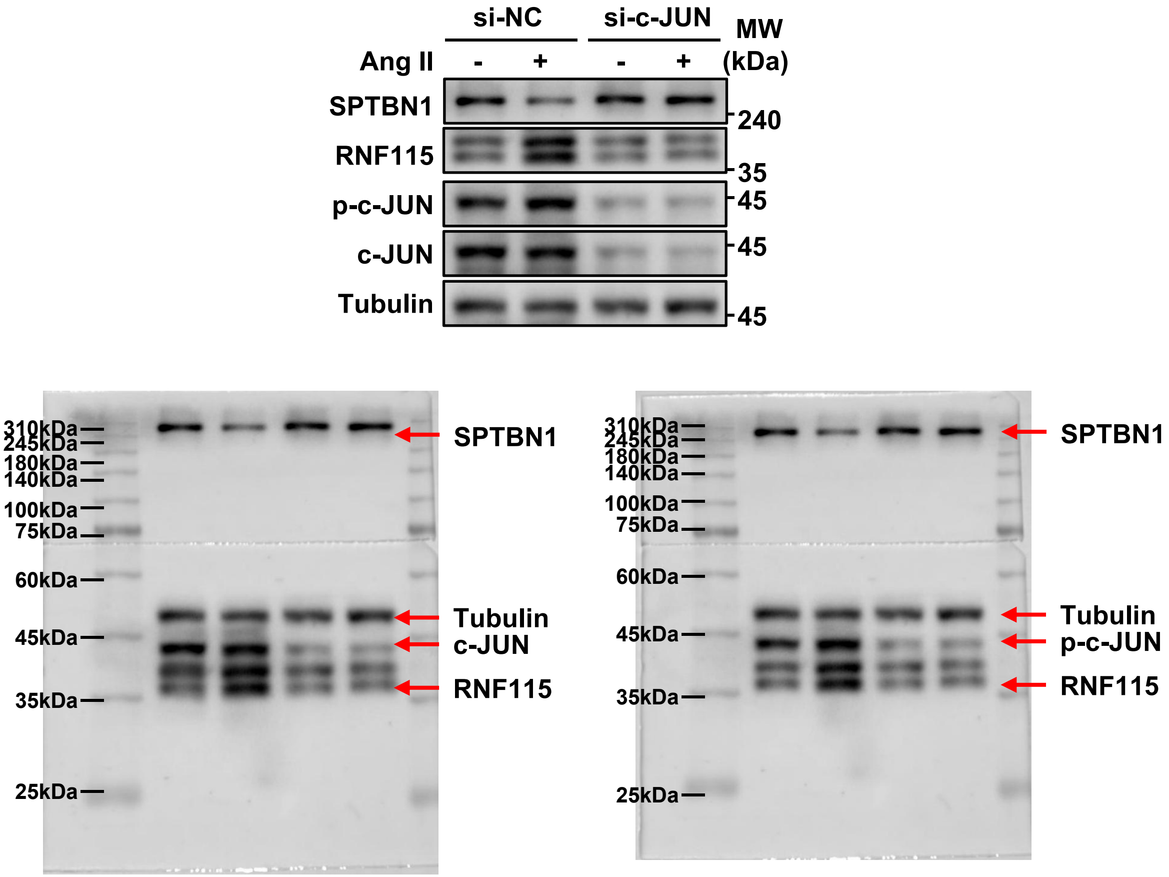

N3

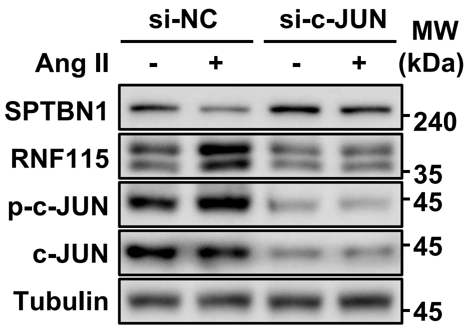
si-NC
si-c-JUN
MW (kDa)
Ang II
-
+
-
+
SPTBN1
240
RNF115
35
p-c-JUN
45
c-JUN
45
Tubulin
45

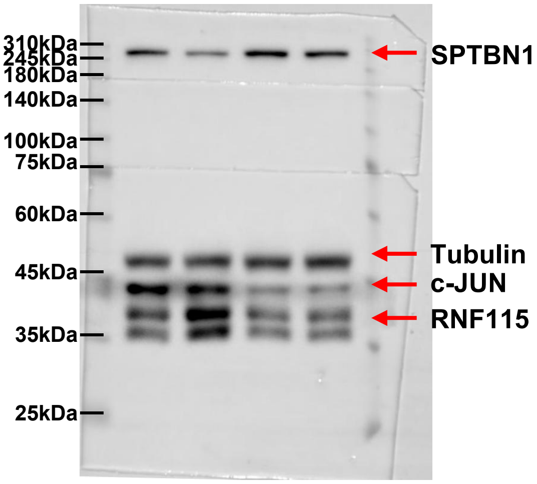
310kDa
245kDa
180kDa
140kDa
100kDa
75kDa
60kDa
45kDa
35kDa
25kDa
SPTBN1
Tubulin
c-JUN
RNF115

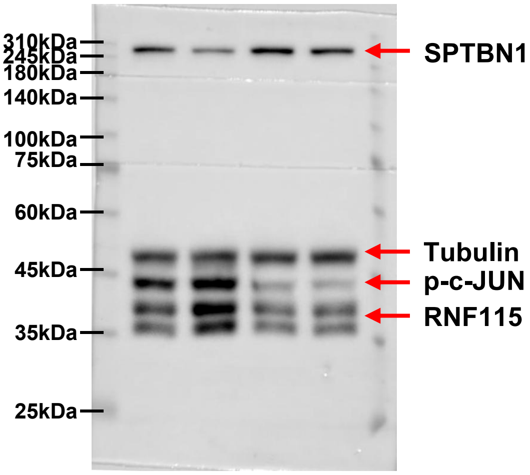
310kDa
245kDa
180kDa
140kDa
100kDa
75kDa
60kDa
45kDa
35kDa
25kDa
SPTBN1
Tubulin
p-c-JUN
RNF115

Figure6G

n=3

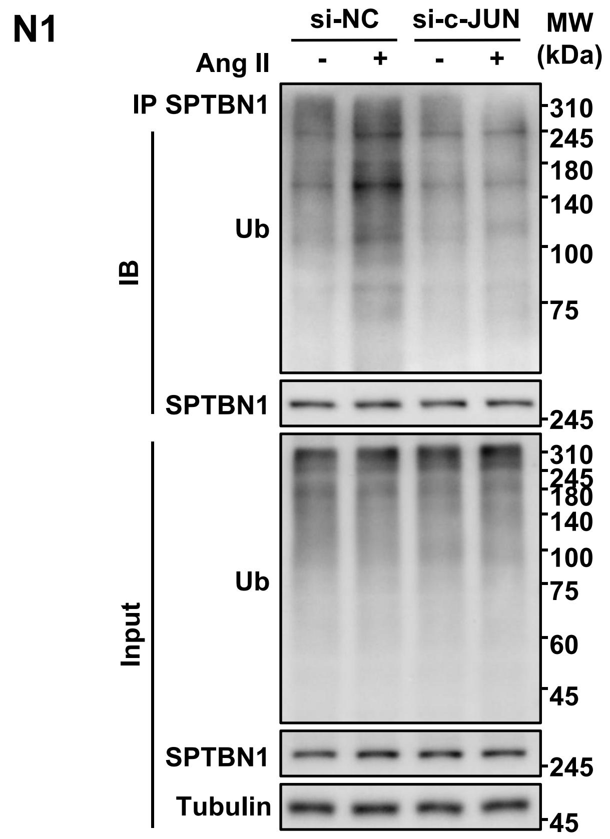
N1
si-NC
si-c-JUN
MW (kDa)
Ang II
- + - +
IP SPTBN1
IB
Ub
SPTBN1
Input
Ub
SPTBN1
Tubulin
310
245
180
140
100
75
245
310
245
180
140
100
75
60
45
245
45

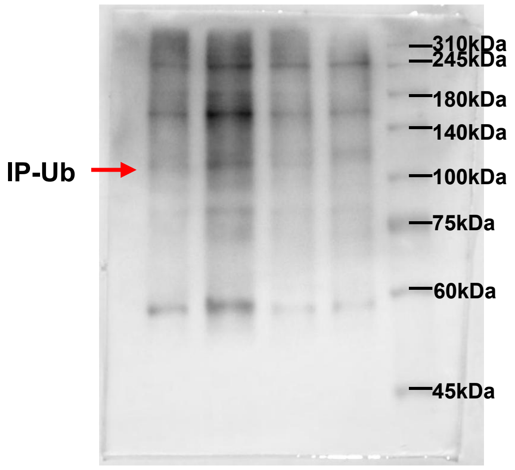
IP-Ub
310kDa
245kDa
180kDa
140kDa
100kDa
75kDa
60kDa
45kDa

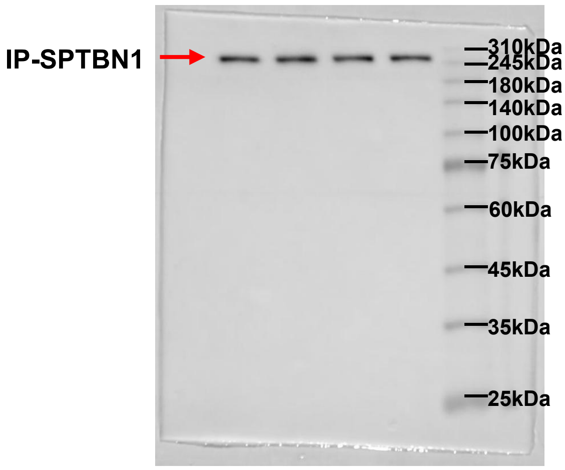
IP-SPTBN1
310kDa
245kDa
180kDa
140kDa
100kDa
75kDa
60kDa
45kDa
35kDa
25kDa

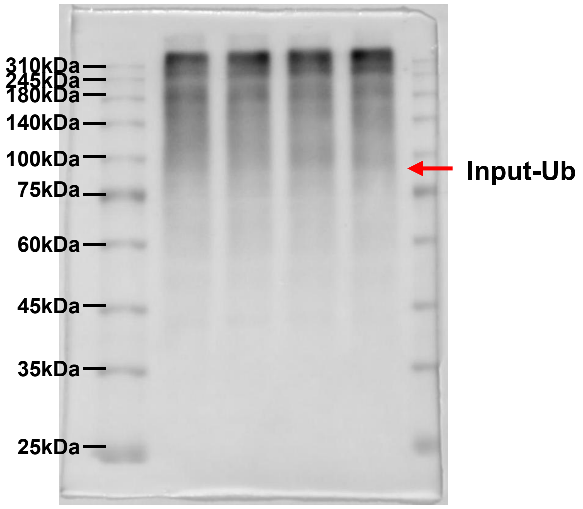
310kDa
245kDa
180kDa
140kDa
100kDa
75kDa
60kDa
45kDa
35kDa
25kDa
Input-Ub

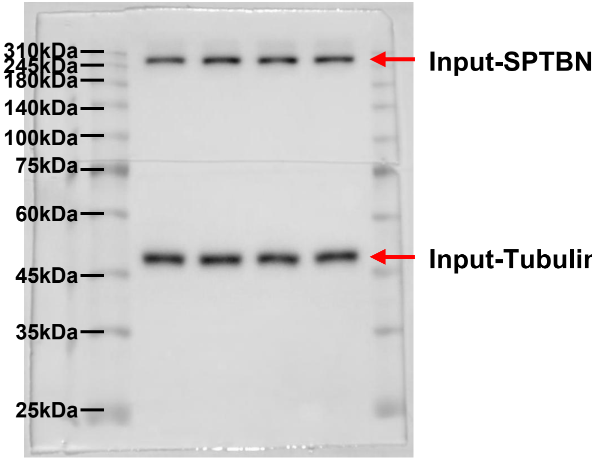
310kDa
245kDa
180kDa
140kDa
100kDa
75kDa
60kDa
45kDa
35kDa
25kDa
Input-SPTBN
Input-Tubulir

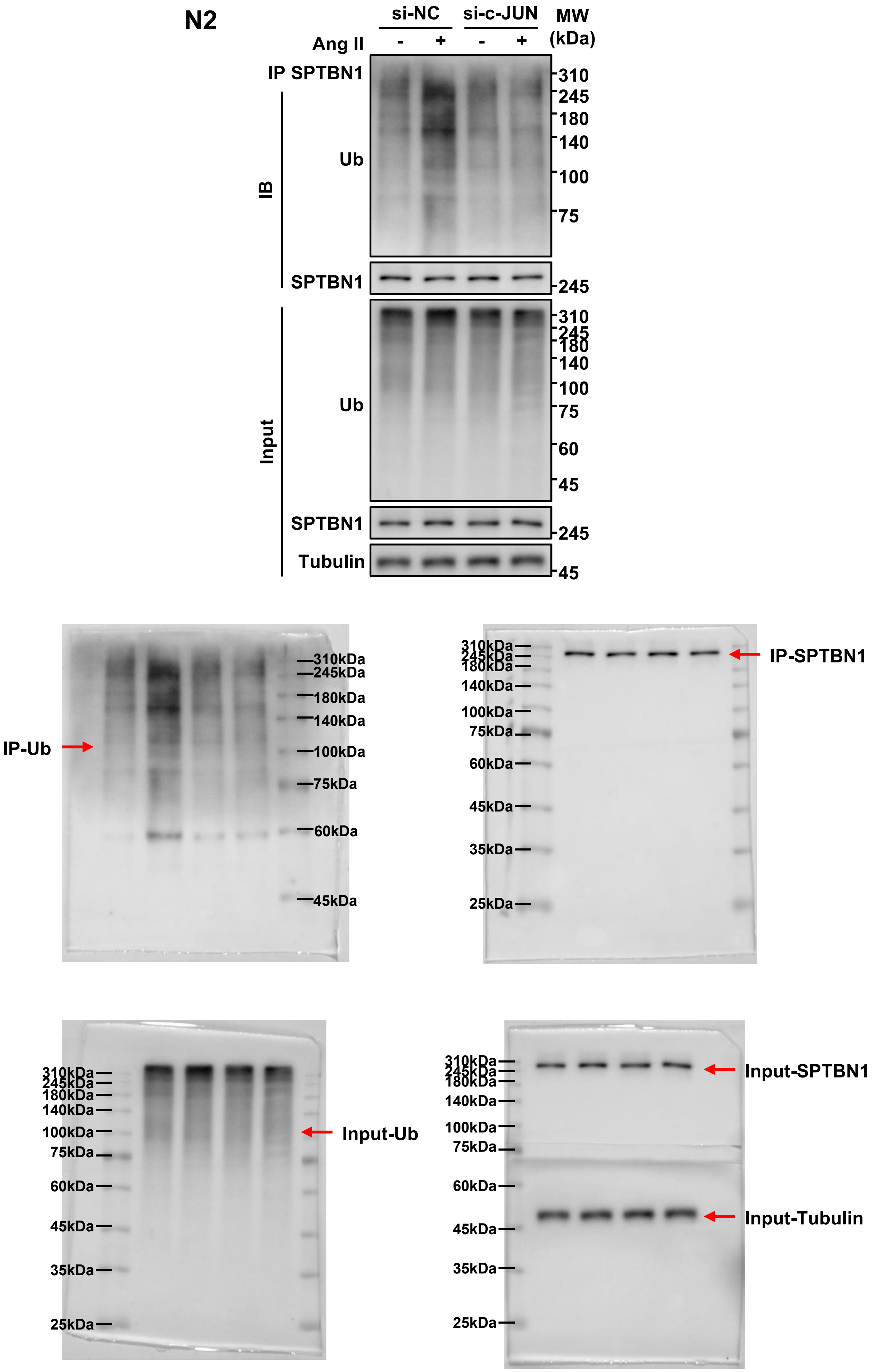
N2
si-NC
si-c-JUN
MW (kDa)
Ang II
-
+
-
+
IP SPTBN1
IB
Ub
SPTBN1
Input
Ub
SPTBN1
Tubulin
310
245
180
140
100
75
60
45
IP-Ub
IP-SPTBN1
Input-Ub
Input-SPTBN1
Input-Tubulin
310kDa
245kDa
180kDa
140kDa
100kDa
75kDa
60kDa
45kDa
35kDa
25kDa

N3

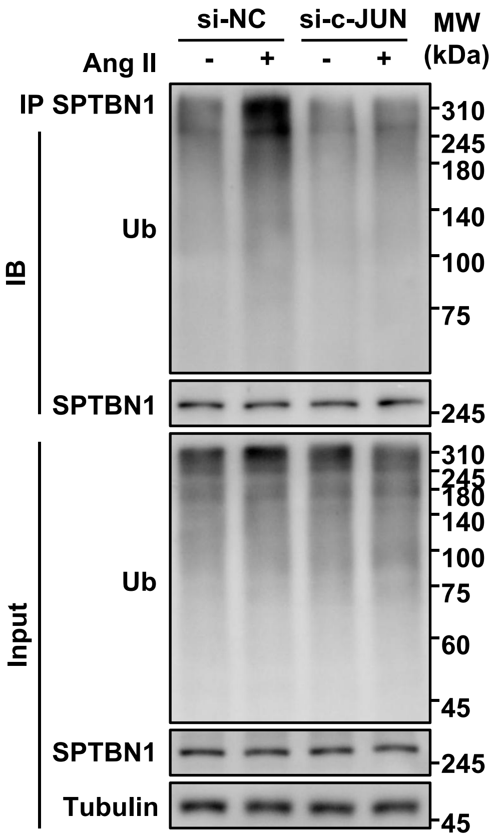

si-NC
si-c-JUN
MW (kDa)
Ang II
- + - +
IP SPTBN1
IB
Ub
SPTBN1
Input
Ub
SPTBN1
Tubulin
310
245
180
140
100
75
245
310
245
180
140
100
75
60
45
245
45

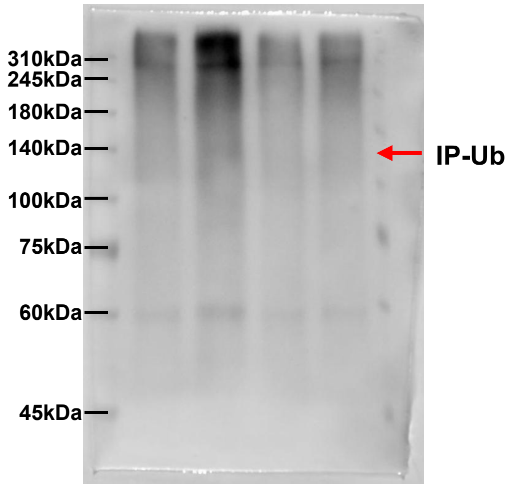

310kDa
245kDa
180kDa
140kDa
100kDa
75kDa
60kDa
45kDa
IP-Ub

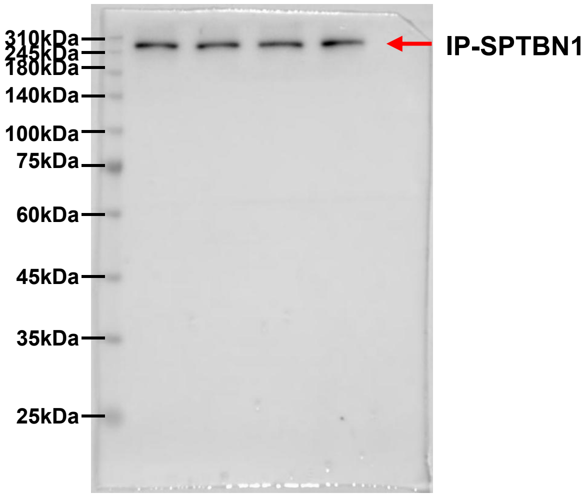

310kDa
245kDa
180kDa
140kDa
100kDa
75kDa
60kDa
45kDa
35kDa
25kDa
IP-SPTBN1

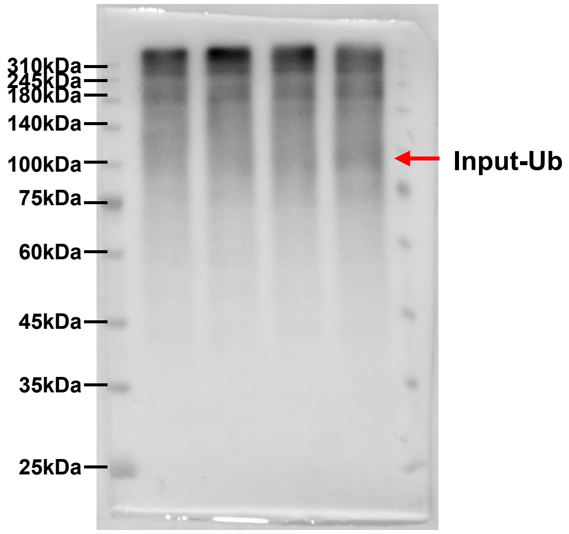

310kDa
245kDa
180kDa
140kDa
100kDa
75kDa
60kDa
45kDa
35kDa
25kDa
Input-Ub

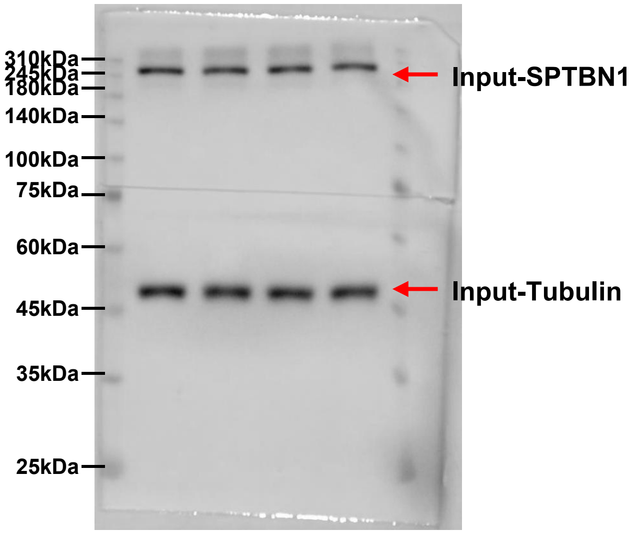

310kDa
245kDa
180kDa
140kDa
100kDa
75kDa
60kDa
45kDa
35kDa
25kDa
Input-SPTBN1
Input-Tubulin

Figure7B

n=3

N1

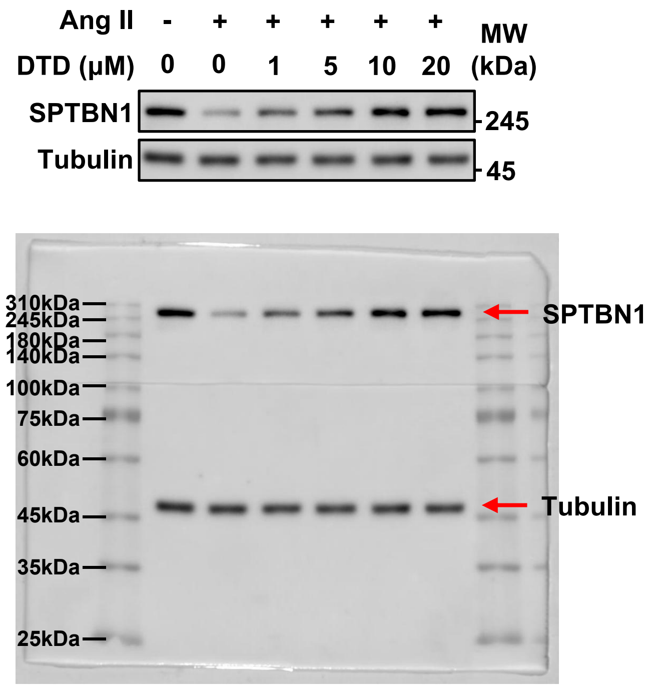

N2

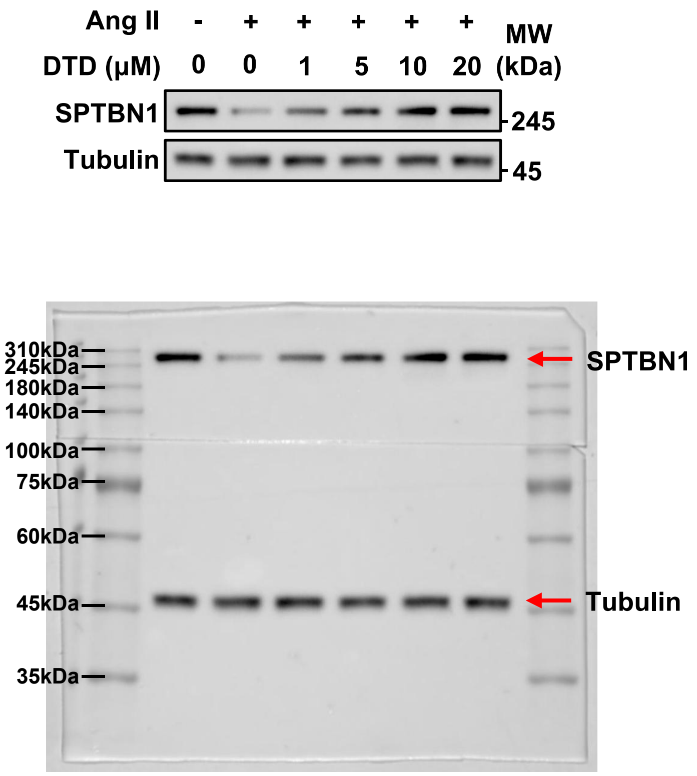

N3

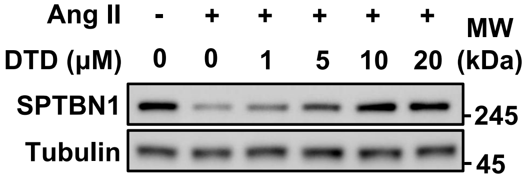
Ang II - + + + + +
DTD (μM) 0 0 1 5 10 20
MW (kDa)
SPTBN1
Tubulin
245
45

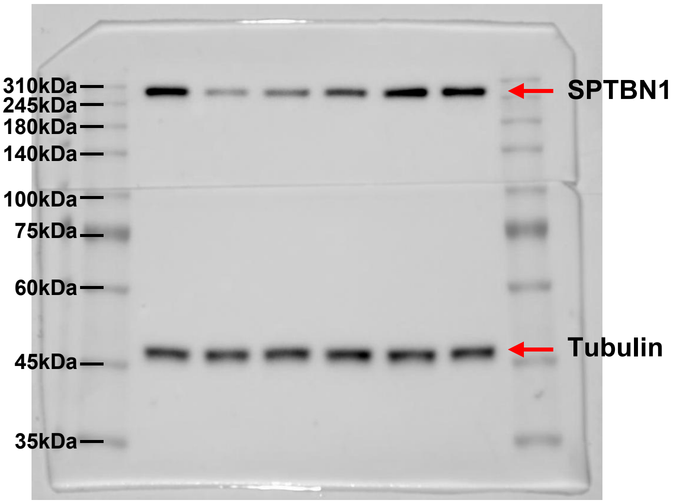
310kDa
245kDa
180kDa
140kDa
100kDa
75kDa
60kDa
45kDa
35kDa
SPTBN1
Tubulin

Figure7C

n=3

N1

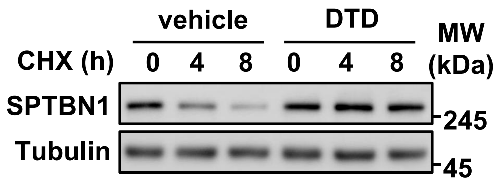

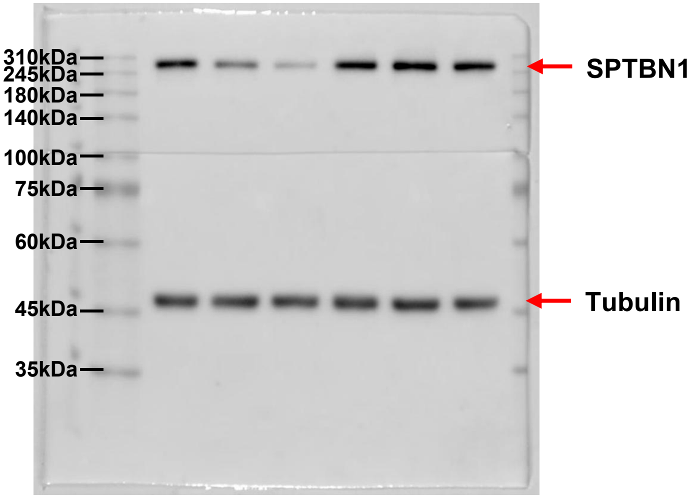

N2

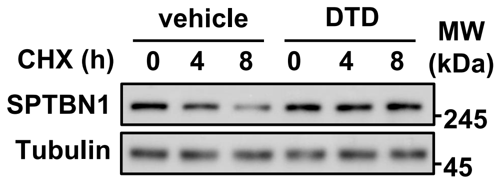

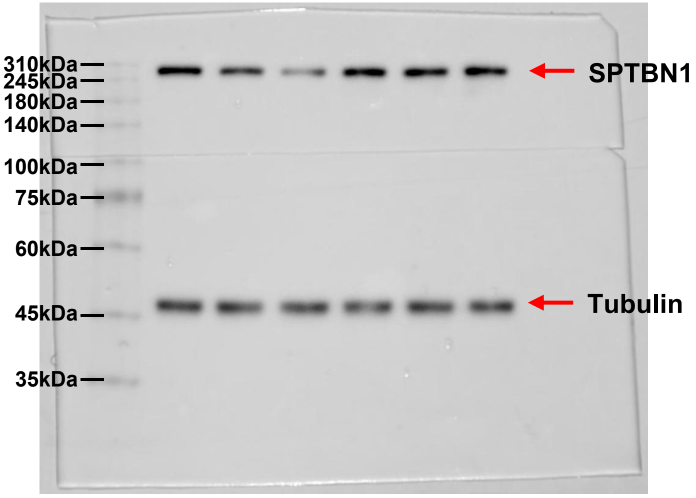

N3

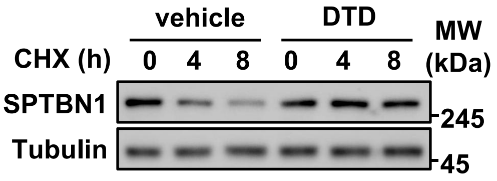
vehicle
DTD
MW (kDa)
CHX (h)
0
4
8
0
4
8
SPTBN1
Tubulin
245
45

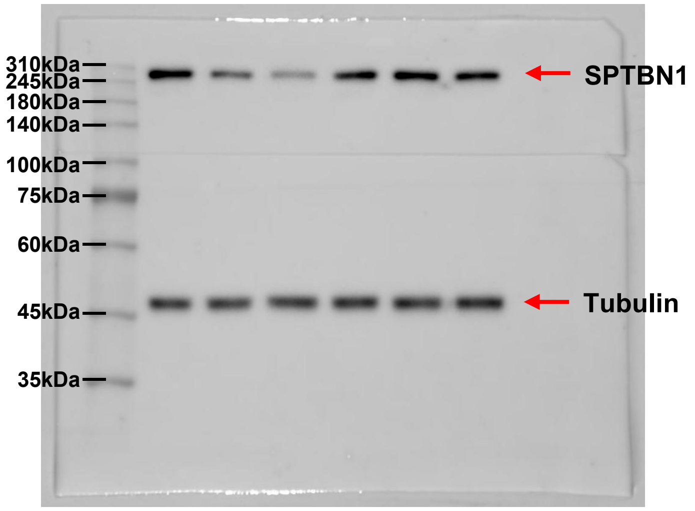
310kDa
245kDa
180kDa
140kDa
100kDa
75kDa
60kDa
45kDa
35kDa
SPTBN1
Tubulin

Figure7F

n=3

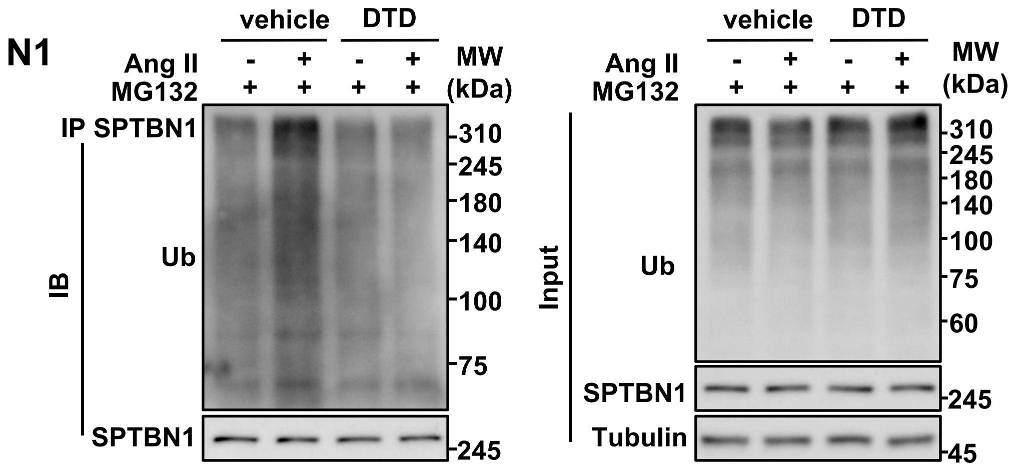
N1
vehicle
DTD
Ang II
MG132
MW (kDa)
IP SPTBN1
IB
Ub
SPTBN1
310
245
180
140
100
75
Input
Tubulin
60
45

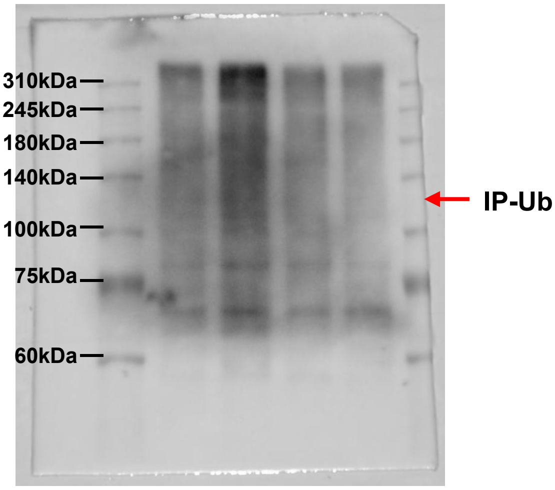
310kDa
245kDa
180kDa
140kDa
100kDa
75kDa
60kDa
IP-Ub

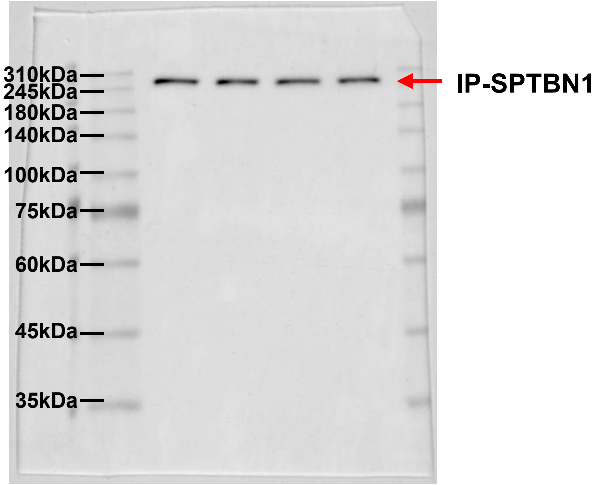
310kDa
245kDa
180kDa
140kDa
100kDa
75kDa
60kDa
45kDa
35kDa
IP-SPTBN1

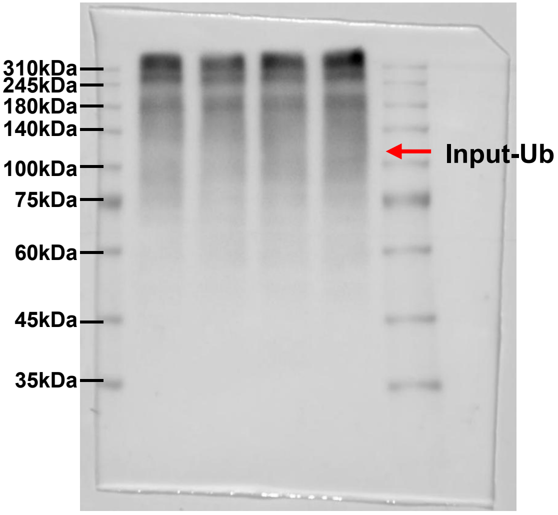
310kDa
245kDa
180kDa
140kDa
100kDa
75kDa
60kDa
45kDa
35kDa
Input-Ub

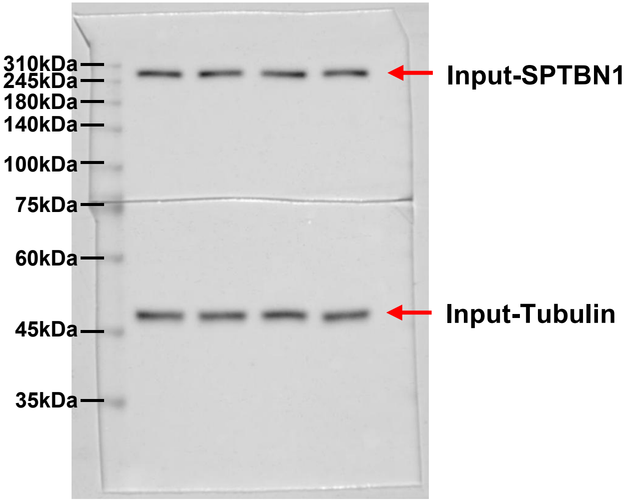
310kDa
245kDa
180kDa
140kDa
100kDa
75kDa
60kDa
45kDa
35kDa
Input-SPTBN1
Input-Tubulin

N2

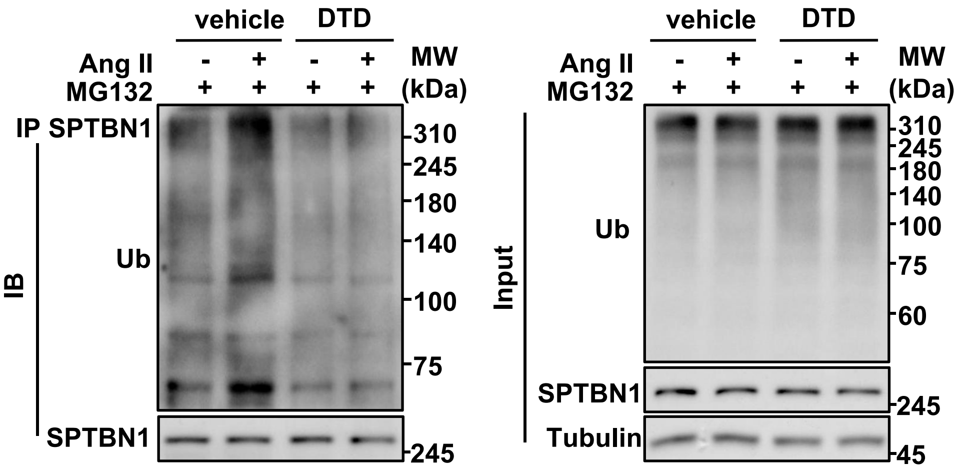
vehicle
DTD
Ang II
MG132
MW (kDa)
IP SPTBN1
IB
Ub
SPTBN1
310
245
180
140
100
75
245
Input
Ub
SPTBN1
Tubulin
310
245
180
140
100
75
60
245
45

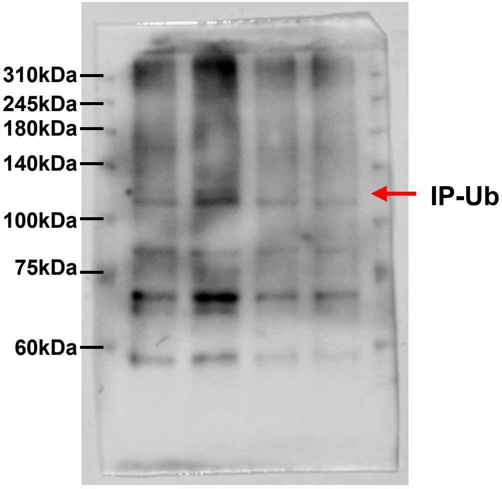
310kDa
245kDa
180kDa
140kDa
100kDa
75kDa
60kDa
IP-Ub

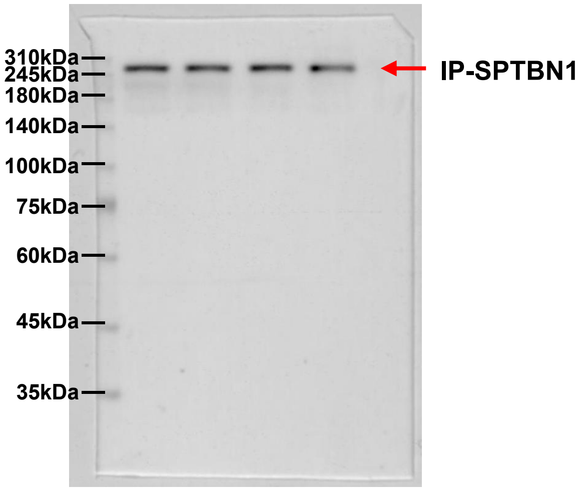
310kDa
245kDa
180kDa
140kDa
100kDa
75kDa
60kDa
45kDa
35kDa
IP-SPTBN1

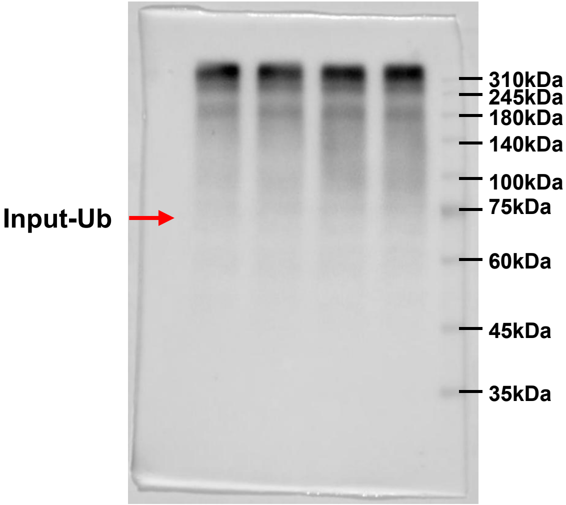
Input-Ub
310kDa
245kDa
180kDa
140kDa
100kDa
75kDa
60kDa
45kDa
35kDa

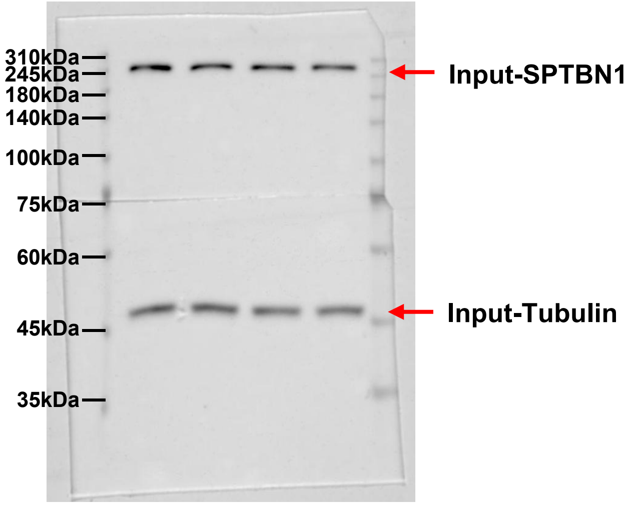
310kDa
245kDa
180kDa
140kDa
100kDa
75kDa
60kDa
45kDa
35kDa
Input-SPTBN1
Input-Tubulin

N3

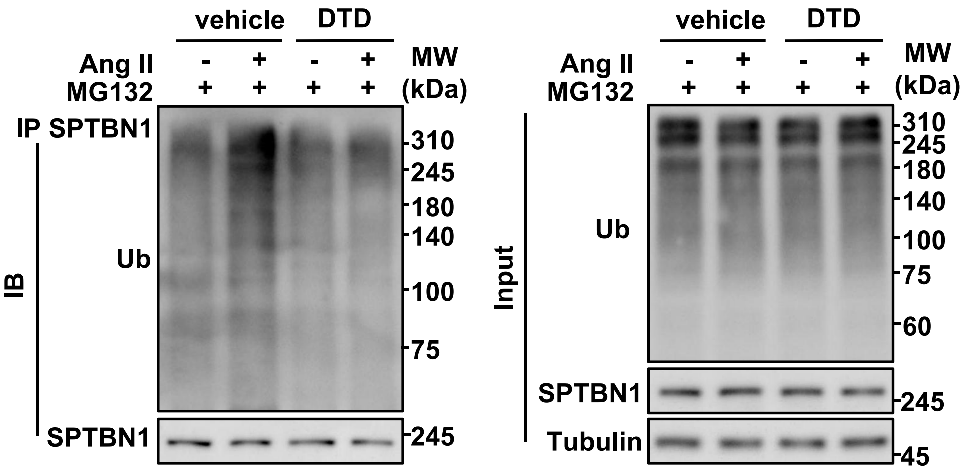
vehicle
DTD
Ang II
MG132
MW (kDa)
IP SPTBN1
IB
Ub
SPTBN1
310
245
180
140
100
75
60
45
Input
Tubulin

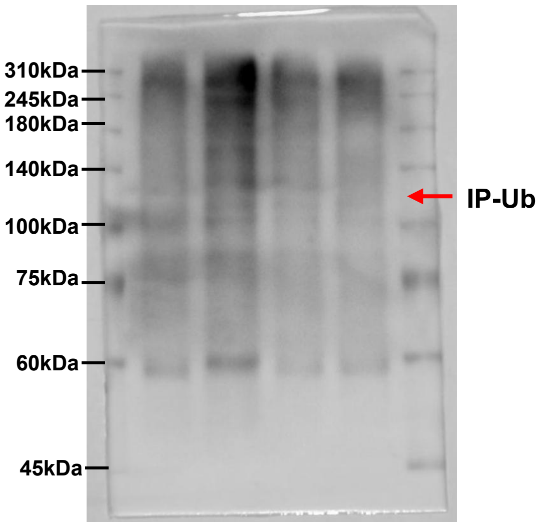
310kDa
245kDa
180kDa
140kDa
100kDa
75kDa
60kDa
45kDa
IP-Ub

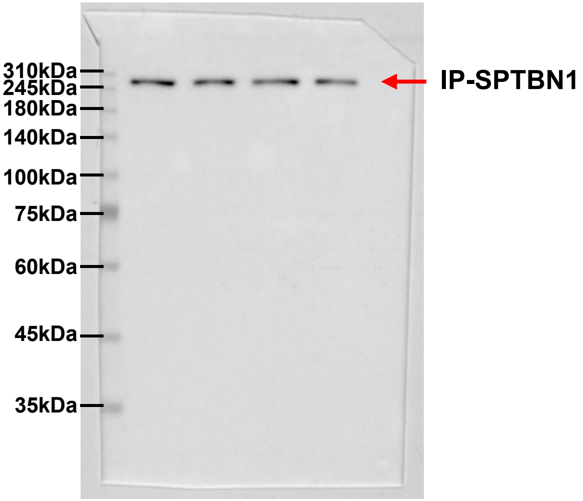
310kDa
245kDa
180kDa
140kDa
100kDa
75kDa
60kDa
45kDa
35kDa
IP-SPTBN1

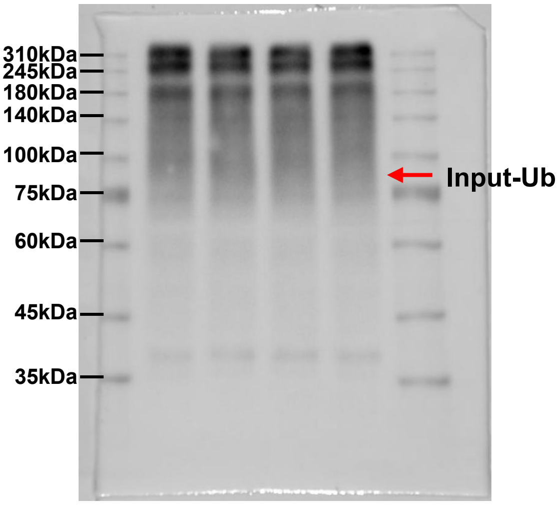
310kDa
245kDa
180kDa
140kDa
100kDa
75kDa
60kDa
45kDa
35kDa
Input-Ub

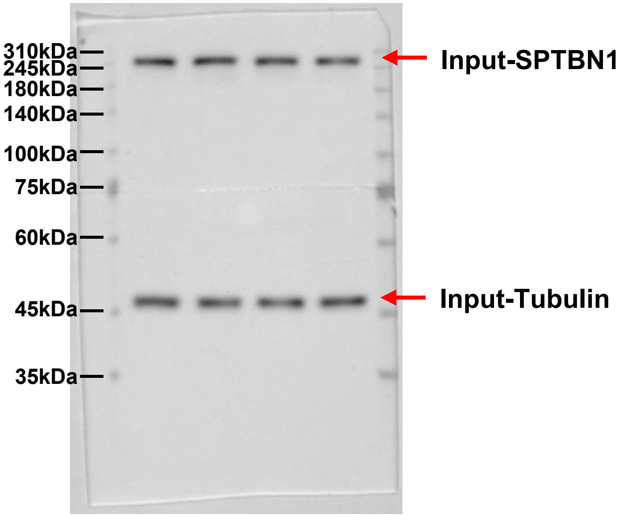
310kDa
245kDa
180kDa
140kDa
100kDa
75kDa
60kDa
45kDa
35kDa
Input-SPTBN1
Input-Tubulin

Figure8J

n=3

N1

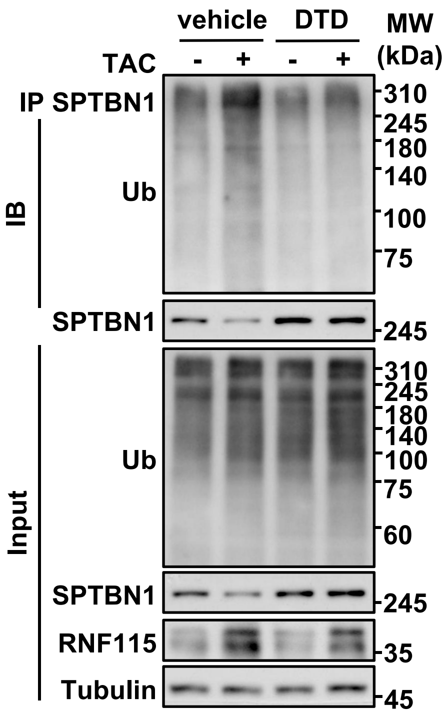

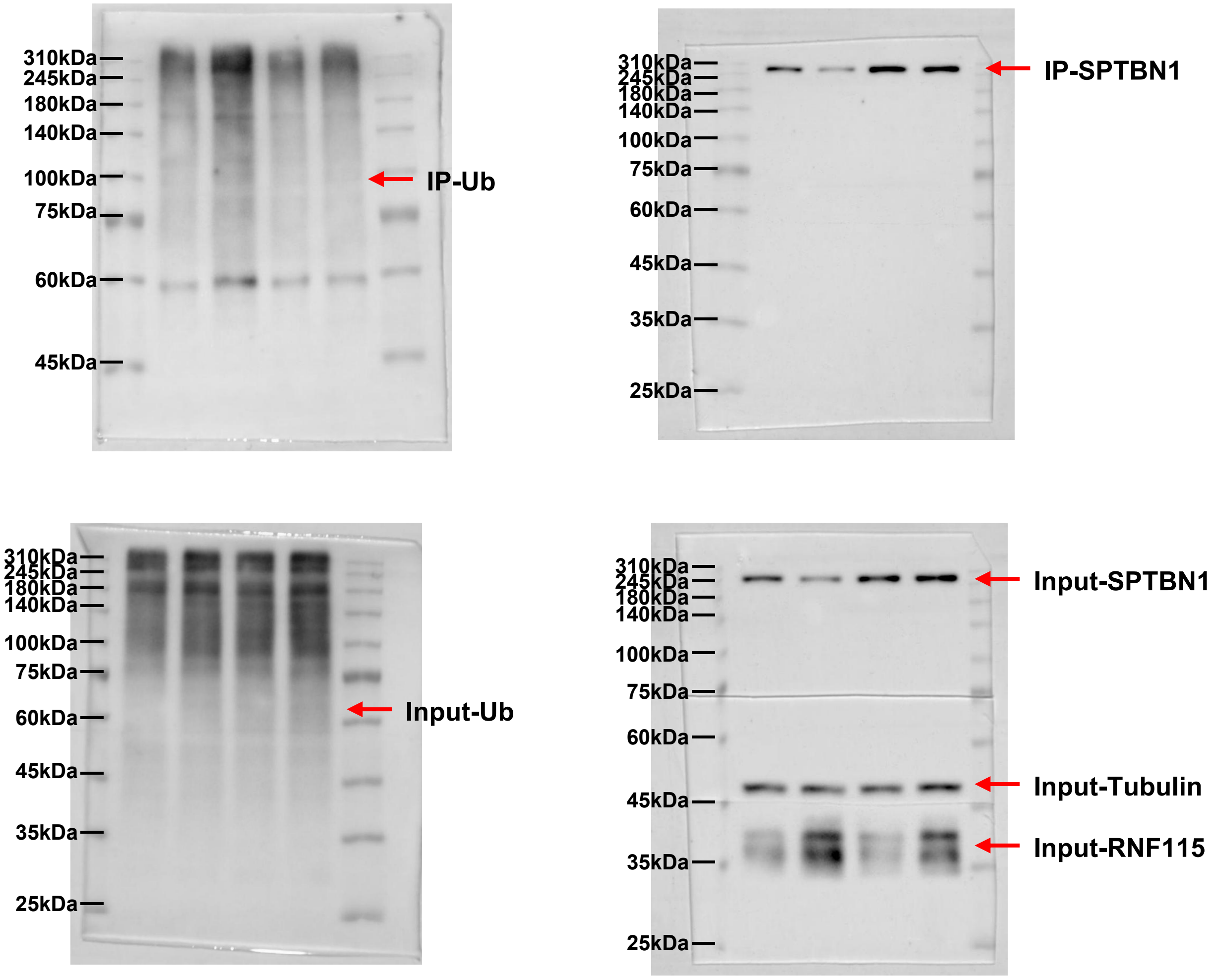

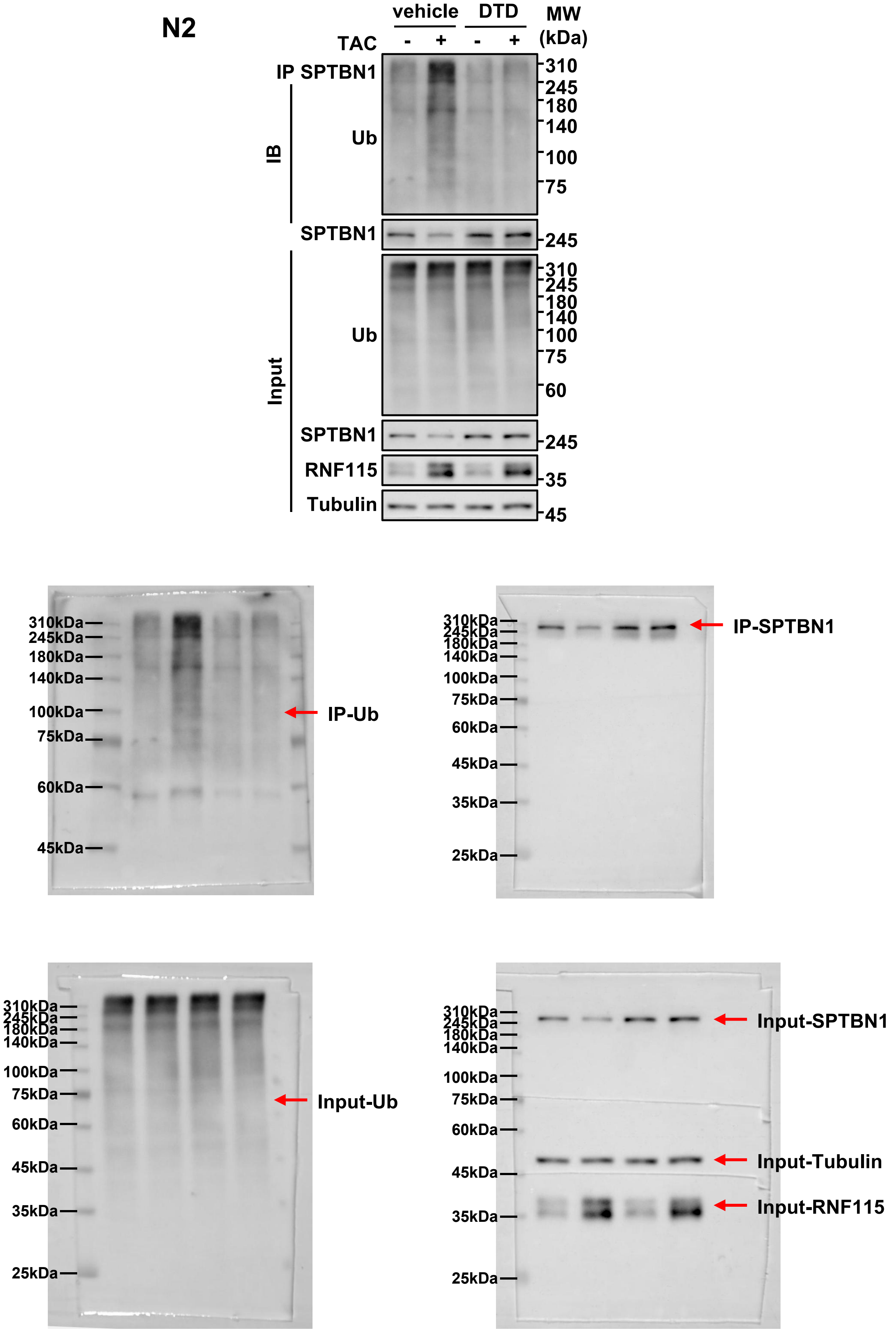
N2
vehicle
DTD
MW (kDa)
TAC
- + - +
IP SPTBN1
IB
Ub
310
245
180
140
100
75
SPTBN1
245
Input
Ub
310
245
180
140
100
75
60
SPTBN1
245
RNF115
35
Tubulin
45
310kDa
245kDa
180kDa
140kDa
100kDa
75kDa
60kDa
45kDa
IP-Ub
310kDa
245kDa
180kDa
140kDa
100kDa
75kDa
60kDa
45kDa
35kDa
25kDa
IP-SPTBN1
310kDa
245kDa
180kDa
140kDa
100kDa
75kDa
60kDa
45kDa
35kDa
25kDa
Input-Ub
310kDa
245kDa
180kDa
140kDa
100kDa
75kDa
60kDa
45kDa
35kDa
25kDa
Input-SPTBN1
Input-Tubulin
Input-RNF115

N3

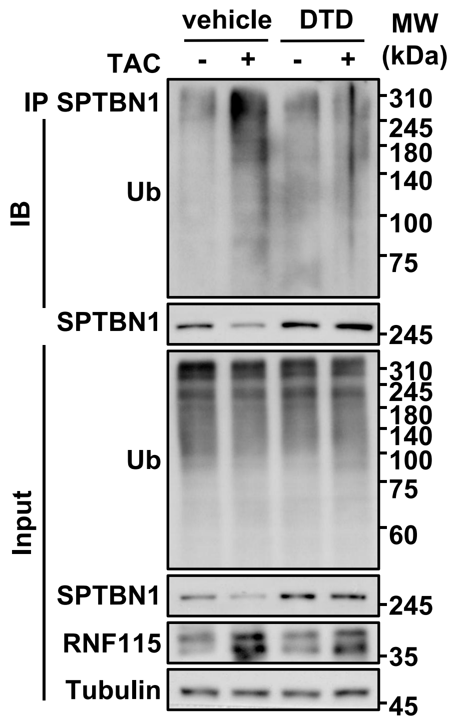

vehicle
DTD
MW (kDa)
TAC
- + - +
IP SPTBN1
IB
Ub
SPTBN1
Input
Ub
SPTBN1
RNF115
Tubulin
310
245
180
140
100
75
245
310
245
180
140
100
75
60
245
35
45

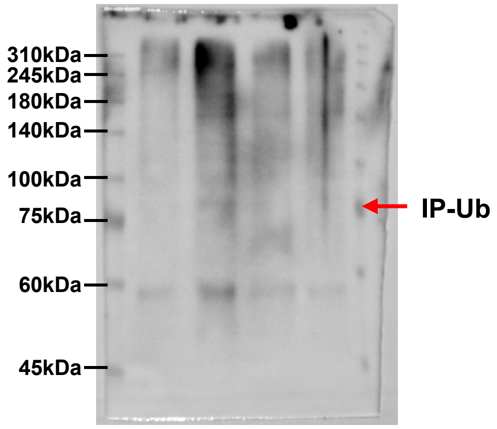

310kDa
245kDa
180kDa
140kDa
100kDa
75kDa
60kDa
45kDa
IP-Ub

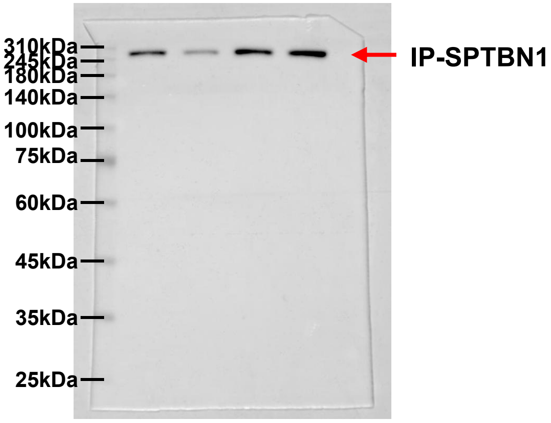

310kDa
245kDa
180kDa
140kDa
100kDa
75kDa
60kDa
45kDa
35kDa
25kDa
IP-SPTBN1

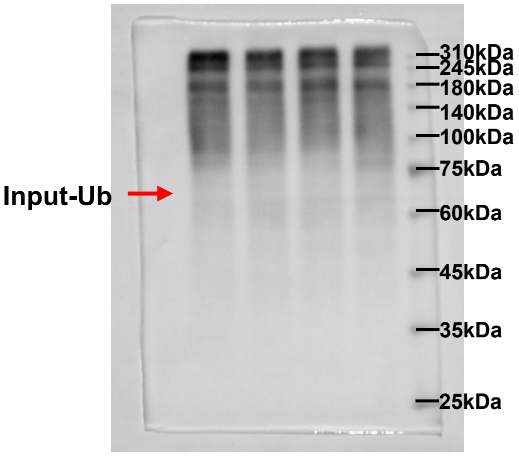

Input-Ub
310kDa
245kDa
180kDa
140kDa
100kDa
75kDa
60kDa
45kDa
35kDa
25kDa

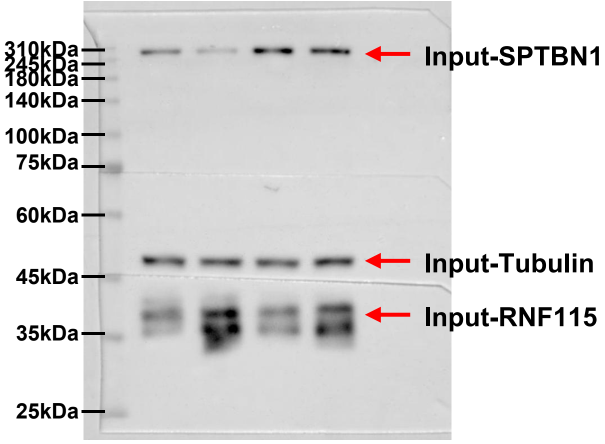

310kDa
245kDa
180kDa
140kDa
100kDa
75kDa
60kDa
45kDa
35kDa
25kDa
Input-SPTBN1
Input-Tubulin
Input-RNF115

Supplementary Figure2B

n=3

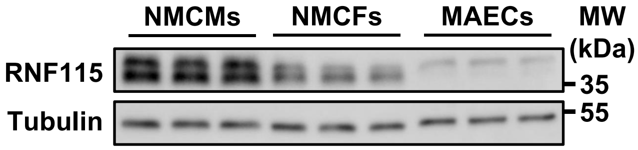

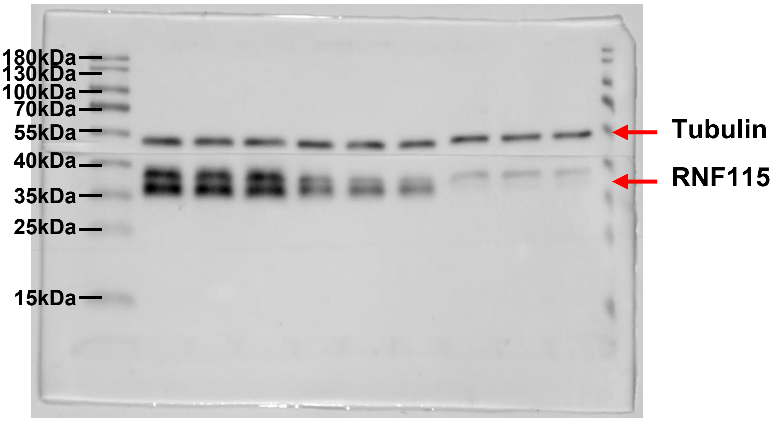

Supplementary Figure2C

n=3

N1

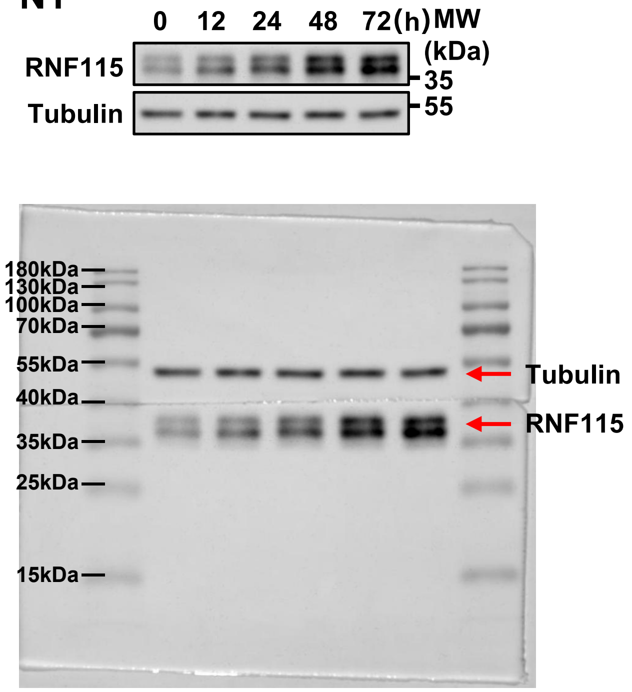

N2

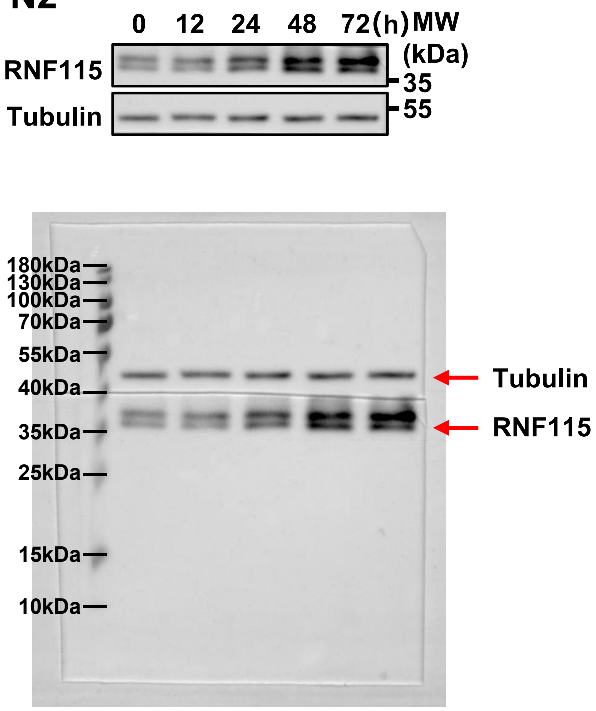

N3

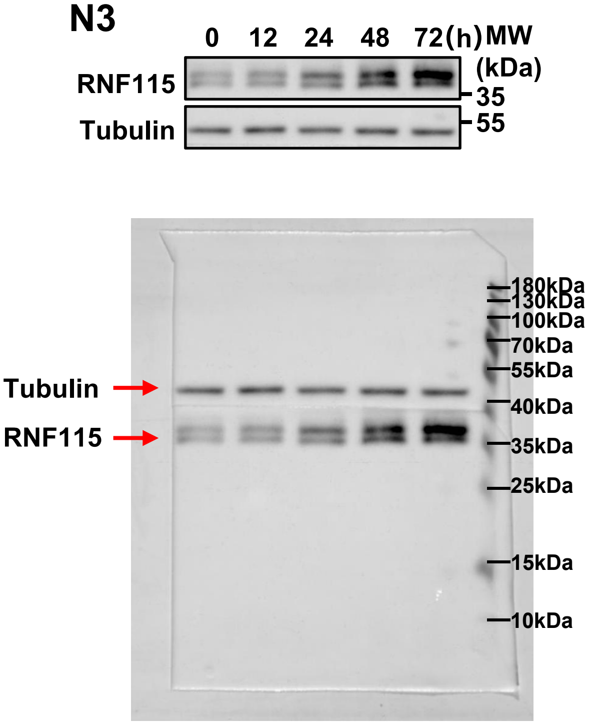

Supplementary Figure4A

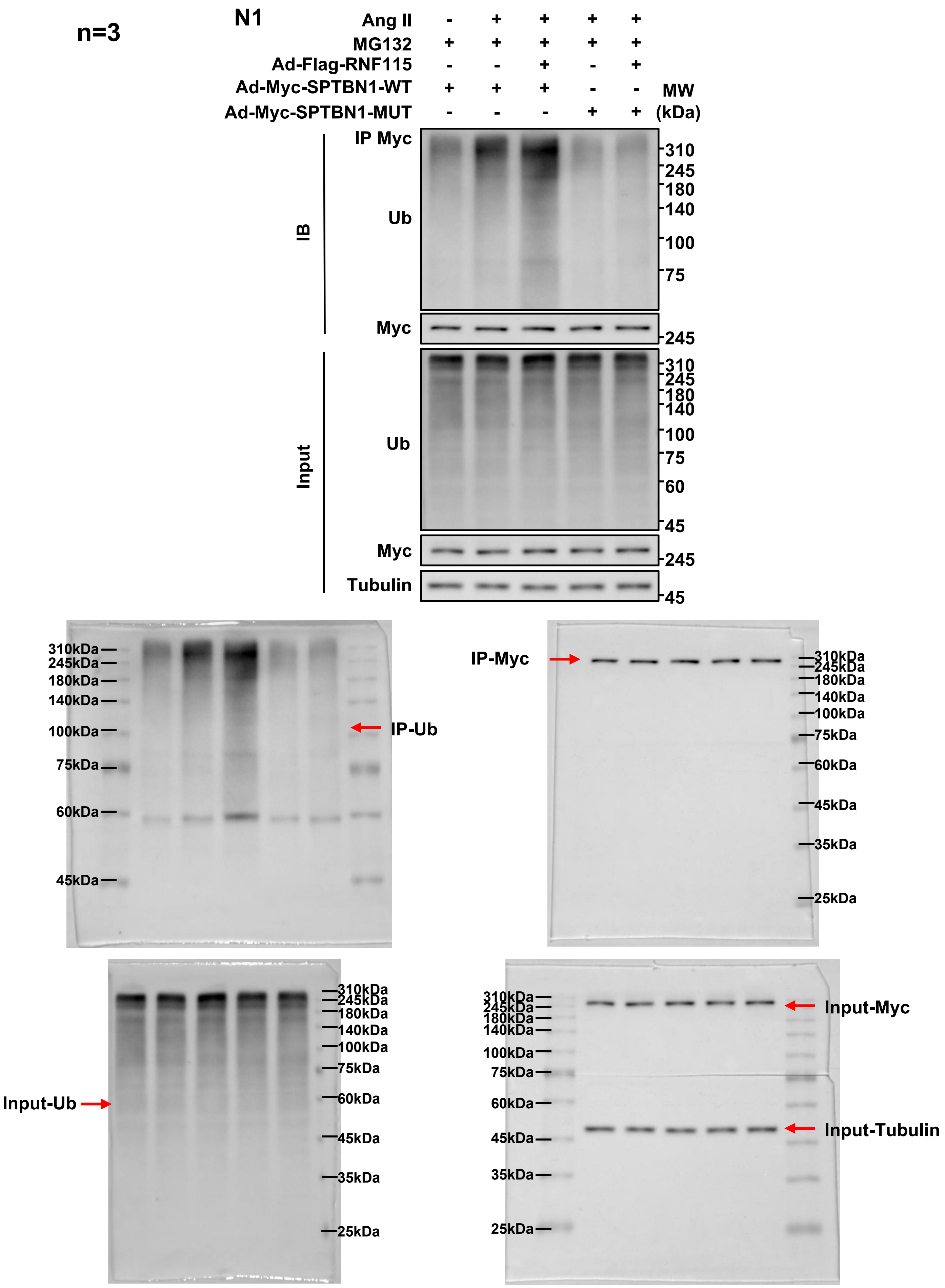

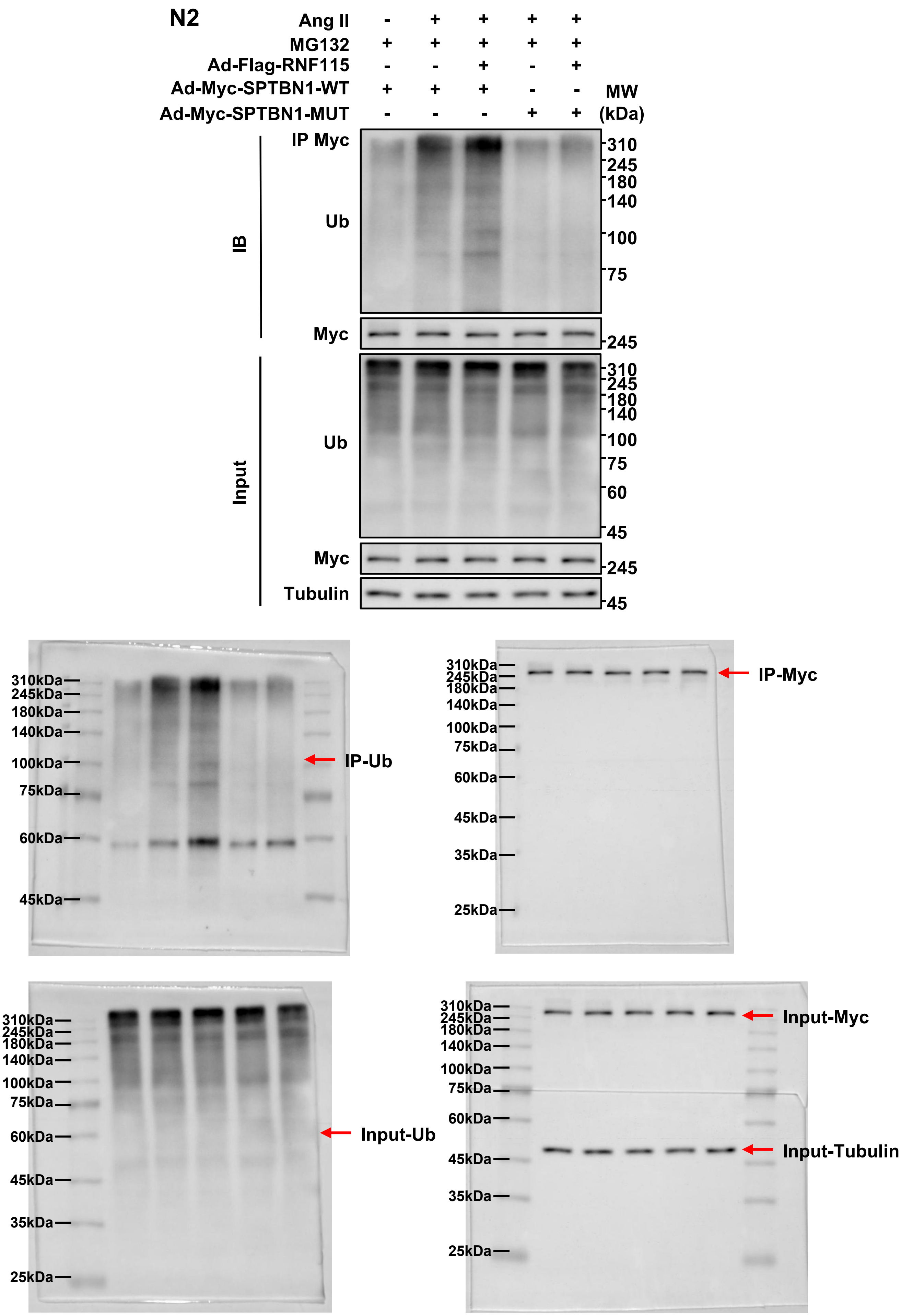

N2
Ang II - + + + +
MG132 + + + + +
Ad-Flag-RNF115 - - + - +
Ad-Myc-SPTBN1-WT + + + - -
Ad-Myc-SPTBN1-MUT - - - + +
MW (kDa)
IB
IP Myc
Ub
Myc
Input
Ub
Myc
Tubulin
310
245
180
140
100
75
60
45
IP-Ub
IP-Myc
Input-Ub
Input-Myc
Input-Tubulin
310kDa
245kDa
180kDa
140kDa
100kDa
75kDa
60kDa
45kDa
35kDa
25kDa

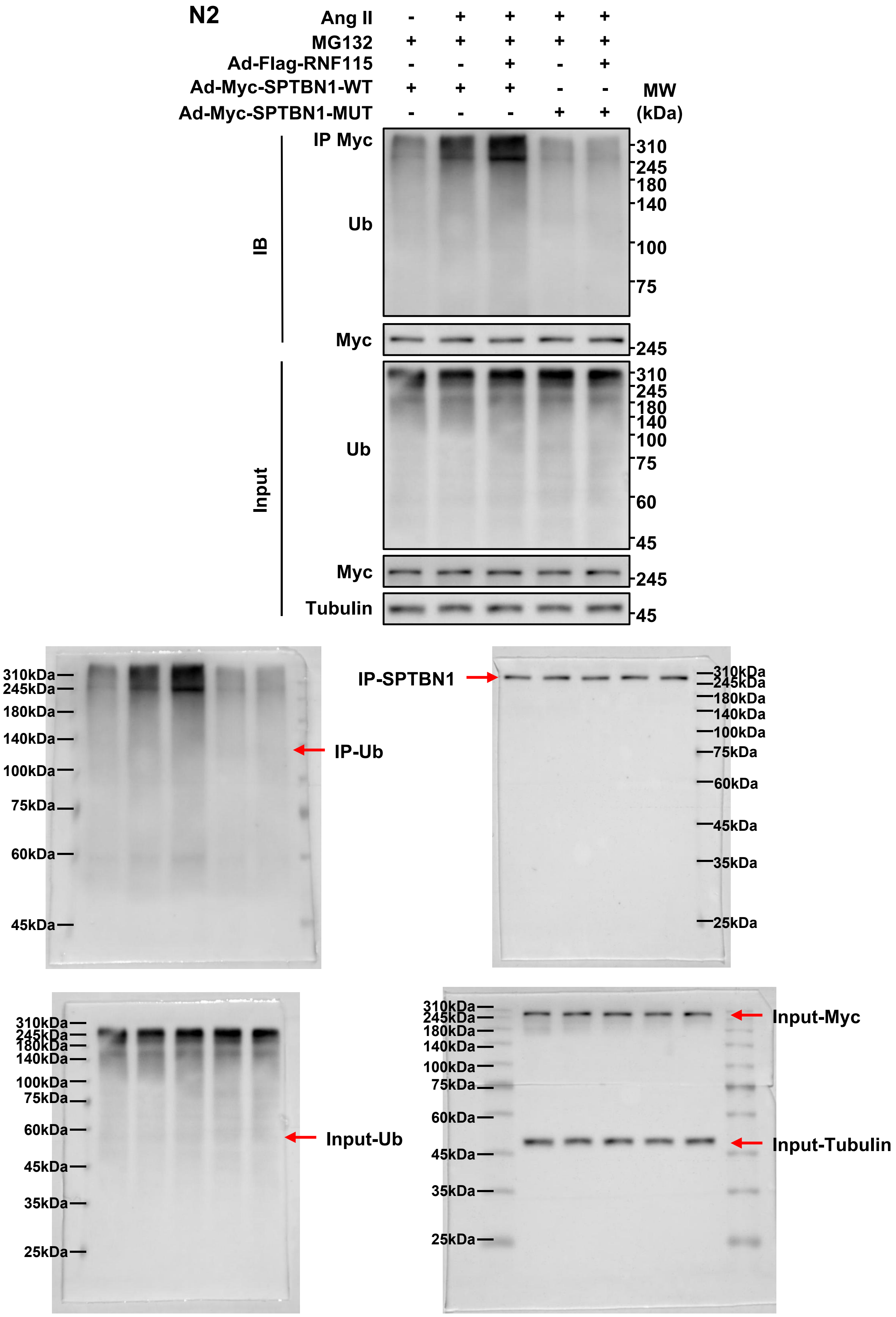
N2
Ang II - + + + +
MG132 + + + + +
Ad-Flag-RNF115 - - + - +
Ad-Myc-SPTBN1-WT + + + - -
Ad-Myc-SPTBN1-MUT - - - + +
MW (kDa)
IB
IP Myc
Ub
310
245
180
140
100
75
Myc
245
Input
Ub
310
245
180
140
100
75
60
45
Myc
245
Tubulin
45
310kDa
245kDa
180kDa
140kDa
100kDa
75kDa
60kDa
45kDa
IP-Ub
IP-SPTBN1
310kDa
245kDa
180kDa
140kDa
100kDa
75kDa
60kDa
45kDa
35kDa
25kDa
310kDa
245kDa
180kDa
140kDa
100kDa
75kDa
60kDa
45kDa
35kDa
25kDa
Input-Ub
310kDa
245kDa
180kDa
140kDa
100kDa
75kDa
60kDa
45kDa
35kDa
25kDa
Input-Myc
Input-Tubulin

Supplementary Figure5B

n=3 N1

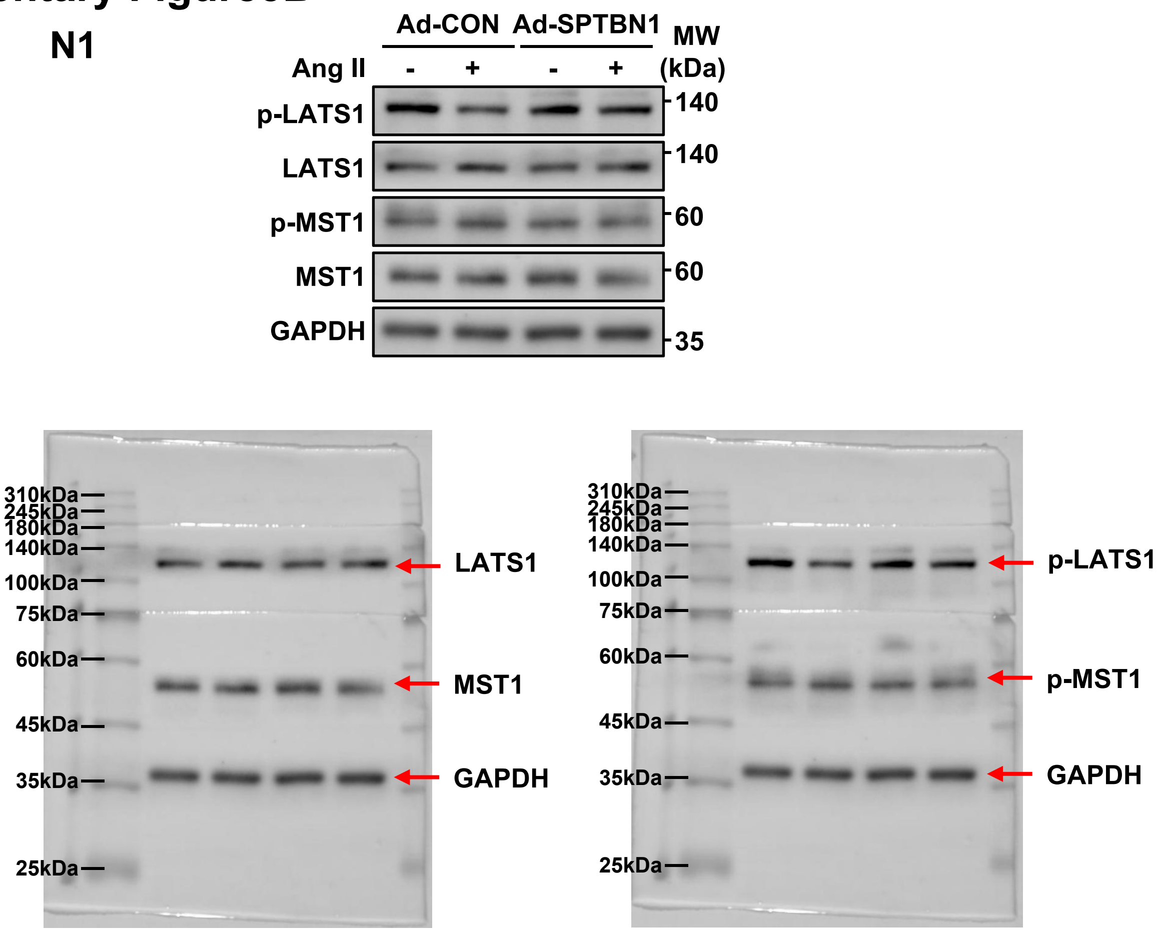

N2

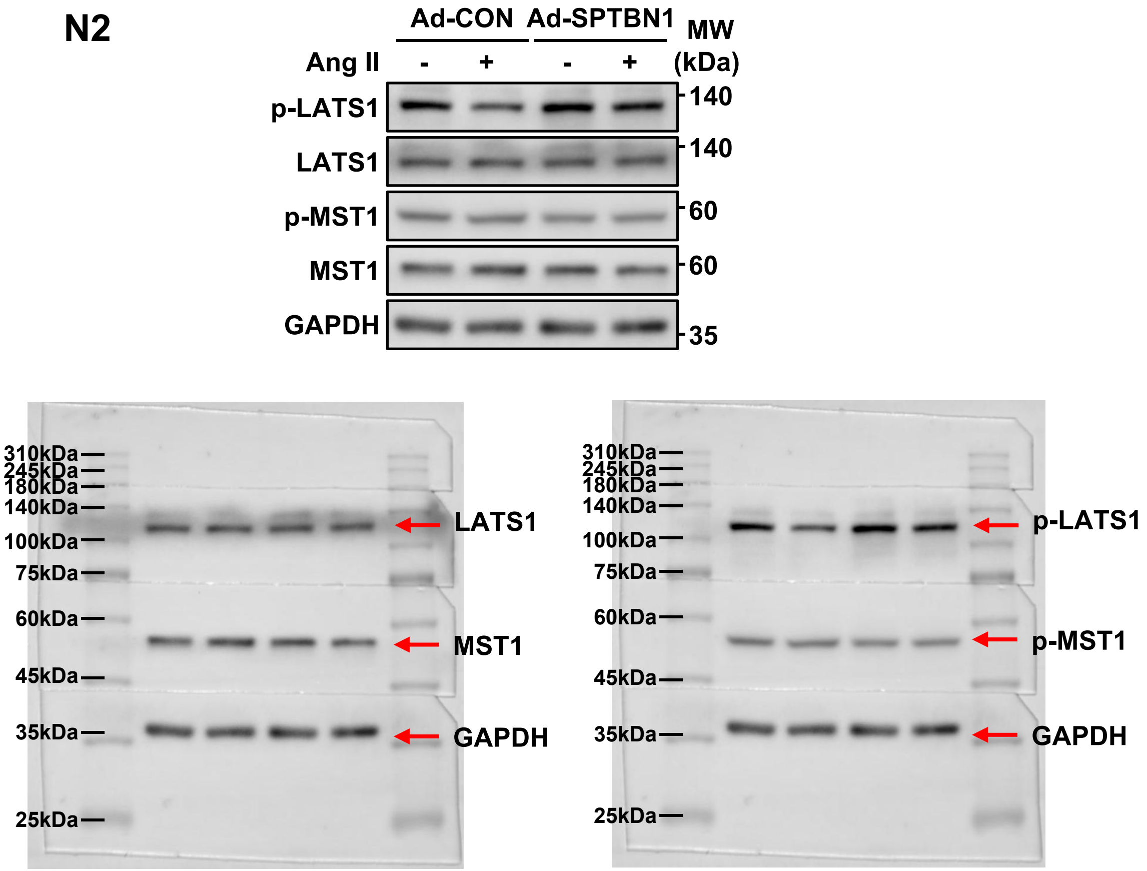

N3

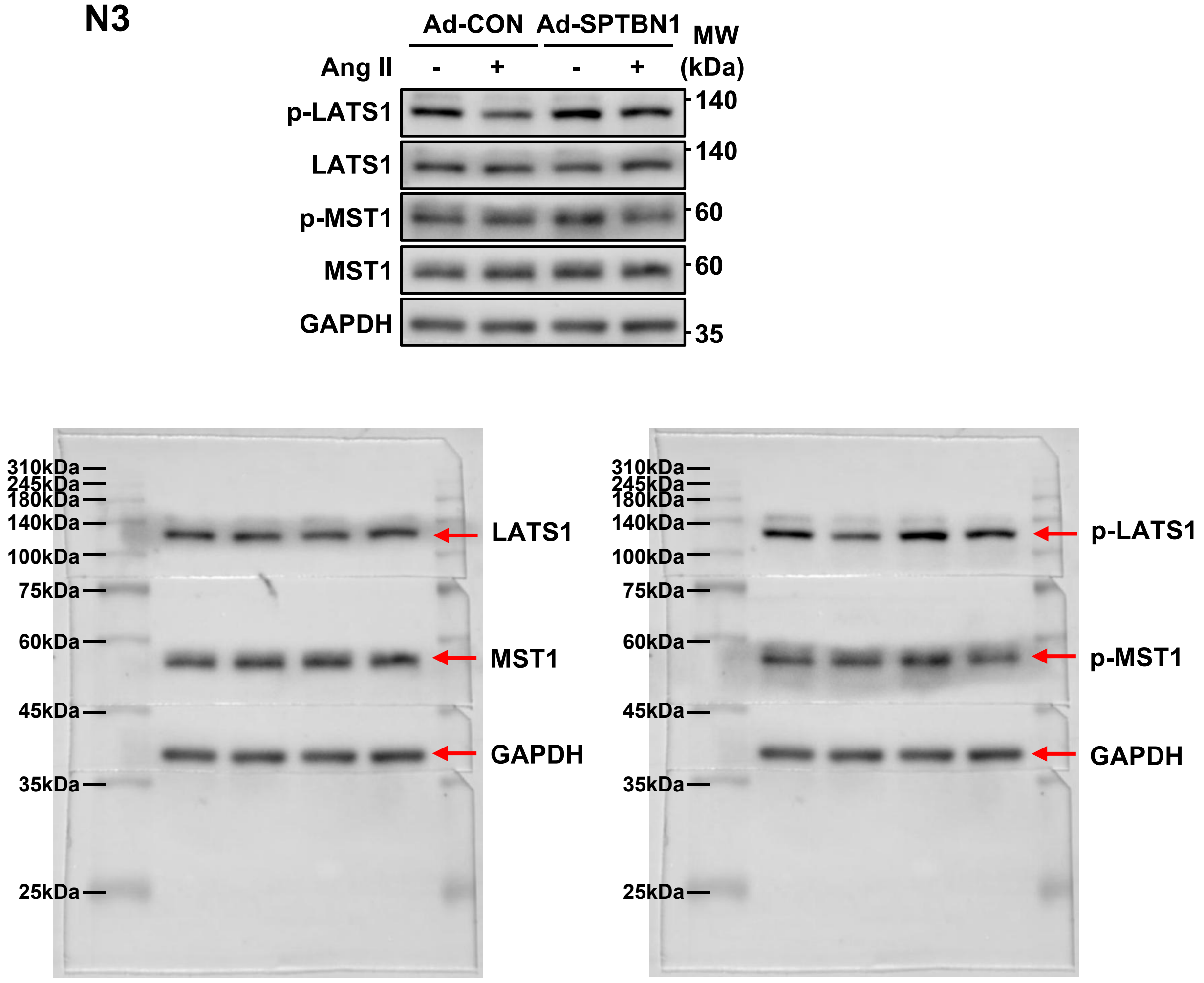

Ad-CON
Ad-SPTBN1
MW (kDa)
Ang II
-
+
-
+
p-LATS1
LATS1
p-MST1
MST1
GAPDH
140
140
60
60
35
310kDa
245kDa
180kDa
140kDa
100kDa
75kDa
60kDa
45kDa
35kDa
25kDa
LATS1
MST1
GAPDH
p-LATS1
p-MST1
GAPDH

Supplementary Figure5C

n=3

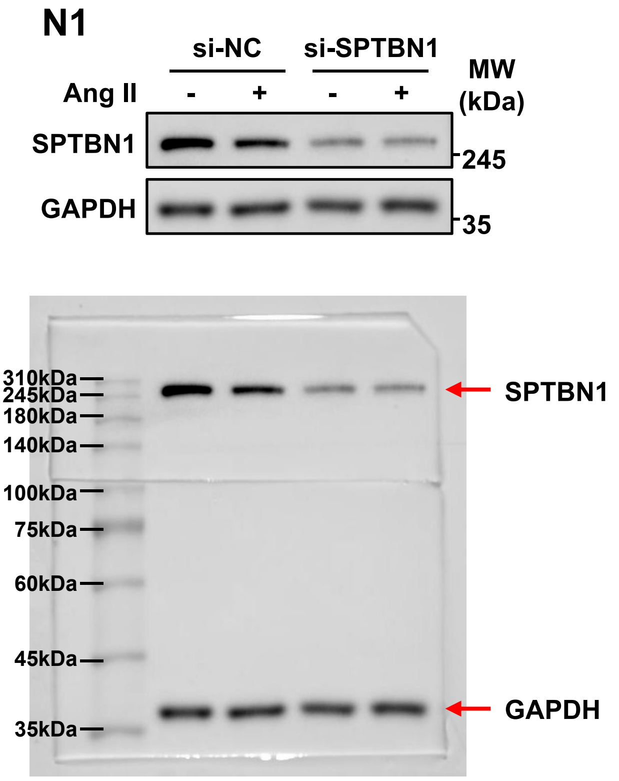

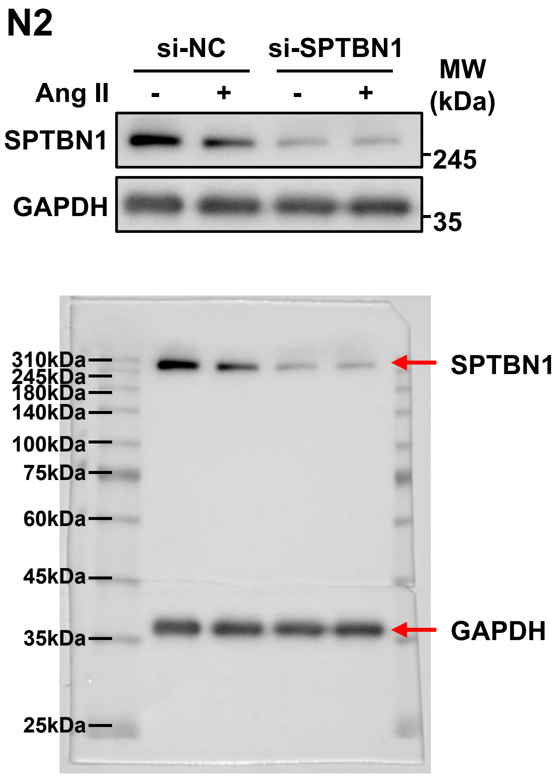

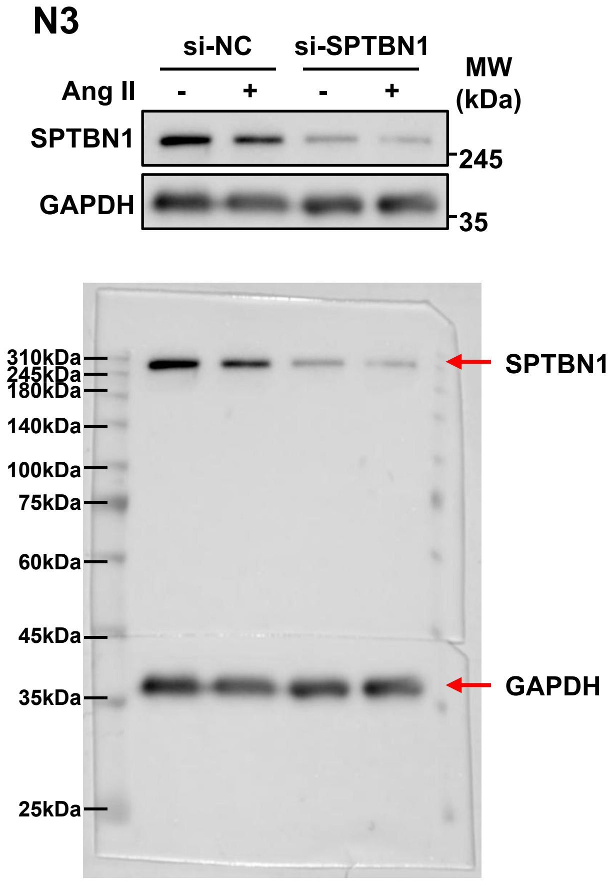

Supplementary Figure5D

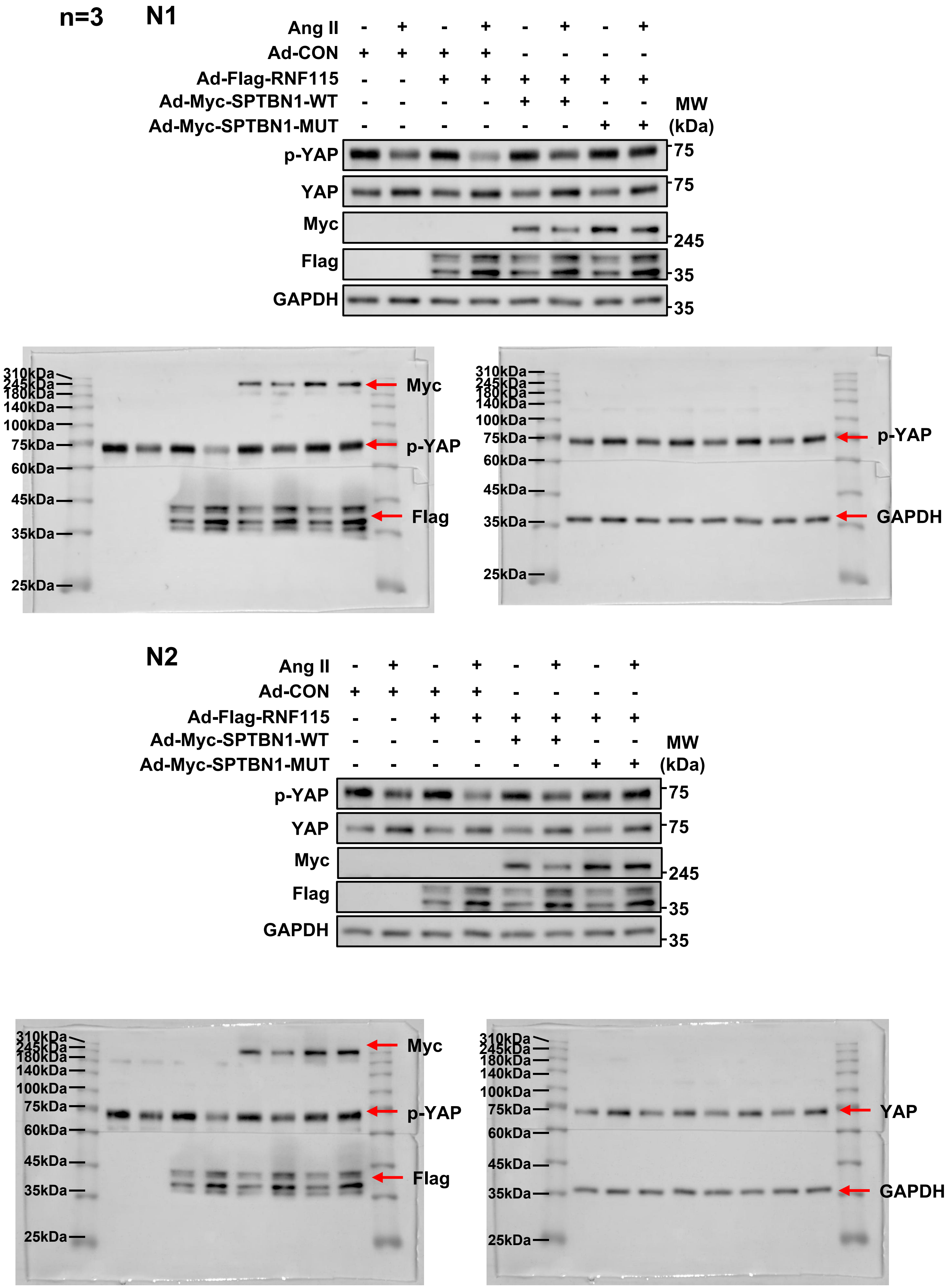

N3

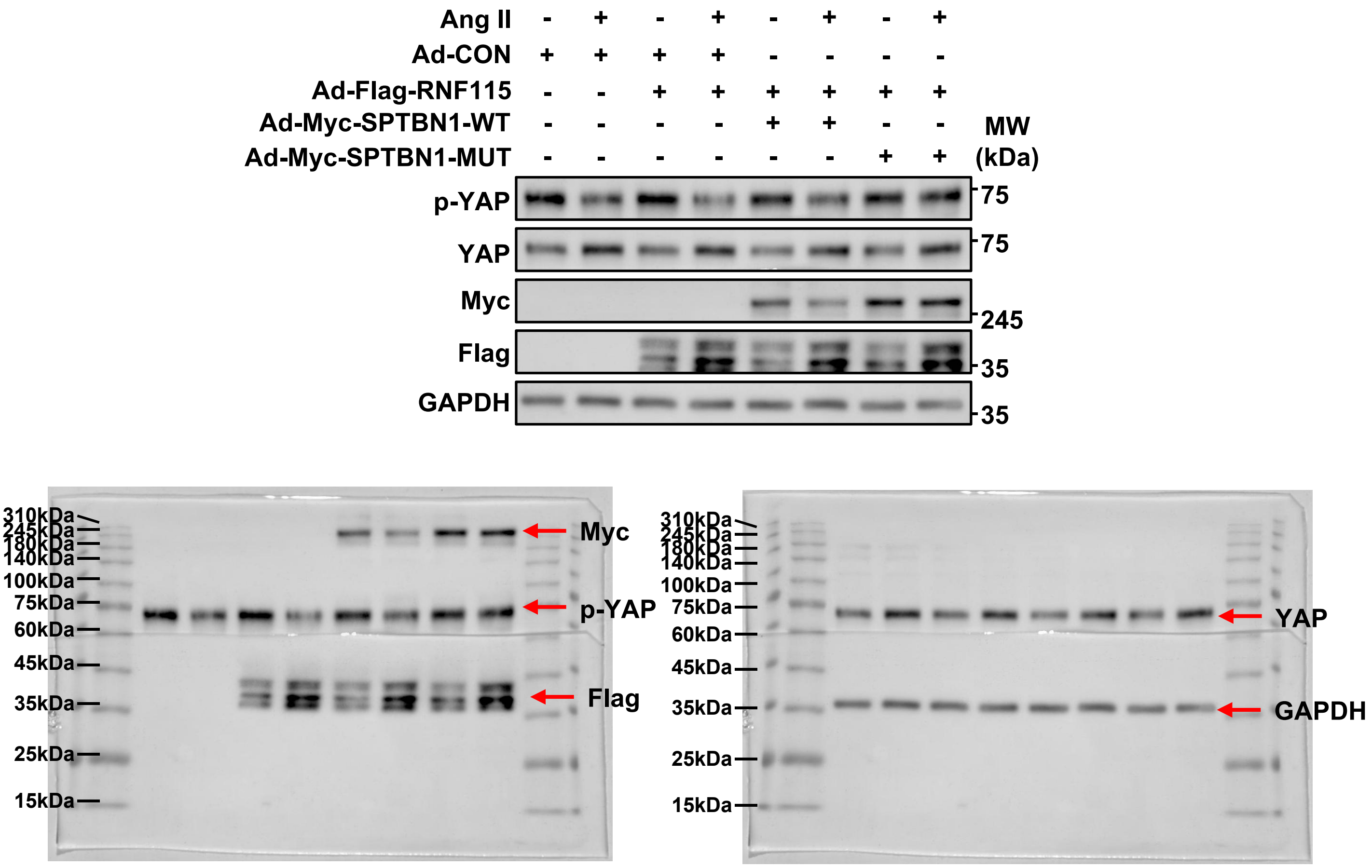

Ang II - + - + - + - +
Ad-CON + + + + - - - -
Ad-Flag-RNF115 - - + + + + + +
Ad-Myc-SPTBN1-WT - - - - + + - -
Ad-Myc-SPTBN1-MUT - - - - - - + +
MW
(kDa)
p-YAP
75
YAP
75
Myc
245
Flag
35
GAPDH
35
310kDa
245kDa
180kDa
140kDa
100kDa
75kDa
60kDa
45kDa
35kDa
25kDa
15kDa
Myc
p-YAP
Flag
310kDa
245kDa
180kDa
140kDa
100kDa
75kDa
60kDa
45kDa
35kDa
25kDa
15kDa
YAP
GAPDH

Supplementary Figure5E

N1

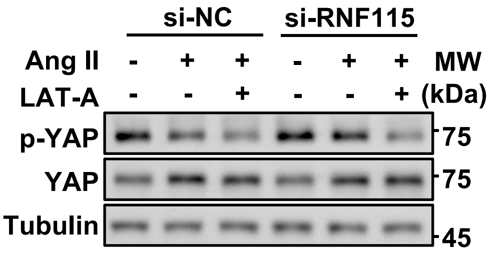

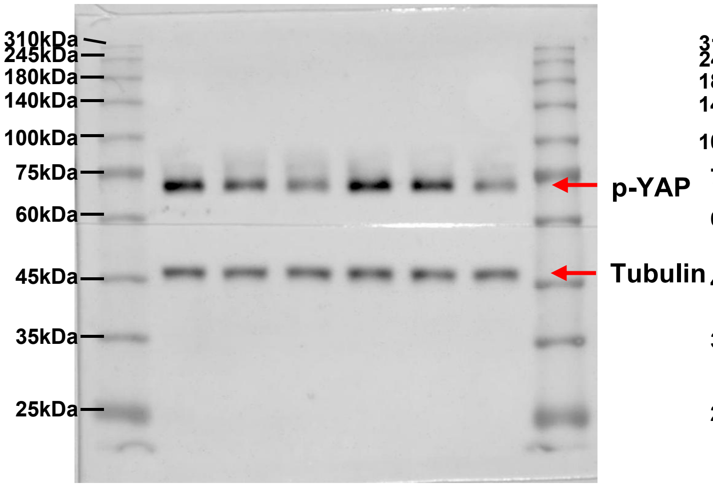

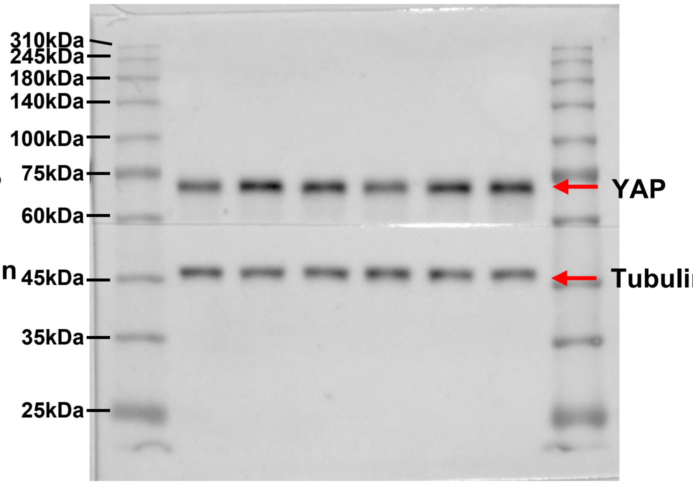

N2

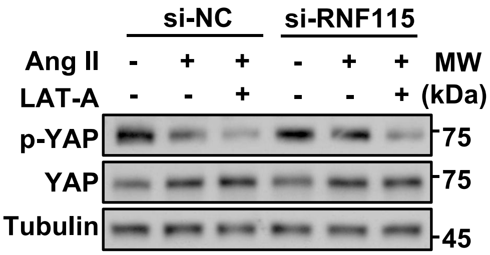

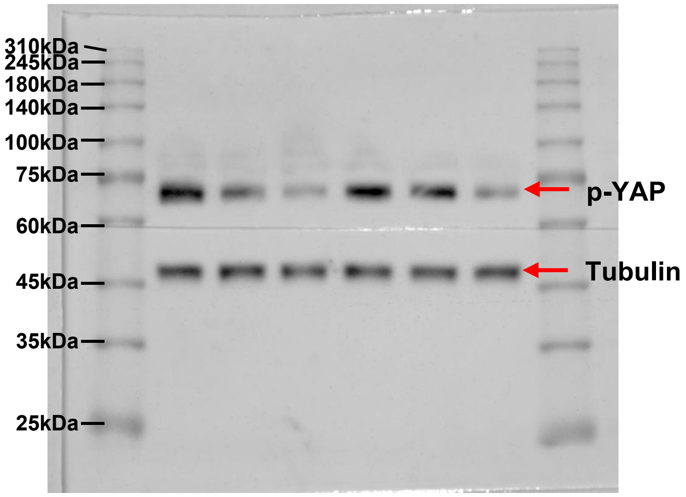

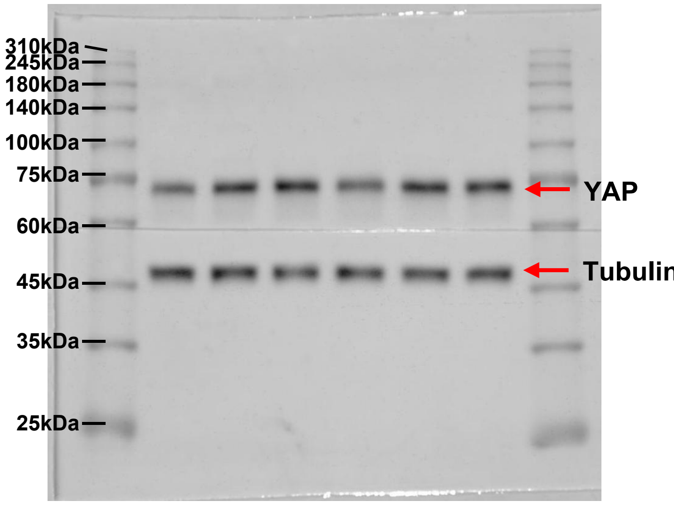

N3

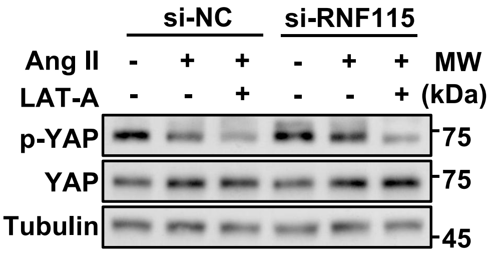
si-NC
si-RNF115
Ang II - + + - + +
LAT-A - - + - - +
MW
(kDa)
p-YAP
YAP
Tubulin
75
75
45

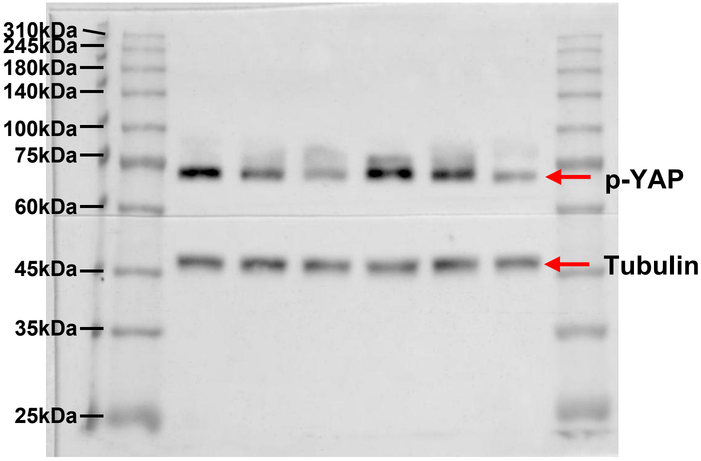
310kDa
245kDa
180kDa
140kDa
100kDa
75kDa
60kDa
45kDa
35kDa
25kDa
p-YAP
Tubulin

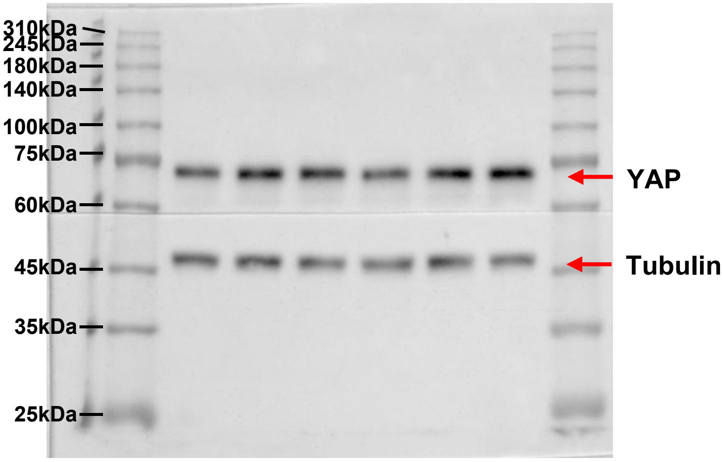
310kDa
245kDa
180kDa
140kDa
100kDa
75kDa
60kDa
45kDa
35kDa
25kDa
YAP
Tubulin

Supplementary Figure7J

n=3

N1

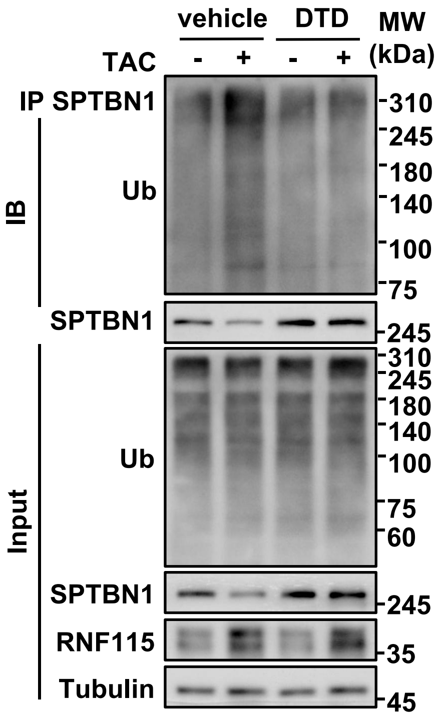

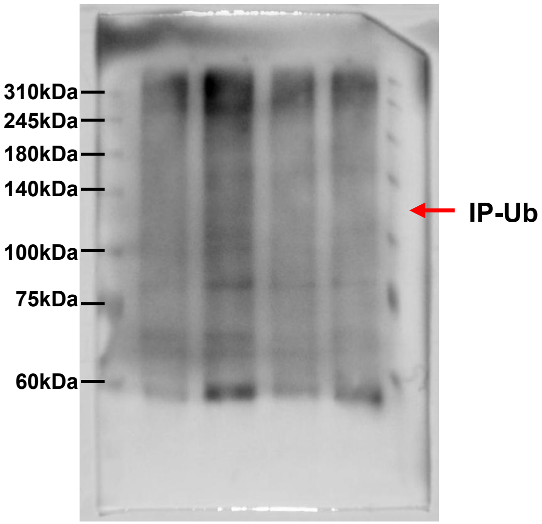

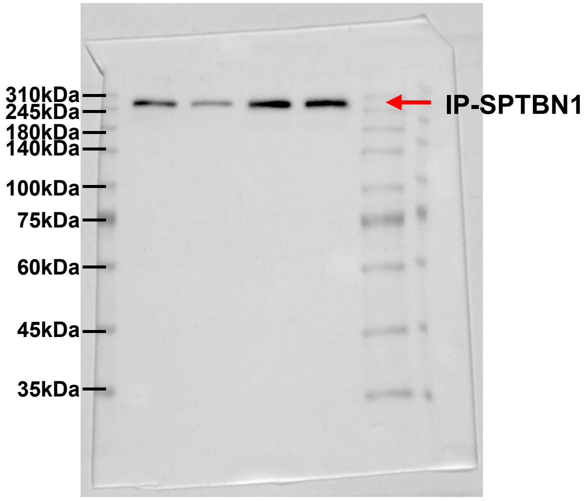

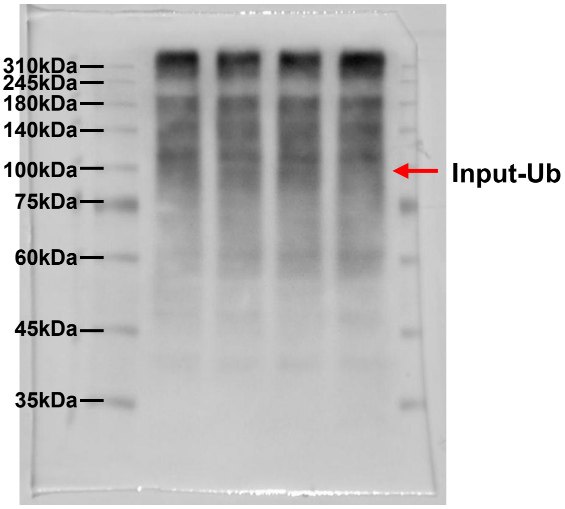

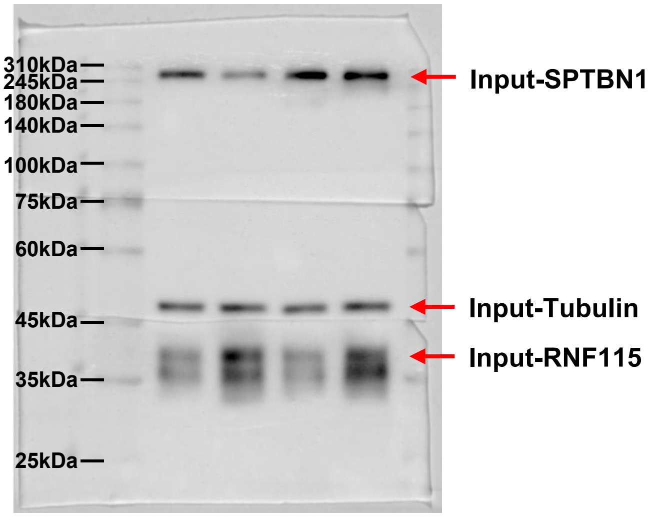

N2

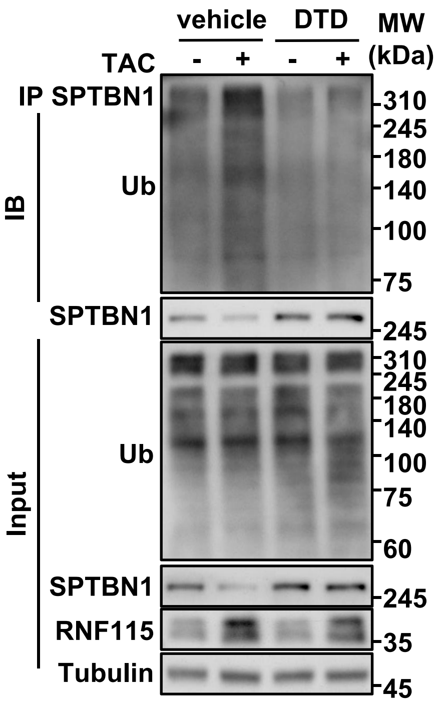
vehicle
DTD
MW (kDa)
TAC - + - +
IP SPTBN1
IB
Ub
310
245
180
140
100
75
SPTBN1
245
Input
Ub
310
245
180
140
100
75
60
SPTBN1
245
RNF115
35
Tubulin
45

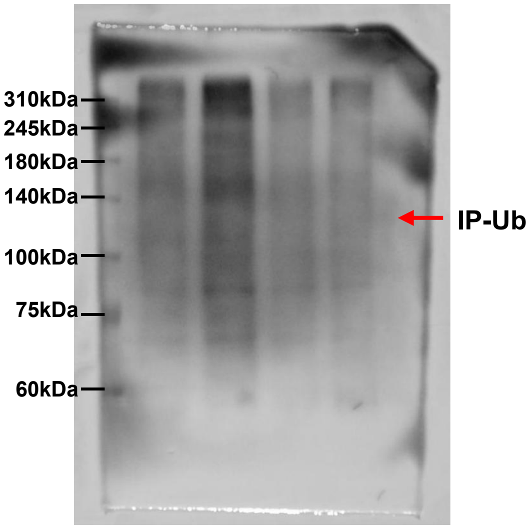
310kDa
245kDa
180kDa
140kDa
100kDa
75kDa
60kDa
IP-Ub

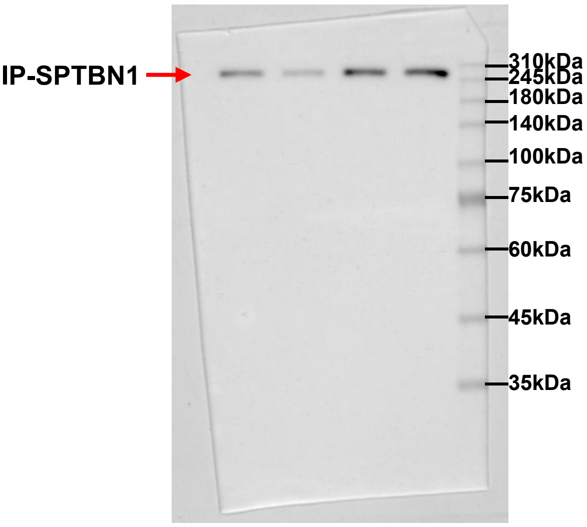
IP-SPTBN1
310kDa
245kDa
180kDa
140kDa
100kDa
75kDa
60kDa
45kDa
35kDa

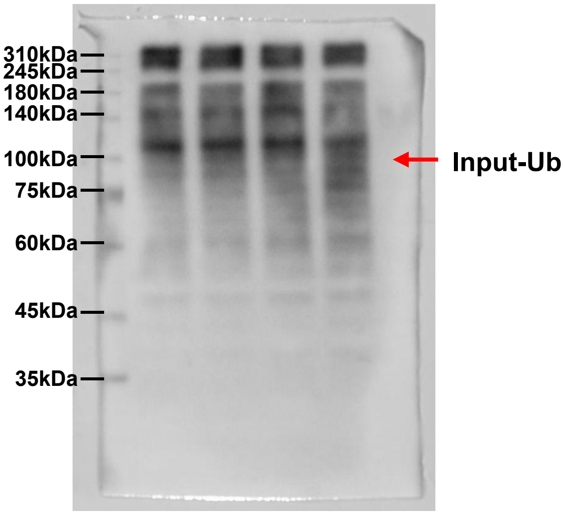
310kDa
245kDa
180kDa
140kDa
100kDa
75kDa
60kDa
45kDa
35kDa
Input-Ub

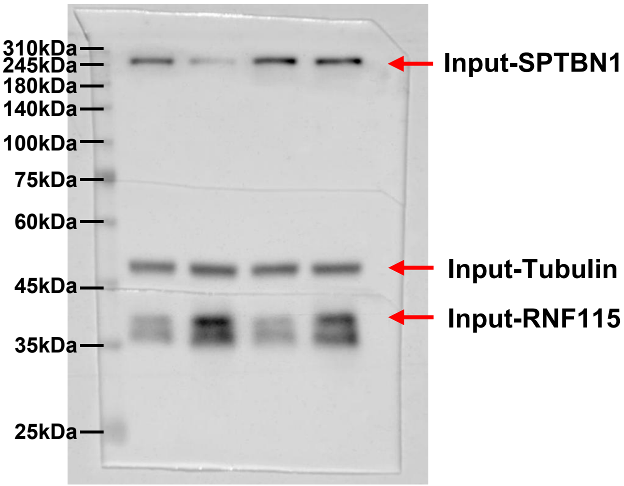
310kDa
245kDa
180kDa
140kDa
100kDa
75kDa
60kDa
45kDa
35kDa
25kDa
Input-SPTBN1
Input-Tubulin
Input-RNF115

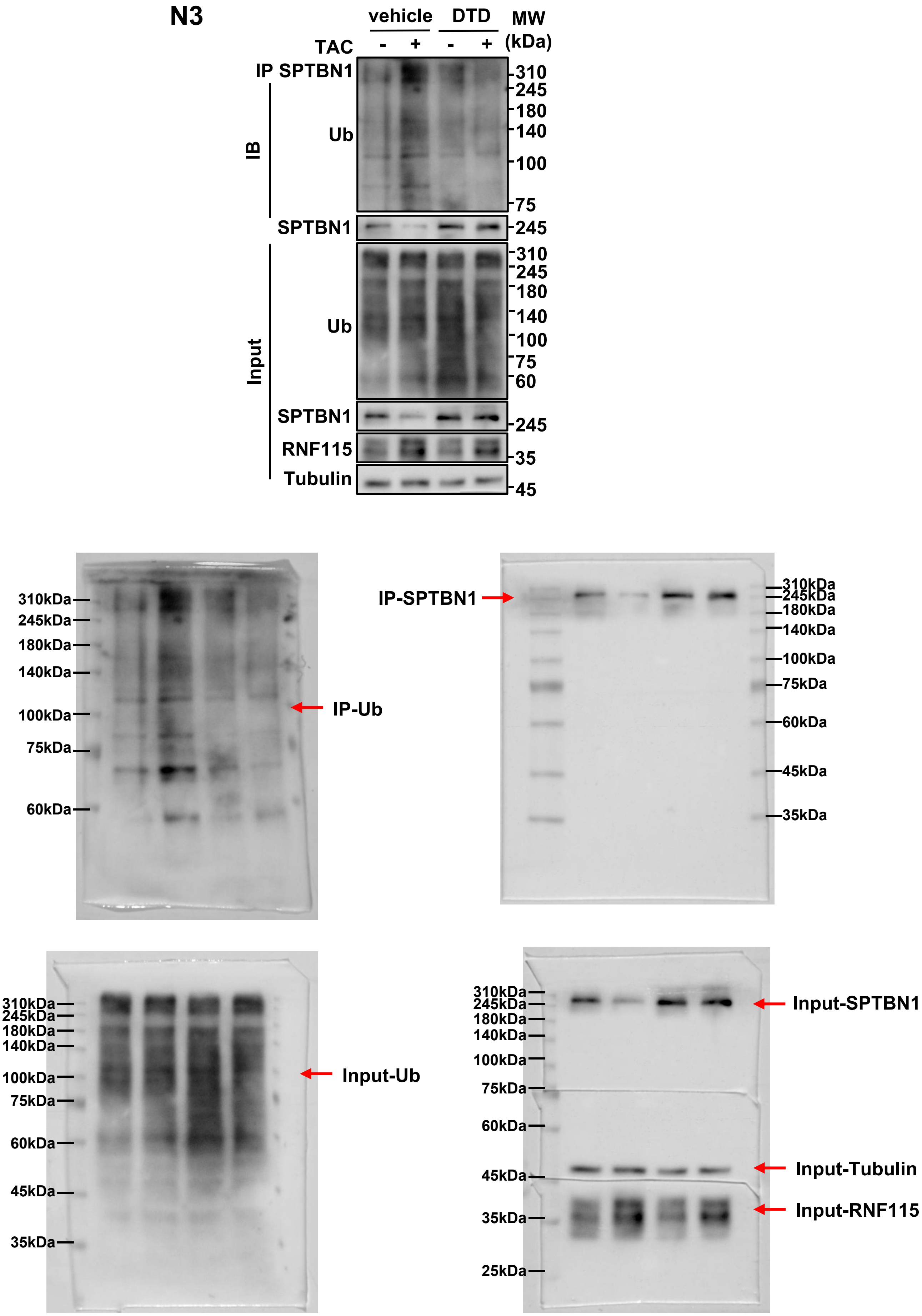
N3
vehicle
DTD
MW
(kDa)
TAC
- + - +
IP SPTBN1
IB
Ub
SPTBN1
Input
Ub
SPTBN1
RNF115
Tubulin
310
245
180
140
100
75
245
310
245
180
140
100
75
60
245
35
45
310kDa
245kDa
180kDa
140kDa
100kDa
75kDa
60kDa
IP-Ub
IP-SPTBN1
310kDa
245kDa
180kDa
140kDa
100kDa
75kDa
60kDa
45kDa
35kDa
310kDa
245kDa
180kDa
140kDa
100kDa
75kDa
60kDa
45kDa
35kDa
Input-Ub
310kDa
245kDa
180kDa
140kDa
100kDa
75kDa
60kDa
45kDa
35kDa
25kDa
Input-SPTBN1
Input-Tubulin
Input-RNF115

Response Figure2C

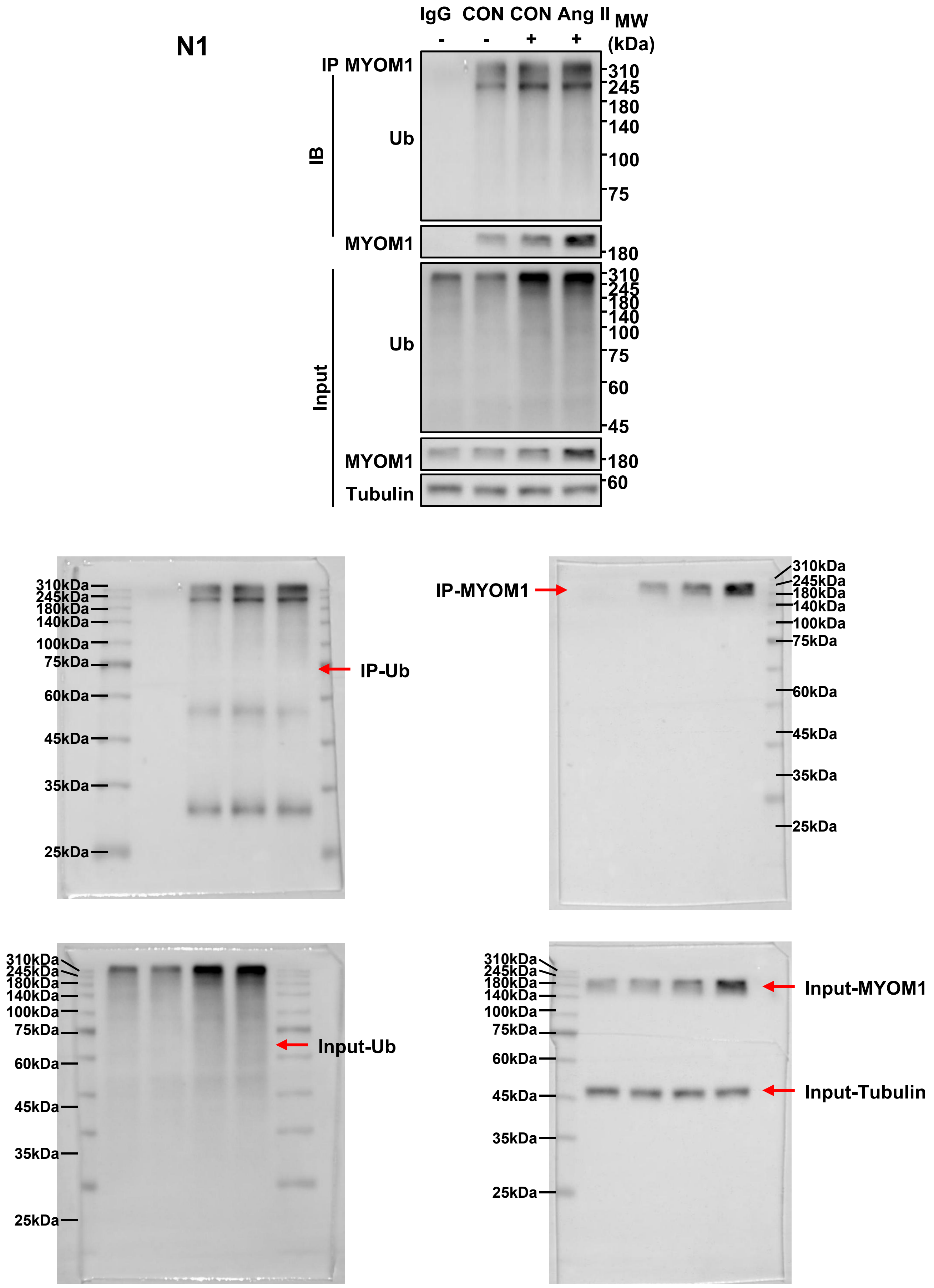

N2

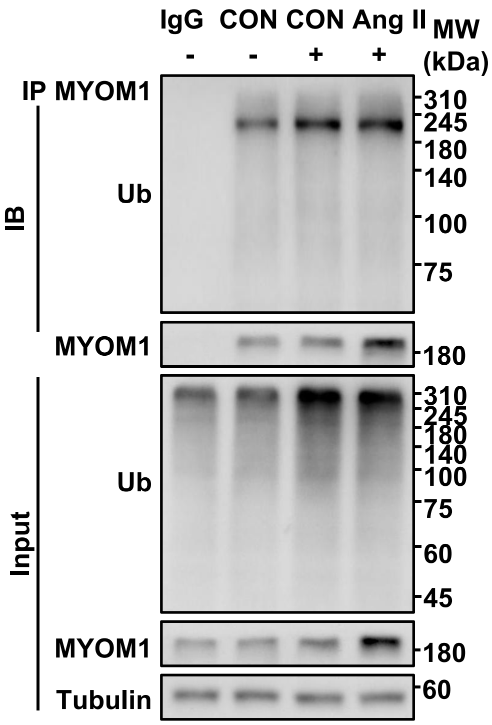
IgG CON CON Ang II
- - + +
MW (kDa)
IP MYOM1
IB
Ub
310
245
180
140
100
75
MYOM1
180
Input
Ub
310
245
180
140
100
75
60
45
MYOM1
180
Tubulin
60

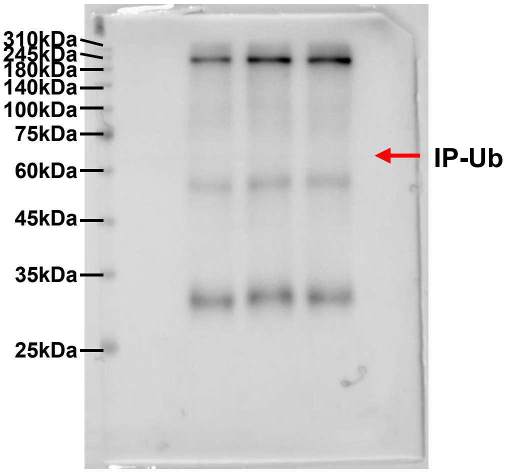
310kDa
245kDa
180kDa
140kDa
100kDa
75kDa
60kDa
45kDa
35kDa
25kDa
IP-Ub

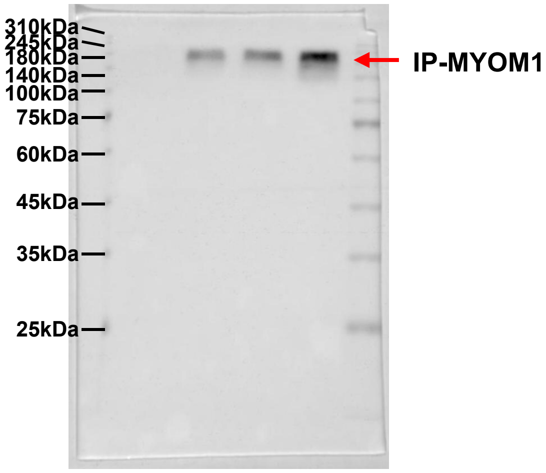
310kDa
245kDa
180kDa
140kDa
100kDa
75kDa
60kDa
45kDa
35kDa
25kDa
IP-MYOM1

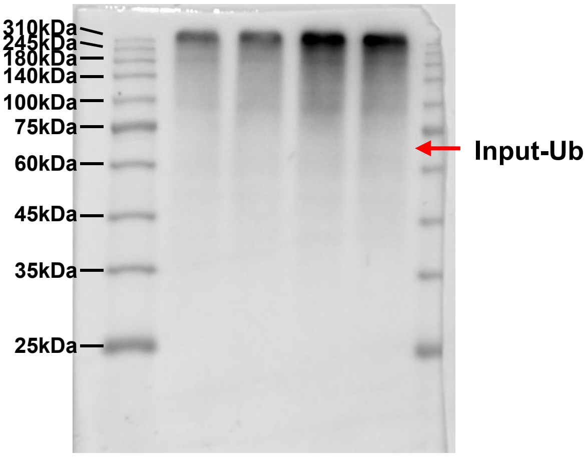
310kDa
245kDa
180kDa
140kDa
100kDa
75kDa
60kDa
45kDa
35kDa
25kDa
Input-Ub

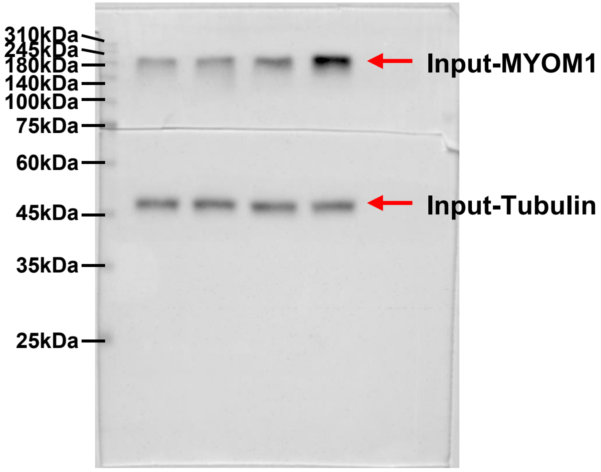
310kDa
245kDa
180kDa
140kDa
100kDa
75kDa
60kDa
45kDa
35kDa
25kDa
Input-MYOM1
Input-Tubulin

N3

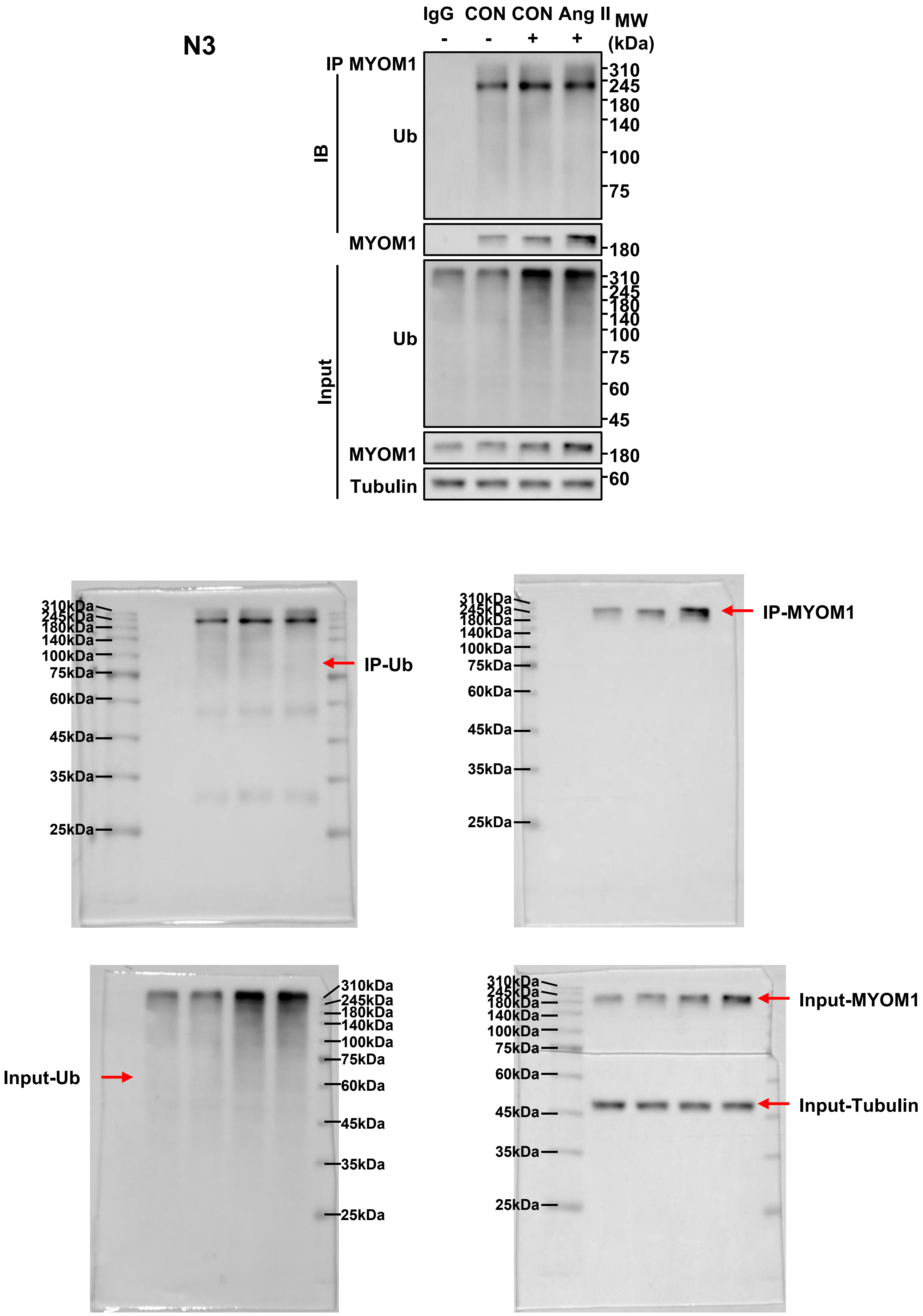
IgG CON CON Ang II
- - + +
MW (kDa)
IP MYOM1
IB
Ub
310
245
180
140
100
75
MYOM1
180
Input
Ub
310
245
180
140
100
75
60
45
MYOM1
180
Tubulin
60
310kDa
245kDa
180kDa
140kDa
100kDa
75kDa
60kDa
45kDa
35kDa
25kDa
IP-Ub
IP-MYOM1
Input-Ub
Input-MYOM1
Input-Tubulin

Response Figure2D

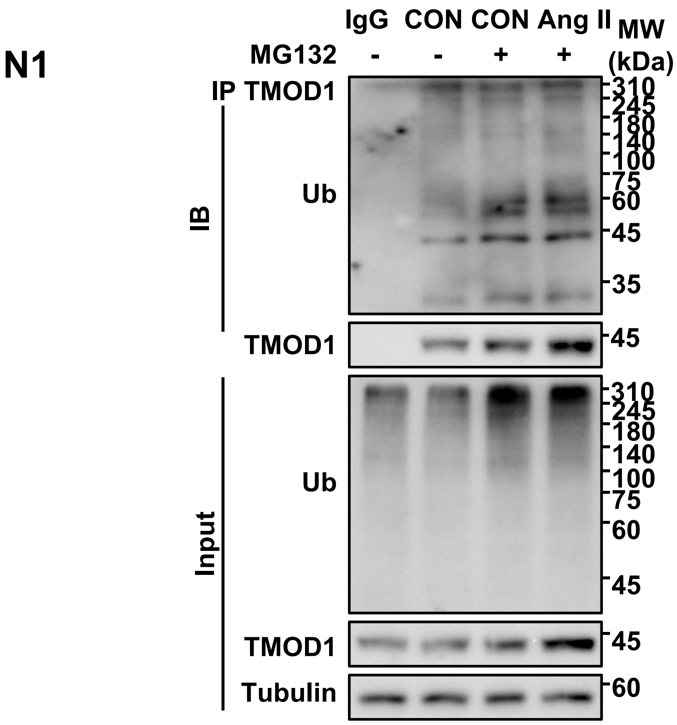

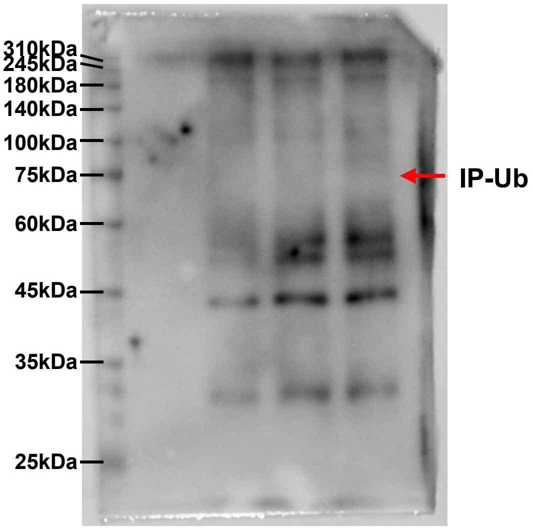

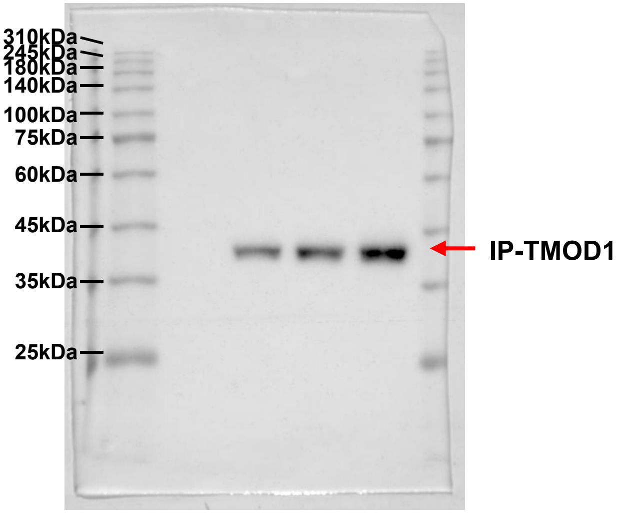

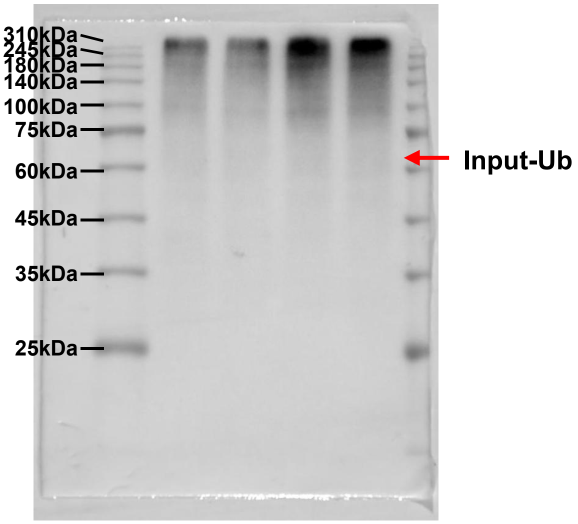

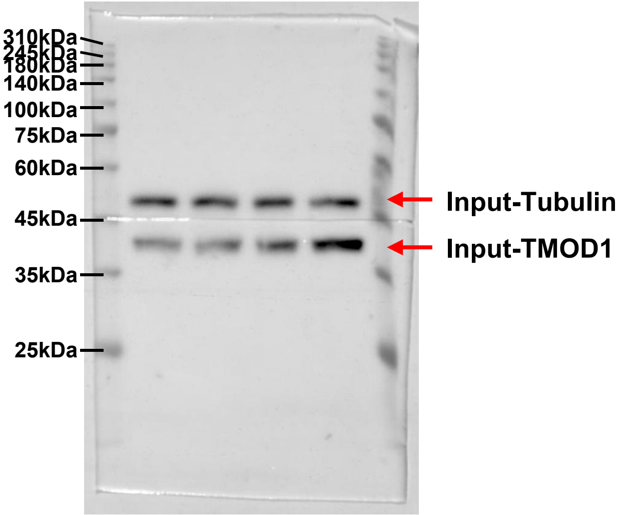

N2

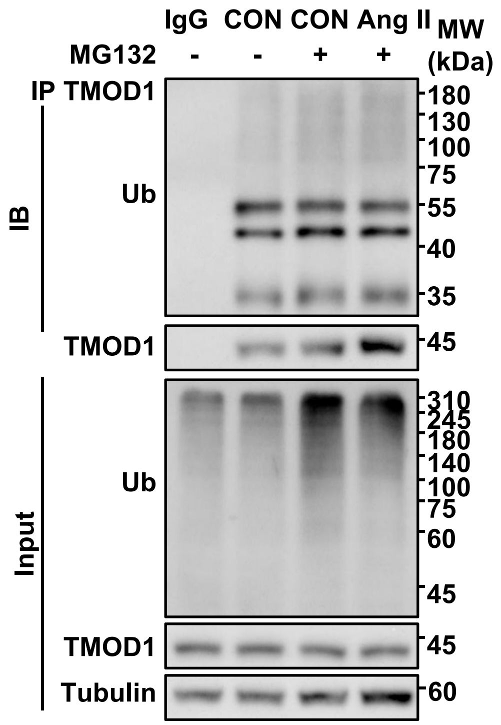
IgG CON CON Ang II
MG132 - - + +
MW (kDa)
IP TMOD1
IB
Ub
180
130
100
75
55
40
35
TMOD1
45
Input
Ub
310
245
180
140
100
75
60
45
TMOD1
45
Tubulin
60

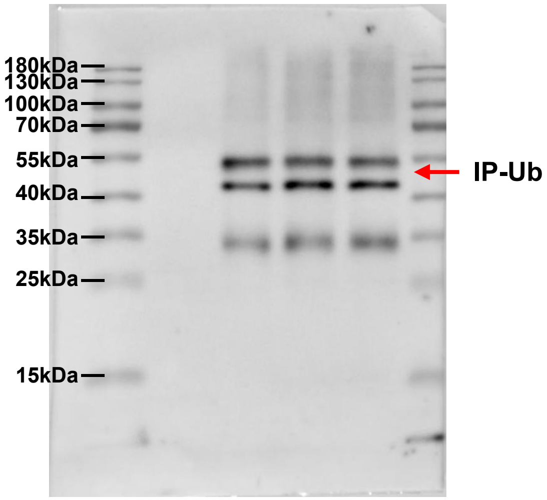
180kDa
130kDa
100kDa
70kDa
55kDa
40kDa
35kDa
25kDa
15kDa
IP-Ub

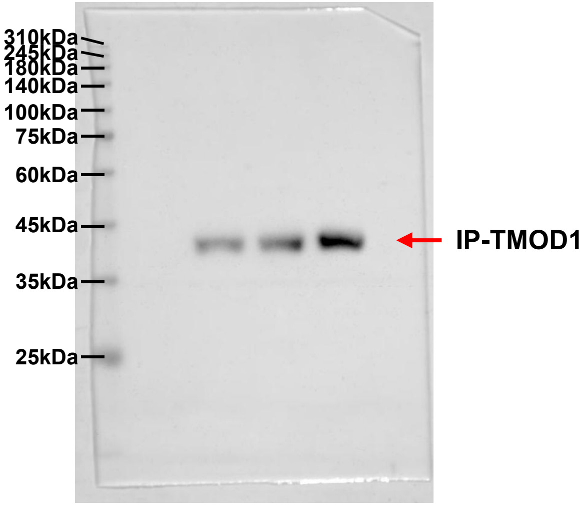
310kDa
245kDa
180kDa
140kDa
100kDa
75kDa
60kDa
45kDa
35kDa
25kDa
IP-TMOD1

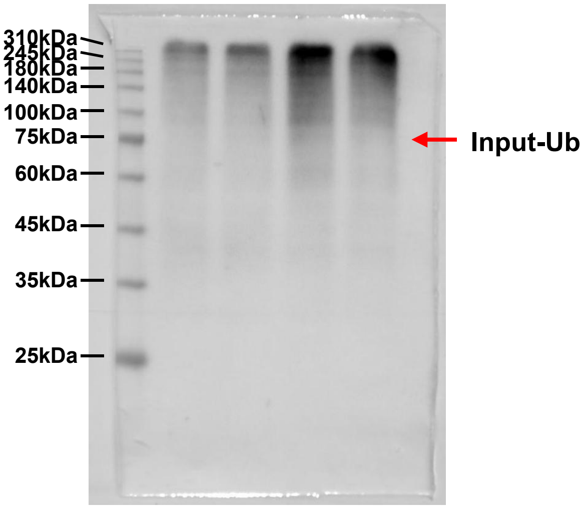
310kDa
245kDa
180kDa
140kDa
100kDa
75kDa
60kDa
45kDa
35kDa
25kDa
Input-Ub

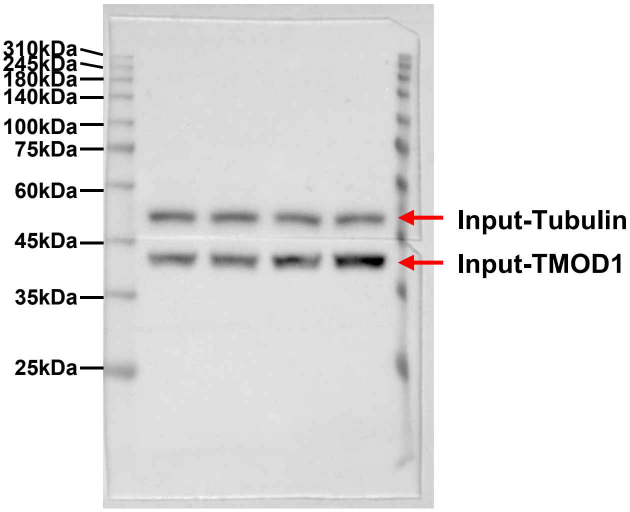
310kDa
245kDa
180kDa
140kDa
100kDa
75kDa
60kDa
45kDa
35kDa
25kDa
Input-Tubulin
Input-TMOD1

N3

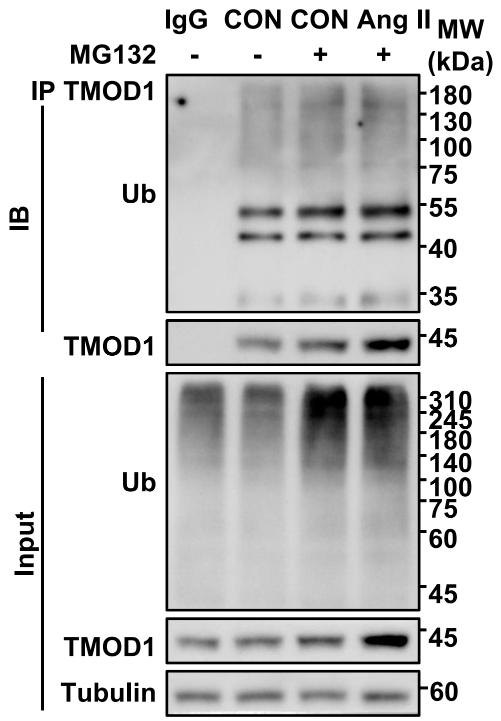
IgG
CON
CON
Ang II
MW
(kDa)
MG132
-
-
+
+
IP TMOD1
IB
Ub
180
130
100
75
55
40
35
TMOD1
45
Input
Ub
310
245
180
140
100
75
60
45
TMOD1
45
Tubulin
60

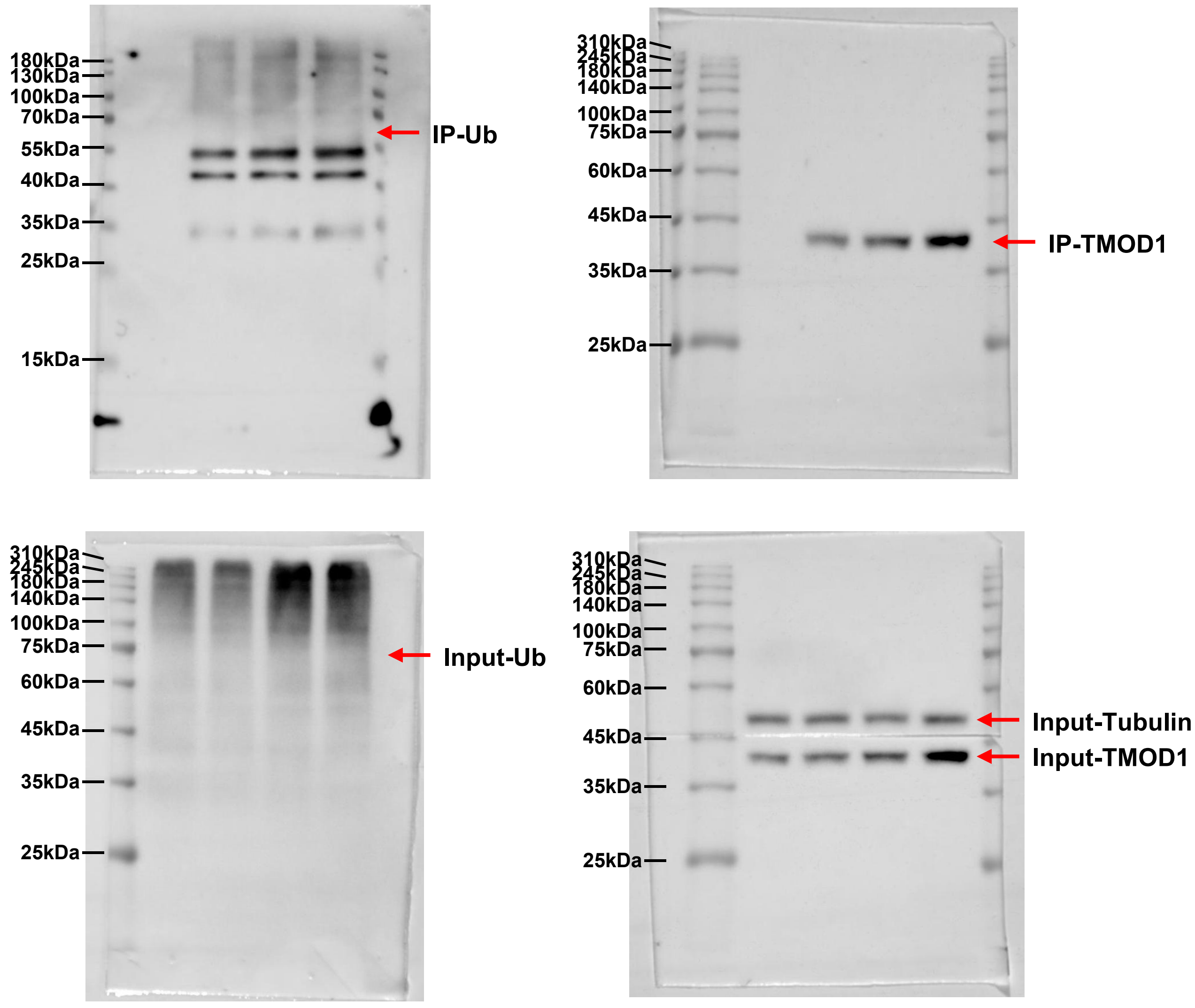
180kDa
130kDa
100kDa
70kDa
55kDa
40kDa
35kDa
25kDa
15kDa
IP-Ub
310kDa
245kDa
180kDa
140kDa
100kDa
75kDa
60kDa
45kDa
35kDa
25kDa
IP-TMOD1
310kDa
245kDa
180kDa
140kDa
100kDa
75kDa
60kDa
45kDa
35kDa
25kDa
Input-Ub
310kDa
245kDa
180kDa
140kDa
100kDa
75kDa
60kDa
45kDa
35kDa
25kDa
Input-Tubulin
Input-TMOD1

Response Figure2E

N1

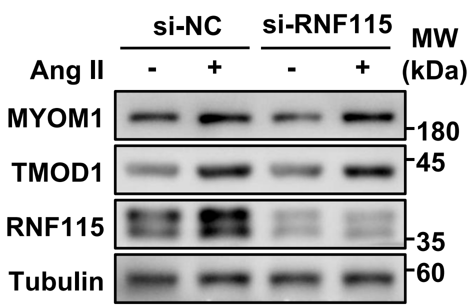

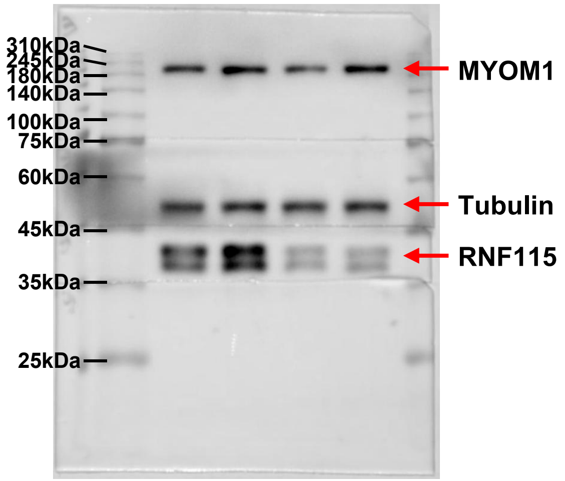

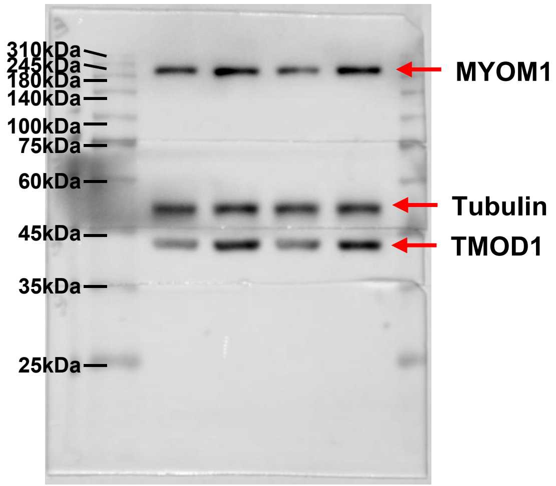

N2

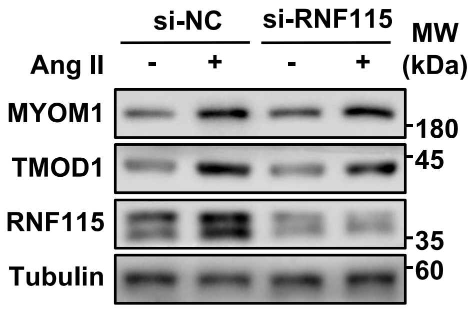

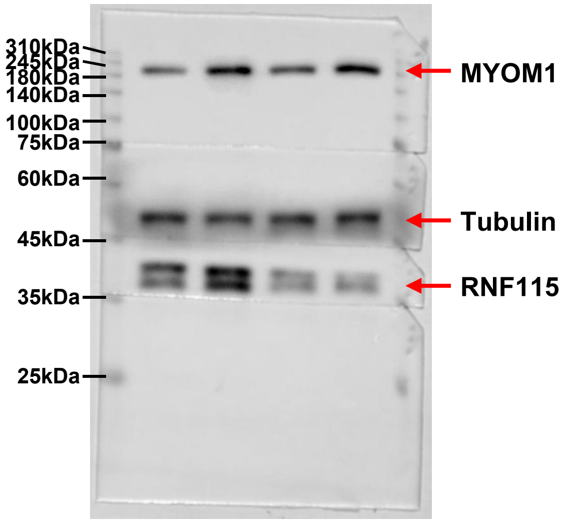

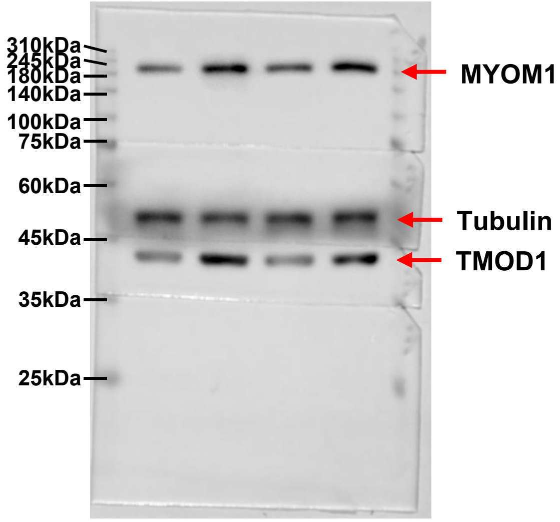

N3

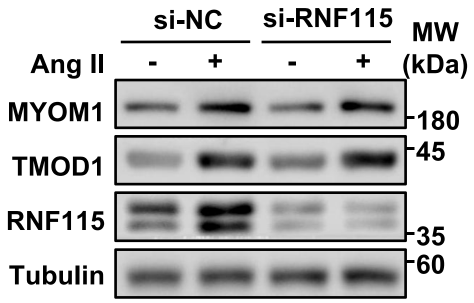
si-NC
si-RNF115
MW
(kDa)
Ang II
-
+
-
+
MYOM1
TMOD1
RNF115
Tubulin
180
45
35
60

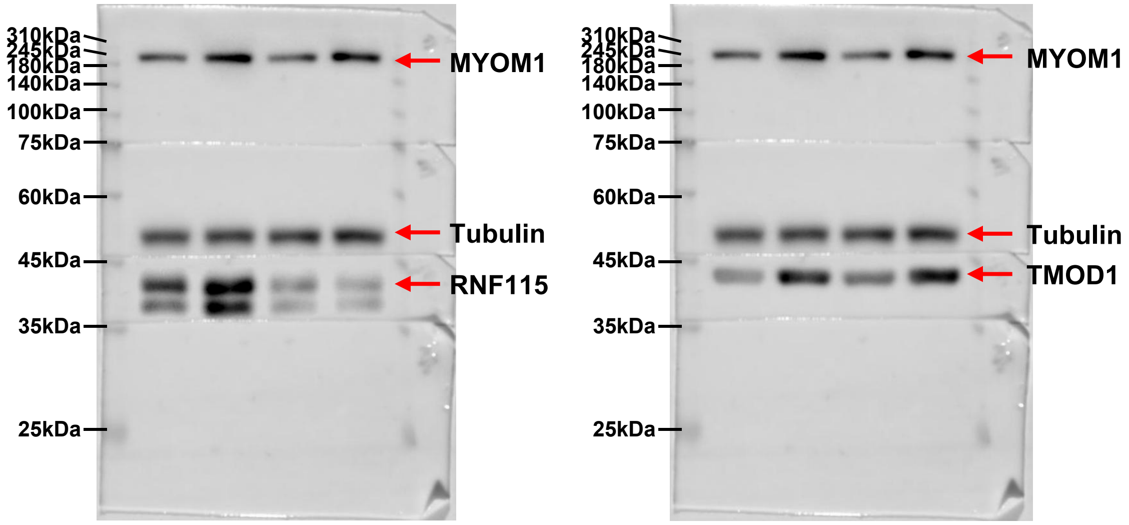
310kDa
245kDa
180kDa
140kDa
100kDa
75kDa
60kDa
45kDa
35kDa
25kDa
MYOM1
Tubulin
RNF115
310kDa
245kDa
180kDa
140kDa
100kDa
75kDa
60kDa
45kDa
35kDa
25kDa
MYOM1
Tubulin
TMOD1
